# Nicotine receptor partial agonists for smoking cessation

**DOI:** 10.1002/14651858.CD006103.pub8

**Published:** 2023-05-05

**Authors:** Jonathan Livingstone-Banks, Thomas R Fanshawe, Kyla H Thomas, Annika Theodoulou, Anisa Hajizadeh, Lilian Hartman, Nicola Lindson

**Affiliations:** Nuffield Department of Primary Care Health SciencesUniversity of OxfordOxfordUK; School of Social and Community MedicineUniversity of BristolBristolUK; University of Oxford Medical SchoolJohn Radcliffe HospitalOxfordUK

## Abstract

**Background:**

Nicotine receptor partial agonists may help people to stop smoking by a combination of maintaining moderate levels of dopamine to counteract withdrawal symptoms (acting as an agonist) and reducing smoking satisfaction (acting as an antagonist). This is an update of a Cochrane Review first published in 2007.

**Objectives:**

To assess the effectiveness of nicotine receptor partial agonists, including varenicline and cytisine, for smoking cessation.

**Search methods:**

We searched the Cochrane Tobacco Addiction Group's Specialised Register in April 2022 for trials, using relevant terms in the title or abstract, or as keywords. The register is compiled from searches of CENTRAL, MEDLINE, Embase, and PsycINFO.

**Selection criteria:**

We included randomised controlled trials that compared the treatment drug with placebo, another smoking cessation drug, e‐cigarettes, or no medication. We excluded trials that did not report a minimum follow‐up period of six months from baseline.

**Data collection and analysis:**

We followed standard Cochrane methods. Our main outcome was abstinence from smoking at longest follow‐up using the most rigorous definition of abstinence, preferring biochemically validated rates where reported. We pooled risk ratios (RRs), using the Mantel‐Haenszel fixed‐effect model. We also reported the number of people reporting serious adverse events (SAEs).

**Main results:**

We included 75 trials of 45,049 people; 45 were new for this update. We rated 22 at low risk of bias, 18 at high risk, and 35 at unclear risk.

We found moderate‐certainty evidence (limited by heterogeneity) that cytisine helps more people to quit smoking than placebo (RR 1.30, 95% confidence interval (CI) 1.15 to 1.47; I^2^ = 83%; 4 studies, 4623 participants), and no evidence of a difference in the number reporting SAEs (RR 1.04, 95% CI 0.78 to 1.37; I^2^ = 0%; 3 studies, 3781 participants; low‐certainty evidence). SAE evidence was limited by imprecision. We found no data on neuropsychiatric or cardiac SAEs.

We found high‐certainty evidence that varenicline helps more people to quit than placebo (RR 2.32, 95% CI 2.15 to 2.51; I^2^ = 60%, 41 studies, 17,395 participants), and moderate‐certainty evidence that people taking varenicline are more likely to report SAEs than those not taking it (RR 1.23, 95% CI 1.01 to 1.48; I^2^ = 0%; 26 studies, 14,356 participants). While point estimates suggested increased risk of cardiac SAEs (RR 1.20, 95% CI 0.79 to 1.84; I^2^ = 0%; 18 studies, 7151 participants; low‐certainty evidence), and decreased risk of neuropsychiatric SAEs (RR 0.89, 95% CI 0.61 to 1.29; I^2^ = 0%; 22 studies, 7846 participants; low‐certainty evidence), in both cases evidence was limited by imprecision, and confidence intervals were compatible with both benefit and harm.

Pooled results from studies that randomised people to receive cytisine or varenicline showed that more people in the varenicline arm quit smoking (RR 0.83, 95% CI 0.66 to 1.05; I^2^ = 0%; 2 studies, 2131 participants; moderate‐certainty evidence) and reported SAEs (RR 0.67, 95% CI 0.44 to 1.03; I^2^ = 45%; 2 studies, 2017 participants; low‐certainty evidence). However, the evidence was limited by imprecision, and confidence intervals incorporated the potential for benefit from either cytisine or varenicline. We found no data on neuropsychiatric or cardiac SAEs.

We found high‐certainty evidence that varenicline helps more people to quit than bupropion (RR 1.36, 95% CI 1.25 to 1.49; I^2^ = 0%; 9 studies, 7560 participants), and no clear evidence of difference in rates of SAEs (RR 0.89, 95% CI 0.61 to 1.31; I^2^ = 0%; 5 studies, 5317 participants), neuropsychiatric SAEs (RR 1.05, 95% CI 0.16 to 7.04; I^2^ = 10%; 2 studies, 866 participants), or cardiac SAEs (RR 3.17, 95% CI 0.33 to 30.18; I^2^ = 0%; 2 studies, 866 participants). Evidence of harms was of low certainty, limited by imprecision.

We found high‐certainty evidence that varenicline helps more people to quit than a single form of nicotine replacement therapy (NRT) (RR 1.25, 95% CI 1.14 to 1.37; I^2^ = 28%; 11 studies, 7572 participants), and low‐certainty evidence, limited by imprecision, of fewer reported SAEs (RR 0.70, 95% CI 0.50 to 0.99; I^2^ = 24%; 6 studies, 6535 participants). We found no data on neuropsychiatric or cardiac SAEs.

We found no clear evidence of a difference in quit rates between varenicline and dual‐form NRT (RR 1.02, 95% CI 0.87 to 1.20; I^2^ = 0%; 5 studies, 2344 participants; low‐certainty evidence, downgraded because of imprecision). While pooled point estimates suggested increased risk of SAEs (RR 2.15, 95% CI 0.49 to 9.46; I^2^ = 0%; 4 studies, 1852 participants) and neuropsychiatric SAEs (RR 4.69, 95% CI 0.23 to 96.50; I^2^ not estimable as events only in 1 study; 2 studies, 764 participants), and reduced risk of cardiac SAEs (RR 0.32, 95% CI 0.01 to 7.88; I^2^ not estimable as events only in 1 study; 2 studies, 819 participants), in all three cases evidence was of low certainty and confidence intervals were very wide, encompassing both substantial harm and benefit.

**Authors' conclusions:**

Cytisine and varenicline both help more people to quit smoking than placebo or no medication. Varenicline is more effective at helping people to quit smoking than bupropion, or a single form of NRT, and may be as or more effective than dual‐form NRT. People taking varenicline are probably more likely to experience SAEs than those not taking it, and while there may be increased risk of cardiac SAEs and decreased risk of neuropsychiatric SAEs, evidence was compatible with both benefit and harm. Cytisine may lead to fewer people reporting SAEs than varenicline. Based on studies that directly compared cytisine and varenicline, there may be a benefit from varenicline for quitting smoking, however further evidence could strengthen this finding or demonstrate a benefit from cytisine.

Future trials should test the effectiveness and safety of cytisine compared with varenicline and other pharmacotherapies, and should also test variations in dose and duration. There is limited benefit to be gained from more trials testing the effect of standard‐dose varenicline compared with placebo for smoking cessation. Further trials on varenicline should test variations in dose and duration, and compare varenicline with e‐cigarettes for smoking cessation.

## Plain language summary

**Can medications like varenicline and cytisine (nicotine receptor partial agonists) help people to stop smoking and do they cause unwanted effects?**

**Key messages**

· Varenicline can help people to stop smoking for at least 6 months. Evidence shows it works better than bupropion and using only one type of nicotine replacement therapy (e.g. only patches). Quit rates might be similar to using more than one type of nicotine replacement therapy at the same time (e.g. patches and gum together).

· Cytisine can help people to stop smoking for at least 6 months. It may work as well as varenicline, but future evidence may show that while it helps, it is not quite as helpful as varenicline.

· Future studies should test the effectiveness and safety of cytisine compared with varenicline and other stop‐smoking medications, and should also  investigate giving cytisine or varenicline at different doses and for different lengths of time.

**What are 'nicotine receptor partial agonists'?**

Smoking tobacco is extremely bad for people’s health. For people who smoke, quitting is the best thing they can do to improve their health. Many people find it difficult to quit smoking. Nicotine receptor partial agonists (NRPAs) are a type of medication used to help people to stop smoking. They help to reduce the withdrawal symptoms people experience when they stop smoking, like cravings and unpleasant mood changes. They also reduce the pleasure people usually experience when they smoke. The most widely‐available treatment in this drug type is varenicline. Cytisine is another, similar medication. They may cause unwanted effects such as feeling sick (nausea) and other stomach problems, difficulties sleeping, abnormal dreams, and headache. They may also lead to potentially serious unwanted effects, such as suicidal thoughts, heart problems and raised blood pressure.

**What did we want to find out?**

We wanted to find out if using NRPAs can help people to quit smoking, and if they cause unwanted effects. We wanted to know:

· how many people stopped smoking for at least 6 months; and

· how many people had unwanted effects.

**What did we do?**

We searched for studies that investigated NRPAs used to help people quit smoking. People in the studies had to be chosen at random to receive an NRPA, or another NRPA, placebo (medication like the NRPA but with no active ingredients) or no treatment. They had to be adult tobacco smokers who wanted to stop smoking.

**What did we find?**

We found 75 studies that compared NRPAs with:

· placebo or no medicine;

· nicotine replacement therapy, such as patches or gum;

· bupropion (another medicine to help people stop smoking);

· another NRPA;

· e‐cigarettes.

The USA hosted the most studies (28 studies). Other studies took place in a range of countries across the world, some in several countries.

**Main results**

People are more likely to stop smoking for at least six months using varenicline than using placebo (41 studies, 17,395 people), bupropion (9 studies, 7560 people), or just one type of nicotine replacement therapy, like patches alone (11 studies, 7572 people). They may be just as likely to quit as people using two or more kinds of nicotine replacement therapy, like patches and gum together (5 studies, 2344 people).

Cytisine probably helps more people to stop smoking than placebo (4 studies, 4623 people) and based on studies that compared cytisine with varenicline (2 studies, 2131 people), there may be a benefit from varenicline for quitting smoking, however further evidence could strengthen this finding or show a benefit from cytisine.

For every 100 people using varenicline to stop smoking, 21 to 25 might successfully stop, compared with only 18 of 100 people using bupropion, 18 of 100 people using a single form of nicotine‐replacement therapy, and 20 of 100 using two or more kinds of nicotine‐replacement therapy. For every 100 people using cytisine to stop smoking, 18 to 23 might successfully stop.

The most common unwanted effect of varenicline is nausea, but this is mostly at mild or moderate levels and usually clears over time. People taking varenicline likely have an increased chance of a more serious unwanted effect that could result in going to hospital, however these are still rare (2.7% to 4% of people on varenicline, compared with 2.7% of people without) and may include many that are unrelated to varenicline. People taking cytisine may also have a slightly increased chance of serious unwanted effects compared with people not taking it, but this may be less likely compared with varenicline.

**What are the limitations of the evidence?**

The evidence for some of our results is very reliable. We’re very confident that varenicline helps people to quit smoking better than many alternatives. We’re less sure of some other results because fewer or smaller studies provided evidence.

Several results suggest one treatment is better or less harmful than another, but the opposite could still be true.

**How up to date is the evidence?**

The evidence is up to date to 29 April 2022.

## Summary of findings

**Summary of findings 1:** Varenicline versus placebo or no medication for smoking cessation

**Varenicline versus placebo or no medication for smoking cessation**						
**Patient or population:** people who smoke tobacco **Setting:** smoking cessation clinics, hospitals, universities and other research centres **Intervention:** varenicline **Comparison:** placebo or no medication						
**Outcomes**	Anticipated absolute effects^*^ (95% CI)		**Relative effect (95% CI)**	**№ of participants (studies)**	**Certainty of the evidence (GRADE)**	**Comments**
	**Risk with placebo or no medication**	**Corresponding risk with varenicline**				
**Smoking abstinence at longest follow‐up** (6+ months) (varenicline vs placebo)	99 per 1000	**230 per 1000** (213 to 249)	**RR 2.32** (2.15 to 2.51)	17,395 (41 studies)	⊕⊕⊕⊕^a^ High	
**SAEs** (varenicline vs placebo or no medication)	27 per 1000	**33 per 1000** (27 to 40)	**RR 1.23** (1.01 to 1.48)	14,356 (26 studies)	⊕⊕⊕⊝^b^ Moderate	
**Neuropsychiatric SAEs** (varenicline vs placebo or no medication)	11 per 1000	**10 per 1000** (7 to 14)	**RR 0.89** (0.61 to 1.29)	7846 (22 studies)	⊕⊕⊝⊝^c^ Low	
**Cardiac SAEs** (varenicline vs placebo or no medication)	11 per 1000	**13 per 1000** (8 to 20)	**RR 1.20** (0.79 to 1.84)	7151 (18 studies)	⊕⊕⊝⊝^c^ Low	
*The risk in the intervention group (and its 95% confidence interval) is based on the assumed risk in the comparison group and the **relative effect** of the intervention (and its 95% CI). The assumed risk in the comparison group is calculated as the median risk in control groups. **CI:** confidence interval; **RR:** risk ratio; **SAE**: serious adverse event						
**GRADE Working Group grades of evidence** **High certainty:** we are very confident that the true effect lies close to that of the estimate of the effect. **Moderate certainty:** we are moderately confident in the effect estimate: the true effect is likely to be close to the estimate of the effect, but there is a possibility that it is substantially different. **Low certainty:** our confidence in the effect estimate is limited: the true effect may be substantially different from the estimate of the effect. **Very low certainty:** we have very little confidence in the effect estimate: the true effect is likely to be substantially different from the estimate of effect.						

^a^Moderate heterogeneity detected, however all but three studies showed positive effect of varenicline, so we did not downgrade on this basis. ^b^Downgraded one level because of imprecision: CI incorporates no clinical difference as well as clinically significant harm. ^c^Downgraded two levels because of imprecision: CI incorporates clinically significant benefit and clinically significant harm.

**Summary of findings 2:** Cytisine versus placebo or no medication for smoking cessation

**Cytisine versus placebo or no medication for smoking cessation**						
**Patient or population:** people who smoke tobacco **Setting:** smoking cessation clinics, hospitals, universities and other research centres **Intervention:** cytisine **Comparison:** placebo or no medication						
**Outcomes**	Anticipated absolute effects^*^ (95% CI)		**Relative effect (95% CI)**	**№ of participants (studies)**	**Certainty of the evidence (GRADE)**	**Comments**
	**Risk with placebo or no medication**	**Corresponding risk with cytisine**				
**Smoking abstinence at longest follow‐up** (6+ months) (cytisine vs placebo)	158 per 1000	**205 per 1000** (181 to 232)	**RR 1.30** (1.15 to 1.47)	4623 (4 studies)	⊕⊕⊕⊝^a^ Moderate	
**SAEs** (cytisine vs placebo or no medication)	46 per 1000	**48 per 1000** (36 to 63)	**RR 1.04** (0.78 to 1.37)	3781 (3 studies)	⊕⊕⊝⊝^b^ Low	
**Neuropsychiatric SAEs** (cytisine vs placebo or no medication)	No data	No data	No data	No data	No data	
**Cardiac SAEs** (cytisine vs placebo or no medication)	No data	No data	No data	No data	No data	
*The risk in the intervention group (and its 95% confidence interval) is based on the assumed risk in the comparison group and the **relative effect** of the intervention (and its 95% CI). The assumed risk in the comparison group is calculated as the median risk in control groups. **CI:** confidence interval; **RR:** risk ratio; **SAE**: serious adverse event						
**GRADE Working Group grades of evidence** **High certainty:** we are very confident that the true effect lies close to that of the estimate of the effect. **Moderate certainty:** we are moderately confident in the effect estimate; the true effect is likely to be close to the estimate of the effect, but there is a possibility that it is substantially different. **Low certainty:** our confidence in the effect estimate is limited; the true effect may be substantially different from the estimate of the effect. **Very low certainty:** we have very little confidence in the effect estimate; the true effect is likely to be substantially different from the estimate of effect.						

^a^Downgraded one level because of heterogeneity: I^2^ = 83%. ^b^Downgraded two levels because of imprecision: CI incorporates clinically significant benefit and clinically significant harm.

**Summary of findings 3:** Cytisine versus varenicline for smoking cessation

**Cytisine versus varenicline for smoking cessation**						
**Patient or population:** people who smoke tobacco **Setting:** community, community pharmacy, participants' homes **Intervention:** cytisine **Comparison:** varenicline						
**Outcomes**	Anticipated absolute effects^*^ (95% CI)		**Relative effect (95% CI)**	**№ of participants (studies)**	**Certainty of the evidence (GRADE)**	**Comments**
	**Risk with varenicline**	**Corresponding risk with cytisine**				
**Smoking abstinence at longest follow‐up** (6+ months)	132 per 1000	**109 per 1000** (87 to 138)	**RR 0.83** (0.66 to 1.05)	2131 (2 studies)	⊕⊕⊕⊝^a^ Moderate	
**SAEs**	49 per 1000	**33 per 1000** (21 to 50)	**RR 0.67** (0.44 to 1.03)	2017 (2 studies)	⊕⊕⊝⊝^b^ Low	
**Neuropsychiatric SAEs**	No data	No data	No data	No data	No data	
**Cardiac SAEs**	No data	No data	No data	No data	No data	
*The risk in the intervention group (and its 95% confidence interval) is based on the assumed risk in the comparison group and the **relative effect** of the intervention (and its 95% CI). The assumed risk in the comparison group is calculated as the median risk in control groups. **CI:** confidence interval; **RR:** risk ratio; **SAE**: serious adverse event						
**GRADE Working Group grades of evidence** **High certainty:** we are very confident that the true effect lies close to that of the estimate of the effect. **Moderate certainty:** we are moderately confident in the effect estimate; the true effect is likely to be close to the estimate of the effect, but there is a possibility that it is substantially different. **Low certainty:** our confidence in the effect estimate is limited; the true effect may be substantially different from the estimate of the effect. **Very low certainty:** we have very little confidence in the effect estimate; the true effect is likely to be substantially different from the estimate of effect.						

^a^Downgraded one level because of imprecision: CI incorporates no difference as well as clinically significant harm. ^b^Downgraded two level because of imprecision: CI incorporates no difference as well as clinically significant benefit, and number of events in analysis very low (n = 82).

**Summary of findings 4:** Cytisine versus nicotine replacement therapy for smoking cessation

**Cytisine versus nicotine replacement therapy for smoking cessation**						
**Patient or population:** people who smoke tobacco **Setting:** participants' homes (participants were callers to a national Quitline) **Intervention:** cytisine **Comparison:** nicotine replacement therapy (NRT)						
**Outcomes**	Anticipated absolute effects^*^ (95% CI)		**Relative effect (95% CI)**	**№ of participants (studies)**	**Certainty of the evidence (GRADE)**	**Comments**
	**Risk with NRT**	**Corresponding risk with cytisine**				
**Smoking abstinence at longest follow‐up** (6+ months)	153 per 1000	**218 per 1000** (173 to 275)	**RR 1.43** (1.13 to 1.80)	1310 (1 study)	⊕⊕⊝⊝^a,b^ Low	
**SAEs**	60 per 1000	**68 per 1000** (45 to 104)	**RR 1.15** (0.76 to 1.75)	1310 (1 study)	⊕⊝⊝⊝^a,c^ Very low	
**Neuropsychiatric SAEs**	No data	No data	No data	No data	No data	
**Cardiac SAEs**	No data	No data	No data	No data	No data	
*The risk in the intervention group (and its 95% confidence interval) is based on the assumed risk in the comparison group and the **relative effect** of the intervention (and its 95% CI). The assumed risk in the comparison group is calculated as the median risk in control groups. **CI:** confidence interval; **NRT:** nicotine replacement therapy; **RR:** risk ratio; **SAE**: serious adverse event						
**GRADE Working Group grades of evidence** **High certainty:** we are very confident that the true effect lies close to that of the estimate of the effect. **Moderate certainty:** we are moderately confident in the effect estimate; the true effect is likely to be close to the estimate of the effect, but there is a possibility that it is substantially different. **Low certainty:** our confidence in the effect estimate is limited; the true effect may be substantially different from the estimate of the effect. **Very low certainty:** we have very little confidence in the effect estimate; the true effect is likely to be substantially different from the estimate of effect.						

^a^Downgraded one level because of risk of bias: sole study at high risk. ^b^Downgraded one level because of imprecision: fewer than 300 events in the analysis. ^c^Downgraded two levels because of imprecision: CI incorporates clinically significant benefit and clinically significant harm.

**Summary of findings 5:** Varenicline versus bupropion for smoking cessation

**Varenicline versus bupropion for smoking cessation**						
**Patient or population:** people who smoke tobacco **Setting:** smoking cessation clinics, hospitals, universities and other research centres **Intervention:** varenicline **Comparison:** bupropion						
**Outcomes**	Anticipated absolute effects^*^ (95% CI)		**Relative effect (95% CI)**	**№ of participants (studies)**	**Certainty of the evidence (GRADE)**	**Comments**
	**Risk with bupropion**	**Corresponding risk with varenicline**				
**Smoking abstinence at longest follow‐up** (6+ months)	177 per 1000	**241 per 1000** (222 to 264)	**RR 1.36** (1.25 to 1.49)	7560 (9 studies)	⊕⊕⊕⊕ High	
**SAEs**	20 per 1000	**18 per 1000** (12 to 27)	**RR 0.89** (0.61 to 1.31)	5317 (5 studies)	⊕⊕⊝⊝^a^ Low	
**Neuropsychiatric SAEs**	2 per 1000	**2 per 1000** (0 to 16)	**RR 1.05** (0.16 to 7.04)	866 (2 studies)	⊕⊕⊝⊝^a^ Low	
**Cardiac SAEs**	0 per 1000	**0 per 1000** (0 to 0)	**RR 3.17** (0.33 to 30.18)	866 (2 studies)	⊕⊕⊝⊝^a^ Low	
*The risk in the intervention group (and its 95% confidence interval) is based on the assumed risk in the comparison group and the **relative effect** of the intervention (and its 95% CI). The assumed risk in the comparison group is calculated as the median risk in control groups. **CI:** confidence interval; **RR:** risk ratio; **SAE**: serious adverse event						
**GRADE Working Group grades of evidence** **High certainty:** we are very confident that the true effect lies close to that of the estimate of the effect. **Moderate certainty:** we are moderately confident in the effect estimate; the true effect is likely to be close to the estimate of the effect, but there is a possibility that it is substantially different. **Low certainty:** our confidence in the effect estimate is limited; the true effect may be substantially different from the estimate of the effect. **Very low certainty:** we have very little confidence in the effect estimate; the true effect is likely to be substantially different from the estimate of effect.						

^a^Downgraded two levels because of imprecision: CI incorporates clinically significant benefit and clinically significant harm.

**Summary of findings 6:** Varenicline versus nicotine replacement therapy monotherapy for smoking cessation

**Varenicline versus nicotine replacement therapy (NRT) monotherapy for smoking cessation**						
**Patient or population:** people who smoke tobacco **Setting:** smoking cessation clinics, hospitals, universities and other research centres **Intervention:** varenicline **Comparison:** n**i**cotine replacement therapy (NRT) monotherapy						
**Outcomes**	Anticipated absolute effects^*^ (95% CI)		**Relative effect (95% CI)**	**№ of participants (studies)**	**Certainty of the evidence (GRADE)**	**Comments**
	**Risk with NRT monotherapy**	**Corresponding risk with varenicline**				
**Smoking abstinence at longest follow‐up** (6+ months)	180 per 1000	**225 per 1000** (205 to 247)	**RR 1.25** (1.14 to 1.37)	7572 (11 studies)	⊕⊕⊕⊕ High	
**SAEs**	9 per 1000	**6 per 1000** (5 to 9)	**RR 0.70** (0.50 to 0.99)	6535 (6 studies)	⊕⊕⊝⊝^a^ Low	No events in two studies
**Neuropsychiatric SAEs**	Not estimable (no events in analysis)	Not estimable (no events in analysis)	Not estimable (no events in analysis)	137 (1 study)		
**Cardiac SAEs**	Not estimable (no events in analysis)	Not estimable (no events in analysis)	Not estimable (no events in analysis)	137 (1 study)		
*The risk in the intervention group (and its 95% confidence interval) is based on the assumed risk in the comparison group and the **relative effect** of the intervention (and its 95% CI). The assumed risk in the comparison group is calculated as the median risk in control groups. **CI:** confidence interval; **NRT:** nicotine replacement therapy; **RR:** risk ratio; **SAE**: serious adverse event						
**GRADE Working Group grades of evidence** **High certainty:** we are very confident that the true effect lies close to that of the estimate of the effect. **Moderate certainty:** we are moderately confident in the effect estimate; the true effect is likely to be close to the estimate of the effect, but there is a possibility that it is substantially different. **Low certainty:** our confidence in the effect estimate is limited; the true effect may be substantially different from the estimate of the effect. **Very low certainty:** we have very little confidence in the effect estimate; the true effect is likely to be substantially different from the estimate of effect.						

^a^Downgraded two levels because of imprecision: fewer than 150 events in the analysis.

**Summary of findings 7:** Varenicline versus combination nicotine replacement therapy for smoking cessation

** Varenicline versus combination nicotine replacement therapy for smoking cessation**						
**Patient or population:** people who smoke tobacco **Setting:** smoking cessation clinics, hospitals, universities and other research centres **Intervention:** varenicline **Comparison:** combination nicotine replacement therapy (NRT)						
**Outcomes**	Anticipated absolute effects^*^ (95% CI)		**Relative effect (95% CI)**	**№ of participants (studies)**	**Certainty of the evidence (GRADE)**	**Comments**
	**Risk with combination NRT**	**Corresponding risk with varenicline**				
**Smoking abstinence at longest follow‐up** (6+ months)	195 per 1000	**199 per 1000** (170 to 234)	**RR 1.02** (0.87 to 1.20)	2344 (5 studies)	⊕⊕⊝⊝^a^ Low	
**SAEs**	2 per 1000	**5 per 1000** (1 to 20)	**RR 2.15** (0.49 to 9.46)	1852 (4 studies)	⊕⊕⊝⊝^b^ Low	
**Neuropsychiatric SAEs**	0 per 1000	**0 per 1000** (0 to 0)	**RR 4.69** (0.23 to 96.50)	764 (2 studies)	⊕⊕⊝⊝^b^ Low	Only one study reported any events
**Cardiac SAEs**	2 per 1000	**1 per 1000** (0 to 19)	**RR 0.32** (0.01 to 7.88)	819 (2 studies)	⊕⊕⊝⊝^b^ Low	Only one study reported any events
*The risk in the intervention group (and its 95% confidence interval) is based on the assumed risk in the comparison group and the **relative effect** of the intervention (and its 95% CI). The assumed risk in the comparison group is calculated as the median risk in control groups. **CI:** confidence interval; **NRT:** nicotine replacement therapy; **RR:** risk ratio; **SAE**: serious adverse event						
**GRADE Working Group grades of evidence** **High certainty:** we are very confident that the true effect lies close to that of the estimate of the effect. **Moderate certainty:** we are moderately confident in the effect estimate; the true effect is likely to be close to the estimate of the effect, but there is a possibility that it is substantially different. **Low certainty:** our confidence in the effect estimate is limited; the true effect may be substantially different from the estimate of the effect. **Very low certainty:** we have very little confidence in the effect estimate; the true effect is likely to be substantially different from the estimate of effect.						

^a^Downgraded two levels because of imprecision: confidence intervals include the potential for clinically significant benefit from either intervention. ^b^Downgraded two levels because of imprecision: CI incorporates clinically significant benefit and clinically significant harm.

**Summary of findings 8:** Varenicline versus e‐cigarettes for smoking cessation

**Varenicline versus e‐cigarettes for smoking cessation**						
**Patient or population:** people who continued to smoke tobacco following acute coronary syndrome **Setting:** hospital **Intervention:** varenicline **Comparison:** e‐cigarettes						
**Outcomes**	Anticipated absolute effects^*^ (95% CI)		**Relative effect (95% CI)**	**№ of participants (studies)**	**Certainty of the evidence (GRADE)**	**Comments**
	**Risk with e‐cigarettes**	**Corresponding risk with varenicline**				
**Smoking abstinence at longest follow‐up** (6+ months)	148 per 1000	481 per 1000 (179 to 1000)	RR 3.25 (1.21 to 8.71)	54 (1 study)	⊕⊝⊝⊝^a,b^ Very low	
**SAEs**	Not estimable (no events in analysis)	Not estimable (no events in analysis)	Not estimable (no events in analysis)	54 (1 study)		
**Neuropsychiatric SAEs**	Not estimable (no events in analysis)	Not estimable (no events in analysis)	Not estimable (no events in analysis)	54 (1 study)		
**Cardiac SAEs**	Not estimable (no events in analysis)	Not estimable (no events in analysis)	Not estimable (no events in analysis)	54 (1 study)		
*The risk in the intervention group (and its 95% confidence interval) is based on the assumed risk in the comparison group and the **relative effect** of the intervention (and its 95% CI). The assumed risk in the comparison group is calculated as the median risk in control groups. **CI:** confidence interval; **RR:** risk ratio; **SAE**: serious adverse event						
**GRADE Working Group grades of evidence** **High certainty:** we are very confident that the true effect lies close to that of the estimate of the effect. **Moderate certainty:** we are moderately confident in the effect estimate; the true effect is likely to be close to the estimate of the effect, but there is a possibility that it is substantially different. **Low certainty:** our confidence in the effect estimate is limited; the true effect may be substantially different from the estimate of the effect. **Very low certainty:** we have very little confidence in the effect estimate; the true effect is likely to be substantially different from the estimate of effect.						

^a^Downgraded two levels because of imprecision: fewer than 150 events in the analysis. ^b^Downgraded one level because of risk of bias: sole study at high risk.

## Background

### Description of the condition

Smoking is the main preventable cause of morbidity and premature death worldwide, killing more than 8 million people each year (WHO 2022). It is also a leading cause of health inequalities (ASH 2019). Quitting tobacco smoking significantly reduces risk of tobacco‐related disease and death (USDHHS 2020). There are a range of interventions available to help people quit smoking, including different kinds of behavioural and pharmacological support, but even with the most effective interventions, long‐term quit rates remain relatively low (Livingstone‐Banks 2022a; Rigotti 2022).

### Description of the intervention and how it might work

Nicotine receptor partial agonists are a family of drugs that aim to mitigate the addictiveness of tobacco by binding to the α4β2 nicotinic acetylcholine receptor (the receptor that mediates nicotine dependence through released dopamine). When bound to the receptor, a partial agonist prompts the receptor to release dopamine in the way nicotine would, and prevents nicotine from tobacco from binding to the receptor. This reduces nicotine withdrawal symptoms and reduces the rewarding effects of tobacco. There are two main nicotine receptor partial agonists: varenicline and cytisine. A third drug, dianicline, was developed but unfavourable results led to its withdrawal from further development (Kirchhoff 2009).

Varenicline was developed by Pfizer Inc in 1997 (Coe 2005), and was approved as a prescription‐only aid to smoking cessation in 2006 by the American Food and Drug Administration under the trade name Chantix, and by the European Medicines Evaluation Agency under the trade name Champix. In July 2007 it was approved by the National Institute for Health and Clinical Excellence (NICE) for prescribing by the UK National Health Service (ASH 2006; NICE 2007). In 2021, the World Health Organization added varenicline to its Essential Medicines List (WHO 2021). Post‐marketing surveillance raised subsequent concerns about possible links between varenicline and major health risks, including suicidal ideation and behaviour, depression, and serious adverse cardiovascular events (FDA 2008), which led to an FDA warning label in 2009. This warning was removed in 2016 after a large trial found no evidence to support the concerns (EAGLES 2016). In 2021, Pfizer announced a recall of varenicline because it exceeded acceptable intake limits of a nitrosamine impurity, called N‐nitroso‐varenicline. While this is believed to only be temporary, it has led to shortages at the time of writing.

Cytisine was developed in Bulgaria in the 1960s, and is less widely available than varenicline (Foulds 2004; Tutka 2005; Tutka 2006). Its original manufacturer, Sopharma Pharmaceuticals, developed their phytoproduct from the plant *Cytisus Laburnum* L. (Golden Rain). Although cytisine is not licensed and available for use as a smoking cessation aid across most countries outside Eastern Europe, it works by the same mechanism as varenicline and it is available for substantially less cost (Tutka 2019; Gotti 2021). An important difference between the treatments is that standard treatment with cytisine lasts 25 days, compared with 12 weeks for varenicline.

### Why it is important to do this review

The scale of the impact on health from tobacco worldwide makes it imperative that we continue to develop our understanding of smoking cessation interventions. While the effectiveness of varenicline for smoking cessation is well established, substantial questions remain about different doses and durations of treatment, and what impact they have on how effective varenicline is at helping people to quit smoking.

Varenicline is a front‐line smoking cessation medication in many countries, and its current shortage poses a substantial challenge for tobacco control strategies around the world. Learning more about how effective and safe cytisine is for smoking cessation may inform decisions about whether to licence it in countries that have historically relied on varenicline.

This is an update of a Cochrane Review first published in 2007, and most recently updated in 2016. The previous update found high‐certainty evidence of a benefit from varenicline, but only included a limited number of studies testing cytisine for smoking cessation (Cahill 2016). New evidence comparing cytisine with placebo and with varenicline warranted an update of this review.

## Objectives

To assess the effectiveness of nicotine receptor partial agonists, including varenicline and cytisine, for smoking cessation.

## Methods

### Criteria for considering studies for this review

#### Types of studies

We included randomised controlled trials (RCTs) and cluster‐RCTs. We did not include quasi‐randomised studies, in which the allocation sequence is not truly random, for example, studies where participant date of birth determines participant allocation.

#### Types of participants

We included studies that recruited adult tobacco smokers. Studies testing nicotine receptor partial agonists to help smokeless tobacco users to quit, or as a relapse prevention intervention among people who are already abstinent from smoking tobacco, are covered in separate Cochrane Reviews (Ebbert 2011a; Livingstone‐Banks 2019; Livingstone‐Banks 2022b).

#### Types of interventions

Selective nicotine receptor partial agonists, including cytisine, dianicline and varenicline (or any other in this class of drug as they reach Phase 3 trial stage), compared with placebo, no medication, or another smoking cessation pharmacotherapy (including nicotine replacement therapy, bupropion, electronic cigarettes, and other nicotine receptor partial agonists). We also included studies that compared different doses and regimes of eligible treatments. Lobeline is covered in an earlier Cochrane Review (Stead 2003). We only included studies that tested the effect of nicotine receptor partial agonists for smoking cessation and not studies focused on harm reduction, which is covered in a separate Cochrane Review (Lindson‐Hawley 2016).

#### Types of outcome measures

##### Primary outcomes

- Abstinence from smoked tobacco at longest follow‐up, at least six months from study baseline. We used the strictest definition of abstinence reported in each study (e.g. prolonged or continuous over point prevalence), and where available, we favoured biochemically validated over self‐reported abstinence. We only included studies that measured abstinence from tobacco smoking at six months or longer from baseline.
- Number of participants who experienced the following adverse events: nausea, insomnia, abnormal dreams, headache, depression, and suicidal ideation
- Number of participants who experienced serious adverse events as defined by the authors of included studies
- Number of participants who experienced neuropsychiatric serious adverse events
- Number of participants who experienced cardiac serious adverse events.

### Search methods for identification of studies

#### Electronic searches

We searched the Cochrane Tobacco Addiction Group's Specialised Register for studies, using relevant terms (e.g. 'cytisine' or 'Tabex' or 'dianicline' or 'varenicline' or 'nicotine receptor partial agonist') in the title or abstract, or as keywords. This Register has been developed from electronic searching of the Cochrane Central Register of Controlled trials (CENTRAL), MEDLINE, Embase, and PsycINFO, together with handsearching of specialist journals, conference proceedings and reference lists of previous trials and overviews. The most recent search of the Register was on 29 April 2022, and included reports of trials indexed in CENTRAL, 2022, Issue 3; MEDLINE (via OVID) to update 20220405; Embase (via OVID) to week 202214; PsycINFO (via OVID) to update 20220404, all from inception. See the Cochrane Tobacco Addiction Group Website for details of the search strategies for these databases. The search strategy for this specific review is listed in Appendix 1. We did not place any limits on our searches (e.g. by language, year of publication, or publication format).

#### Searching other resources

Our search of the Cochrane Tobacco Addiction Group Specialised Register also covered ongoing and unpublished trials included in the following databases, as these are indexed in CENTRAL.

- US National Institutes of Health Ongoing Trials Register ClinicalTrials.gov (www.clinicaltrials.gov, searched via CENTRAL); and
- World Health Organization (WHO) International Clinical Trials Registry Platform (apps.who.int/trialsearch, searched via CENTRAL).

We also checked the reference lists of included studies for potentially eligible trials.

### Data collection and analysis

#### Selection of studies

For this update we screened the search results in two stages using the software Covidence. Two review authors (of JL‐B, AT, AH and NL) independently screened the title and abstract of each study found in our searches. We then reviewed the full text of all potentially eligible reports in duplicate. At each stage, we resolved any disagreement through discussion and if needed by referring to a third review author. We noted the reasons for study exclusion at full‐text stage for our PRISMA diagram illustrating the flow of studies (Liberati 2009).

#### Data extraction and management

Two review authors (of JL‐B, AT, AH, LH, TRF, and KT) independently extracted the following information about each included study in duplicate, using a prepiloted data extraction form. We resolved disagreement through discussion and if needed by referring to a third review author.

- Country and setting (e.g. primary care, community, hospital outpatient/inpatient)
- Method of recruiting participants
- Definition of smoker used
- Methods of randomisation and allocation, and blinding of study personnel, participants and assessors
- Demographic characteristics of participants (e.g. average age, sex, average cigarettes per day)
- Intervention and control description (dose, provider, duration, number of visits, etc.)
- Outcomes including definition of abstinence used, and biochemical validation of cessation
- Proportion of participants with follow‐up data
- Any adverse events
- Declarations of interest and sources of study funding

#### Assessment of risk of bias in included studies

We assessed each included study using Cochrane's RoB 1 tool for the following domains of risk (Higgins 2011).

- Random sequence generation (selection bias)
- Allocation concealment (selection bias)
- Blinding of participants and study personnel and blinding/objectivity of outcome assessment (performance bias and detection bias)
- Incomplete outcome data (attrition bias)
- Selective reporting (reporting bias)
- Other potential risks of bias. For this domain, we assessed and reported any forms of bias present in studies that did not fall under one of the above domains. Where no relevant form of other potential bias was found, we left this field blank.

Two review authors (of JL‐B, AT, AH, LH, TRF, and KT) independently judged each study as at low, unclear, or high risk of bias for each domain, justifying judgements using information from the study report. We resolved disagreements in judgements through discussion and by referral to a third review author where needed.

#### Measures of treatment effect

We present estimates of effects for individual studies using risk ratios (RRs), calculated as ((number of events in intervention condition/intervention denominator)/(number of events in control condition/control denominator)), with a 95% confidence interval (CI). An RR greater than one indicates a higher rate of outcome (either smoking abstinence or adverse events) in the intervention group than in the control group.

#### Unit of analysis issues

As cluster‐randomised trials are eligible for inclusion in this review, there is the potential for unit of analysis issues. Where required, we adjusted for clustering using an intraclass correlation, either from the study in question or from a similar study. Where studies compared more than one eligible intervention arm with a non‐intervention control, we either pooled intervention arms together (assuming they did not differ in pharmacotherapy given) or added them separately to the meta‐analysis and split the control group data evenly between them, to avoid double‐counting any participants in the analysis.

#### Dealing with missing data

We conducted our analyses on an intention‐to‐treat basis, including all participants in the study arms to which they were randomised, regardless of whether they received the intervention. We counted participants lost to follow‐up as continuing smoking, which is standard in the field (West 2005). Where study reports lacked the information needed for our analyses, we tried to contact study authors to ask for this information. Attempts to contact study authors are recorded in the Characteristics of included studies tables.

#### Assessment of heterogeneity

To investigate heterogeneity, we used the I^2^ statistic, given by the formula [(Q ‐ df)/Q] × 100%, where Q is the Chi^2^ statistic and df is its degrees of freedom (Higgins 2003). This describes the percentage of variability in effect estimates that is due to heterogeneity rather than to sampling error (chance). We interpreted the I^2^ result using the following overlapping bands (Deeks 2022):

- 0% to 40%: might not be important
- 30% to 60%: may represent moderate heterogeneity
- 50% to 90%: may represent substantial heterogeneity
- 75% to 100%: considerable heterogeneity

Where we found moderate to substantial heterogeneity, we investigated further using subgroup analyses based on study characteristics decided upon through review author consensus. In the event of considerable unexplained statistical heterogeneity (i.e. I^2^ =75% or over), we evaluated whether it was still appropriate to report a pooled result (Deeks 2022).

#### Assessment of reporting biases

For the two smoking cessation comparisons with 10 or more studies, we assessed the risk of reporting bias using a funnel plot. Regardless of the number of studies included, we considered the possibility of reporting bias in our discussion.

#### Data synthesis

We conducted a narrative summary of the included studies and, where more than one study reported an outcome comparing an eligible intervention with placebo, no medication, another eligible intervention, or the same intervention delivered with a different dose or regime, we conducted meta‐analyses to pool data from sufficiently similar studies using a Mantel‐Haenszel fixed‐effect model to calculate pooled RRs with 95% CIs.

#### Subgroup analysis and investigation of heterogeneity

Where studies compared an intervention pharmacotherapy with either placebo or no medication and there was substantial heterogeneity, we considered subgrouping analyses based on comparator and using the I^2^ statistic to test for difference between subgroups and decide whether to report an overall pooling or by subgroup only.

#### Sensitivity analysis

We conducted sensitivity analyses testing the effect of removing studies we judged to be at high risk of bias to see if those studies affected the overall result. In analyses where we pooled studies that compared an intervention with either placebo or no medication but did not subgroup, we conducted sensitivity analyses testing removing studies comparing against no medication. In our comparison of varenicline versus placebo we also conducted a further ad hoc sensitivity analysis to explore the high level of heterogeneity, removing studies that used an extended treatment course of 24 or 52 weeks rather than the 12 weeks of the other studies.

Because we were primarily interested in whether there is evidence that varenicline works differently for disease‐specific populations and people in specific subgroups and healthcare settings, we conducted sensitivity analyses, treating studies in these populations and settings as subgroups of the main analyses and using the I^2^ statistic to test for subgroup differences.

#### Summary of findings and assessment of the certainty of the evidence

Following standard Cochrane methods, we produced summary of findings tables for smoking abstinence at longest follow‐up and all of our serious adverse events outcomes for each comparison of varenicline or cytisine with placebo or another pharmacotherapy (Schünemann 2022). Two review authors (JLB, NL) assessed the certainty of the evidence using the five GRADE considerations (risk of bias, inconsistency, imprecision, indirectness, and publication bias (Schünemann 2013).

## Results

### Description of studies

#### Results of the search

Our literature searches for this update found 810 studies (from 885 records). After we removed duplicates, 682 studies remained for title and abstract screening. We ruled out 544 studies at this stage, leaving 138 studies for full‐text screening. From this, we identified 45 new included studies and 20 new ongoing studies, combined with studies from previous updates of this review, this resulted in a total of 75 included studies of 45,049 people and 28 ongoing studies. See Figure 1 for PRISMA diagram detailing study flow (Liberati 2009). For this update, we contacted authors of four studies and received additional results data.

**1:**
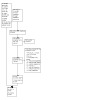
Study flow diagram of searches for 2022 update

For this update, we excluded 14 previously included studies because they focused on relapse prevention (these studies are covered in a separate review; Livingstone‐Banks 2019), or had previously been included for data on harms but did not fully meet our inclusion criteria.

#### Included studies

Full details of the included studies are given in the Characteristics of included studies tables.

##### Cytisine

Eight studies in just under 9000 people investigated cytisine as a smoking cessation drug. Four studies compared cytisine with placebo, two with varenicline, one with nicotine replacement therapy (NRT), and one with no medication. Pastorino 2022 also compared longer with shorter duration cytisine. Studies tested cytisine at a dose of 9 mg per day for 20 to 25 days, except for Pastorino 2022, which gave cytisine for 40 and 84 days in different arms.

Studies were conducted in Australia, Bangladesh and Pakistan, Italy, East Germany, Kyrgyzstan, New Zealand, and Poland. Two studies took place in smoking cessation clinics, and two in the community. Walker 2014 recruited people who contacted a national smoking quitline. Vinnikov 2008 was set in a Kyrgyz mining company, and Dogar 2020 took place in tuberculosis treatment centres. Pastorino 2022 recruited heavy smokers participating in a lung‐screening trial.

##### Varenicline

Sixty‐eight studies of over 37,000 people tested varenicline for smoking cessation. This excludes two studies that compared varenicline with cytisine, which are described above.

###### Setting

Twenty‐eight studies were conducted in the USA, four in Canada, three in China, three in Japan, two in France, two in Greece, two in India, two in Turkey, one in Australia, one in Iran, one in Denmark, one in Finland, one in Israel, and one in Spain. Fifteen studies took place internationally, in between two and 15 countries. The studies were conducted in smoking cessation clinics, hospitals, universities and other research centres.

###### Participants

Participants in the majority of trials were adult smokers, willing to make a quit attempt. Several trials were conducted in clinical subgroups, including hospital inpatients (Carson‐Chahhoud 2020; Hong 2015; Le Mao 2020; Steinberg 2011; Windle 2018; Wong 2012), and disease‐specific patient groups: cardiovascular disease (Rigotti 2010; Windle 2018); chronic obstructive pulmonary disease (Hong 2015; Le Mao 2020; Tashkin 2011; Yang 2016); HIV (Ashare 2019; Mercie 2018); asthma (Westergaard 2015); substance use disorder (Nahvi 2014a; Stein 2013); alcohol dependence (Hurt 2018; O'Malley 2018; Zawertailo 2020); depression (Anthenelli 2013; Cinciripini 2018); and bipolar/schizophrenia, schizoaffective disorder (Chengappa 2014; Williams 2012). EAGLES 2016 enrolled two cohorts of adult smokers with and without histories of psychiatric disorders, including primary affective disorders (70%), anxiety disorders (19%), psychotic disorders (9.5%) and personality disorders (0.6%). Gonzales 2014 recruited people who had previously used varenicline in an unsuccessful quit attempt. De Dios 2012 and Ebbert 2016 tested varenicline in light smokers.

###### Interventions

Forty‐seven trials used the standard 12‐week regimen of varenicline, routinely titrating the first week up to the recommended daily dose of 1 mg twice a day. Nakamura 2007, Nides 2006 and Oncken 2006 tested 1 mg per day, and Niaura 2008 allowed participants to regulate their own dosage throughout the treatment phase. Ebbert 2015 and Stein 2013 tested a 24‐week regimen, and Williams 2007 tested 52 weeks.

###### Comparators

Forty‐five studies compared varenicline with placebo and five with no medication. Of the 14 studies that compared varenicline with NRT, 12 randomised participants to receive single‐form NRT and five to a combination of two or more forms of NRT (3 studies tested varenicline against both NRT monotherapy and combination NRT). Ten studies compared varenicline with bupropion.

Seven studies compared standard varenicline with either a lower dose (4 studies) or a longer duration (3 studies).

###### Outcomes

All studies measured smoking cessation at least six months after study baseline. Follow‐up lengths ranged from six months to two years. Many studies biochemically validated abstinence using either exhaled carbon monoxide, or salivary or urinary cotinine.

Thirty‐eight studies measured adverse events, including nausea, insomnia, abnormal dreams, headache, depression, and suicidal ideation. Twenty‐eight measured serious adverse events, neuropsychiatric serious adverse events and cardiac serious adverse events.

##### Dianicline

One trial investigated dianicline. It was set in 22 sites across six European countries (Tonstad 2011). Dianicline was administered as a 40 mg tablet twice a day for seven weeks, with brief counselling at each contact. Final follow‐up of the participants was at 26 weeks, with self‐reported abstinence verified by expired carbon monoxide and by plasma cotinine samples.

##### Study funding

Of the trials included in this review, 35 received funds from pharmaceutical companies with interests in the treatment being tested, 13 received free study medications, and four trials without pharmaceutical support had authors who had received funds for other work. Fifteen studies reported no conflicts, and two did not report study funding or author declarations of interests. This is significant because a recent analysis found that authors of opinion pieces on varenicline who reported financial ties to the pharmaceutical industry (as a conflict of interest or funding source) were more likely to minimise the cardiovascular and psychiatric risk of varenicline compared to those without conflicts of interest or industry funding (odds ratio 4.00, 95% CI 1.32 to 12.16 for cardiovascular risk; odds ratio 8.51, 95% CI 3.79 to 19.11 for psychiatric risk; Fabbri 2022).

#### Excluded studies

We list 95 potentially eligible but ultimately excluded studies, along with reasons for exclusion, in the Characteristics of excluded studies tables. Common reasons for exclusion were following up with participants for less than six months, not randomising participants, testing an eligible intervention for an ineligible purpose (smoking reduction or alcohol dependence), or testing another intervention as an adjunct to an eligible one. 

For this update we excluded 14 studies that were included in the previous version of the review. Evins 2014, Tonstad 2006, Tønnesen 2013, and NCT00828113 recruited already abstinent participants and tested varenicline for relapse prevention, a topic covered in a different Cochrane Review (Livingstone‐Banks 2019). We excluded Hajek 2015 because follow‐up was under six months. Brandon 2011, Ebbert 2011b, Faessel 2009, Fagerström 2010, Garza 2011, Hughes 2011, McClure 2013, Meszaros 2013 and Mitchell 2012 were previously included for data on harms only but did not meet all of our prespecified inclusion criteria.

We did not find sufficient information to include or exclude two studies. These are listed in Characteristics of studies awaiting classification.

#### Ongoing studies

We found 28 eligible ongoing studies, some with multiple relevant comparisons. Studies that tested varenicline compared it with placebo (11 studies), no medication (two studies), bupropion (one study), NRT (seven studies), e‐cigarettes (one study), and different doses or regimes of varenicline (six studies). Studies that tested cytisine compared it with placebo (two studies), varenicline (two studies), NRT (one study), and e‐cigarettes (one study).

Studies were set in various populations and setting, including HIV (three studies), hospital and perioperative patients (three studies), cardiovascular disease (three studies), substance abuse (two studies), mental health (two studies), and single studies in lung cancer, diabetes, chronic obstructive pulmonary disease, adolescents, and e‐cigarette users who smoke.

Further details of the ongoing studies are given in the Characteristics of ongoing studies tables.

### Risk of bias in included studies

Overall, we judged 22 studies to be at low risk of bias (low risk of bias across all domains), 18 at high risk of bias (high risk of bias in at least one domain), and the remaining 35 at unclear risk of bias. Our judgements on the risks of bias of all the included studies are summarised in Figure 2 and Figure 3, and reasons for the judgements are detailed in the Characteristics of included studies tables.

**2:**
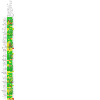
Risk of bias summary: review authors' judgements about each risk of bias item for each included study

**3:**
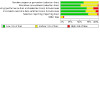
Risk of bias graph: review authors' judgements about each risk of bias item presented as percentages across all included studies

#### Allocation

We separately assessed allocation bias resulting from randomisation sequence generation and from allocation concealment. We judged 29 studies to be at unclear risk of allocation bias as a result of insufficient information about randomisation sequence generation. The remaining studies were judged at low risk, with none deemed to be at high risk.

We judged one study to be at high risk of allocation bias as a result of inadequate concealment of randomisation because that study's participant allocation was unblinded. We judged 34 studies to be at unclear risk because there was insufficient information to make a judgement. We judged the remaining studies to be at low risk.

#### Blinding

We judged 10 studies to be at high risk of performance or detection bias because they were open‐label studies without blinding of participant allocation. We judged 13 studies to be at unclear risk because there was insufficient information to make a judgement. The remaining studies were deemed at low risk.

#### Incomplete outcome data

We judged five studies to be at high risk of attrition bias, four because of either high levels of attrition or highly differential attrition rates between study arms, and one that did not provide a baseline number of participants, and reported only those followed up at 12 months as a denominator (Benli 2017). We judged 20 studies to be at unclear risk because of insufficient reporting of follow‐up rates for us to make a judgement. The remaining studies were deemed at low risk.

#### Selective reporting

We judged two studies to be at high risk of reporting bias. In Nakamura 2007, continuous abstinence rates for all participants were reported, but demographics, withdrawal and craving measures, and point‐prevalence abstinence were reported for the nicotine‐dependent subset of participants only. The trial registry entry for Tuisku 2016 planned a 12‐month follow‐up, which was not reported in their results paper. However, it is possible that this may be reported in a subsequent paper. We judged 18 studies to be at unclear risk because there was insufficient information to make a judgement; typically because no protocol or trial registry entry was available. We judged the remaining studies to be at low risk.

#### Other potential sources of bias

We judged two studies to be at high risk of bias for other reasons. For Ioakeimidis 2018, we found only an abstract and poster, which reported different quit rates in the e‐cigarette arm. Zincir 2013 reported that no participants experienced adverse events, which is unlikely given standard definitions of adverse events.

We judged five studies to be at unclear risk of bias from other sources. In Rose 2013, there was a minor unexplained reporting disparity, with different denominators given for the varenicline arm. Walker 2014 supplied cytisine for free, while NRT users had to pay a nominal charge (NZD 3 for an 8‐week course of each NRT item). Le Mao 2020 reported that their small sample size was because of premature interruption of pharmaceutical funding. Cinciripini 2013 began comparing nortriptyline with bupropion, but after three months nortriptyline was changed to varenicline. In Chengappa 2014, four participants in each arm received bupropion for depression. Three out of 15 varenicline quitters and one out of three placebo quitters were on long‐term bupropion.

### Effects of interventions

See: Table 1; Table 2; Table 3; Table 4; Table 5; Table 6; Table 7; Table 8

See summary of findings tables (Table 1; Table 2; Table 3; Table 4; Table 5; Table 6; Table 7; Table 8).

#### Cytisine versus placebo or no medication

##### Smoking cessation

We pooled five studies that compared cytisine to either placebo or no medication, subgrouping by comparator type (placebo versus no medication). We found evidence of a substantial subgroup difference (I^2^ = 97.3%; Analysis 1.1) and so present the subgroup effects separately here. Four studies, including 4623 participants, compared standard dose cytisine (9 mg per day) with placebo. More people successfully quit smoking in the cytisine arm (RR 1.30, 95% CI 1.15 to 1.47; moderate‐certainty evidence; Analysis 1.1.1), but there was a high level of heterogeneity (I^2^ = 83%). We present the pooled estimate despite this heterogeneity as all point estimates suggested a benefit from cytisine. Pastorino 2022 compared standard‐dose cytisine with no medication. This three‐arm trial tested different durations of cytisine (40 days and 84 days), so we split the control arm. More people randomised to receive cytisine successfully quit than in the no‐medication arm (RR 4.44, 95% CI 3.06 to 6.46; I^2^ = 0%; 869 participants; Analysis 1.1.2).

We were unable to conduct our planned sensitivity analysis removing studies at high risk of bias because we judged all studies comparing with placebo to be at low or unclear risk and the sole study comparing with no medication to be at high risk.

##### Adverse events

None of the studies in this comparison measured our prespecified adverse events outcomes, so we pooled all non‐serious adverse events. Results from four studies of 4052 participants showed that more people randomised to receive cytisine reported experiencing non‐serious adverse events than those randomised to receive placebo or no medication (RR 1.22, 95% CI 1.07 to 1.39; I^2^ = 0%; Analysis 1.2). However, a sensitivity analysis removing one study comparing cytisine with no medication resulted in a confidence interval that crossed the null (RR 1.19, 95% CI 0.97 to 1.46; I^2^ = 0%; 3 studies, 3183 participants).

##### Serious adverse events

Results from three studies of 3781 participants comparing cytisine with placebo or no medication showed no evidence of difference in the number who experienced serious adverse events (RR 1.04, 95% CI 0.78 to 1.37; I^2^ = 0%; low‐certainty evidence; Analysis 1.3). A sensitivity analysis removing one study comparing cytisine with no medication did not affect the interpretation of this result (RR 1.15, 95% CI 0.79 to 1.67; I^2^ = 0%; 2 studies, 3012 participants). None of the studies measured neuropsychiatric or cardiac serious adverse events.

#### Cytisine: variations in usage

Pastorino 2022 compared 40 days and 84 days of cytisine, and found that more people successfully quit on the longer treatment, although confidence intervals did cross the null, indicating the potential for no difference in the effects (RR 1.28, 95% CI 0.98 to 1.67; 480 participants; Analysis 2.1).

#### Cytisine versus varenicline

##### Smoking cessation

Two studies including 2131 people compared standard‐dose cytisine (9 mg per day) with standard‐dose varenicline (2 mg per day). The point estimate showed more people quitting in the varenicline arm, but confidence intervals indicate imprecision and incorporate the potential for no difference or slight benefit from cytisine (RR 0.83, 95% CI 0.66 to 1.05; I^2^ = 0%; moderate‐certainty evidence; point estimate favours varenicline; Analysis 3.1).

##### Adverse events

Two studies of 2017 participants found that people randomised to receive cytisine were less likely to report experiencing nausea (RR 0.41, 95% CI 0.33 to 0.50; I^2^ = 0%; Analysis 3.2) and abnormal dreams (RR 0.60, 95% CI 0.50 to 0.73; I^2^ = 58%; Analysis 3.3) than those in the varenicline arm.

The same two studies of 2017 participants, found no evidence of clear differences between the cytisine and varenicline arms in the number of people experiencing insomnia (RR 0.90, 95% CI 0.73 to 1.10; I^2^ = 68%; Analysis 3.4), headaches (RR 1.02, 95% CI 0.79 to 1.33; I^2^ = 0%; Analysis 3.5), and suicidal ideation (RR 0.33, 95% CI 0.01 to 8.02; I^2^ not estimable as events only in 1 study; Analysis 3.7). However, in all cases confidence intervals indicated imprecision, and the potential for more adverse events when using either treatment.

One study of 679 participants did not find evidence of a clear difference between cytisine and varenicline arms in the number of people experiencing depression (RR 3.04, 95% CI 0.12 to 74.47; Analysis 3.6); however, this result should also be treated with caution because of substantial imprecision.

##### Serious adverse events

Two studies of 2017 participants compared the number of people in cytisine and varenicline arms reporting experiencing serious adverse events. The point estimate showed that fewer people in the cytisine arm reported serious adverse events (RR 0.67, 95% CI 0.44 to 1.03; I^2^ = 45%; low‐certainty evidence; point estimate favours cytisine; Analysis 3.8), but confidence intervals did incorporate the potential for no difference. Neither study measured neuropsychiatric or cardiac serious adverse events.

#### Cytisine versus nicotine replacement therapy

##### Smoking cessation

Walker 2014 provided participants with cytisine, compared with an eight‐week course of NRT, supplied in the form of vouchers that required redemption by participants. Participants in the cytisine arm also received vouchers for NRT to use after their initial 25‐day course of cytisine, and study authors reported that at week one 26 participants were using NRT obtained through the vouchers; only 19 participants used NRT and cytisine concomitantly. This study found that more people in the cytisine arm successfully quit than in the NRT arm (RR 1.43, 95% CI 1.13 to 1.80; 1310 participants; low‐certainty evidence; Analysis 4.1).

##### Adverse events

Nausea was the only one of our adverse event outcomes measured. Walker 2014 reported that, compared with people randomised to receive NRT, those in the cytisine arm were more likely to report experiencing nausea (RR 15.00, 95% CI 3.60 to 62.51; 1310 participants; Analysis 4.2).

##### Serious adverse events

Walker 2014 did not find evidence of a difference in the rate of serious adverse events between those randomised to receive cytisine or NRT (RR 1.15, 95% CI 0.76 to 1.75; 1310 participants; very low‐certainty evidence; Analysis 4.3). Walker 2014 did not measure neuropsychiatric or cardiac serious adverse events.

#### Varenicline versus placebo or no medication

##### Smoking cessation

We pooled studies that compared varenicline to either placebo or no medication, subgrouping by comparator type (placebo or no medication). We found evidence of a substantial subgroup difference (I^2^ = 95.8%; Analysis 5.1) and so present the subgroup effects separately here. Forty‐six studies compared standard‐dose varenicline (2 mg per day) with either placebo or no medication. Our meta‐analysis found that more people successfully quit smoking when randomised to receive varenicline compared with placebo (RR 2.32, 95% CI 2.15 to 2.51; I^2^ = 60%, 41 studies, 17,395 participants; high‐certainty evidence; Analysis 5.1.1) or with no medication (RR 1.57, 95% CI 1.37 to 1.80; I^2^ = 95%; 5 studies, 1050 participants; Analysis 5.1.2). Despite the substantial heterogeneity in the latter subgroup we present the pooled estimate as all the individual study point estimates suggested a benefit of varenicline.

A sensitivity analysis removing studies at high risk of bias did not reduce the heterogeneity found in the varenicline versus placebo analysis or the interpretation of the effect. However, removing three studies that used an extended treatment course of 24 or 52 weeks rather than the 12 weeks of the other studies, resulted in a minor impact, reducing the I^2^ statistic to 53%. We were unable to conduct a sensitivity analysis removing high risk of bias studies for the varenicline versus no medication analysis, as we judged all studies to be at high risk.

King 2022 tested varenicline combined with NRT patch against NRT patch with a varenicline placebo. We did not include this study in our analysis, but it showed no clear evidence of a difference in quit rates as a result of adding varenicline (RR 0.94, 95% CI 0.51 to 1.72; 122 participants). However, confidence intervals incorporated the possibilities of both an increased and a decreased quit rate, as well as no difference.

##### Adverse events

Studies comparing varenicline with placebo or no medication found that people randomised to receive varenicline were more likely to report experiencing nausea (RR 2.61, 95% CI 2.44 to 2.80; I^2^ = 79%; 36 studies, 17,080 participants; Analysis 5.2), insomnia (RR 1.37, 95% CI 1.27 to 1.47; I^2^ = 29%; 35 studies, 16,803 participants; Analysis 5.3), abnormal dreams (RR 1.82, 95% CI 1.67 to 1.97; I^2^ = 70%; 32 studies, 16,211 participants; Analysis 5.4), and headaches (RR 1.11, 95% CI 1.03 to 1.19; I^2^ = 30%; 31 studies, 16,326 participants; Analysis 5.5). Statistical heterogeneity was substantial in our analyses for nausea and abnormal dreams, but we decided to present the pooled estimate because the point estimates of individuals studies were almost entirely in the same direction.

Studies comparing varenicline with placebo or no medication did not find clear evidence of difference in the numbers of participants who reported experiencing depression (RR 1.05, 95% CI 0.91 to 1.20; I^2^ = 0%; 32 studies, 15,922 participants; Analysis 5.6), and found fewer people reporting suicidal ideation in the varenicline arm (RR 0.69, 95% CI 0.44 to 1.08; I^2^ = 0%; 22 studies, 12,343 participants; Analysis 5.7). However, confidence intervals indicated imprecision, and included the potential for harm as well as no difference.

We conducted sensitivity analyses removing studies comparing varenicline with no medication rather than placebo, but this had no substantial impact on heterogeneity or results.

##### Serious adverse event

###### Serious adverse events

Twenty‐six studies of 14,356 participants found that more people randomised to receive varenicline reported experiencing serious adverse events than those randomised to receive placebo or no medication (RR 1.23, 95% CI 1.01 to 1.48; I^2^ = 0%; moderate‐certainty evidence; Analysis 5.8). Absolute rates for serious adverse events were 3.3% and 2.7% in varenicline and control arms respectively. King 2022 tested varenicline combined with NRT patch against NRT patch with a varenicline placebo. We did not include this study in our analysis, but it reported two participants with serious adverse events in the varenicline arm and none in the NRT‐alone arm. A sensitivity analysis removing one study comparing varenicline with no medication had no substantial impact on this result.

###### Neuropsychiatric serious adverse events

The point estimate from pooling 22 studies of 7846 people showed that fewer people reported experiencing neuropsychiatric serious adverse events in the varenicline arm compared with placebo or no medication (RR 0.89, 95% CI 0.61 to 1.29; I^2^ = 0%; low‐certainty evidence; Analysis 5.9). However confidence intervals demonstrated imprecision, also encompassing the possibility of more neuropsychiatric serious adverse events in the varenicline arm. A sensitivity analysis removing one study comparing varenicline with no medication had no substantial impact on this result.

###### Cardiac serious adverse events

The point estimate from pooling 18 studies of 7151 people showed that more people reported experiencing cardiac serious adverse events in the varenicline arm compared with placebo or no medication (RR 1.20, 95% CI 0.79 to 1.84; I^2^ = 0%; low‐certainty evidence; Analysis 5.10). However confidence intervals demonstrated imprecision, also encompassing the possibility of no difference or fewer serious adverse events in the varenicline arm. A sensitivity analysis removing one study comparing varenicline with no medication had no substantial impact on this result.

#### Varenicline: variations in usage

##### Low‐dose varenicline versus placebo

Four studies tested varenicline at doses lower than standard (under 2 mg per day). Three studies tested 1 mg per day compared with placebo and still found that more people quit in the varenicline arm (RR 1.87, 95% CI 1.35 to 2.60; 906 participants; Analysis 6.1.1). There was substantial heterogeneity (I^2^ = 71%), however in all cases point estimates favoured varenicline.

Niaura 2008 allowed participants to choose their own dose of varenicline, ranging between 0.5 mg and 2.0 mg daily, and found more people in the varenicline arm quit than in the placebo arm (RR 2.92, 95% CI 1.57 to 5.41; 320 participants; Analysis 6.1.2).

##### Higher‐dose varenicline versus lower‐dose varenicline

Four studies compared varenicline at 2 mg per day with 1 mg per day and did not provide clear evidence of a difference in how many people quit (RR 1.12, 95% CI 0.97 to 1.30; 1563 participants; I^2^ = 44%; Analysis 6.2.1); however confidence intervals encompassed potential benefit and a slight disadvantage of the higher dose. Nides 2006 also compared other dosages and also did not find clear evidence of a difference in quit rates among participants randomised to receive 2 mg compared with 0.3 mg per day (RR 1.84 95% CI 0.89 to 3.84; 253 participants; Analysis 6.2.2) or 1 mg compared with 0.3 mg per day (RR 0.71, 95% CI 0.28 to 1.81; 254 participants; Analysis 6.2.3). However, in both cases confidence intervals were wide and may indicate benefit and harm of higher doses.

##### Longer‐duration varenicline versus standard‐duration varenicline

Three studies tested extended durations of varenicline compared with standard duration of varenicline (12 weeks). We found no clear evidence of a difference as a result of extending varenicline treatment to 24 weeks (RR 0.97, 95% CI 0.77 to 1.23; I^2^ = 17%; 2 studies, 1458 participants; Analysis 6.3.1) or 52 weeks (RR 1.30, 95% CI 0.70 to 2.43; 1 study; 107 participants; Analysis 6.3.2). However, the confidence intervals indicate imprecision and uncertainty in the point estimates.

##### Six weeks versus one week of varenicline preloading

Bohadana 2020 tested varenicline with a preloading period of six weeks before quit date against the standard one week of preloading and found that more people in the six‐week preloading arm quit than in the one‐week arm (RR 5.60, 95% CI 2.24 to 14.02; 242 participants).

#### Varenicline in specific patient groups

Studies testing varenicline against placebo or no medication in specific patient populations did not find any clear evidence of varenicline working differently in these groups than in the general population.

Analyses found more people successfully quitting in the varenicline arm than in control in studies of people with cardiovascular disease (RR 1.88, 95% CI 1.44 to 2.47; I^2^ = 81%; 2 studies, 1006 participants; Analysis 7.1); schizophrenia, bipolar disorder, or another psychiatric disorder (RR 2.26, 95% CI 1.78 to 2.86; I^2^ = 0%; 3 studies, 2245 participants; Analysis 7.4); depression (RR 2.17, 95% CI 1.45 to 3.24; I^2^ = 0%; 2 studies, 745 participants; Analysis 7.5); HIV (RR 1.96, 95% CI 1.06 to 3.63; I^2^ = 0%; 2 studies, 427 participants; Analysis 7.8); and chronic obstructive pulmonary disease (RR 1.47, 95% CI 1.28 to 1.69; I^2^ = 94%; 4 studies of 860 participants; Analysis 7.2).

Treating these trials as a subgroup of the main analysis (Analysis 5.1), testing for subgroup differences showed no evidence that varenicline works differently from in the general population: cardiovascular disease, P = 0.24, I^2^ = 26.2%; psychiatric disorders, P = 0.40, I^2^ = 0%; depression, P = 0.92, I^2^ = 0%; HIV, P = 0.69, I^2^ = 0%). Testing the COPD result for subgroup difference initially showed evidence that varenicline may work differently, with lower effectiveness, in this population (P < 0.00001, I^2^ = 96.8%), however this difference disappeared when we performed a sensitivity analysis removing studies comparing varenicline with no medication rather than placebo (P = 1.00, I^2^ = 0%).

Analyses presented inconclusive evidence for three patient populations: asthma (RR 1.25, 95% CI 0.38 to 4.14; 1 study; 52 participants; Analysis 7.3), substance use disorder (RR 3.72, 95% CI 0.50 to 27.59; I^2^ = 0%; 2 studies, 294 participants; Analysis 7.6), and alcohol dependence (RR 3.01, 95% CI 0.92 to 9.92; I^2^ = 54%; 3 studies, 195 participants; Analysis 7.7). However, point estimates all favoured varenicline, and wide confidence intervals were likely the result of very low numbers of events. Treating the trials as a subgroup of the main analysis (Analysis 5.1) and testing for subgroup differences showed no evidence that varenicline works differently in these populations (asthma: P = 0.76, I^2^ = 0.4%; substance use disorder: P = 0.61, I^2^ = 0%; alcohol dependence: P = 0.70, I^2^ = 0.4%).

#### Varenicline in specific settings or subgroups

Six studies of 1324 participants tested varenicline against placebo or no medication among hospital inpatients and perioperative patients and found that more people successfully quit in the varenicline arm (RR 1.27, 95% CI 1.12 to 1.43; I^2^ = 58%; Analysis 8.1). Treating these trials as a subgroup of the main analysis (Analysis 5.1), which includes studies conducted in both clinical and community settings such as cessation clinics and university campuses, and testing for subgroup differences did show evidence of subgroup difference, with lower effectiveness (P < 0.00001; I^2^ = 98.6%). Heterogeneity remained when we performed a sensitivity analysis removing studies comparing varenicline with no medication rather than placebo (P < 0.00001; I^2^ = 96%).

Gonzales 2014 tested varenicline against placebo among people who had previously used varenicline for two weeks or more, at least three months prior to admission to the study, and had not successfully quit but were motivated to try again. This single study found that more people successfully quit in the varenicline arm (RR 6.15, 95% CI 2.98 to 12.70; 494 participants; Analysis 8.2).

Two studies of 114 participants tested varenicline against placebo among light smokers and found that more people successfully quit in the varenicline arm (RR 4.16, 95% CI 1.58 to 10.97; I^2^ = 0%; Analysis 8.3). Treating these trials as a subgroup of the main analysis (Analysis 5.1) and testing for subgroup differences showed no evidence that varenicline works differently in this population (P = 0.20, I^2^ = 39.3%).

#### Varenicline versus bupropion

##### Smoking cessation

Nine studies of 7560 participants compared varenicline with bupropion and found that more people quit smoking when using varenicline (RR 1.36, 95% CI 1.25 to 1.49; I^2^ = 0%; high‐certainty evidence; Analysis 9.1).

Johns 2017b randomised 300 participants to receive varenicline, bupropion, or both. They did not provide any useable results data but reported that more people quit in the combined varenicline and bupropion arm compared with the bupropion‐ or varenicline‐alone arms. However, only an abstract was available for this study, and without further information, this result should be treated with caution.

##### Adverse events

Studies comparing varenicline with bupropion found that people randomised to receive varenicline were more likely to report experiencing nausea (RR 2.46, 95% CI 2.20 to 2.75; I^2^ = 0%; 4 studies, 5808 participants; Analysis 9.2), abnormal dreams (RR 1.56, 95% CI 1.39 to 1.76; I^2^ = 0%; 4 studies, 5808 participants; Analysis 9.4), and headache (RR 1.24, 95% CI 1.06 to 1.45; I^2^ = 13%; 3 studies, 4888 participants; Analysis 9.5). However, people randomised to receive varenicline were less likely to report experiencing insomnia (RR 0.84, 95% CI 0.75 to 0.93; I^2^ = 75%; 6 studies, 6789 participants; Analysis 9.3).

Two studies of 4210 people did not find evidence a difference between those randomised to receive varenicline or bupropion in reported rates of depression (RR 0.90, 95% CI 0.35 to 2.35; I^2^ = 0%; Analysis 9.6) or suicidal ideation (RR 1.99, 95% CI 0.18 to 21.93; I^2^ not estimable as events only in 1 study; Analysis 9.7).

##### Serious adverse events

###### Serious adverse events

Five studies of 5317 people did not find evidence of a clear difference in the number of people reporting experiencing serious adverse events (RR 0.89, 95% CI 0.61 to 1.31; I^2^ = 0%; low‐certainty evidence; Analysis 9.8).

###### Neuropsychiatric serious adverse events

Two studies of 866 people did not find evidence of a clear difference in the number of people reporting experiencing neuropsychiatric serious adverse events (RR 1.05, 95% CI 0.16 to 7.04; I^2^ = 10%; low‐certainty evidence; Analysis 9.9), though confidence intervals were very wide.

###### Cardiac serious adverse events

Two studies of 866 people did not find evidence of a clear difference in the number of people reporting experiencing cardiac serious adverse events (RR 3.17, 95% CI 0.33 to 30.18; I^2^ = 0%; low‐certainty evidence; Analysis 9.10), though confidence intervals were very wide.

#### Varenicline versus nicotine replacement therapy (NRT) monotherapy

##### Smoking cessation

Eleven studies of 7572 participants compared varenicline with NRT monotherapy and found that more people quit smoking in the varenicline arm (RR 1.25, 95% CI 1.14 to 1.37; I^2^ = 28%; high‐certainty evidence; Analysis 10.1).

##### Adverse events

Studies comparing varenicline with NRT monotherapy found that people randomised to receive varenicline were more likely to report experiencing nausea (RR 2.69, 95% CI 2.41 to 3.01; I^2^ = 59%; 6 studies, 6500 participants; Analysis 10.2) and headache (RR 1.14, 95% CI 1.01 to 1.28; I^2^ = 69%; 4 studies, 6287 participants; Analysis 10.5). However, they did not find evidence of a clear difference in the number of people reporting experiencing insomnia (RR 1.08, 95% CI 0.96 to 1.21; I^2^ = 42%; 5 studies, 6319 participants; Analysis 10.3), abnormal dreams (RR 0.93, 95% CI 0.83 to 1.05; I^2^ = 67%; 4 studies, 5803 participants; Analysis 10.4), or depression (RR 0.94, 95% CI 0.76 to 1.16; I^2^ = 0%; 3 studies, 5541 participants; Analysis 10.6). The pooled point estimate from two studies found a higher rate of suicidal ideation among participants in the varenicline arm (RR 5.00, 95% CI 0.87 to 28.77; I^2^ = 0%; 2 studies, 4876 participants; Analysis 10.7), but confidence intervals were very wide and incorporated the potential for no difference or reduced harm. Statistical heterogeneity was substantial in several analyses, but we decided to present the pooled estimate because the point estimates of individuals studies were almost entirely in the same direction in each analysis.

##### Serious adverse events

###### Serious adverse events

Six studies of 6535 people comparing varenicline with NRT monotherapy found that people randomised to receive varenicline were less likely to report experiencing serious adverse events (RR 0.70, 95% CI 0.50 to 0.99; I^2^ = 24%; low‐certainty evidence; Analysis 10.8).

###### Neuropsychiatric serious adverse events

Only one study measured neuropsychiatric serious adverse events, and reported no events in either arm (Rohsenow 2017; 137 participants).

###### Cardiac serious adverse events

Only one study measured cardiac serious adverse events, and reported no events in either arm (Rohsenow 2017; 137 participants).

#### Varenicline versus combination NRT

##### Smoking cessation

Five studies of 2344 participants compared varenicline with combination NRT and did not detect evidence of a clear difference in the number of people who quit smoking, although confidence intervals indicate imprecision, which reduces our certainty in the effect (RR 1.02, 95% CI 0.87 to 1.20; I^2^ = 0%; low‐certainty evidence; Analysis 11.1).

##### Adverse events

Studies comparing varenicline with combination NRT found that people randomised to receive varenicline were more likely to report experiencing nausea (RR 1.76, 95% CI 1.45 to 2.15; I^2^ = 47%; 3 studies, 1609 participants; Analysis 11.2), insomnia (RR 1.40, 95% CI 1.15 to 1.70; I^2^ = 82%; 3 studies, 1609 participants; Analysis 11.3), and abnormal dreams (RR 1.59, 95% CI 1.22 to 2.08; 1 study; 549 participants; Analysis 11.4). However, they did not find evidence of a clear difference in the number of people reporting experiencing headache (RR 0.98, 95% CI 0.78 to 1.23; I^2^ = 0%; 3 studies, 1609 participants; Analysis 11.5), depression (RR 1.08, 95% CI 0.83 to 1.40; I^2^ = 82%; 3 studies, 1609 participants; Analysis 11.6), or suicidal ideation (RR 0.94, 95% CI 0.06 to 14.79; I^2^ not estimable as events only in 1 study; 2 studies, 764 participants; Analysis 11.7). Statistical heterogeneity was substantial in several analyses, but we decided to present the pooled estimate because the point estimates of individual studies were almost entirely in the same direction.

##### Serious adverse events

###### Serious adverse events

Pooled data from four studies of 1852 people showed more people in the varenicline arm reporting serious adverse events compared with combination NRT (RR 2.15, 95% CI 0.49 to 9.46; I^2^ = 0%; low‐certainty evidence; Analysis 11.8). However, confidence intervals were very wide and included the potential for no difference or reduced risk.

###### Neuropsychiatric serious adverse events

Two studies of 764 people reported the number of people reporting neuropsychiatric serious adverse events. While the point estimate suggested participants receiving varenicline were more likely to report experiencing neuropsychiatric serious adverse events, confidence intervals were extremely wide, and incorporated both benefit and harm (RR 4.69, 95% CI 0.23 to 96.50; I^2^ not estimable as events only in 1 study; low‐certainty evidence; Analysis 11.9).

###### Cardiac serious adverse events

Two studies of 819 people reported the number of people reporting cardiac serious adverse events. While the point estimate suggested that participants receiving varenicline were less likely to report experiencing cardiac serious adverse events, confidence intervals were very wide, and incorporated both benefit and harm (RR 0.32, 95% CI 0.01 to 7.88; I^2^ not estimable as events only in 1 study; low‐certainty evidence; Analysis 11.10).

#### Varenicline versus e‐cigarettes

##### Smoking cessation

One study of 54 participants compared varenicline with e‐cigarettes and found that more people quit smoking in the varenicline arm (RR 3.25, 95% CI 1.21 to 8.71; very low‐certainty evidence; Analysis 12.1).

##### Adverse events

The only one of our adverse event outcomes reported by this study was nausea. While the point estimate suggested that participants receiving varenicline were more likely to report experiencing nausea, confidence intervals were extremely wide, and incorporated both benefit and harm (RR 3.00, 95% CI 0.33 to 27.06; Analysis 12.2).

##### Serious adverse events

While the study reported serious adverse events, neuropsychiatric serious adverse events, and cardiac serious adverse events as outcomes, they reported no events among study participants in either arm.

#### Dianicline versus placebo

##### Smoking cessation

Tonstad 2011 (602 participants) compared dianicline with placebo and did not detect evidence of a clear difference in the number of people who quit smoking (RR 1.20, 95% CI 0.82 to 1.75; Analysis 13.1), however, results were imprecise and confidence intervals encompass the possibility of benefit and harm of dianicline.

##### Adverse events

Tonstad 2011 reported that more people randomised to receive dianicline reported experiencing nausea (RR 2.83, 95% CI 1.88 to 4.27; 602 participants; Analysis 13.2) and depression (RR 8.05, 95% CI 1.01 to 63.99; 602 participants; Analysis 13.4) than those in the placebo arm. They did not detect a difference in the number of people reporting experiencing headaches (RR 1.23, 95% CI 0.82 to 1.85; 602 participants; Analysis 13.3). The study did not measure our other adverse event outcomes.

##### Serious adverse events

Tonstad 2011 did not detect a difference in rates of serious adverse events (RR 1.01, 95% CI 0.20 to 4.95; 602 participants; Analysis 13.5) or cardiac serious adverse events (RR 1.01, 95% CI 0.06 to 16.02; 602 participants; Analysis 13.6) between participants in the dianicline and placebo study arms. However, in both instances confidence intervals were very wide and included the potential for both harm and benefit. The study did not measure neuropsychiatric serious adverse events.

## Discussion

### Summary of main results

This review includes eight studies that investigated cytisine use in just under 9000 people, 68 studies that investigated varenicline use in over 37,000 people, and one study that investigated dianicline use in 602 people. Forty‐five of these studies are new to this update.

We found moderate‐certainty evidence that cytisine probably helps more people to quit smoking than placebo. While people randomised to receive cytisine were more likely to experience adverse events than those in the placebo arm, low‐certainty evidence gave no clear indication of an increased risk of serious adverse events. We found no data on neuropsychiatric or cardiac serious adverse events.

Our analysis did not find definitive evidence of a difference in cessation rates between cytisine and varenicline. The point estimate favoured varenicline, but this moderate‐certainty evidence is subject to imprecision and may change as more evidence becomes available. A component network meta‐analysis that is currently underway may reveal a more certain result by also employing data from indirect comparisons (Lindson 2022). Although the point estimates in the separate analyses comparing varenicline with placebo and cytisine with placebo did differ (with the varenicline analysis producing a higher risk ratio) the issue with this type of indirect comparison is it does not adjust for potential differences in baseline event rates. In our analysis, Dogar 2020 was the largest study that compared cytisine with placebo. It recruited people diagnosed with pulmonary tuberculosis who, as part of the behavioural aspect of the intervention, were informed of the dangers of continued tobacco use in people with tuberculosis. This made for a highly motivated population, who also happened to smoke fewer cigarettes per day than in other cytisine trials. These two characteristics may have contributed to the higher placebo arm quit rates in the cytisine studies and minimised the benefit gained from pharmacotherapy. These factors may also explain why the results from Dogar 2020 are less compelling than those of the other studies, and may also account for the statistical heterogeneity in the cytisine versus placebo analysis (I^2^ = 83%). 

Participants in the cytisine arm were less likely to experience nausea or abnormal dreams than those in the varenicline arm, and there was no evidence of a difference in rates of insomnia, headache, depression, or suicidal ideation. The same studies provided low‐certainty evidence of fewer people experiencing serious adverse events in the cytisine arm compared with varenicline. However, in all cases, confidence intervals indicated imprecision, and the potential for more adverse events when using either treatment. We found no data on neuropsychiatric or cardiac serious adverse events.

Low‐certainty evidence suggested that cytisine may help more people to quit than NRT monotherapy (Walker 2014). However, after the initial 25‐day course, participants in the cytisine arm also received vouchers for NRT. This may distort the results as some participants in the cytisine arm may have in fact received two pharmacotherapies, but study authors reported that few participants in the cytisine arm used their NRT vouchers. Low‐certainty evidence did not show a difference in the number of people reporting serious adverse events. We found no data on neuropsychiatric or cardiac serious adverse events.

Evidence on the effect of different lengths of cytisine treatment was sparse and inconclusive. 

There is high‐certainty evidence that varenicline increases the chances of successful smoking cessation by more than two‐fold compared with placebo. Since the previous version of this review was published (Cahill 2016), this estimate has remained stable, despite the growing inclusion of pragmatic trials in real‐world settings and those conducted in particular groups of smokers previously excluded from clinical trials, such as those in lower‐ and middle‐income countries, and in disease‐specific populations. 

We also found high‐certainty evidence that varenicline helped more people to quit than bupropion, or NRT monotherapy, with no clear evidence of difference between varenicline and bupropion in rates of serious adverse events, neuropsychiatric serious adverse events, or cardiac serious adverse events (all low‐certainty evidence), and low‐certainty evidence suggesting reduced risk of serious adverse events compared with NRT. We found no data comparing varenicline with NRT monotherapy for neuropsychiatric or cardiac serious adverse events.

Low‐certainty evidence did not show a difference in quit rates compared with combination NRT, and while low‐certainty evidence suggested potentially increased risk of serious adverse events and neuropsychiatric serious adverse events, and reduced risk of cardiac serious adverse events, in all cases confidence intervals were very wide, encompassing both substantial harm and benefit. 

One small study of 54 people provided very‐low certainty evidence of more people quitting with varenicline than with e‐cigarettes (Ioakeimidis 2018); however this study was at high risk of bias and imprecise due to few events, and while they reported serious adverse events, neuropsychiatric serious adverse events, and cardiac serious adverse events as outcomes, they reported no events among study participants in either arm. Studies that tested varenicline versus placebo in specific populations and settings did not demonstrate varenicline working differently than it does in the general population in disease‐specific groups of patients (e.g. cardiovascular, chronic obstructive pulmonary disease, HIV, schizophrenia and psychiatric disorders, depression, alcohol dependence), or in specific subgroups or settings (e.g. hospital inpatients, light‐smokers, smokers who failed to quit on varenicline previously).

Analyses found evidence of increased rates of adverse events such as nausea, insomnia, abnormal dreams, and headache among people randomised to receive varenicline compared with placebo. However, we found no clear evidence of increased rates of depression or suicidal ideation, although confidence intervals indicated imprecision, and the potential for more or fewer adverse events when using varenicline compared with control. Moderate‐certainty serious adverse event data suggest there may be a 23% increased risk of such events among the varenicline groups compared with the controls. However, serious adverse events were still rare (2.7% to 4% of people on varenicline, compared with 2.7% of people without) and this finding is based on simple counts across the trials of participants reporting one or more such events, thus not distinguishing between events attributed and those unrelated to treatment. We did not find evidence of an increased risk of neuropsychiatric serious adverse events, but pooled results did suggest a potential increased risk of cardiac serious adverse events, although again these results were subject to imprecision, and we deemed this evidence to be low certainty because of its compatibility with both increased and decreased risk of harm.

One trial compared dianicline with placebo for smoking cessation and the results were inconclusive (Tonstad 2011).

### Overall completeness and applicability of evidence

We conducted systematic searches of multiple online databases, including clinical trials registries and followed Cochrane methods for screening. We therefore expect that any published trials we have missed will be through chance rather than systematic error. We were able to assess publication bias for two comparisons by constructing funnel plots: varenicline versus placebo (Figure 4), and varenicline versus NRT monotherapy (Figure 5). Figure 5 appears to show a small amount of asymmetry that may suggest a lack of smaller trials with negative findings. However, the number of studies is still very low with few smaller studies, and so we cannot treat this as definitive evidence of publication bias. These were the only cessation comparisons with enough studies to construct a funnel plot, so we were unable to assess publication bias for other comparisons. As such, we cannot ignore the possibility of publication bias for some comparisons in this review.

**4:**
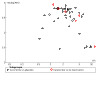
Funnel plot of comparison 6: varenicline (1.0 mg 2/d) vs placebo, outcome: 6.1 abstinence at longest follow‐up

**5:**
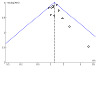
Funnel plot of comparison 13: varenicline vs nicotine replacement therapy monotherapy, outcome: 13.1 abstinence at longest follow‐up

The benefits of varenicline for smoking cessation is now well established, with the point estimate remaining unchanged as more studies (including non‐manufacturer‐ (Pfizer) funded trials) accumulate. Trials are now being conducted and reported in areas where the evidence is less comprehensive, such as testing cytisine (against placebo and varenicline), and testing varenicline in specific populations and settings, and in variations of treatment dose or duration.

### Quality of the evidence

We judged the evidence comparing varenicline with placebo for smoking cessation to be of high certainty. While we detected moderate heterogeneity (I^2^ = 60%), all but three studies had a point estimate that favoured varenicline over placebo, so we did not downgrade on this basis. The effectiveness of varenicline for smoking cessation has remained constant through several updates of this review, and we think it is unlikely to change with further evidence. We judged the evidence on serious adverse events to be of moderate certainty, downgraded because of imprecision. For cardiac and neuropsychiatric serious adverse events, we downgraded evidence to low because of imprecision. Despite respectable numbers of studies and participants in each analysis, because of the rarity of these kinds of adverse events, there were very few events in the analyses, with some studies reporting no events at all.

We judged the evidence comparing cytisine with placebo to be of moderate certainty for smoking cessation and low certainty for serious adverse events, downgraded due to substantial unexplained heterogeneity and imprecision, respectively. We judged the evidence comparing cytisine with varenicline for smoking cessation and serious adverse events to be of moderate and low certainty, respectively, downgraded for imprecision in both cases.

We judged the evidence comparing varenicline with bupropion and NRT monotherapy for smoking cessation to be of high certainty, and with combination NRT to be of low certainty, limited by imprecision. Only one small study compared varenicline with e‐cigarettes for smoking cessation, and we graded this evidence as of very low certainty because of imprecision and risk of bias.

### Potential biases in the review process

We have followed standard Cochrane methodology, which endeavours to minimise biases in the review process, so we are confident that any errors in our data will be through chance rather than systematic error. However, it is impossible to rule out individual errors in the review process.

A potential limitation to our review is that for data on harms we relied on adverse events and serious adverse events as defined by papers reporting included studies. This does not take into account whether those events were genuinely attributable to the tested interventions. Further, we only considered the number of participants reporting these events, which does not account for people who experienced more than one event.

Another potential limitation is that the majority of varenicline trials reported in this review received either funding or study medication from Pfizer Inc, the manufacturers of varenicline. Evidence from systematic reviews suggests that industry‐funded trials, although conducted to a high standard, are more likely to have outcomes favourable to the product sponsor than studies with other sponsors (Etter 2007; Walsh 2011). However, we deem the provision of study medication less likely to amount to the kind of sponsorship that may bias results, and modern trials increasingly report funders and medication providers as having no involvement in trial conduct or decision making.

### Agreements and disagreements with other studies or reviews

Reviews of controlled studies of cytisine have focused upon its potential as an established and affordable aid to smoking cessation (Etter 2006; Etter 2008; Tutka 2005; Tutka 2006; Tutka 2008). Many of the early cytisine studies excluded from this review are discussed and evaluated in Etter 2006, who concluded that cytisine may be effective for smoking cessation. A systematic review and network meta‐analysis (Leaviss 2014), compared the benefits and cost‐effectiveness of cytisine (2 trials: Vinnikov 2008; West 2011), with varenicline (21 trials). While the analysis found both treatments to be effective for smoking cessation, cytisine delivered more quality‐adjusted life‐years at a lower cost than varenicline. Cytisine was also associated with lower rates of headache and nausea than varenicline. Our analyses on harms, using direct evidence, found lower rates of nausea, but did not find evidence of difference in rates of headache. A recent review of cytisine found similar results to ours, though with a slightly higher point estimate for smoking cessation (Tutka 2019). This is likely because they had broader inclusion criteria than our review, and included studies with shorter follow‐up periods.

A Cochrane overview and network meta‐analysis of a number of pharmacological interventions for smoking cessation assessed 12 Cochrane Reviews published to November 2012 (Cahill 2013), and therefore drew on the previous version of this review. Comparisons between varenicline, bupropion and single‐treatment NRT found varenicline to be superior to both treatments (OR 1.59, 95% credible interval 1.29 to 1.96 and OR 1.57, 95% credible interval 1.29 to 1.91, respectively). Varenicline demonstrated comparable benefits for smoking cessation to combination NRT (OR 1.06, 95% credible interval 0.75 to 1.48), but the number of NRT trials informing this comparison was low (9 trials). This review is currently being updated (Lindson 2022). A 2012 network meta‐analysis (Mills 2012), comparing high‐dose and combination NRT versus varenicline and versus bupropion across 146 RCTs, found varenicline (11 trials) to be superior to placebo and to bupropion at all time points, and similar in benefits for smoking cessation to standard and to high‐dose NRT, in line with our findings. A more recent systematic review with network meta‐analysis reported similar findings to ours, reporting a benefit from varenicline compared with placebo (OR 2.69, 95% CI 2.27 to 3.19), bupropion (OR 1.46, 95% CI 1.18 to 1.81), and standard‐dose NRT (OR 1.32, 95% CI 1.05 to 1.65; Thomas 2020). However, they did not find evidence of increased rates of serious adverse events amongst those randomised to receive varenicline compared with placebo (OR 1.09, 95% CI 0.91 to 1.34), contrary to the increased risk we detected. This may be due to their choice of a random‐effects rather than fixed‐effect model for their analysis. They did not find clear evidence of a difference in cardiac serious adverse events (OR 0.76, 95% CI 0.41 to 1.25) and neuropsychiatric serious adverse events (OR 0.96, 95% CI 0.76 to 1.21), which is in line with our findings.

An earlier systematic review and meta‐analysis of 39 RCTs (10,761 participants) by the same team assessed the risk of neuropsychiatric adverse events among users of varenicline (Thomas 2015). In line with our findings, the authors found no clear evidence of an increased risk of suicide or attempted suicide (Peto odds ratio (OR) 1.67, 95% CI 0.33 to 8.57), suicidal ideation (Peto OR 0.58, 95% CI 0.28 to 1.20), depression (Peto OR 0.96, 95% CI 0.75 to 1.22) or death (Peto OR 1.05, 95% CI 0.47 to 2.38) associated with varenicline. Lee 2016 compared cardiovascular serious adverse event rates between people randomised to receive varenicline or placebo. They did not find evidence of increased risk of cardiovascular serious adverse events (RR 1.03, 95% CI 0.72 to 1.49) in 38 trials of 12,706 people. This result was consistent among cardiovascular (RR 1.04, 95% CI 0.57 to 1.89) and non‐cardiovascular patients (RR 1.03, 95% CI 0.64 to 1.64).

## Authors' conclusions

**Implications for practice**

- Cytisine is likely to help more people to quit smoking than placebo or no medication.
- Varenicline at standard dosage (1.0 mg twice a day) increased the chances of successful long‐term smoking cessation by more than two‐fold compared with placebo. We did not find evidence that varenicline is less effective in any of the specific populations we investigated.
- Varenicline is more effective at helping people to quit smoking than bupropion, or a single form of nicotine replacement therapy, and may be as effective as or more effective than dual‐form nicotine replacement therapy.
- People taking varenicline are probably more likely to experience serious adverse events than those not taking it, and while there may be increased risk of cardiac serious adverse events and decreased risk of neuropsychiatric serious adverse events, evidence was compatible with both benefit and harm.
- Cytisine may lead to fewer people reporting serious adverse events than varenicline. There may be a benefit from varenicline for quitting smoking, however further evidence could strengthen this finding or demonstrate a benefit from cytisine.

**Implications for research**

- Future trials should test the effectiveness and safety of cytisine compared with varenicline and other pharmacotherapies, and should also test variations in dose and duration.
- There is limited benefit to be gained from more trials testing the effect of standard dose varenicline compared with placebo for smoking cessation.
- Further varenicline trials should test the effect of variations in dose and duration and preloading varenicline before quitting, and may be useful in specific populations and settings where there is a plausible rationale that the effect may differ.

## What's new

**Table:** 

**Date**	**Event**	**Description**
4 May 2023	New search has been performed	New searches conducted 29 April 2022 adding 45 new studies
4 May 2023	New citation required and conclusions have changed	New searches conducted 29 April 2022. Analyses and conclusions updated

## History

Protocol first published: Issue 3, 2006 Review first published: Issue 1, 2007

**Table:** 

**Date**	**Event**	**Description**
31 January 2016	New citation required and conclusions have changed	Additional comparisons. Analyses expanded and restructured. SAE information updated
31 January 2016	New search has been performed	39 trials of varenicline now included
16 May 2013	Amended	Minor change made to labelling on forest plot.
14 March 2012	New search has been performed	Seven new included studies (5 varenicline, 1 cytisine, 1 dianicline) and 14 new excluded studies added, plus safety data.
14 March 2012	New citation required and conclusions have changed	Safety profile modified, as new possible cardiovascular and psychiatric adverse events information incorporated. Efficacy findings unchanged but confirmed.
13 January 2011	Amended	Vinnikov trial of cytisine added to Studies awaiting Classification, for inclusion in next update.
8 November 2010	New search has been performed	Six new RCTs added; sources of funding added for all trials. Ongoing trials section expanded.
8 November 2010	New citation required and conclusions have changed	Surveillance data and secondary analyses do not support fears about safety. Efficacy conclusions strengthened but unchanged.
17 July 2008	Amended	Date of last search amended (2007 corrected to 2008); Source of support added.
12 May 2008	New citation required and conclusions have changed	Three new included trials, switch in the MA metric from OR to RR, updated background section and new safety information.
15 March 2008	New search has been performed	New search conducted.
30 August 2007	Amended	Converted to new review format.
15 November 2006	New citation required and conclusions have changed	Substantive amendment.

## Appendices

### Appendix 1. Search strategy

CTAG Specialised Register (CRS web)

1. (cytisine or Tabex or dianicline or varenicline or champix or chantix):TI,AB,MH,EMT,XKY,KY,KW 2. MeSH DESCRIPTOR Nicotine WITH AG AI 3. MeSH DESCRIPTOR Nicotinic Agonists 4. MeSH DESCRIPTOR Nicotinic Antagonists 5. nicotinic agonist*:TI,AB,MH,EMT,XKY,KY,KW 6. nicotinic antagonist*:TI,AB,MH,EMT,XKY,KY,KW 7. nicotin* NEAR2 partial:TI,AB,MH,EMT,XKY,KY,KW #1 OR #2 OR #3 OR #4 OR #5 OR #6 OR #7  

### Appendix 2. Glossary of tobacco‐related terms

**Table:**

**Term**	**Definition**
**Abstinence**	A period of being quit, i.e. stopping the use of cigarettes or other tobacco products. May be defined in various ways; see also: point prevalence abstinence; prolonged abstinence; continuous/sustained abstinence
**Biochemical verification**	Also called 'biochemical validation' or 'biochemical confirmation' A procedure for checking a tobacco user's report that he or she has not smoked or used tobacco. It can be measured by testing levels of nicotine or cotinine or other chemicals in blood, urine, or saliva, or by measuring levels of carbon monoxide in exhaled breath or in blood.
**Bupropion**	A pharmaceutical drug originally developed as an antidepressant, but now also licensed for smoking cessation; trade names Zyban, Wellbutrin (when prescribed as an antidepressant)
**Carbon monoxide (CO)**	A colourless, odourless, highly poisonous gas found in tobacco smoke and in the lungs of people who have recently smoked, or (in smaller amounts) in people who have been exposed to tobacco smoke. May be used for biochemical verification of abstinence.
**Cessation**	Also called 'quitting' The goal of treatment to help people achieve abstinence from smoking or other tobacco use, also used to describe the process of changing behaviour.
**Continuous abstinence**	Also called 'sustained abstinence' A measure of cessation often used in clinical trials involving avoidance of all tobacco use since the quit day until the time the assessment is made. The definition occasionally allows for lapses. This is the most rigorous measure of abstinence.
**'Cold turkey'**	Quitting abruptly, and/or quitting without behavioural or pharmaceutical support.
**Craving**	A very intense urge or desire (to smoke) See: Shiffman 2004
**Dopamine**	A neurotransmitter in the brain that regulates mood, attention, pleasure, reward, motivation and movement
**Efficacy**	Also called 'treatment effect' or 'effect size' The difference in outcome between the experimental and control groups
**Harm reduction**	Strategies to reduce harm caused by continued tobacco/nicotine use, such as reducing the number of cigarettes smoked, or switching to different brands or products, e.g. potentially reduced exposure products (PREPs), smokeless tobacco
**Lapse/slip**	Terms sometimes used for a return to tobacco use after a period of abstinence. A lapse or slip might be defined as a puff or two on a cigarette. This may proceed to relapse, or abstinence may be regained. Some definitions of continuous, sustained or prolonged abstinence require complete abstinence, but some allow for a limited number or duration of slips. People who lapse are very likely to relapse, but some treatments may have their effect by helping people recover from a lapse.
**nAChR**	Neural nicotinic acetylcholine receptors Areas in the brain that are thought to respond to nicotine, forming the basis of nicotine addiction by stimulating the overflow of dopamine
**Nicotine**	An alkaloid derived from tobacco, responsible for the psychoactive and addictive effects of smoking
**Nicotine replacement therapy (NRT)**	A smoking cessation treatment in which nicotine from tobacco is replaced for a limited period by pharmaceutical nicotine. This reduces the craving and withdrawal experienced during the initial period of abstinence while users are learning to be tobacco‐free. The nicotine dose can be taken through the skin, using patches, by inhaling a spray, or by mouth using gum or lozenges.
**Outcome**	Often used to describe the result being measured in trials that is of relevance to the review. For example smoking cessation is the outcome used in reviews of ways to help smokers quit. The exact outcome in terms of the definition of abstinence and the length of time that has elapsed since the quit attempt was made may vary from trial to trial.
**Pharmacotherapy**	A treatment using pharmaceutical drugs, e.g. nicotine replacement therapy, bupropion
**Point prevalence abstinence (PPA)**	A measure of cessation based on behaviour at a particular point in time, or during a relatively brief specified period, e.g. 24 hours, 7 days. It may include a mixture of recent and long‐term quitters. cf. prolonged abstinence, continuous abstinence
**Prolonged abstinence**	A measure of cessation that typically allows a 'grace period' following the quit date (usually of about two weeks), to allow for slips/lapses during the first few days when the effect of treatment may still be emerging. See: Hughes 2003
**Relapse**	A return to regular smoking after a period of abstinence
**Secondhand smoke**	Also called passive smoking or environmental tobacco smoke (ETS) A mixture of smoke exhaled by smokers and smoke released from smouldering cigarettes, cigars, pipes, bidis, etc. The smoke mixture contains gases and particulates, including nicotine, carcinogens and toxins.
**Self‐efficacy**	The belief that one will be able to change one's behaviour, e.g. to quit smoking
**SPC [Summary of Product Characteristics]**	Advice from the manufacturers of a drug, agreed with the relevant licensing authority, to enable health professionals to prescribe and use the treatment safely and effectively.
**Tapering**	A gradual decrease in dose at the end of treatment, as an alternative to abruptly stopping treatment
**Titration**	A technique of dosing at low levels at the beginning of treatment, and gradually increasing to full dose over a few days, to allow the body to get used to the drug. It is designed to limit side effects.
**Withdrawal**	A variety of behavioural, affective, cognitive and physiological symptoms, usually transient, which occur after use of an addictive drug is reduced or stopped. See: Shiffman 2004

## Data and analyses

**Comparison 1:** Cytisine vs placebo or no medication

Outcome or subgroup title	No. of studies	No. of participants	Statistical method	Effect size
1.1 Abstinence at longest follow‐up	5		Risk Ratio (M‐H, Fixed, 95% CI)	Subtotals only
1.1.1 Cytisine vs placebo	4	4623	Risk Ratio (M‐H, Fixed, 95% CI)	1.30 [1.15, 1.47]
1.1.2 Cytisine vs no medication	1	869	Risk Ratio (M‐H, Fixed, 95% CI)	4.44 [3.06, 6.46]
1.2 Adverse events	4	4052	Risk Ratio (M‐H, Fixed, 95% CI)	1.22 [1.07, 1.39]
1.3 Serious adverse events	3	3781	Risk Ratio (M‐H, Fixed, 95% CI)	1.04 [0.78, 1.37]

**Comparison 2:** Cytisine: longer vs shorter duration

Outcome or subgroup title	No. of studies	No. of participants	Statistical method	Effect size
2.1 Abstinence at longest follow‐up	1	470	Risk Ratio (M‐H, Fixed, 95% CI)	1.28 [0.98, 1.67]
2.1.1 84 days vs 40 days	1	470	Risk Ratio (M‐H, Fixed, 95% CI)	1.28 [0.98, 1.67]

**Comparison 3:** Cytisine vs varenicline

Outcome or subgroup title	No. of studies	No. of participants	Statistical method	Effect size
3.1 Abstinence at longest follow‐up	2	2131	Risk Ratio (M‐H, Fixed, 95% CI)	0.83 [0.66, 1.05]
3.2 Nausea	2	2017	Risk Ratio (M‐H, Fixed, 95% CI)	0.41 [0.33, 0.50]
3.3 Abnormal dreams	2	2017	Risk Ratio (M‐H, Fixed, 95% CI)	0.60 [0.50, 0.73]
3.4 Insomnia	2	2017	Risk Ratio (M‐H, Fixed, 95% CI)	0.90 [0.73, 1.10]
3.5 Headache	2	2017	Risk Ratio (M‐H, Fixed, 95% CI)	1.02 [0.79, 1.33]
3.6 Depression	1	679	Risk Ratio (M‐H, Fixed, 95% CI)	3.04 [0.12, 74.47]
3.7 Suicidal ideation	2	2017	Risk Ratio (M‐H, Fixed, 95% CI)	0.33 [0.01, 8.02]
3.8 SAEs	2	2017	Risk Ratio (M‐H, Fixed, 95% CI)	0.67 [0.44, 1.03]

**Comparison 4:** Cytisine vs NRT

Outcome or subgroup title	No. of studies	No. of participants	Statistical method	Effect size
4.1 Abstinence at longest follow‐up	1	1310	Risk Ratio (M‐H, Fixed, 95% CI)	1.43 [1.13, 1.80]
4.2 Nausea	1	1310	Risk Ratio (M‐H, Fixed, 95% CI)	15.00 [3.60, 62.51]
4.3 SAEs	1	1310	Risk Ratio (M‐H, Fixed, 95% CI)	1.15 [0.76, 1.75]

**Comparison 5:** Varenicline 2 mg per day vs placebo or no medication

Outcome or subgroup title	No. of studies	No. of participants	Statistical method	Effect size
5.1 Abstinence at longest follow‐up	46		Risk Ratio (M‐H, Fixed, 95% CI)	Subtotals only
5.1.1 Varenicline vs placebo	41	17395	Risk Ratio (M‐H, Fixed, 95% CI)	2.32 [2.15, 2.51]
5.1.2 Varenicline vs no medication	5	1050	Risk Ratio (M‐H, Fixed, 95% CI)	1.57 [1.37, 1.80]
5.2 Nausea	36	17080	Risk Ratio (M‐H, Fixed, 95% CI)	2.61 [2.44, 2.80]
5.3 Insomnia	35	16803	Risk Ratio (M‐H, Fixed, 95% CI)	1.37 [1.27, 1.47]
5.4 Abnormal dreams	32	16211	Risk Ratio (M‐H, Fixed, 95% CI)	1.82 [1.67, 1.97]
5.5 Headache	31	16326	Risk Ratio (M‐H, Fixed, 95% CI)	1.11 [1.03, 1.19]
5.6 Depression	32	15922	Risk Ratio (M‐H, Fixed, 95% CI)	1.05 [0.91, 1.20]
5.7 Suicidal ideation	22	12343	Risk Ratio (M‐H, Fixed, 95% CI)	0.69 [0.44, 1.08]
5.8 SAEs	26	14356	Risk Ratio (M‐H, Fixed, 95% CI)	1.23 [1.01, 1.48]
5.9 Neuropsychiatric SAEs (not deaths)	22	7846	Risk Ratio (M‐H, Fixed, 95% CI)	0.89 [0.61, 1.29]
5.10 Cardiac SAEs, including deaths	18	7151	Risk Ratio (M‐H, Fixed, 95% CI)	1.20 [0.79, 1.84]

**Comparison 6:** Varenicline: variations in usage

Outcome or subgroup title	No. of studies	No. of participants	Statistical method	Effect size
6.1 Low‐dose varenicline vs placebo	4		Risk Ratio (M‐H, Fixed, 95% CI)	Subtotals only
6.1.1 1 mg per day	3	946	Risk Ratio (M‐H, Fixed, 95% CI)	1.87 [1.35, 2.60]
6.1.2 As desired	1	320	Risk Ratio (M‐H, Fixed, 95% CI)	2.92 [1.57, 5.41]
6.2 Higher‐dose varenicline versus lower‐dose varenicline	4		Risk Ratio (M‐H, Fixed, 95% CI)	Subtotals only
6.2.1 2 mg per day vs 1 mg per day	4	1563	Risk Ratio (M‐H, Fixed, 95% CI)	1.12 [0.97, 1.30]
6.2.2 2 mg per day vs 0.3 mg per day	1	253	Risk Ratio (M‐H, Fixed, 95% CI)	1.84 [0.89, 3.84]
6.2.3 1 mg per day vs 0.3 mg per day	1	254	Risk Ratio (M‐H, Fixed, 95% CI)	0.71 [0.28, 1.81]
6.3 Longer vs standard duration varenicline	3		Risk Ratio (M‐H, Fixed, 95% CI)	Subtotals only
6.3.1 24 weeks vs 12 weeks	2	1458	Risk Ratio (M‐H, Fixed, 95% CI)	0.97 [0.77, 1.23]
6.3.2 52 weeks vs 12 weeks	1	107	Risk Ratio (M‐H, Fixed, 95% CI)	1.30 [0.70, 2.43]
6.4 6 week vs 1 week preloading	1	242	Risk Ratio (M‐H, Fixed, 95% CI)	5.60 [2.24, 14.02]

**Comparison 7:** Varenicline (vs placebo or no medication) in specific patient groups

Outcome or subgroup title	No. of studies	No. of participants	Statistical method	Effect size
7.1 Cardiovascular disease	2	1006	Risk Ratio (M‐H, Fixed, 95% CI)	1.88 [1.44, 2.47]
7.2 COPD	4	860	Risk Ratio (M‐H, Fixed, 95% CI)	1.47 [1.28, 1.69]
7.3 Asthma	1	52	Risk Ratio (M‐H, Fixed, 95% CI)	1.25 [0.38, 4.14]
7.4 Schizophrenia/bipolar/psychiatric disorder	3	2245	Risk Ratio (M‐H, Fixed, 95% CI)	2.26 [1.78, 2.86]
7.5 Depression	2	745	Risk Ratio (M‐H, Fixed, 95% CI)	2.17 [1.45, 3.24]
7.6 Substance use disorder/ methadone‐maintained at 24 weeks	2	294	Risk Ratio (M‐H, Fixed, 95% CI)	3.72 [0.50, 27.59]
7.7 Alcohol‐dependence	3	195	Odds Ratio (M‐H, Fixed, 95% CI)	3.01 [0.92, 9.92]
7.8 HIV	2	427	Risk Ratio (M‐H, Fixed, 95% CI)	1.96 [1.06, 3.63]

**Comparison 8:** Varenicline in specific settings/subgroups

Outcome or subgroup title	No. of studies	No. of participants	Statistical method	Effect size
8.1 Hospital inpatients/perioperative patients	6	1324	Risk Ratio (M‐H, Fixed, 95% CI)	1.27 [1.12, 1.43]
8.2 Smokers with a previous quit attempt on varenicline	1		Risk Ratio (M‐H, Fixed, 95% CI)	Subtotals only
8.3 Light or heavy smokers	2		Risk Ratio (M‐H, Fixed, 95% CI)	Subtotals only
8.3.1 Light smokers	2	114	Risk Ratio (M‐H, Fixed, 95% CI)	4.16 [1.58, 10.97]

**Comparison 9:** Varenicline vs bupropion

Outcome or subgroup title	No. of studies	No. of participants	Statistical method	Effect size
9.1 Abstinence at longest follow‐up	9	7560	Risk Ratio (M‐H, Fixed, 95% CI)	1.36 [1.25, 1.49]
9.2 Nausea	4	5808	Risk Ratio (M‐H, Fixed, 95% CI)	2.46 [2.20, 2.75]
9.3 Insomnia	6	6789	Risk Ratio (M‐H, Fixed, 95% CI)	0.84 [0.75, 0.93]
9.4 Abnormal dreams	4	5808	Risk Ratio (M‐H, Fixed, 95% CI)	1.56 [1.39, 1.76]
9.5 Headache	3	4888	Risk Ratio (M‐H, Fixed, 95% CI)	1.24 [1.06, 1.45]
9.6 Depression	2	4210	Risk Ratio (M‐H, Fixed, 95% CI)	0.90 [0.35, 2.35]
9.7 Suicidal ideation	2	4210	Risk Ratio (M‐H, Fixed, 95% CI)	1.99 [0.18, 21.93]
9.8 SAEs	5	5317	Risk Ratio (M‐H, Fixed, 95% CI)	0.89 [0.61, 1.31]
9.9 Neuropsychiatric SAEs (not deaths)	2	866	Risk Ratio (M‐H, Fixed, 95% CI)	1.05 [0.16, 7.04]
9.10 Cardiac SAEs, including deaths	2	866	Risk Ratio (M‐H, Fixed, 95% CI)	3.17 [0.33, 30.18]

**Comparison 10:** Varenicline vs NRT monotherapy

Outcome or subgroup title	No. of studies	No. of participants	Statistical method	Effect size
10.1 Abstinence at longest follow‐up	11	7572	Risk Ratio (M‐H, Fixed, 95% CI)	1.25 [1.14, 1.37]
10.2 Nausea	6	6500	Risk Ratio (M‐H, Fixed, 95% CI)	2.69 [2.41, 3.01]
10.3 Insomnia	5	6319	Risk Ratio (M‐H, Fixed, 95% CI)	1.08 [0.96, 1.21]
10.4 Abnormal dreams	4	5803	Risk Ratio (M‐H, Fixed, 95% CI)	0.93 [0.83, 1.05]
10.5 Headache	4	6287	Risk Ratio (M‐H, Fixed, 95% CI)	1.14 [1.01, 1.28]
10.6 Depression	3	5541	Risk Ratio (M‐H, Fixed, 95% CI)	0.94 [0.76, 1.16]
10.7 Suicidal ideation	2	4876	Risk Ratio (M‐H, Fixed, 95% CI)	5.00 [0.87, 28.77]
10.8 SAEs	6	6535	Risk Ratio (M‐H, Fixed, 95% CI)	0.70 [0.50, 0.99]
10.9 Neuropsychiatric SAEs (not deaths)	1	0	Risk Ratio (M‐H, Fixed, 95% CI)	Not estimable
10.10 Cardiac SAEs, including deaths	1	0	Risk Ratio (M‐H, Fixed, 95% CI)	Not estimable

**Comparison 11:** Varenicline vs combination NRT

Outcome or subgroup title	No. of studies	No. of participants	Statistical method	Effect size
11.1 Abstinence at longest follow‐up	5	2344	Risk Ratio (M‐H, Fixed, 95% CI)	1.02 [0.87, 1.20]
11.2 Nausea	3	1609	Risk Ratio (M‐H, Fixed, 95% CI)	1.76 [1.45, 2.15]
11.3 Insomnia	3	1609	Risk Ratio (M‐H, Fixed, 95% CI)	1.40 [1.15, 1.70]
11.4 Abnormal dreams	1	549	Risk Ratio (M‐H, Fixed, 95% CI)	1.59 [1.22, 2.08]
11.5 Headache	3	1609	Risk Ratio (M‐H, Fixed, 95% CI)	0.98 [0.78, 1.23]
11.6 Depression	3	1609	Risk Ratio (M‐H, Fixed, 95% CI)	1.08 [0.83, 1.40]
11.7 Suicidal ideation	2	764	Risk Ratio (M‐H, Fixed, 95% CI)	0.94 [0.06, 14.79]
11.8 SAEs	4	1852	Risk Ratio (M‐H, Fixed, 95% CI)	2.15 [0.49, 9.46]
11.9 Neuropsychiatric SAEs (not deaths)	2	764	Risk Ratio (M‐H, Fixed, 95% CI)	4.69 [0.23, 96.50]
11.10 Cardiac SAEs, including deaths	2	819	Risk Ratio (M‐H, Fixed, 95% CI)	0.32 [0.01, 7.88]

**Comparison 12:** Varenicline vs e‐cigarettes

Outcome or subgroup title	No. of studies	No. of participants	Statistical method	Effect size
12.1 Abstinence at longest follow‐up	1	54	Risk Ratio (M‐H, Fixed, 95% CI)	3.25 [1.21, 8.71]
12.2 Nausea	1	54	Risk Ratio (M‐H, Fixed, 95% CI)	3.00 [0.33, 27.06]
12.3 SAEs	1	0	Risk Ratio (M‐H, Fixed, 95% CI)	Not estimable
12.4 Neuropsychiatric SAEs (not deaths)	1	0	Risk Ratio (M‐H, Fixed, 95% CI)	Not estimable
12.5 Cardiac SAEs, including deaths	1	0	Risk Ratio (M‐H, Fixed, 95% CI)	Not estimable

**Comparison 13:** Dianicline vs placebo

Outcome or subgroup title	No. of studies	No. of participants	Statistical method	Effect size
13.1 Abstinence at longest follow‐up	1	602	Risk Ratio (M‐H, Fixed, 95% CI)	1.20 [0.82, 1.75]
13.2 Nausea	1	602	Risk Ratio (M‐H, Fixed, 95% CI)	2.83 [1.88, 4.27]
13.3 Headache	1	602	Risk Ratio (M‐H, Fixed, 95% CI)	1.23 [0.82, 1.85]
13.4 Depression	1	602	Risk Ratio (M‐H, Fixed, 95% CI)	8.05 [1.01, 63.99]
13.5 Serious adverse events	1	602	Risk Ratio (M‐H, Fixed, 95% CI)	1.01 [0.20, 4.95]
13.6 Cardiac SAEs, including deaths	1	602	Risk Ratio (M‐H, Fixed, 95% CI)	1.01 [0.06, 16.02]

## Characteristics of studies

### Characteristics of included studies [ordered by study ID]

**Table:** Anthenelli 2013

***Study characteristics***		
Methods	Country: USA (9 centres) and international (24 centres, across Bosnia & Herzogovina, Croatia, Germany, Hungary, Romania, Russian Federation, Spain) Setting: academic clinical trial centres and SC clinics Aim: to assess the efficacy and safety of 12 weeks of varenicline treatment or placebo for SC, with 40 weeks of non‐treatment follow‐up, in adults with current or past depression (MDD) Study design: double‐blind placebo‐controlled RCT Dates conducted: March 2010 ‐ June 2012	
Participants	525 adult smokers, aged 18‐75, smoking at least 10 CPD, motivated to quit, diagnosed with unipolar MDD without psychotic features. 37% male, mean age 46, average CPD at baseline 22, mean FTND 5.9. Allocated to varenicline (256) or placebo (269) Exclusion criteria: current or past diagnosis of dementia, schizophrenia, schizoaffective disorder, or other psychotic disorder, bipolar I disorder, bipolar II disorder. People with antisocial, schizotypal, or any other personality disorder severe enough to compromise their ability to comply with the study requirements Current use of either bupropion or nortriptyline	
Interventions	Varenicline 1 mg x 2/day, titrated for first week Placebo inactive tablets, same regimen All participants received manual‐guided SC support, telephone support and one‐to‐one 10‐min counselling by the same person where possible. Participants in both groups could reduce the dosage if they wished TQD was set for week 1 visit Treatment period was 12 weeks. Visits at screening, baseline, weekly for weeks 1‐12, and then at weeks 13, 16, 24, 32, 40, 52 (or early termination); phone calls at weeks 14, 20, 28, 36, 44 and 48. Weekly pill counts to assess adherence Safety data were reviewed regularly by an external independent data safety monitoring committee	
Outcomes	Primary: CO‐confirmed CAR for weeks 9‐12 Secondary: CO‐confirmed CAR for weeks 9‐24, 9‐52; 7‐day PPA at weeks 12, 24, 52; AEs and SAEs Verification: CO < 10 ppm	
Notes	New for 2016 update Study funding: "This study was funded by Pfizer. Dr. Anthenelli’s writing of this manuscript was funded, in part, by a Department of Veterans Affairs Merit Review award (NEUA‐003‐08S) and by a National Institute on Alcohol Abuse and Alcoholism grant (AA019720). Dr. Morris was supported, in part, by grants from the University of California, San Francisco, Smoking Cessation Leadership Center, and Colorado Department of Public Health and Environment. Drs. Ramey, Tsilkos, Russ, and Yunis and Ms. Dubrava are employees of Pfizer. Editorial support was provided by Abegale Templar, PhD, of Engage Scientific and funded by Pfizer." Author declaration of interests: not reported	
***Risk of bias***		
**Bias**	**Authors' judgement**	**Support for judgement**
Random sequence generation (selection bias)	Low risk	"Eligible participants were randomly assigned to varenicline or placebo in a 1:1 ratio by using a computer generated, 4‐block randomization scheme at each site."
Allocation concealment (selection bias)	Low risk	"Randomization was stratified by antidepressant medication use at baseline (any vs. none) and baseline Montgomery–Åsberg Depression Rating Scale (MADRS) score (11 vs. 11) (32). Investigators obtained participant identification numbers and randomized study drug assignments by using a Web‐based or telephone call‐in computerized drug management system."
Blinding (performance bias and detection bias) All outcomes	Low risk	"The study drug was supplied in blinded bottles by the sponsor to the study sites, where they were dispensed according to computerized instructions."
Incomplete outcome data (attrition bias) All outcomes	Low risk	68.4% of varenicline group completed study (lost 15.6% in treatment and 16% in follow‐up); 66.6% of placebo group completed study (lost 21.9% in treatment and 11.5% in follow‐up)
Selective reporting (reporting bias)	Low risk	Results reported for all outcomes pre‐specified in NCT record.

**Table:** Ashare 2019

***Study characteristics***		
Methods	Country: USA Setting: not explicitly reported, likely at home and in hospital Aim: to evaluate the safety and efficacy of varenicline for SC among people living with HIV Study design: parallel placebo‐controlled phase 3 RCT Dates conducted: October 2012‐September 2018	
Participants	179 adult smokers all with a confirmed HIV diagnosis, currently smoking and motivated to quit. All treated with antiretroviral therapy with HIV loads < 1000 copies/mL and CD4+ counts > 200 cells/mm^3^. 32% female, mean age 48.6, average CPD at baseline 11.5	
Interventions	Varenicline 1 mg x 2/day Placebo inactive tablets, same regimen All participants received 6 sessions over 9 weeks of interactive behavioural support in person or by telephone. Treatment period was 12 weeks.	
Outcomes	Primary: 7‐day PPA, CO‐confirmed, at weeks 12 and 24 Secondary: CA and time to relapse	
Notes	New for 2022 update Funding source: "National Institute on Drug Abuse, Penn Center for AIDS Research, Penn Mental Health AIDS Research Center. Pfizer provided medication and placebo free of charge" Declaration of interests: "Dr. Schnoll has provided consultation to Pfizer, GlaxoSmithKline, and Curaleaf. Dr. Gross serves on a Pfizer Data and Safety Monitoring Board for a drug unrelated to smoking or HIV. Dr. Ashare has an investigator‐initiated grant from Novo Nordisk for a drug unrelated to the current study."	
***Risk of bias***		
**Bias**	**Authors' judgement**	**Support for judgement**
Random sequence generation (selection bias)	Low risk	"Eligible participants were randomized 1:1 by a computer‐generated protocol provided by the study statistician to the University of Pennsylvania’s Investigational Drug Service (IDS)"
Allocation concealment (selection bias)	Low risk	Randomisation conducted separately by the University of Pennsylvania’s Investigational Drug Service (IDS), and all other study personnel were blinded. Therefore, concealment was likely adequate.
Blinding (performance bias and detection bias) All outcomes	Low risk	Abstinence was biochemically validated and "All participants and study personnel, aside from IDS [Investigational Drug Service], were blinded from treatment arm allocation throughout the trial."
Incomplete outcome data (attrition bias) All outcomes	Low risk	Attrition rates were similar between study arms.
Selective reporting (reporting bias)	Low risk	Prespecified outcomes from trial registry entry all reported in published results

**Table:** Aubin 2008

***Study characteristics***		
Methods	Country: Belgium, France, Netherlands, UK, USA Setting: 24 research centres Aim: to compare the efficacy of varenicline with nicotine patch, both open‐label Dates conducted: January 2005‐June 2006 Study design: open‐label randomised trial	
Participants	757 healthy adults, recruited from SC clinics or by local advertising, aged 18‐75, weight > 45.5 kg, BMI 15‐38, smoking ≥ 15 CPD. Varenicline arm 378, NRT arm 379. Mean age 42.9, 49.2% men, 93% white. Mean CPD 22.7. Previous use of nicotine patch 47.4%, previous use of bupropion 20%. Mean FTND 5.5. Exclusion criteria: standard pharmacotherapy trial criteria, + participants must not have been in a varenicline trial in previous year, or used NRT in previous 6 months	
Interventions	Varenicline 1 mg x 2/day for 12 weeks, titrated 1st week Nicotine patch (21 mg weeks 2‐6, 14 mg weeks 7‐9, 7 mg weeks 10‐11) All participants received *Clearing the Air* booklet at baseline, and brief counselling (≤ 10 min) at each clinic visit or by phone. TQD was at week 1 visit. Weekly visits throughout treatment phase, plus a phone call 3 days post‐TQD In follow‐up phase, clinic visits at weeks 13, 16, 24, 32, 40, 48 and 52, plus brief phone calls at weeks 14, 20, 28, 36 and 44	
Outcomes	CO‐confirmed CAR for last 4 weeks' treatment (varenicline weeks 9‐12, NRT weeks 8‐11) CO‐confirmed CAR at weeks 9‐24 and 9‐52 (varenicline) and 8‐24 and 8‐52 (NRT) 7‐day PPA at EoT and at weeks 24 and 52 Other outcomes: weight change, withdrawal symptoms (using MNWS and mCEQ), AEs Validation was by expired CO ≤ 10 ppm Dropouts and losses to follow‐up were included in the analyses as continuing smokers (ITT analysis) Attrition in treatment phase was 17.3% varenicline, 20.3% NRT. Losses to follow‐up 17% in each group 65.7% of varenicline and 62.2% of NRT groups completed study	
Notes	New for 2008 update Study funding: study funded by Pfizer Author declaration of interests: not reported	
***Risk of bias***		
**Bias**	**Authors' judgement**	**Support for judgement**
Random sequence generation (selection bias)	Low risk	"Using a central computer‐generated sequence"
Allocation concealment (selection bias)	Low risk	Central allocation
Blinding (performance bias and detection bias) All outcomes	Low risk	"Using an open‐label design". However, comparators were two active treatments, minimising risk of performance bias, and abstinence was biochemically validated
Incomplete outcome data (attrition bias) All outcomes	Low risk	"Missing CO data were assumed to be < 10 ppm provided other conditions were met", i.e. no NRT other than prescribed patches. Missing = negative assumption reduced successes by 1 in each group
Selective reporting (reporting bias)	Low risk	All predicted outcomes fully reported

**Table:** Baker 2016

***Study characteristics***		
Methods	Country: USA Setting: 2 University sites in Wisconsin (Madison, Milwaukee) Aim: to compare the efficacy of varenicline with nicotine patch, and with combination NRT (C‐NRT) Dates conducted: May 2012‐November 2015 Study design: open‐label randomised trial (no placebo)	
Participants	1086 healthy adults, recruited from participants in the ongoing Wisconsin Smokers Health Study or by media and community outreach, aged 17+, smoking ≥ 5 CPD, motivated to quit Varenicline arm 424, nicotine patch arm 241, combination NRT arm 421 Mean age 48.1, 47.9% men, 67% white. Mean CPD 17. Mean FTND 4.8 Exclusion criteria: standard pharmacotherapy trial criteria, CO < 4 ppm, no suicide attempts in previous 5 years, or current suicidal ideation, diagnosis or treatment of psychoses in previous 10 years	
Interventions	Varenicline 1 mg x 2/day for 12 weeks, titrated 1st week Nicotine patch: 11+ CPD on 21 mg weeks 1‐8, 14 mg weeks 9‐10, 7 mg weeks 11‐12; 5‐10 CPD on 14 mg weeks 1‐10, 7 mg weeks 11‐12 Nicotine patch as above, plus nicotine lozenge (2 mg or 4 mg), at least 5 times a day for 12 weeks All participants received counselling (20 min at visits 1, 2 and 3, and 10 min by phone and at visits 4, 5) at 1 week pre‐TQD and at TQD, weeks 1, 4, 12 post‐TQD, plus phone call at week 8 In follow‐up phase, participants were contacted at weeks 26 and 52 by phone	
Outcomes	All comparisons were based on varenicline and C‐NRT versus patch (reference arm), and on varenicline versus C‐NRT CO‐confirmed PPA at week 26 CO‐confirmed PA from day 7 post‐TQD to day 181 CO‐confirmed PPA at weeks 4, 12, 52 Other outcomes: adherence, withdrawals, AEs Validation was by expired CO ≤ 9 ppm and ≤ 5 ppm Dropouts and losses to follow‐up were included in the analyses as continuing smokers (ITT analysis) Withdrawal rates were 8.3% varenicline, 6.2% nicotine patch, 3.1% C‐NRT	
Notes	The trial was funded by grant 5R01HL109031 from National Heart, Lung, and Blood Institute, and by grant K05CA139871 from the National Cancer Insitute New for 2016 update Author declaration of interests: "All authors have completed and submitted the ICMJE Form for Disclosure of Potential Conflicts of Interest. Dr Stein reports receipt of data and safety monitoring board honoraria from Lilly and Abbott. No other disclosures were reported."	
***Risk of bias***		
**Bias**	**Authors' judgement**	**Support for judgement**
Random sequence generation (selection bias)	Low risk	"computer‐based randomisation"
Allocation concealment (selection bias)	High risk	"Treatment assignment was unblinded" (open‐label)
Blinding (performance bias and detection bias) All outcomes	High risk	"Treatment assignment was unblinded" (open‐label) "The follow‐up telephone assessments were intended to be blinded, but a database search by interviewers could have revealed treatment assignment"
Incomplete outcome data (attrition bias) All outcomes	Low risk	Low rates of attrition, ITT analysis used
Selective reporting (reporting bias)	Low risk	All predicted outcomes reported, protocol available

**Table:** Baker 2021

***Study characteristics***		
Methods	Country: USA Setting: 2 research clinics in Wisconsin Aim: to compare combinations of varenicline plus the nicotine or placebo patch vs combinations used for either 12 weeks (standard duration) or 24 weeks (extended duration) Study design: double‐blind, 2 × 2 factorial RCT	
Participants	1251 adult smokers, smoking at least ≥ 5 CPD and motivated to quit. 54% female, mean age 49.1, average CPD at baseline 16	
Interventions	Varenicline monotherapy for 12 weeks. 1 mg x 2/day, titrated for first week Varenicline 1 mg x 2/day for 12 weeks, titrated for first week. Plus nicotine patch 14 mg/day for 12 weeks Varenicline monotherapy for 24 weeks. 1 mg x 2/day, titrated for first week Varenicline 1 mg x 2/day for 24 weeks, titrated for first week. Plus nicotine patch 14 mg/day for 24 weeks All participants received a total of 1.5 h (6 x 15 min over 10 weeks) of interactive behavioural support, either as face‐to‐face consultations or via telephone. Treatment period was either 12 or 24 weeks	
Outcomes	Primary outcome: CO‐confirmed self‐reported 7‐day PPA at 52 weeks Secondary outcome: prolonged CO‐confirmed self‐reported abstinence ‐ from day 7‐week 52 after TQD) Verification: CO ≤ 5 ppm	
Notes	New for 2022 update. Study funding: "Pfizer supplied the study with free active and placebo varenicline as per an investigator‐initiated research agreement. This research was supported by grant 5R01HL109031 from the National Heart, Lung, and Blood Institute and grant K05CA139871 from the National Cancer Institute (both awarded to the University of Wisconsin Center for Tobacco Research and Intervention)." Author declaration of interests: "Dr Baker reported receiving personal fees from the National Cancer Institute for editing a monograph and grants from the National Cancer Institute. Dr Bolt reported receiving grants from the National Institutes of Health. Dr Fiore reported receiving personal fees from the National Cancer Institute. No other disclosures were reported."	
***Risk of bias***		
**Bias**	**Authors' judgement**	**Support for judgement**
Random sequence generation (selection bias)	Low risk	Use of software (SASProcPlanversion9.4) to generate randomisation schedule. Quote: "Participants were randomized to 1 of 2 levels of the 2 experimental factors (medication type and medication duration) via a database that used stratified permuted block randomization."
Allocation concealment (selection bias)	Unclear risk	Allocation process not reported (how interventions (active or placebo pills and patches) were put into the letters and sent to participants)
Blinding (performance bias and detection bias) All outcomes	Low risk	Participants and study personnel blinded to allocation. Abstinence biochemically validated
Incomplete outcome data (attrition bias) All outcomes	Low risk	Loss to follow‐up < 50% overall, loss similar across treatment arms, missing observations were assumed to reflect current smoking
Selective reporting (reporting bias)	Low risk	Outcomes pre‐specified in trial registry all reported in results paper

**Table:** Benli 2017

***Study characteristics***		
Methods	Study design: RCT Country: Turkey Setting: SC clinic Recruitment method: participants applied to the SC clinic directly by calling the Turkish Ministry of Health's 'stop smoking' helpline and making an appointment.	
Participants	An unspecified number of participants were randomised. 405 participants were analysed. 17.5% female; average age 35.2; average age 35.2; average CPD 23; mean FTND 6.3	
Interventions	Bupropion. Provided for 3 months Varenicline. Provided for 3 months Common components: behavioural therapy support with a biopsychosocial approach	
Outcomes	SC: 7‐day PPA at 12 months. Validated by a CO level ≤ 5 ppm	
Notes	Funding source: no funding Author conflicts of interest: the authors declare that they have no competing interests Unclear from study reporting if study is a genuine trial or a follow‐up of treatment in a clinic. However, treatment was randomly allocated, so we have treated this study as a trial.	
***Risk of bias***		
**Bias**	**Authors' judgement**	**Support for judgement**
Random sequence generation (selection bias)	Unclear risk	Quote: “Patients who were to receive the medication were randomly determined by the medication support center in order to provide a constant distribution rate of varenicline and bupropion and so that physicians would not be aware of the medication distribution.” Comment: no further detail is provided.
Allocation concealment (selection bias)	Unclear risk	Quote: “Patients who were to receive the medication were randomly determined by the medication support center in order to provide a constant distribution rate of varenicline and bupropion and so that physicians would not be aware of the medication distribution.” Comment: no further detail is provided.
Blinding (performance bias and detection bias) All outcomes	Unclear risk	Quote: “Patients who were to receive the medication were randomly determined by the medication support center in order to provide a constant distribution rate of varenicline and bupropion and so that physicians would not be aware of the medication distribution” Comment: some attempt appears to have been made to blind physicians to group assignment, however no further detail is given, so it is unclear whether participants and outcome assessors were blinded.
Incomplete outcome data (attrition bias) All outcomes	High risk	Only those followed up at 12 months are included in analysis
Selective reporting (reporting bias)	Unclear risk	No protocol found

**Table:** Bohadana 2020

***Study characteristics***		
Methods	Country: Israel Setting: Pulmonary Institute of Shaare Zedek Medical Center (SZMC), Jerusalem, Israel, in participants' home Aim: to assess whether varenicline preloading for 6 weeks prior to the TQD reduced pre‐quit smoke intake and enhanced 6‐month abstinence outcomes compared with the standard 1‐week preloading. Study design: parallel placebo‐controlled RCT	
Participants	242 daily smokers, smoking ≥ 10 CPD, and had smoked for ≥ 5 pack years, and were motivated to quit. 27% female, mean age 48, average CPD at baseline 25	
Interventions	1 week of varenicline preload: 5 mg of varenicline once daily for 3 days and 0.5 mg twice daily for 4 days (week 1), followed by varenicline 1 mg twice daily for 5 additional weeks 6 weeks of varenicline preload: matched placebo tablets for 5 weeks, followed by a 1‐week titration of varenicline as in group 1 TQD was 6 weeks post‐initiation of treatment.	
Outcomes	Primary: 24‐week biochemically verified CAR from weeks 6–30 Secondary: 23‐week CAR from 1‐week post‐TQD (week 7) to week 30; 7‐day PPA at week 30 Biochemically validated: expired CO ≤ 5 ppm and urine cotinine equivalent concentration ≤ 1 mg/mL	
Notes	New for 2022 update Study funding: "2013 Global Research Award for Nicotine Dependence (GRAND) supported by Pfizer, Inc. (#WI182915)" Author declaration of interests: "None"	
***Risk of bias***		
**Bias**	**Authors' judgement**	**Support for judgement**
Random sequence generation (selection bias)	Unclear risk	"Randomisation was accomplished by extracting a systematic sample via a pre‐prepared list of alternate allocations"
Allocation concealment (selection bias)	Unclear risk	"These procedures were performed by the randomisation monitor (VP) in a secure room where the study medication, provided free of charge by Pfizer Inc., NY, was stored in identically packaged, blinded bottles containing varenicline or placebo. Participants were referred to the secure room where they were randomly allocated (in a 1:1 ratio) to the experimental group."
Blinding (performance bias and detection bias) All outcomes	Low risk	Double‐blind during preloading phase (open‐label afterwards as both groups were receiving identical treatments).
Incomplete outcome data (attrition bias) All outcomes	Low risk	Attrition similar in both groups, overall < 50%
Selective reporting (reporting bias)	Low risk	Outcomes reported as in clinical trials record

**Table:** Bolliger 2011

***Study characteristics***		
Methods	Countries: Brazil, Colombia, Costa Rica, Egypt, Jordan, Lebanon, Mexico, Saudi Arabia, South Africa, United Arab Emirates, Venezuela Setting: 42 research centres (51.2% Latin America, 30.6% Africa, 18.2% Middle East) Aim: to test the efficacy and tolerability of varenicline in regions not previously exposed to SC RCTs of varenicline Dates conducted: April 2008‐August 2009 Study design: double‐blind placebo‐controlled RCT	
Participants	593 adults, recruited from SC clinics, aged 18‐75, weight > 45.5 kg, BMI 15‐38, smoking ≥ 10 CPD, motivated to quit. Randomised to varenicline 394 (390 got medication), or placebo 199 (198 got medication). Mean age 43.5, 63.6% men, mean CPD 23.8, mean FTND 6.0. 55% had no prior quit attempt Exclusion criteria: standard pharmacotherapy trial criteria, + participants must not have used NRT, bupropion, clonidine or nortriptyline in previous 6 months	
Interventions	Varenicline 1 mg x 2/day, titrated during week 1 Placebo inactive tablets, same regimen Treatment period was 12 weeks. All participants received *You can quit smoking* self‐help booklet (available in English, Spanish, Portugese and Arabic) at baseline, and brief counselling (≤ 10 min) at each clinic or telephone contact. TQD set for week 1. Clinic visits at weeks 2, 3, 4, 6, 8, 10 and 12 throughout treatment phase, plus a phone call 3 days post‐TQD In follow‐up phase, clinic visits at weeks 13, 16, 20 and 24, plus brief phone calls at weeks 14, 18 and 22	
Outcomes	Primary outcome: CO‐validated CAR at 9‐12 weeks Secondary outcomes: CO‐validated CAR at 9‐24 weeks; 7‐day PPA at weeks 12 and 24 Other outcomes: AEs, clinically significant changes in vital signs, SAEs Abstinence was assessed using the Nicotine‐Use Inventory (NUI); validation was by expired CO ≤ 10 ppm Dropouts and losses to follow‐up were included in the analyses as continuing smokers (ITT analysis). [4 (V) 1 (P) who did not receive allocated intervention re‐included in denominators in this analysis.] Attrition in treatment phase was 11.2% (V) and 20.6% (P); in follow‐up phase 2.5% (V) and 0.5% (P)	
Notes	New for 2012 update The study was funded and managed by Pfizer Inc Author declaration of interests: "(1) the institutions of Drs. Bolliger, Issa, Posadas‐Valay, and Safwat received financial support from Pfizer for the clinical trial; (2) Drs. Bolliger, Issa, Posadas‐Valay, and Safwat received no financial support from Pfizer for the submitted work; (3) Drs. Bolliger, Issa, Posadas‐Valay, and Safwat have specified relationships with Pfizer that might have an interest in the submitted work in the previous 3 years, including investigator payments, consulting honoraria, and grants; (4) their spouses, partners, and children have no financial relationships that may be relevant to the submitted work; and (5) Drs. Bolliger, Issa, Posadas‐Valay, and Safwat have no nonfinancial interests that may be relevant to the submitted work. Drs. Bolliger, Issa, Posadas‐Valay, and Safwat did not receive financial support with respect to the writing or development of the manuscript. Dr. Abreu, Mr. Correia, Dr. Park, and Mr. Chopra are employees of, and stockholders in, Pfizer."	
***Risk of bias***		
**Bias**	**Authors' judgement**	**Support for judgement**
Random sequence generation (selection bias)	Low risk	"Using a block randomization within each site, eligible participants were randomly assigned in a 2:1 ratio to receive varenicline or placebo"
Allocation concealment (selection bias)	Low risk	"a web‐based or telephone call‐in drug management system directed by the sponsor"
Blinding (performance bias and detection bias) All outcomes	Low risk	"All of the study personnel and participants were blinded to treatment assignment until the end of the nontreatment follow‐up phase"
Incomplete outcome data (attrition bias) All outcomes	Low risk	Dropouts and attrition fully reported
Selective reporting (reporting bias)	Low risk	All predicted and expected outcomes reported

**Table:** Carson‐Chahhoud 2020

***Study characteristics***		
Methods	Country: Australia Setting: respiratory, cardiology, neurology, vascular and general medicine wards of 3 Adelaide (South Australia) hospitals Aim: to evaluate efficacy and safety of varenicline + quitline counselling vs quitline counselling alone in people admitted with smoking‐related acute illnesses Study design: phase II/III open‐label single‐blind RCT Dates conducted: August 2008‐December 2011 Study name: Smoking Termination Opportunity for inPatients (STOP)	
Participants	392 adult smokers, aged 18‐75, smoking 10 CPD+, willing to quit, admitted with acute smoking‐related illnesses; randomised to varenicline + counselling (196) or counselling alone (196) Mean age 53, 32% women, 96% white, mean CPD 25, mean FTND 5.6 Exclusion criteria: standard pharmacotherapy criteria, acute or pre‐existing psychiatric illness, history of psychosis or suicidal ideation, use of varenicline in past 12 months	
Interventions	Varenicline 1.0 mg x 2/d for 12 weeks, including week 1 at titrated dose (described as standard MIMS dosing schedule), + counselling Counselling only Both groups received Quit SA 5A behavioural counselling, i.e. maximum of 8 calls over 3 months. Also booklet *Quit because you can,* + stickers and fridge magnets. Participants had to set a TQD within first 2 weeks Contacts were attempted with all participants at days 3 and 5, weeks 1, 2, 3, 4, 12 (EoT). Additional contacts at weeks 26 and 52	
Outcomes	Primary outcome: self‐reported CAR (< 5 cigarettes in total) (2 weeks‐12 months) Secondary outcomes: CAR at 4, 12 and 26 weeks. 7‐day PPA each week for first 4 weeks; craving; reduced hospital bed utilisation; reduction in healthcare costs CO validation ≤ 10 ppm used only in "a random sub‐set of subjects"	
Notes	Partially funded by the Department of Respiratory Medicine, Queen Elizabeth Hospital, Adelaide, SA New for 2012 update (study ID was Smith 2012; changed for 2015 update) Author declaration of interests: "The authors have read the journal’s policy and have the following potential competing interests: KVCC was paid an honorarium and provided with economy airfares and accommodation by Pfizer Australia to present at the 2019 Smoking Exchange Summit in New South Wales where she spoke about ‘culturalspecific issues in smoking cessation’ and as an invited panellist in a plenary session about ‘a national approach to smoking cessation’. In 2017 she received an honorarium and provided with economy airfares and accommodation to speak about 12‐month results of the STOP trial at the annual Pfizer Australia conference in New South Wales, Hunter Valley. This does not alter our adherence to PLOS ONE policies on sharing data and materials. There are no patents, products in development or marketed products associated with this research to declare."	
***Risk of bias***		
**Bias**	**Authors' judgement**	**Support for judgement**
Random sequence generation (selection bias)	Low risk	"A pre‐defined, central, computer‐generated randomization sequence was used to assign subjects in a 1:1 ratio to either 12 weeks of treatment with varenicline plus Quitline‐counseling or 12 weeks of Quitline‐counseling alone."
Allocation concealment (selection bias)	Low risk	"using opaque, sealed envelopes with consecutive numbers"
Blinding (performance bias and detection bias) All outcomes	High risk	Open‐label design. Attempt at single‐blinding (statistical investigator). "Participants and investigators were not blinded to treatment assignment". "Randomization and allocation concealment were performed by respiratory staff independent of the study". Biochemical validation of abstinence conducted in a random subset of participants via exhaled CO levels ≤ 10 ppm
Incomplete outcome data (attrition bias) All outcomes	Unclear risk	"Missing data from questionnaire (e.g., a question missed when administering follow‐up) were randomly imputed via a computer programme" 84% varenicline completed the study at 52 weeks, vs 82% in the placebo group
Selective reporting (reporting bias)	Unclear risk	Reuslts unavailable for some secondary outcomes prespecified in NCT record, however results may still be forthcoming in future publications

**Table:** Chen 2020

***Study characteristics***		
Methods	Country: USA Aim: to compare the efficacies of cNRT and varenicline with one another and with placebo, as a function of the rs16969968 genotype Dates: May 2015‐August 2019 Study design: genotype‐stratified randomised, double‐blind, placebo‐controlled clinical trial	
Participants	822 current smokers, smoking > 5 CPD, with exhaled CO ≥ 8 ppm. 54.6% female, mean age 46.5, baseline average CPD 17.6	
Interventions	Placebo cNRT (nicotine patch 21 mg/day + nicotine lozenge 2 or 4 mg/day) Varenicline 2 x 1 mg/day Randomisation was stratified by rs16969968 genotype in blocks of 6 patients (2 per treatment per block)	
Outcomes	Primary: 7‐day PPA at week 12 Secondary: 7‐day PPA with CO verification at 6 months; 7‐day PPA at 1 year by self‐report; AEs, and adherence Abstinence was defined as no self‐reported smoking for at least 7 days before the assessment with biochemical verification (CO < 8 ppm) for those self‐reporting abstinence.	
Notes	New for 2022 update Funding by National Institute on Drug Abuse, Siteman Cancer Center and NCI Cancer Center Support Grant. Pfizer supplied the study medication free of charge. Author declaration of interests: "L.‐S.C. received free supply of study medication (varenicline and placebo) for this research project via an investigator‐initiated research agreement (IIR) from Pfizer. This free Pfizer product constitutes the support for this study. Pfizer supports the Principal Investigator to exercise the academic freedom and encourages publication of study results whether or not they are favorable for the Pfizer Product. R.M.C. or a member of his family owns stock in Pfizer Inc. L.J.B. is listed as an inventor on Issued US Patent 8,080,371 “Markers for Addiction” covering the use of certain single nucleotide polymorphisms in determining the diagnosis, prognosis, and treatment of addiction, and served as a consultant for the pharmaceutical company Pfizer Inc. (New York City, NY) in 2008. The spouse of N.L.S. is also listed as an inventor on Issued US Patent 8,080,371 “Markers for Addiction.” All other authors declared no competing interests for this work."	
***Risk of bias***		
**Bias**	**Authors' judgement**	**Support for judgement**
Random sequence generation (selection bias)	Low risk	"SAS Version 9 statistical software will be used to generate the random assignment table stratified by CHRNA5 genotype rs16969968" From protocol
Allocation concealment (selection bias)	Low risk	"The group assignment and genotype will be coded to ensure that the double blind is maintained, and the interface will prevent staff from having access to the participant’s assignment and genotype until after the baseline and post treatment assessments have been completed."
Blinding (performance bias and detection bias) All outcomes	Low risk	Placebo‐controlled and matched contact between groups
Incomplete outcome data (attrition bias) All outcomes	Low risk	Attrition < 50% and similar between arms
Selective reporting (reporting bias)	Low risk	Results for all prespecified outcomes reported

**Table:** Chengappa 2014

***Study characteristics***		
Methods	Country: Pittsburgh, USA Setting: 2 outpatient clinics, Western Psychiatric Institute and Clinic; and Dubois Medical Regional Center, Pennsylvania Aim: to assess the efficacy and safety of varenicline to assist in SC among patients with bipolar disorder who were euthymic and motivated to quit smoking Study design: double‐blind placebo‐controlled RCT Dates conducted: February 2010‐March 2013	
Participants	60 outpatient smokers with DSM‐IV‐diagnosed bipolar disorder, aged 18‐65, stable state or on medication, willing to quit in the next 30 days, 10+ CPD; randomised to varenicline (31) or placebo (29) Mean age 46, 69% women, 66% white, mean CPD 18.1, mean FTND 6.2 Exclusions: bupropion use (for SC); usual pharmacological criteria	
Interventions	Varenicline 1 mg x 2/day, titrated for first week Placebo inactive tablets, same regimen All participants received 15‐min SC counselling at each visit. CO tested and pill counts at each visit. Participants in both groups could reduce the dosage if they wished. TQD was set for week 2 onwards (i.e. full dosage reached) Treatment period was 12 weeks. Weekly pill counts to assess adherence Safety data were reviewed blind monthly by an external independent data safety and monitoring board (DSMB)	
Outcomes	Primary: 7‐day PPA, CO‐verified, at 12 weeks Secondary outcomes: 7‐day PPA at 24 weeks; CA at 12, 24 weeks Validation: CO < 10 ppm	
Notes	New for 2016 update Study funding: "The National Institute of Mental Health (NIMH) of the NIH, under award R21MH087928 (Dr Chengappa), provided the main funding for this study. Pfizer provided drug/placebo and an investigator‐initiated grant, WS‐515343 (Dr Chengappa). These monies channeled through the University of Pittsburgh were used to offset costs of study procedures, participant payments, and a percentage of the time and effort of research staff and faculty salaries." Author declaration of interests: "Dr Turkin has served on the speaker’s bureau of Forest, Sunovion, and Otsuka; and owns shares of Pfizer stock. Dr George has received investigator‐initiated and contract research support from Pfizer, served as a consultant to Novartis, and received honoraria from National Institutes of Health (NIH) and American College of Neuropsychopharmacology. Drs Chengappa, Perkins, Brar, and Levine, and Mss Schlicht and Hetrick and have no financial disclosures with regards to this study."	
***Risk of bias***		
**Bias**	**Authors' judgement**	**Support for judgement**
Random sequence generation (selection bias)	Unclear risk	Method not stated, other than "stratified by gender"
Allocation concealment (selection bias)	Unclear risk	Not stated
Blinding (performance bias and detection bias) All outcomes	Low risk	"The treatment assignment was blinded to participating subjects, raters, investigators and statisticians"
Incomplete outcome data (attrition bias) All outcomes	Unclear risk	24 participants in each group completed treatment phase, and 24 (77%) and 20 (69%) completed full study in varenicline and placebo groups respectively Data were analysed using ITT with LOCF
Selective reporting (reporting bias)	Low risk	Prespecified outcomes from trial registry all reported
Other bias	Unclear risk	8 participants (4 in each arm) were on bupropion for depression; 3/15 varenicline quitters and 1/3 placebo quitters were on long‐term bupropion

**Table:** Cinciripini 2013

***Study characteristics***		
Methods	Country: Houston, TX, USA Setting: University of Texas MD Anderson Cancer Center Aim: to assess the relative efficacy of varenicline and bupropion SR plus intensive counselling on SC and emotional functioning Study design: double‐blind placebo‐controlled RCT Dates conducted: August 2006‐October 2007	
Participants	294 volunteer smokers, aged 18‐65, 5+ CPD, fluent in English, no uncontrolled chronic illness, baseline CO > 6 ppm. Mean age 44, 39% women, 66% white, mean CPD 20, mean FTND 4.5, mean baseline CO 24.5 ppm. Allocated to varenicline (86), bupropion (102) or placebo (106) Exclusions: usual pharma exclusions, current or history of psychotic disorder, moderate or high risk of suicidality, contra‐indications to varenicline or bupropion	
Interventions	Varenicline: 12‐week course (1 mg x 2/day) + non‐active bupropion course (placebo) Bupropion: 12‐week course (150 mg x 2/day) + non‐active varenicline course (placebo) Placebo: 12‐week course (placebo pill x 2/day) Blinded study physician could adjust dosages to reduce side effects if required throughout study All participants got intensive counselling, i.e. 6 x in‐person 30‐min individual counselling sessions and 4 x 15‐min phone calls during treatment phase, based on MI techniques. During follow‐up, each participant got a 15‐min in‐person visit at 3 months and 6 months, and a 15‐min phone call at 4 months	
Outcomes	Primary: PA at EoT Secondary: PA at 3 months post‐quit, 6 months post‐quit; CA at 3 months post‐quit, 6 months post‐quit; 7‐day PPA at EoT, 3 months post‐quit, 6 months post‐quit Validation: CO < 10 ppm. Self‐reported abstainers were asked to send a salivary cotinine sample (< 15 ng/mL) by post	
Notes	New for 2016 update Funding: NIDA grant DA017073, NCI grant P50CA70907; varenicline supplied by Pfizer Author declaration of interests: "Dr Cinciripini served on the scientific advisory board of Pfizer, conducted educational talks sponsored by Pfizer on SC (2006‐2008), and has received grant support from Pfizer."	
***Risk of bias***		
**Bias**	**Authors' judgement**	**Support for judgement**
Random sequence generation (selection bias)	Unclear risk	"Adaptive randomization (minimization) was used to stratify the groups for sex, race/ethnicity, history of depression, and baseline smoking rate."
Allocation concealment (selection bias)	Unclear risk	Not stated
Blinding (performance bias and detection bias) All outcomes	Low risk	Abstinence was biochemically validated
Incomplete outcome data (attrition bias) All outcomes	Low risk	Losses to treatment and follow‐up reported, and key variables with significant differences (FTND, years of education) identified between those who stayed in and those who left. ITT analysis conducted. By 6 months, 21/86 for varenicline (24.4%), 29/102 for bupropion (28.4%) and 30/106 for placebo (28.3%) had been lost to follow‐up
Selective reporting (reporting bias)	Low risk	Abstinence outcomes reported as prespecified in trial registry record
Other bias	Unclear risk	Study began as nortriptyline vs bupropion; 3 months later, 19 people had been recruited to bupropion and 18 to placebo; nortriptyline was replaced as the target treatment by varenicline. The nortriptyline phase group (cohort 1) had 19 days of medication and 3 counselling sessions before TQD, whereas varenicline phase group (cohort 2) had 12 days of medication and 2 counselling sessions before TQD. No differences were found between the 2 cohorts, nor between overall findings and cohort 2 findings. Study authors therefore combined both groups into a single study cohort for analysis.

**Table:** Cinciripini 2018

***Study characteristics***		
Methods	Country: USA Setting: hospital‐based outpatient clinic Aim: to determine if varenicline and bupropion combination treatment would result in higher abstinence rates than varenicline alone Study design: double‐blind, parallel‐group RCT	
Participants	386 adult smokers who were "moderately dependent", and motivated to quit. 41.56% female, mean age 48.97, baseline average CPD 19.66	
Interventions	Varenicline 2 x 1 mg/day (titrated for first week) plus placebo Varenicline 2 x 1 mg/day (titrated for first week) plus bupropion 2 x 150 mg/day (titrated for first 3 days) Placebo Participants were randomised in a 3:1 ratio of active treatment:placebo All smokers received 13 x 15‐min in‐person individual behavioral SC support, and 2 brief supportive telephone calls.	
Outcomes	Primary: PA at the 12‐month follow‐up as a function of treatment, specifically between the 2 active treatment groups Secondary: PA at EoT and the 6‐month follow‐up; all other abstinence definitions; nicotine withdrawal, affect; smoking reinforcement/satisfaction; sleep problems Self‐reported abstinence was validated by exhaled CO < 4 ppm	
Notes	New for 2022 update Study funding: "United States National Institutes of Health (NIH) grant R01DA024709 (PI Cinciripini) and by The University of Texas MD Anderson’s Cancer Center Support Grant CA016672, funded by the National Cancer Institute (NCI)." Author declaration of interests: "Dr. Cinciripini served on the scientific advisory board of Pfizer Pharmaceuticals, conducted educational talks sponsored by Pfizer on SC (2006–2008), and has received grant support and medication support from Pfizer. Dr. Karam‐Hage participated in two multi‐site Pfizer‐funded trials and received varenicline from Pfizer to conduct 4 NIH funded trials. Pfizer (New York, NY) provided the active and matching placebo varenicline capsules. All other authors declare that they have no conflicts of interest."	
***Risk of bias***		
**Bias**	**Authors' judgement**	**Support for judgement**
Random sequence generation (selection bias)	Unclear risk	Group assignment was generated with an algorithm developed and managed by study data managers, whose role was limited to data quality and integrity management.
Allocation concealment (selection bias)	Low risk	Allocation method not reported but likely low risk of selection bias "Participants were enrolled onto the trial by study staff […] Participants, medical and research staff who interacted with participants, and the study investigators were blinded to group assignment"
Blinding (performance bias and detection bias) All outcomes	Low risk	Placebo controlled. "Participants, medical and research staff who interacted with participants, and the study investigators were blinded to group assignment". Cessation was biochemically validated
Incomplete outcome data (attrition bias) All outcomes	Low risk	Complete loss to follow‐up = 28%, similar loss to follow‐up across groups (36%, 29%, 23%)
Selective reporting (reporting bias)	Low risk	Outcomes on Clinicaltrials.gov record are reported

**Table:** Courtney 2021

***Study characteristics***		
Methods	Country: Australia Setting: in the community Aim: to determine if cytisine is non‐inferior to varenicline regarding SC Study design: non‐inferiority, outcome‐blinded, open‐label RCT	
Participants	1452 adult smokers, motivated to quit. 51% female, mean age 43, baseline average CPD 18. Recruited via advertisements and SC telephone line	
Interventions	Cytisine 1.5 mg 6 times daily initially, then gradually reduced over the 25‐day course Varenicline 0.5 mg tablets titrated to 1 mg twice daily No placebo control group. All participants received up to 6 telephone calls providing interactive behavioural support.	
Outcomes	Primary: 6‐month CA verified using a CO breath test at 7‐month follow‐up Secondary: self‐reported 3‐ and 6‐month CA; 7‐day PPA at 4 weeks after baseline and at 4‐month and 7‐month follow‐up; cigarette consumption at each follow‐up	
Notes	New for 2022 update Funding by Australian National Health and Medical Research Council, and the Australian government (under the Substance Misuse Prevention and Service Improvements Fund). Author declaration of interests: "Dr McRobbie reported receiving honoraria from Pfizer for speaking at smoking cessation meetings and attending advisory board meetings. Drs McRobbie and Walker reported previously receiving cytisine from Sopharma for the conduct of a noninferiority trial of cytisine vs nicotine replacement therapy. Dr Tutka reported serving as consultant to Aflofarm, which is a manufacturer of cytisine. Dr Mendelsohn reported receiving funding from Pfizer Australia, GlaxoSmithKline, and Johnson & Johnson Pacific for teaching, consulting, serving on an advisory board, and conference expenses. Dr Kwan reported receiving speaking fees from Pfizer. Dr Walker reported receiving cytisine from Achieve Life Sciences for the conduct of a noninferiority trial of cytisine (Tabex) vs varenicline; receiving investigator‐initiated grants and smoking cessation medication (varenicline) and matching placebo from Pfizer for the conduct of a relapse prevention trial in patients with chronic obstructive pulmonary disease who smoke; and serving as a consultant for and receiving honoraria and travel support for speaking at research meetings from Achieve Life Sciences and Pfizer (manufacturers of smoking cessation medications). Dr Gartner reported receiving grants from the Australian Research Council, Metro South Health Service, Central Queensland Hospital and Health Service, Arthritis Australia, and the HIV Foundation Queensland. Dr Ferguson reported previously serving as a consultant to Pfizer and GlaxoSmithKline Consumer Healthcare on matters relating to smoking cessation and harm minimization; having been a member of a scientific advisory board for Johnson & Johnson; receiving researcher‐initiated project grant funding from Pfizer (through the Grand initiative); and having provided consulting services to JUUL Labs Inc while working as a consultant for Pinney Associates. Dr Zwar reported receiving honoraria from Pfizer and GlaxoSmithKline for advice on smoking cessation education programs and for conference expenses. Dr West reported serving as a consultant to Pfizer, which manufactures varenicline, and receiving grants from Pfizer. Dr Farrell reported receiving unrestricted research funding from Mundipharma, Seqirus, and Indivior. No other disclosures were reported."	
***Risk of bias***		
**Bias**	**Authors' judgement**	**Support for judgement**
Random sequence generation (selection bias)	Low risk	"A data management system (UNICOM Intelligence) located at the social research center was used to assign a unique randomization number to study participants using a pregenerated randomization list embedded in the system"
Allocation concealment (selection bias)	Low risk	"A data management system (UNICOM Intelligence) located at the social research center was used to assign a unique randomization number to study participants using a pregenerated randomization list embedded in the system. Only an independent statistician located at the social research center had access to the pregenerated randomization list. After the baseline computer‐assisted telephone interview, the datamanagement system was used to randomly assign each participant to 1 of the treatment groups in a 1:1 ratio (Figure 1)." The permuted block randomization used unequal block sizes of 12 and 16.
Blinding (performance bias and detection bias) All outcomes	Low risk	Open‐label study; however, both groups received active SC treatment. Abstinence was biochemically validated.
Incomplete outcome data (attrition bias) All outcomes	Low risk	Attrition rates were < 50% and similar between arms.
Selective reporting (reporting bias)	Low risk	All pre‐specified outcomes from protocol reported in results paper

**Table:** Cox 2022

***Study characteristics***		
Methods	Study design: RCT Country: USA Recruitment method: through media and physician referral Double‐blind parallel‐group RCT	
Participants	500 adult African Americans who smoked 5+ CPD, motivated to quit. Baseline average CPD: 12.5. Average age 52. 87% female	
Interventions	Varenicline (standard dose) vs placebo All participants received culturally relevant individualised counselling over 16 weeks	
Outcomes	7‐day PPA at 6 months biochemically validated by saliva cotinine 15 ng/mL	
Notes	Study funding: “This work was funded by the National Institute on Drug Abuse (R01DA035796; Cox). Pfizer Global Pharmaceuticals and Pfizer Global Medical Grants provided study medication. Analytical chemistry was supported in part by P30 DA 012393 from the National Institute on Drug Abuse and S10 RR 026437 from the National Center for Research Resources (Benowitz). Dr Tyndale is supported by the Canada Research Chairs Program (Canada Research Chair in Pharmacogenomics). Dr Ahluwalia is supported by P20GM130414, a National Institutes of Health– funded Centers of Biomedical Research Excellence. […] The funders had no role in the design and conduct of the study; collection, management, analysis, and interpretation of the data; preparation, review, or approval of the manuscript; and decision to submit the manuscript for publication.” Author declaration of interests: “Dr Nollen reported receiving grants from the National Institutes of Health during the conduct of the study and nonfinancial support from Pfizer, which has provided study medication for this and other studies, outside the submitted work. Dr Mayo reported receiving grants from the National Institutes of Health during the conduct of the study. Dr Faseru reported receiving grants from the National Institutes of Health during the conduct of the study. Dr Ellerbeck reported receiving grants from the National Institute on Drug Abuse during the conduct of the study. Dr Tyndale reported consulting for Quinn Emanuel Urquhart & Sullivan and Ethimos and receiving grant support from the National Institutes of Health and Canadian Institutes of Health Research during the conduct of the study for work on other projects. Dr Benowitz reported receiving personal fees from Pfizer for serving on an advisory committee and Achieve Life Sciences for serving on a data and safety monitoring board and being a paid expert witness in litigation against tobacco companies outside the submitted work. Dr Ahluwalia reported receiving personal fees from Respira Technologies as a consultant and equity owner outside the submitted work. No other disclosures were reported”	
***Risk of bias***		
**Bias**	**Authors' judgement**	**Support for judgement**
Random sequence generation (selection bias)	Low risk	"participants were assigned to receive varenicline or placebo in a 3:2 ratio via computergenerated individual random assignment to allow more participants to receive active treatment and concurrently strengthen evaluation of medication adverse events (Figure 1). Randomization was stratified by smoking level (≤10 or >10 cigarettes/d) and sex. A block size of 10 was used to generate the randomization schema."
Allocation concealment (selection bias)	Low risk	"The university pharmacy bottled study medication, per randomization code, labelled with participant ID numbers to ensure that study staff, investigators, and participants were blinded to treatment assignment."
Blinding (performance bias and detection bias) All outcomes	Low risk	Abstinence was biochemically validated.
Incomplete outcome data (attrition bias) All outcomes	Low risk	Attrition rates similar between study arms
Selective reporting (reporting bias)	Low risk	Outcomes pre‐specified in NCT record are reported in results paper

**Table:** De Dios 2012

***Study characteristics***		
Methods	Country: Rhode Island and Massachussetts, USA Setting: Butler Hospital, RI Aim: to assess the relative efficacy of varenicline and NRT on SC in Latino light smokers (< 10 CPD) Study design: feasibility double‐blind placebo‐controlled 3‐arm RCT Dates conducted: April 2010‐July 2010	
Participants	32 Latino volunteer light smokers (≤ 10 CPD), aged 18+, willing to set a quit date. Mean age 42, 53.1% women, mean CPD 7.6, mean FTND 2.9. Allocated to varenicline (10), NRT (11), placebo (11) Exclusions: usual pharmacological conditions, on NRT or smokeless tobacco, history of suicide attempts, chronic or acute psychiatric disorder, employed as a pilot, driver or heavy machinery operator	
Interventions	Varenicline 12‐week treatment course, titrated 1st week NRT 24‐h patch: 12 weeks: 4 weeks @ 14 mg, 8 weeks @ 7 mg Varenicline‐placebo, i.e. identical tablet, same regimen All participants received a 30‐min face‐to‐face "culturally informed" SC behavioural intervention, + a non‐tailored self‐help brochure, all available in both English and Spanish. All participants were compensated for attendance, and could receive travel vouchers if necessary	
Outcomes	Primary: 7‐day PPA at 6 months Secondary: 7‐day PPA at weeks 1, 2, 1 month, 2 months, 3 months, 4 months; adherence Validation: CO < 5 ppm; salivary cotinine (not for the NRT group) > 10 mg/mL AEs not reported in detail, although study reports that "There was no pattern that suggested a higher side‐effect profile for those in the varenicline group"	
Notes	New for 2016 update Funding: NCI grant R01CA0129226‐S1 (De Dios); NIDA grant K24‐DA000512 (Stein); NIDA grant R01‐DA1234, NCI grant K07‐CA95623 (Stanton) Author declaration of interests: "The authors of this article do not have any conflicts of interest to declare."	
***Risk of bias***		
**Bias**	**Authors' judgement**	**Support for judgement**
Random sequence generation (selection bias)	Unclear risk	Not stated
Allocation concealment (selection bias)	Unclear risk	Not stated
Blinding (performance bias and detection bias) All outcomes	Unclear risk	"Study personnel and participants in the two‐pill groups (varenicline and varenicline‐placebo) were blinded to treatment condition. The research pharmacy maintained the study blind." NRT group could not be blinded to treatment; outcome assessment blinding not reported
Incomplete outcome data (attrition bias) All outcomes	Low risk	Losses to follow‐up were fully reported; per protocol and ITT analyses conducted
Selective reporting (reporting bias)	Unclear risk	Protocol unavailable

**Table:** Dogar 2020

***Study characteristics***		
Methods	Country: Bangladesh and Pakistan Setting: 32 tuberculosis treatment centres, and at home Aim: to assess the effectiveness and safety of cytisine as a SC aid in patients with tuberculosis Study design: randomised, double‐blind, placebo‐controlled trial	
Participants	2472 adult daily smokers with pulmonary tuberculosis diagnosed in the previous 4 weeks, and motivated to quit smoking. 24.1% female, mean age 42.5, average CPD at baseline 11	
Interventions	Cytisine (9 mg on day 0, gradually reduced to 1.5 mg by day 25) Placebo Randomisation was 1:1 across trial arms. All participants received interactive in‐person behavioural support, which included a 5‐min session on the day of enrolment, and a 10‐min session on the quit date.	
Outcomes	Primary: CA at 6 months, defined as self‐report (of not having used > 5 cigarettes, bidis, a water pipe, or smokeless tobacco products since the quit date) Abstinence was confirmed biochemically by a breath CO < 10 ppm. Secondary: CA at 12 months; PPA at weeks 5 and 12, and 6 and 12 months; early lapses; clinical tuberculosis score; chest X‐ray grade, sputum smear microscopy; adherence to tuberculosis treatment; tuberculosis treatment outcome; Mood and Physical Symptoms Scale score; Strength of Urges To Smoke scores; time to first use of tobacco product of the day	
Notes	New for 2022 update Funding by European Union Horizon 2020 research and innovation programme. Aflofarm provided the study drug cytisine at no cost. Author declaration of interests: "Dogar 2020: DK reports an unrestricted grant from Pfizer in 2009 for an investigator initiated trial on the effectiveness of practice nurse counselling and varenicline for SC in primary care (Dutch Trial Register NTR3067). EK reports participation in clinical studies by Pfizer, and has received grants from Pfizer, before and during the conduct of the study. KS reports a research grant from Pfizer to study the effects of varenicline on waterpipe SC. All other authors declare no competing interests."	
***Risk of bias***		
**Bias**	**Authors' judgement**	**Support for judgement**
Random sequence generation (selection bias)	Low risk	Block randomisation plus stratification; Randomisation lists were pregenerated by the trial statistician at York Trials Unit (University of York).
Allocation concealment (selection bias)	Low risk	"Randomisation lists were pregenerated by the trial statistician at York Trials Unit (University of York) and held securely at the offices of the study partners (ARK Foundation [Dhaka, Bangladesh] and The Initiative [Islamabad, Pakistan]) for sequential allocation by masked trial coordinating staff."
Blinding (performance bias and detection bias) All outcomes	Low risk	Abstinence was biochemically validated and investigators, clinicians, and patients were masked to treatment allocation.
Incomplete outcome data (attrition bias) All outcomes	Low risk	Attrition rates were very low and similar between study arms.
Selective reporting (reporting bias)	Low risk	Pre‐specified outcomes are reported.

**Table:** EAGLES 2016

***Study characteristics***		
Methods	Countries: Argentina, Australia, Brazil, Bulgaria, Canada, Chile, Denmark, Finland, Germany, Mexico, New Zealand, Russian Federation, Slovakia, South Africa, Spain, USA Setting: multiple research centres Aim: to evaluate the efficacy of varenicline, bupropion SR, nicotine patch and placebo for SC, and to assess how far this is moderated by the presence of psychiatric disorders Dates conducted: November 2011‐January 2015 Study design: phase 4 triple‐dummy, double‐blind placebo‐controlled parallel‐group RCT Study name: EAGLES (Evaluating Global Events in a Global Smoking Cessation Study)	
Participants	Treatment‐seeking adult smokers, aged 18‐75, smoking at least 10 CPD, with exhaled CO > 10 ppm at screening. Participants in the psychiatric disorder cohort had to have a current or lifetime stable psychiatric diagnosis, confirmed by Structured Clinical Interview for DSM‐IV disorders, i.e. no acute exacerbation in the previous 6 months, no changes to treatment for 3 months, not imminently likely to change treatment, and not at risk of self‐harm. Allocation for the phychiatric cohort was balanced across 4 diagnostic group disorders, i.e. mood, anxiety, psychotic, personality 44% men, mean age 46, mean CPD 20.7, mean FTND 5.8 Exclusions: past or current diagnosis of schizophreniform or delusional disorders, all delirium, dementia, and other cognitive disorders, and all substance‐induced disorders (other than nicotine) In the psychiatric disorders group, 70% had primary affective disorders, 19% anxiety disorders, 9.5% psychotic disorders, 0.6% personality disorders, and at least ⅓ were taking psychotropic medications Participants were grouped by the presence (4116) or absence (4028) of a history of psychiatric disorders Psychiatric disorders: varenicline 1032; bupropion 1033, NRT patch 1025, placebo 1026 No psychiatric disorders: varenicline 1005; bupropion 1001, NRT patch 1013, placebo 1009 Safety analyses were conducted in cohorts of 4074 (psychiatric) and 3984 (non‐psychiatric)	
Interventions	Varenicline, 1 mg x 2/day (1 week titrated, then 11 weeks full dose) Bupropion SR, 150 mg x 2/day (titrated for 3 days, then full dose for 11 weeks) Nicotine patch, 21 mg x 7 weeks, 14 mg x 2 weeks, 7 mg x 2 weeks (11 weeks) Triple‐dummy placebo for each arm of the trial (12 weeks) All participants received counselling (up to 10 min) at all contacts, and were encouraged to complete all visits even if treatment was discontinued Participants were monitored at weeks 1‐6, 8, 12, 13, 16, 20, 24; contacts were up to 15 face‐to‐face visits and 11 telephone visits	
Outcomes	At least 1 SAE of anxiety depression, feeling abnormal, or hostility, and/or moderate or severe AE of agitation, aggression, delusions, hallucinations, homicidal ideation, mania, panic paranoia, psychosis, suicidal ideation/behaviour/completed 4‐week abstinence confirmed by CO < 10 ppm at weeks 9‐12, and 15‐week abstinence at weeks 9‐24 In the non‐psychiatric cohort, 78.9% completed treatment, and 78.4% completed the study In the psychiatric cohort, 74.2% completed treatment, and 77.8% completed the study	
Notes	Trial was funded by Pfizer and GlaxoSmithKline Author declaration of interests: "RMA reports receiving grants from Pfizer and Alkermes, and providing consulting and advisory board services to Pfizer, Arena Pharmaceuticals, and Cerecor. RMA’s writing of this manuscript was supported, in part, by National Institute on Alcohol Abuse and Alcoholism grant numbers U01 AA013641 and R01 AA019720; National Institute on Drug Abuse/Veterans Affairs Cooperative Studies numbers 1031 and 1032; and Veterans Affairs Merit Award number NEUA‐003‐08S. NLB reports providing consulting and advisory board services to Pfizer and GlaxoSmithKline, and having been a paid expert witness in litigation against tobacco companies. RW reports receiving grants from Pfizer, Johnson & Johnson, and GlaxoSmithKline, and receiving personal fees for advisory board services from Pfizer and GlaxoSmithKline. RW’s salary is funded by Cancer Research UK. AEE reports receiving grants from Pfizer and Forum Pharmaceuticals, and receiving personal fees for advisory board services from Pfizer and Reckitt Benckiser. AEE’s writing of the manuscript was supported by a National Institute on Drug Abuse Career Award in Patient‐Oriented Research, number K24 DA030443. LSA, TM, DL, and CR are employees and stockholders of Pfizer. JA is an employee of GlaxoSmithKline and stockholder of that company. AK is a PAREXEL employee working on behalf of GlaxoSmithKline. The opinions expressed in this Article are the authors’ own, and do not necessarily reflect the views of their employers."	
***Risk of bias***		
**Bias**	**Authors' judgement**	**Support for judgement**
Random sequence generation (selection bias)	Low risk	"computer‐generated randomisation schedule ... using a block size of 8 (1:1:1:1 ratio) for each of the 20 diagnosis by region combinations"
Allocation concealment (selection bias)	Low risk	"Investigators obtained participant identification numbers via a web‐based or telephone call‐in drug management system"
Blinding (performance bias and detection bias) All outcomes	Low risk	"Study product kit codes did not allow deciphering of randomised treatment or block size. As such, participants, investigators, and research personnel were masked to treatment assignment" "The triple dummy design feature required participants to take study medication as masked tablets dispensed in separate varenicline and bupropion pill bottles each with matching placebo along with either applying active or placebo patches on a daily basis"
Incomplete outcome data (attrition bias) All outcomes	Low risk	All losses fully accounted for; ITT analysis conducted throughout
Selective reporting (reporting bias)	Low risk	All protocol‐reported outcomes were addressed.

**Table:** Ebbert 2015

***Study characteristics***		
Methods	Country: 65 centres in 10 countries: USA (14), Australia (4), Canada (6), Czech Republic (7), Egypt (3), Germany (7), Japan (6), Mexico (4), Taiwan (7), UK (7) Setting: clinics, hospitals, academic research centres Aim: to determine the efficacy and safety of varenicline for increasing smoking abstinence rates through smoking reduction Study design: double‐blind placebo‐controlled multinational RCT Study name: Reduce to Quit Dates conducted: July 2011‐July 2013	
Participants	1510 adult smokers, unwilling to quit abruptly (within the next month), aged 18+, smoking mean 10+ CPD, interested in trying to quit within 3 months. Mean age 44.5, 43.7% women, mean CPD 20.7, mean FTND 5.5. Allocated to varenicline (760) or placebo (750) Exclusions: suicidal behaviour in previous 2 years or history of suicide attempts; major depression, anxiety; diagnosis of psychosis, panic disorder, post‐traumatic stress disorder, schizophrenia	
Interventions	Varenicline 24 weeks, titrated 1st week (12 weeks to quit + 12 weeks post‐quit) Placebo 24 weeks, titrated 1st week (12 weeks to quit + 12 weeks post‐quit) All participants asked to reduce their smoking rate by 50% by week 4, by 75%+ by week 8, and 100% by week 12. Individual 10‐min counselling at each visit (18 face‐to‐face and 10 phone calls), + a copy of *Clearing the air: quit smoking today*	
Outcomes	Primary: CAR at weeks 15‐24 Secondary: CAR at weeks 21‐24, 15‐52, 21‐52; 7‐day PPA at weeks 24, 52 Validation: CO < 10 ppm	
Notes	New for 2016 update Funding: Pfizer Author declaration of interests: "The authors have completed and submitted the ICMJE Form for Disclosure of Potential Conflicts of Interest. Dr Ebbert reports receiving grants from JHP Pharmaceuticals, Orexigen, Pfizer, and the National Institutes of Health; and receiving personal fees from GlaxoSmithKline during the conduct of the study. Dr Hughes reports receiving personal fees from Alere/Free and Clear, Cicatelli, DLA Piper, Dorrffermeyer, Embera, Equinox, GlaxoSmithKline, Healthwise, Nicoventures, Pfizer, Pro Ed, Publicis, Selecta, and nonfinancial support from Swedish Match. Dr West reports receiving grants, personal fees, and nonfinancial support from Pfizer, GlaxoSmithKline, and Johnson & Johnson. His salary is funded by a Centre grant from Cancer Research UK. Dr Rennard reports being an advisory board member for A2B Bio, Almirall, Dalichi Sankyo, Novartis, Nycomed, and Pfizer; consulting for Almirall, APT Pharma/Britnall, AstraZeneca, Boehringer Ingelheim, Chiesi, CSL Behring, Decision Resource, Dunn Group, Easton Associates, Gerson, GlaxoSmithKline, MedImmune, Novartis, Pearl, Roche, Takeda, and Theravance; receiving lecture fees from CME Incite, Forest, Novis, PriMed, and Takeda; receiving grants from AstraZeneca, Boehringer Ingelheim, GlaxoSmithKline, Johnson & Johnson, Novartis, and Otsuka. Drs Russ, McRae, Yu, Dutro, Park, and Ms Treadow are employees and stockholders of Pfizer."	
***Risk of bias***		
**Bias**	**Authors' judgement**	**Support for judgement**
Random sequence generation (selection bias)	Low risk	"Participants were randomized to receive varenicline or placebo for 24 weeks of treatment in a 1:1 ratio using a computer generated block randomization schedule within site"
Allocation concealment (selection bias)	Low risk	"Investigators obtained participant identification numbers and treatment group assignments through a web‐based or telephone call‐in drug management system"
Blinding (performance bias and detection bias) All outcomes	Low risk	"Participants, investigators, and research personnel were blinded to randomization until after the database was locked"
Incomplete outcome data (attrition bias) All outcomes	Low risk	Losses fully reported. ITT analyses conducted for efficacy (760 varenicline, 750 placebo), and treated denominators for safety outcomes (751 varenicline, 742 placebo)
Selective reporting (reporting bias)	Low risk	Prespecified outcomes from trial registry reported in results paper

**Table:** Ebbert 2016

***Study characteristics***		
Methods	Country: USA Setting: community Aim: to evaluate the efficacy of varenicline for smoking abstinence among light smokers Study design: placebo‐controlled RCT	
Participants	93 participants, all light smokers (defined as 5‐10 CPD), and motivated to quit. 60% female, mean age 37, baseline mean CPD 7.7	
Interventions	Varenicline 2 x 1 mg/day Placebo All participants received in‐person interactive behavioural support, consisting of 6 x 10‐min sessions	
Outcomes	Primary: 7‐day PPA rate at week 12. Biochemically verified with exhaled C0 < 8 ppm Secondary: PA	
Notes	Funding and medication provided by Pfizer Author declaration of interests: "JOE reports grants from Pfizer during the conduct of the study and grants from Takeda, the US Department of Defense, and the NIH outside the submitted work. RTH reports grants from Pfizer during the conduct of the study and grants from the NIH outside the submitted work. JTH reports grant from Pfizer during the conduct of the study. ITC and DRS declare no conflicts of interest."	
***Risk of bias***		
**Bias**	**Authors' judgement**	**Support for judgement**
Random sequence generation (selection bias)	Low risk	"A computer‐generated randomization sequence assigned participants in a 1:1 ratio to treatment conditions"
Allocation concealment (selection bias)	Low risk	"Pharmacy personnel dispensed study medication into containers labeled according to study identification numbers. Study participants, investigators, and pharmacy staff were blinded to treatment assignment."
Blinding (performance bias and detection bias) All outcomes	Low risk	"Study participants, investigators, and pharmacy staff were blinded to treatment assignment." Abstinence biochemically validated.
Incomplete outcome data (attrition bias) All outcomes	High risk	Study attrition rates were high and varied significantly between study arms: " Study completion rates were 62% (28/45) in the varenicline group and 42% (20/48) in the placebo group."
Selective reporting (reporting bias)	Unclear risk	All outcomes reported in methods are reported in results. However, trial protocol or registry entry unavailable

**Table:** Fouz‐Roson 2017

***Study characteristics***		
Methods	Country: Spain Setting: Stop‐Smoking Clinic Aim: to test for differences between standard 1‐ and 0.5‐mg doses of varenicline in smoking abstinence, adherence and side effects Study design: open‐label randomised parallel‐group controlled trial with 1‐year follow‐up	
Participants	484 adult smokers aged between 20‐80. 40% female, mean age 50.67 years and a smoking history of 37.5 pack‐years. 60.5% had already made at least one attempt to quit previously. Exclusion criteria were: advanced neoplastic disease, advanced chronic kidney disease, pregnancy, breastfeeding or inclusion in a fertility programme	
Interventions	Lower‐dose varenicline: 0.5 mg/12 h for 8 weeks (titrated for the first week) Higher‐dose varenicline 1 mg/12 h for 8 weeks (titrated for the first week) All participants received 6 sessions of interactive behavioural support, as face‐to‐face cognitive behavioural therapy sessions. Participants also had access to telephone support as needed.	
Outcomes	Primary: continuous self‐reported abstinence during 1 year, verified biochemically with exhaled CO < 6 ppm Secondary: adherence and side effects	
Notes	New for 2022 update Funding research grant awarded by the Fundación Neumosur, within the ‘Asociación de Neumología y Cirugía Torácica del SUR’, partial funding by Grifols Author declaration of interests: "All the authors are grateful for the research grant awarded by the Fundación Neumosur within the ‘Asociación de Neumología y Cirugía Torácica del SUR’, as well as the funding made available to the Respiratory Unit of the Virgen Macarena University Hospital by Grifols. Research Ethics Committee Registry. C.P.‐ C.I. 2217 (promotor code and internal code)."	
***Risk of bias***		
**Bias**	**Authors' judgement**	**Support for judgement**
Random sequence generation (selection bias)	Low risk	"Randomization was conducted by a statistician from our hospital, who generated a list of study group assignments for 460 patients, using computer‐generated blocks of 10, with MAS software version 2.1".
Allocation concealment (selection bias)	Unclear risk	Concealment not reported
Blinding (performance bias and detection bias) All outcomes	Low risk	Study open label, but abstinence was biochemically validated
Incomplete outcome data (attrition bias) All outcomes	Low risk	Attrition rates were very low in both study arms.
Selective reporting (reporting bias)	Unclear risk	No protocol or trial registry entry found

**Table:** Gonzales 2006

***Study characteristics***		
Methods	Country: USA Setting: 19 research centres Aim: to test the efficacy and safety of varenicline for SC Dates conducted: June 2003‐April 2005 Study design: double‐blind placebo‐controlled parallel‐group RCT	
Participants	1025 healthy adult volunteers, recruited through media advertising. Allocated to varenicline (352), bupropion (329) or placebo (344). 54% men, 79% white, mean age 42.4, mean CPD 21, mean FTND score 5.3. No significant differences between groups at baseline Exclusion criteria: standard pharmacotherapy trial criteria, + use of tobacco products other than cigarettes; use of NRT, clonidine, nortriptyline within last month; BMI < 15 or > 38 or weight < 45.5 kg; any prior use of bupropion or varenicline	
Interventions	Varenicline 1 mg x 2/day Bupropion 150 mg x 2/day Placebo inactive tablets, same regimen Treatment period was 12 weeks. All participants received *Clearing the Air* self‐help booklet at baseline, and brief counselling (≤ 10 min) at each clinic visit. Weekly visits throughout treatment phase, plus a phone call 3 days post‐TQD In follow‐up phase, clinic visits at weeks 13, 24, 36, 44 and 52, plus brief phone calls at weeks 16, 20, 28, 32, 40 and 48	
Outcomes	Primary outcome: CO‐validated CAR at 9‐12 weeks Secondary outcomes: CO‐validated CAR at 9‐24 weeks and 9‐52 weeks; 7‐day PPA at weeks 12, 24 and 52 Other outcomes: weight change, withdrawal symptoms (using MNWS, QSU‐brief and mCEQ), AEs Validation was by expired CO ≤ 10 ppm Dropouts and losses to follow‐up were included in the analyses as continuing smokers (ITT analysis) Attrition in treatment phase was 31.5%, losses to follow‐up 16% of treatment completers	
Notes	This trial had the same aims and study design as Jorenby 2006 Study funding: "This study was supported by Pfizer Inc, which provided funding, study drug and placebo, and monitoring" Author declaration of interests: "Dr Gonzales reports having received research contracts from Pfizer, SanofiAventis, GlaxoSmithKline, and Nabi Biopharmaceuticals; consulting fees and honoraria from Pfizer, SanofiAventis, and GlaxoSmithKline; and owning 5 shares of Pfizer stock. Dr Rennard reports having had or currently having a number of relationships with companies who provide product and/or services relevant to outpatient management of chronic obstructive pulmonary disease. These relationships include serving as a consultant for Adams, Almirall, Altana, Array Biopharma, AstraZeneca, Aventis, Biolipox, Centocor, Dey, Critical Therapeutics, GlaxoSmithKline, Johnson & Johnson,Merck, Novartis,Ono Pharma,Otsuka, RJ Reynolds, Roche, Sankyo, Schering‐Plough, Scios, and Wyeth; advising regarding clinical trials for Altana, AstraZeneca, Aventis, Centocor, GlaxoSmithKline, Novartis, Pfizer, and Philip Morris; and speaking at continuing medical education programs and performing funded research both at basic and clinical levels for Altana, AstraZeneca, Boehringer Ingelheim, GlaxoSmithKline, and Novartis. Dr Nides reports having received research grants, consulting fees, and honoraria from Pfizer, SanofiAventis, and GlaxoSmithKline. Dr Oncken reports having received research grants, consulting fees, and honoraria from Pfizer; receiving, at no cost, nicotine replacement and placebo products from GlaxoSmithKline for SC studies; and receiving honoraria from Pri‐Med. Drs Azoulay, Watsky, Gong, Williams, and Reeves and Mr Billing report owning Pfizer stock or having stock options in Pfizer"	
***Risk of bias***		
**Bias**	**Authors' judgement**	**Support for judgement**
Random sequence generation (selection bias)	Low risk	"predefined ... computer‐generated randomization sequence", 1:1:1, using block size of 6, stratified by centre
Allocation concealment (selection bias)	Low risk	Central allocation
Blinding (performance bias and detection bias) All outcomes	Low risk	"Participants and investigators were blinded to drug treatment assignments[, and] ... were not encouraged to guess their treatment assignment"
Incomplete outcome data (attrition bias) All outcomes	Low risk	Considered abstinent if, at next non‐missed visit, they reported no smoking... Missing CO but otherwise OK considered abstinent, except at end of study, where all criteria had to be present
Selective reporting (reporting bias)	Low risk	All expected and predicted outcomes covered

**Table:** Gonzales 2014

***Study characteristics***		
Methods	Country: 37 centres in 8 countries: USA (8), Australia (4), Belgium (4), Canada (4), Czech Republic (4), France (3), Germany (5), UK (5) Setting: clinics, hospitals, academic research centres Aim: to evaluate the efficacy and safety of retreatment with varenicline in smokers who had taken varenicline for ≥ 2 weeks in a previous SC attempt Study design: double‐blind placebo‐controlled multinational RCT Dates conducted: December 2010‐November 2012	
Participants	498 adult smokers (varenicline 251, placebo 247) with previous use of 2+ weeks of varenicline at least 3 months prior to screening, aged 18+, CPD 10+, motivated to quit. Mean age 47.5, 50.4% women, 93% white, mean CPD 20.5, mean FTND 5.5	
Interventions	Varenicline 12 weeks, titrated in 1st week, 1 mg x 2/day Placebo, identical regimen Brief (< 10 min) counselling at each contact. TQD set for week 1 visit. Clinic visits at weeks 1, 2, 3, 4, 6, 8, 9, 10, 11, 12; 13, 16, 24, 32, 40, 48, 52. Brief phone calls at weeks 5, 7, 14, 20, 36, 44. Dosage could be halved if intolerable	
Outcomes	Primary: CAR at weeks 9‐12, 9‐52 Secondary: CAR at weeks 9‐24; 7‐day PPA at weeks 12, 24, 52 Validation: CO < 10 ppm	
Notes	New for 2016 update Funding: Pfizer Author declaration of interests: "D.G. reports grants, and nonfinancial and other support from Pfizer during the conduct of the study; and grants, stock ownership, and nonfinancial and other support from Pfizer, grants from Nabi Biopharmaceuticals, and personal fees and nonfinancial support from GlaxoSmithKline outside the submitted work. P.H. reports grants from Queen Mary University of London during the conduct of the study and grants and personal fees from Pfizer, McNeil, GlaxoSmithKline, and Novartis outside the submitted work. L.P. reports grants from Pfizer outside the submitted work. K.N. reports funding and nonfinancial support from Pfizer during the conduct of the study and personal fees from Pfizer (Belgium) outside the submitted work, for serving as an advisory board member and for lectures. L.‐J.T, T.D.M., and J.T. are employees of Pfizer."	
***Risk of bias***		
**Bias**	**Authors' judgement**	**Support for judgement**
Random sequence generation (selection bias)	Low risk	"Eligible participants were randomly assigned to receive either varenicline or placebo at a 1:1 ratio for 12 weeks of drug treatment using computer‐generated block randomization within each site"
Allocation concealment (selection bias)	Unclear risk	Not stated
Blinding (performance bias and detection bias) All outcomes	Unclear risk	Not stated
Incomplete outcome data (attrition bias) All outcomes	Low risk	Losses to follow‐up fully reported. ITT analyses conducted
Selective reporting (reporting bias)	Low risk	Prespecified outcomes from trial registry reported in results paper

**Table:** Gray 2019

***Study characteristics***		
Methods	Country: USA Setting: university outpatient clinic Aim: to evaluate whether varenicline, when added to brief cessation counselling, is efficacious and safe for SC in adolescents Study design: parallel double‐blind placebo‐controlled RCT	
Participants	157 treatment‐seeking adolescent current smokers, aged 14‐21, and motivated to quit. 40% female, mean age 19.4, baseline average CPD 11.3. Participants must have smoked daily for at least 6 months and had at least one failed attempt at quitting. Exclusion criteria: history of mood or psychotic disorder, suicidality, homicidality, or significant hostility or aggression; substance dependence other than nicotine, unstable medical disorder, pregnancy, breastfeeding, or use of medications with SC efficacy; known hypersensitivity to varenicline	
Interventions	Varenicline 2 x 1 mg/day Placebo Participants were randomised in a 1:1 ratio. All participants received in‐person interactive behavioural support weekly for 12 weeks.	
Outcomes	Primary: 7‐day abstinence at the end of the 12 weeks of treatment. Confirmed with urine‐cotinine level Secondary: weekly abstinence throughout active treatment; abstinence at post‐treatment follow‐up visits; time to first 7‐day abstinence	
Notes	New for 2022 update. Study authors provided additional results data upon request Funding by grants from then NIH, and varenicline and placebo were supplied at no cost by Pfizer. Author declaration of interests: "Dr Gray reported consulting for Pfizer, Inc, and receiving grant support from the National Institutes of Health (NIH). Mr Baker reported receiving grant support from the NIH. Dr McClure reported receiving grant support from the NIH. Dr Tomko reported receiving grant support from the NIH during the conduct of the study and outside the submitted work. Dr Squeglia reported receiving grant support from the NIH. Dr Saladin reported receiving grant support from the NIH. Dr Carpenter reported consulting for Pfizer, Inc, during the conduct of the study. No other disclosures were reported."	
***Risk of bias***		
**Bias**	**Authors' judgement**	**Support for judgement**
Random sequence generation (selection bias)	Unclear risk	Stated randomised, but randomisation method not specified
Allocation concealment (selection bias)	Unclear risk	Concealment not reported
Blinding (performance bias and detection bias) All outcomes	Low risk	Trial described as double blinded and equivalent face to face contact identical between study arms. Abstinence biochemically validated
Incomplete outcome data (attrition bias) All outcomes	Low risk	Attrition rates were similar between study arms
Selective reporting (reporting bias)	Low risk	Pre‐specified outcomes reported

**Table:** Heydari 2012

***Study characteristics***		
Methods	Country: Tehran, Iran Setting: SC clinics in the Tobacco Prevention and Control Research Centre, Shahid Beheshti University of Medical Sciences Aim: to evaluate the effectiveness of varenicline in the Iranian community of tobacco quitters and compare it with other treatment methods Study design: 3‐arm randomised parallel clinical study Dates conducted: 2009‐2010 Analysis: 91 participants per group were required	
Participants	272 treatment‐seeking participants: brief advice (91), NRT (92), varenicline (89). 41.2% women, mean age 42.5 years, mean FTND 5.5	
Interventions	Control group: no pharmacotherapy NRT: 8 weeks of 15 mg NRT patches 8 weeks of 1 mg x 2/day varenicline (titrated 1st week) All participants were managed by the same physician. All received brief (5 min) education and counselling at 4 x weekly sessions. TQD was day 14	
Outcomes	Abstinence at 6 months and 12 months, in person or by phone, verified by expired CO (cut‐off value not given)	
Notes	Funding: Masih Daneshvari Hospital Research Institute, Tehran New for 2016 update Author declaration of interests: not reported	
***Risk of bias***		
**Bias**	**Authors' judgement**	**Support for judgement**
Random sequence generation (selection bias)	Unclear risk	"Smokers who attended the clinic for help in quitting were divided randomly"
Allocation concealment (selection bias)	Unclear risk	Not stated
Blinding (performance bias and detection bias) All outcomes	High risk	Open‐label; blinding of outcome assessors not reported, but abstinence biochemically validated
Incomplete outcome data (attrition bias) All outcomes	Low risk	No attrition: "Participants entered the study of their own accord and none left the study"
Selective reporting (reporting bias)	Low risk	Prespecified outcomes from trial registry reported in results paper

**Table:** Hong 2015

***Study characteristics***		
Methods	Country: China Setting: hospital Study design: parallel RCT	
Participants	300 COPD inpatients/perioperative patients, all smokers. 3% female, mean age 58	
Interventions	5As (Ask, Advise, Assess, Assist, and Arrange) 5As + varenicline All participants received interactive behavioural support, either in person or on the telephone	
Outcomes	Self‐reported CA at 24 weeks	
Notes	Study funding: not reported Author declaration of interests: not reported	
***Risk of bias***		
**Bias**	**Authors' judgement**	**Support for judgement**
Random sequence generation (selection bias)	Unclear risk	Stated randomised but methods not reported
Allocation concealment (selection bias)	Unclear risk	Concealment not reported
Blinding (performance bias and detection bias) All outcomes	High risk	Not placebo‐controlled
Incomplete outcome data (attrition bias) All outcomes	Low risk	Study reported no participants lost to follow‐up
Selective reporting (reporting bias)	Unclear risk	Protocol unavailable

**Table:** Hurt 2018

***Study characteristics***		
Methods	Country: USA Study design: phase II/III randomised quadruple‐blind placebo‐controlled trial Study period: July 2011‐April 2013	
Participants	33 adult alcohol‐dependent smokers	
Interventions	Varenicline 1 mg 2 x day for 12 weeks vs placebo	
Outcomes	PA at 12 weeks (EoT), and at 6 months Abstinence self‐reported and biochemically validated by exhaled CO	
Notes	Study results posted on www.clinicaltrials.gov May 2014 Study funding: "This current study was supported by grant R21 DA30645 to Dr Richard D. Hurt and Pfizer IIR to Dr J. Taylor Hays" Author declaration of interests: "R.T.H. reports research grants from Pfizer and the NIH. J.T.H. reports research grants from Pfizer. J.O.E. reports grants from Pfizer during the conduct of the study and grants from Takeda, the U.S. Department of Defense and the NIH outside the submitted work. R.D.H. reports a research grant from the NIH. All other others have nothing to disclose"	
***Risk of bias***		
**Bias**	**Authors' judgement**	**Support for judgement**
Random sequence generation (selection bias)	Unclear risk	Stated "randomized" but method not reported
Allocation concealment (selection bias)	Unclear risk	Not stated
Blinding (performance bias and detection bias) All outcomes	Low risk	"Pharmacy personnel dispensed study medication into containers labeled with study identification numbers. Study participants, investigators, and pharmacy staff were blinded to treatment assignment." Abstinence biochemically validated
Incomplete outcome data (attrition bias) All outcomes	High risk	High attrition rates: 4/16 varenicline group (1 withdrawal, 3 lost), 12/17 placebo group (7 withdrawals, 5 lost)
Selective reporting (reporting bias)	Low risk	Pre‐specified outcomes from NCT record all reported in results paper

**Table:** Ikonomidis 2017

***Study characteristics***		
Methods	Country: Greece Setting: hospital SC clinic Study design: parallel RCT Recruitment method: approached everyone attending SC clinic	
Participants	188 adults who smoke and were motivated to quit. Average age 50. 45% female. Baseline CPD 30	
Interventions	Nicotine patch + nicotine gum (12 weeks) Varenicline (12 weeks) No behavioural support	
Outcomes	SC at 52 weeks, verified by CO < 10 ppm	
Notes	Study funding: "The study was funded by a grant from the Hellenic Cardiac Society" Author declaration of interests: "The authors declared they do not have anything to disclose regarding conflict of interest with respect to this manuscript."	
***Risk of bias***		
**Bias**	**Authors' judgement**	**Support for judgement**
Random sequence generation (selection bias)	Unclear risk	"One hundred eighty‐eight current smokers were randomized to varenicline or nicotine replacement treatment (NRT) for a 3‐month period" No further information provided
Allocation concealment (selection bias)	Unclear risk	"One hundred eighty‐eight current smokers were randomized to varenicline or nicotine replacement treatment (NRT) for a 3‐month period" No further information provided
Blinding (performance bias and detection bias) All outcomes	Low risk	Both groups received active treatments and SC was biochemically validated.
Incomplete outcome data (attrition bias) All outcomes	Unclear risk	Attrition rates unclear
Selective reporting (reporting bias)	Low risk	No evidence of selective reporting

**Table:** Ioakeimidis 2018

***Study characteristics***		
Methods	Country: Greece Setting: hospital Aim: to compare the SC efficacy and safety of e‐cigarettes vs varenicline among patients who continue to smoke after acute coronary syndrome Study design: parallel RCT	
Participants	54 smokers, smoking ≥ 10 CPD and expressing motivation to quit. All had previous acute coronary syndrome (mean time interval between acute coronary syndrome and study entry 6 ± 2.7 months) Baseline average CPD 12	
Interventions	Varenicline (dose not reported) E‐cigarette (12 mg/mL nicotine) All participants received "low intensity" interactive behavioural support.	
Outcomes	Primary: PPA, defined by self‐report of complete abstinence in the 7 days before the 24‐week clinic visit. Abstinence was not biochemically verified.	
Notes	New for 2022 update Study funding: not reported Author declaration of interests: not reported	
***Risk of bias***		
**Bias**	**Authors' judgement**	**Support for judgement**
Random sequence generation (selection bias)	Unclear risk	No information provided on randomisation methods
Allocation concealment (selection bias)	Unclear risk	No information provided on randomisation methods
Blinding (performance bias and detection bias) All outcomes	Low risk	2 effective treatments provided and equal amounts of contact between arms
Incomplete outcome data (attrition bias) All outcomes	Unclear risk	Not specified
Selective reporting (reporting bias)	Unclear risk	Abstract/poster only so not able to judge
Other bias	High risk	Abstract and poster only. 2 different figures presented for quit rate in e‐cigarettes arm (no difference in those presented in varenicline arm) between abstract and poster. Poster percentage aligns with figure, so using that (16.5%) as opposed to abstract figure (32.5%). Contacted study authors but no reply. Calculated n quit based on percentages but unclear what denominators were; e‐cigarettes calculates back to whole number for e‐cigarettes but not for varenicline

**Table:** Johns 2017a

***Study characteristics***		
Methods	Study design: RCT Country: India Setting: not reported Recruitment method: not reported	
Participants	200 people who smoked with one of the following: previous lung disease, a family history of lung cancer, past cancer treatment, lowered immunity, previous smoking‐related cancers, exposure to certain chemicals and radon gas	
Interventions	Counselling alone Counselling for 3 weeks + varenicline (1 mg twice/day) for 6 weeks Counselling for 6 weeks + varenicline (1 mg twice/day) for 6 weeks	
Outcomes	PPA at 52 weeks, verified by exhaled CO < 10 ppm	
Notes	Study funding: not reported Author declaration of interests: not reported Abstract only	
***Risk of bias***		
**Bias**	**Authors' judgement**	**Support for judgement**
Random sequence generation (selection bias)	Unclear risk	No information provided
Allocation concealment (selection bias)	Unclear risk	No information provided
Blinding (performance bias and detection bias) All outcomes	High risk	Open‐label
Incomplete outcome data (attrition bias) All outcomes	Unclear risk	Attrition rates not reported
Selective reporting (reporting bias)	Unclear risk	Both continuous and PPA collected but only point prevalence reported (abstract only)

**Table:** Johns 2017b

***Study characteristics***		
Methods	Study design: RCT Country: India Setting and recruitment method not specified	
Participants	300 participants randomised	
Interventions	Bupropion, 150 mg twice daily for 12 weeks Varenicline, 1 mg twice daily for 12 weeks Bupropion and varenicline, taken according to schedules above	
Outcomes	SC: CA at 6 months. Validated by CO AEs: period of measurement not detailed	
Notes	Funding source: none specified Author conflicts of interest: none specified Abstract only. Insufficient data to add to MA	
***Risk of bias***		
**Bias**	**Authors' judgement**	**Support for judgement**
Random sequence generation (selection bias)	Unclear risk	States trial was randomised, no further detail given
Allocation concealment (selection bias)	Unclear risk	No relevant information given
Blinding (performance bias and detection bias) All outcomes	Unclear risk	States only that the study was "double‐blind", no further detail given
Incomplete outcome data (attrition bias) All outcomes	Unclear risk	No relevant information given
Selective reporting (reporting bias)	Unclear risk	Protocol unavailable

**Table:** Jorenby 2006

***Study characteristics***		
Methods	Country: USA Setting: 14 research centres Aim: to test the efficacy and safety of varenicline for SC Dates conducted: June 2003‐March 2005 Study design: double‐blind placebo‐controlled RCT	
Participants	1027 healthy adult volunteers. Allocated to varenicline (344), bupropion (342) or placebo (341). 58% men, 84% white, mean age 43.3, mean CPD 22, mean FTND score 5.3. No significant differences between groups at baseline Exclusion criteria: standard pharmacotherapy trial criteria, + use of tobacco products other than cigarettes; use of NRT, clonidine, nortriptyline within last month; BMI < 15 or > 38 or weight < 45.5 kg; any prior use of bupropion or varenicline	
Interventions	Varenicline 1 mg x 2/day Bupropion 150 mg x 2/day Placebo inactive tablets, same regimen Treatment period was 12 weeks. All participants received brief counselling (≤ 10 min) at each clinic visit Weekly visits throughout treatment phase, plus a phone call 3 days post‐TQD In follow‐up phase, clinic visits at weeks 13, 24, 36, 44 and 52, plus brief phone calls at weeks 16, 20, 28, 32, 40 and 48	
Outcomes	Primary outcome: CO‐validated CAR at 9‐12 weeks Secondary outcomes: CO‐validated CAR at 9‐24 weeks and 9‐52 weeks; 7‐day PPA at weeks 12, 24 and 52 Other outcomes: weight change, withdrawal symptoms (using MNWS, QSU‐brief and mCEQ), AEs Validation was by expired CO ≤ 10 ppm Dropouts and losses to follow‐up were included in the analyses as continuing smokers (ITT analysis) Attrition in treatment phase was 29.3%, losses to follow‐up 8% of treatment completers	
Notes	This trial had the same aims and study design as Gonzales 2006. Study funding: "The data reported in this article were derived from a clinical trial sponsored by Pfizer Inc, which provided funding, study drug and placebo, and monitoring." Author declaration of interests: "Dr Jorenby reported receiving research support from Pfizer, Nabi Biopharmaceutical, Sanofi‐Aventis and consulting fees from Nabi Biopharmaceutical. Dr Hays reported receiving a research grant from Pfizer. Dr Rigotti reported receiving research grant funding and consulting fees from GlaxoSmithKline, which markets smoking cessation medications, and Pfizer and Sanofi‐Aventis, which are developing smoking cessation medications. Dr Rigotti also reported receiving consulting fees from Merck, which is developing smoking cessation medications."	
***Risk of bias***		
**Bias**	**Authors' judgement**	**Support for judgement**
Random sequence generation (selection bias)	Low risk	"computer‐generated list"
Allocation concealment (selection bias)	Low risk	"completed centrally ... and sites used an electronic system to assign participants to treatment"
Blinding (performance bias and detection bias) All outcomes	Unclear risk	"in a double‐blind manner"
Incomplete outcome data (attrition bias) All outcomes	Low risk	CA for missed visits: if self‐reported abstinent at next visit, assumed abstinent, except at week 52 visit when all criteria had to be met
Selective reporting (reporting bias)	Low risk	All expected and predicted outcomes covered

**Table:** King 2022

***Study characteristics***		
Methods	Country: USA Setting: 2 outpatient clinics Aim: to determine whether combined treatment with varenicline tartrate and nicotine patch improves CA from cigarette smoking among smokers who drink heavily Study design: double‐blind, placebo‐controlled, superiority RCT	
Participants	122 current smokers participants, all alcohol‐dependent. Eligible participants smoked between 5 and 30 CPD and drank heavily (> 14 drinks per week for men or > 7 drinks per week for women and ≥ 1 heavy drinking day (defined as > 5 drinks per occasion for men or > 4 drinks per occasion for women) per month for the past year) and were motivated to quit. 45.1% female, mean age 44, baseline average CPD 11.8	
Interventions	Nicotine patch + varenicline 2 x 1 mg/day Nicotine patch + placebo Intervention duration was 12 weeks. Participants were randomised in a 1:1 ratio. All participants received 2x interactive behavioural support sessions 2 weeks apart.	
Outcomes	Primary: self‐reported cigarette CA through weeks 9‐12. Abstinence was biochemically confirmed at the week 12 study visit with exhaled CO < 10 ppm. Secondary: frequency of weekly drinking and weekly heavy drinking during the study period	
Notes	New for 2022 update Funding by grant P30 CA14599 from the University of Chicago Medicine Comprehensive Cancer Center, Global Research Award for Nicotine Dependence from Pfizer, Women’s Board of the University of Chicago Cancer Research Foundation. Author declaration of interests: "Dr King reported receiving grants from Pfizer during the conduct of the study and personal fees from the Respiratory Health Association outside the submitted work. Dr de Wit reported being on the board of directors of PharmAla Biotech; being a scientific advisor to Awakn Life Sciences, Gilgamesh Pharmaceuticals, and Schedule I Therapeutics; and receiving research support from the Beckley Foundation outside the submitted work. Dr Grant reported receiving grants from Biohaven Pharmaceuticals, Otsuka Pharmaceutical Company, and Promentis Pharmaceuticals outside the submitted work. No other disclosures were reported."	
***Risk of bias***		
**Bias**	**Authors' judgement**	**Support for judgement**
Random sequence generation (selection bias)	Low risk	"A 1:1 randomization list was generated by the data manager using a random number generator..."
Allocation concealment (selection bias)	Unclear risk	No information provided
Blinding (performance bias and detection bias) All outcomes	Low risk	Placebo‐controlled, matched behavioural support and biochemically validated cessation
Incomplete outcome data (attrition bias) All outcomes	Low risk	Attrition rates low and similar between study arms
Selective reporting (reporting bias)	Low risk	All prespecified outcomes reported either in published results paper or on trial registry website

**Table:** Le Mao 2020

***Study characteristics***		
Methods	Country: France Setting: hospital, home Aim: to demonstrate that, in smoker patients hospitalised for COPD exacerbation, early initiation of varenicline for 12 weeks, combined with an intensive counselling, is associated with a higher CA rate as compared to intensive counselling alone Study design: multicentre, prospective, double‐blind, randomised trial	
Participants	81 smokers hospitalised for COPD exacerbation. Participants must have been hospitalised for at least 24 h, and smoked ≥ 10 CPD during the last year, and were motivated to quit smoking. 39.5% female, mean age 56.8, baseline average CPD 23.1 Exclusion criteria: patients who used concomitant treatment for SC, past history of severe depression requiring therapy drugs within 5 years or/with ≥ 2 episodes of severe depression requiring medication, attempted suicide	
Interventions	Placebo Varenicline 2 x 1mg/day (titrated for the first week) Participants were randomised on a 1:1 ratio. All participants received interactive behavioural support in person (weeks 1, 4, 8, 12, 26, 52) and on the telephone (weeks 2, 18, 34, 42).	
Outcomes	Primary: CA rate at week 52, defined by the rate of participants who presented the all following criteria: SC for weeks 8‐12, exhaled CO level ≤ 10 ppm at each clinic visit. < 6 cigarettes to weeks 12‐52. complete SC during the 7 previous days before week 52 Secondary CA rate at weeks 12 and 26; punctual abstainer rate (PAR) defined as patient’s rate who did not smoke within 7 days prior to follow‐up visit with exhaled CO level of < 10 ppm at weeks 12, 26 and 52; AEs; nicotine substitute consumption	
Notes	New for 2022 update Funding by "Programme Hospitalier de Recherche Clinique", Ministère de la Santé, Pfizer. Author declaration of interests: "All authors have completed and submitted the ICMJE Form for Disclosure of Potential Conflicts of Interest. Dr. Couturaud reports having received research grant support from Pfizer and fees for board memberships or symposia from Bayer and Astra Zeneca and having received travel support from Bayer, Daiichi Sankyo, Leo Pharma, Intermune and Actelion. Dr. Le Mao declares he has no conflict of interest related to this research. Dr. Tromeur declares she has no conflict of interest related to this research. Dr. Paleiron declares he has no conflict of interest related to this research. Dr. Sanchez reports having received research grant support from Bayer, Daiichi Sankyo and Portola Pharmaceuticals, and fees or non‐financial support for consultancy activities from Actelion, GlaxoSmithKline, Boehringer Ingelheim and Chiesi. Dr Gagnadoux declares he has no conflict of interest related to this research. Dr Jouneau reports grants from AIRB, Boehringer Ingelheim, LVL, Novartis and Roche, and personal fees from Actelion, AIRB, AstraZeneca, BMS, Boehringer Ingelheim, Chiesi, GSK, LVL, Mundipharma, Novartis, Pfizer and Roche. Dr A. Magnan reports personal fees and non‐financial support from GlaxoSmithKline, Novartis, Boehringer Ingelheim, AstraZeneca, Stallergnes, ALK, MundiPharma, Teva, Menarini and Meda Pharma, during the past 5 years. Dr Hayem‐Vannimenus declares she has no conflict of interest related to this research. Dr Dansou declares she has no conflict of interest related to this research. Dr Proust reports fees for consulting from Novartis and personal fees or nonfinancial support from AstraZeneca, Boehringer, Chiesi, Mundifarma, Glaxo‐Smith‐Klein, Novartis, Pearl, Portola, Roche, Sanofi, and Teva. Ms Dion declares he has no conflict of interest related to this research. Dr Larhantec declares he has no conflict of interest related to this research. Ms Le Brestec declares she has no conflict of interest related to this research. Dr. Dewitte declares he has no conflict of interest related to this research. Dr. Roche declares he has no conflict of interest related to this research. Dr Leroyer reports having received research grant support from Pfizer and fees for board memberships or symposia from Bayer and Astra Zeneca and having received travel support from Bayer, Daiichi Sankyo, Leo Pharma, Intermune and Actelion. [...] The funding source was not involved in designing or conducting the study, collecting, managing, analysing or interpreting the data, preparing, reviewing or approving the manuscript, or deciding to submit this for publication. An academic steering committee assumed overall responsibility for all these steps. Dr Couturaud takes responsibility for data access and integrity of the data."	
***Risk of bias***		
**Bias**	**Authors' judgement**	**Support for judgement**
Random sequence generation (selection bias)	Low risk	"Investigators obtained patient randomisation numbers and treatment group assignments through a central computerised internet‐based system."
Allocation concealment (selection bias)	Low risk	"Investigators obtained patient randomisation numbers and treatment group assignments through a central computerised internet‐based system."
Blinding (performance bias and detection bias) All outcomes	Low risk	Abstinence biochemically validated
Incomplete outcome data (attrition bias) All outcomes	Low risk	Attrition rates similar between study arms
Selective reporting (reporting bias)	Low risk	Study registered: NCT01694732. Prespecified outcomes reported
Other bias	Unclear risk	"The main limitation of our study is the small sample size due to premature interruption in relation with the premature interruption of pharmaceutical funding (the study was co‐financed by an institutional French grant and pharmaceutical grant)."

**Table:** Lerman 2015

***Study characteristics***		
Methods	Country: USA and Canada Setting: community Aim: to evaluate whether a genetically informed biomarker of nicotine clearance, the nicotine metabolite ratio (NMR; 3′‐hydroxycotinine:cotinine), predicts response to NRT or varenicline for SC Study design: 4‐site NMR‐stratified multicentre, placebo‐controlled RCT	
Participants	1246 smokers, aged 18–65 years old and reported smoking ≥ 10 CPD for ≥ 6 months (verified with exhaled CO > 10 ppm). 44% female, mean age 46, baseline average CPD 18	
Interventions	Placebo pill + placebo patch Nicotine patch + placebo pill ‐ 11 weeks of patches to match the duration of varenicline after the TQD: 21 mg (6 weeks), 14 mg (2 weeks), and 7 mg (3 weeks) Varenicline + placebo patch ‐ 12 weeks (including 1 week before the TQD), 2 x 1 mg/day (titrated for first week) Participants were assigned in a 1:1:1 ratio, by NMR group. All participants received interactive behavioural support. This included 1 in‐person session pre‐quit, and 4 x 15‐min follow‐up telephone calls.	
Outcomes	Primary: 7‐day PPA at EoT (week 11) to estimate the pharmacological effect by NMR group during the medication period. Abstinence was biochemically verified with exhaled CO < 8 ppm. Secondary end points were side‐effects, withdrawal symptoms, and 6‐month and 12‐month quit rates	
Notes	New for 2022 update Funding by National Institute on Drug Abuse, National Cancer Institute, National Human Genome Research institute, National Institute on General Medical sciences, Abramson Cancer Centre at the University of Pennsylvania, Commonwealth of Pennsylvania Department of Health, Canadian Institutes of Health Research, Canada Foundation for Innovation, Ontario Ministry of Research and Innovation. Pfizer Inc. provided varenicline and placebo pills at no cost. Author declaration of interests: "Lerman received study medication and placebo, as well as support for medication packaging, from Pfizer. She has also consulted to Gilead, and has been a paid expert witness in litigation against tobacco companies. Cinciripini served on the scientific advisory board of Pfizer Pharmaceuticals, conducted educational talks sponsored by Pfizer on smoking cessation from 2006‐2008, and has received grant support from Pfizer. Schnoll received medication and placebo free of charge from Pfizer for a different project, and has consulted to Pfizer and GlaxoSmithKline. George has had both investigator‐initiated and industry‐sponsored grants from Pfizer in the past 12 months, and serves on a Data Monitoring Committee for Novartis. Benowitz has served as a consultant to several pharmaceutical companies that market smoking cessation medications and has been a paid expert witness in litigation against tobacco companies. Tyndale has acted as a consultant to pharmaceutical companies, primarily on smoking cessation. The remaining authors report no conflicts of interest."	
***Risk of bias***		
**Bias**	**Authors' judgement**	**Support for judgement**
Random sequence generation (selection bias)	Low risk	"Biostatistician, independent of study investigators, developed the randomisation procedure which was integrated into a centralised data management system. Subjects were randomised to the treatment arms in a 1:1:1 ratio. Randomisation was stratified by baseline NMR status and study site, and blocked in blocks of 12 patients (4/treatment block) to ensure approximate balance"
Allocation concealment (selection bias)	Low risk	Allocation performed using centralised data management system
Blinding (performance bias and detection bias) All outcomes	Low risk	Abstinence biochemically validated
Incomplete outcome data (attrition bias) All outcomes	Low risk	Attrition rates similar between study arms
Selective reporting (reporting bias)	Low risk	Prespecified outcomes reported

**Table:** Littlewood 2017

***Study characteristics***		
Methods	Country: USA Setting: at home with in‐person study visits (unclear setting) Aim: to evaluate the effectiveness of varenicline for SC, and examine the influence of psychological factors on treatment outcome Study design: double‐blind, placebo‐controlled, RCT	
Participants	205 current cigarette smokers interested in quitting, 34% female, mean age 34, baseline average CPD 16	
Interventions	Placebo Varenicline ‐ 2 x 1 mg/day (titrated for first week) Participants were randomised in a 1:1 ratio. Interventions lasted 12 weeks. All participants received in‐person interactive behavioural support, comprising a 30‐min baseline counselling session and a 10‐20‐min follow‐up at weeks 2, 6, and 12.	
Outcomes	CA for the past month, biochemically confirmed with exhaled CO < 6 ppm Average number CPD 7‐day PPA	
Notes	New for 2022 update Funding by National Institute on Drug Abuse (NIDA) 1R01DA025074‐01A2. Author declaration of interests: "The authors declare that they have no conflict of interest."	
***Risk of bias***		
**Bias**	**Authors' judgement**	**Support for judgement**
Random sequence generation (selection bias)	Low risk	Urn‐randomisation used
Allocation concealment (selection bias)	Low risk	"In order to maintain the blind, the pharmacist controlled the pre‐generated urn randomization schedule and packed varenicline tablets in opaque capsules with microcrystalline cellulose, an inert powder commonly used in packaging medications."
Blinding (performance bias and detection bias) All outcomes	Low risk	Participants and staff measuring the outcome were blinded to allocation. Abstinence biochemically validated.
Incomplete outcome data (attrition bias) All outcomes	High risk	52% of participants lost to follow‐up
Selective reporting (reporting bias)	Unclear risk	No trial registration but reports expected outcomes

**Table:** Mercie 2018

***Study characteristics***		
Methods	Country: France Setting: 30 HIV clinics in French hospitals Aim: to assess the efficacy and safety of varenicline with counselling to aid SC in people living with HIV Study design: randomised, parallel, double‐blind, multicentre, placebo‐controlled phase 3 trial	
Participants	248 people living with HIV who had smoked at least 10 CPD for ≥ 1 year. Participants were motivated to stop smoking, were not dependent on another psychoactive substance. Participants with no history of depression or suicide attempt were eligible. 17% female, mean age 45, baseline number of CPD 20	
Interventions	Participants were randomised on a 1:1 ratio. Intervention duration was 12 weeks Placebo Varenicline ‐ 2 x 1 mg/day (titrated for first week) All participants received interactive, in‐person, behavioural support comprising 10‐15 sessions over 1 year	
Outcomes	Primary: proportion of smokers continuously abstinent from week 9‐week 48, biochemically verified by exhaled CO < 10 ppm	
Notes	New for 2022 update Funding by ANRS, Emerging Infectious diseases, Pfizer. Author declaration of interests: "The institution of JR has received funds from Institut National de la Santé et de la Recherche Médicale (Inserm)‐France Recherche Nord et Sud Sida‐hiv hépatites (ANRS). XD has received grant support from Pfizer. J‐MM is a member of scientific advisory boards of Merck laboratories, Gilead, Bristol‐Myers Squibb, ViiV Healthcare, and Janssen and has received grant support from Merk laboratories and Gilead. BS has received honoraria for seminars from Merck laboratories, Gilead, and Janssen and support for the IAS 2014 conference from Merck laboratories. The institution of CF and GC has received grant support from Inserm‐ANRS and Pfizer. GC has received grant support for International Workshop on HIV and Hepatitis Observational Databases from Gilead, Tibotec‐Janssen, Roche, Merck laboratories, Janssen Pharmaceuticals, Boehringer Ingelheim, Bristol‐Myers Squibb, GlaxoSmithKline, ViiV Healthcare, Mylan, Abbvie, and Abbott and grant support for ongoing clinical trials of Inserm‐ANRS from Gilead, Tibotec‐Janssen, Merck laboratories, Boehringer Ingelheim, and Abbott. All other authors declare no competing interests."	
***Risk of bias***		
**Bias**	**Authors' judgement**	**Support for judgement**
Random sequence generation (selection bias)	Low risk	"Randomisation (1:1) was done centrally via electronic case report software (CS software, Ennov‐Clinsight), on the basis of a list generated with SAS software, version 9.2 (PROC PLAN procedure, block size 8)"
Allocation concealment (selection bias)	Low risk	"Only the trial statistician (JA) had access to the randomisation list during the trial."
Blinding (performance bias and detection bias) All outcomes	Low risk	CO ≤ 10 ppm and "Patients and investigators were masked to treatment group allocation."
Incomplete outcome data (attrition bias) All outcomes	Low risk	"71 (58%) participants in the varenicline group versus 81 (65%) in the placebo group completed follow‐up at 48 weeks"
Selective reporting (reporting bias)	Unclear risk	Not all prespecified outcomes reported in results paper. However, authors state "Other objectives described in the protocol will be reported elsewhere".

**Table:** Nahvi 2014a

***Study characteristics***		
Methods	Country: USA Setting: 3 urban outpatient clinics for substance use disorder (SUD) in the Bronx, NY Aim: to test the efficacy and safety of varenicline for SC among opioid‐dependent people on a maintenance regimen Study design: randomised quadruple‐blind controlled trial Dates conducted: August 2009‐September 2011	
Participants	112 smokers in methadone treatment for substance abuse, aged 18+, CPD 5+, motivated to quit within next 6 months. Allocated 57 varenicline, 55 placebo. 52% women, 54% Hispanic, mean CPD 15, mean FTND 4	
Interventions	Varenicline: 12‐week standard regimen, titrated for 1st week Control: identical placebo tablets and regimen All participants set a TQD 1 week after treatment began. All were offered structured, brief (≤ 10 min) individual in‐person counselling by a physician or tobacco specialist at baseline and at 2‐, 4‐, 8‐ and 12‐week visits. All participants were also offered free quitline support	
Outcomes	7‐day PPA at 12 and 24 weeks Validation: expired CO < 8 ppm	
Notes	New for 2016 update Funding: National Center for Research Resources grant UL1 RR025750 to SN, and the National Institute on Drug Abuse grants K23 DA025736 to SN and R25 DA023021 to SN and JHA Author declaration of interests: "The authors have no connection with the tobacco, alcohol, pharmaceutical or gaming industries or anybody substantially funded by one of these organizations. The Albert Einstein College of Medicine Office of Biotechnology and Business Development receives funding from Pfizer, which manufactures varenicline; neither that office nor Pfizer had any role in the study design, analyses or decision to submit the manuscript for publication. None of the authors have direct or indirect funding from Pfizer"	
***Risk of bias***		
**Bias**	**Authors' judgement**	**Support for judgement**
Random sequence generation (selection bias)	Low risk	"Treatment group allocation was computer‐generated, and stratified by the three clinic sites in blocks of six within each stratum"
Allocation concealment (selection bias)	Low risk	"a central data manager concealed the allocation sequence using a password‐protected file, assigned subjects to treatment groups and faxed pre‐printed medication orders to the study pharmacist. The pharmacist prepared the research medication by compounding varenicline tablets or placebo lactose powder to create identical‐appearing capsules. The pharmacist marked medication bottles with subjects’ study identification numbers, and delivered medications for individual study subjects to clinical sites, where they were distributed to each subject by the research assistant"
Blinding (performance bias and detection bias) All outcomes	Low risk	"The pharmacist prepared the research medication by compounding varenicline tablets or placebo lactose powder to create identical‐appearing capsules. The pharmacist marked medication bottles with subjects’ study identification numbers, and delivered medications for individual study subjects to clinical sites, where they were distributed to each subject by the research assistant" "All subjects, research assistants, counsellors and physicians were blinded to treatment assignment"
Incomplete outcome data (attrition bias) All outcomes	Low risk	Losses during treatment (varenicline 6, placebo 9) and during follow‐up (varenicline 2, placebo 3) fully reported; ITT analyses conducted
Selective reporting (reporting bias)	Low risk	Prespecified outcomes from trial registry reported in results paper

**Table:** Nakamura 2007

***Study characteristics***		
Methods	Country: Japan Setting: 19 study sites Aim: to test efficacy, safety and tolerability of 3 doses of varenicline over 12 weeks Dates conducted: not stated Study design: double‐blind, placebo‐controlled, parallel‐group RCT	
Participants	619 healthy Japanese adult volunteers, aged 20‐75, smoking ≥ 10 CPD. Allocated to varenicline 0.25 mg x 2/day (153), 0.5 mg x 2/day (156), 1.0 mg x 2/day (156) or placebo x 2/day (154). 1 participant withdrew before treatment, and is excluded from ITT denominator. 1 road traffic accident death removed from varenicline group at 52 weeks Participants stratified by level of nicotine dependence, measured by Tobacco Dependence Screener scale (≥ 5) and by FTND. 515 (83.3%) classified as nicotine‐dependent Demographic data only supplied for nicotine‐dependent group (515/618): 75% men, mean age 39.8, mean CPD 24, mean FTND score 5.6 Exclusion criteria: standard pharmacotherapy trial criteria, + use of NRT within last 30 days, use of pipe tobacco, snuff, chewing tobacco, cigars within last 30 days and throughout trial	
Interventions	Varenicline 0.25 mg x 2/day Varenicline 0.50 mg x 2/day Varenicline 1.00 mg x 2/day Placebo tablet x 2/day Treatment period 12 weeks, 1st week titrated dosage. All participants received a SC booklet *Clearing the Air* at baseline, + brief counselling (≤ 10 min) at each clinic visit. Weekly visits throughout treatment phase, plus a 5‐min phone call at TQD and +3 days post‐TQD In follow‐up phase, clinic visits at weeks 13, 16, 24, 36, 44 and 52, plus brief phone calls at weeks 20, 28, 32, 40 and 48	
Outcomes	Primary outcome: CO‐validated CAR at 9‐12 weeks Secondary outcomes: CO‐validated CAR at 9‐24 weeks and 9‐52 weeks; 7‐day PPA at weeks 2, 12, 24 and 52 Validation was by expired CO ≤ 10 ppm Other outcomes: withdrawal symptoms (using MNWS, QSU‐brief and mCEQ), AEs Dropouts and losses to follow‐up were included in the analyses as continuing smokers (ITT analysis) Attrition in treatment phase was 6.4%, losses to follow‐up 11.4% of treatment completers (excluding 1 death)	
Notes	Trial was funded by Pfizer Inc New for 2008 update Author declaration of interests: "Dr. Nakamura has received research contracts from Pfizer Japan Inc. (Tokyo, Japan), Novartis Pharma K.K. (Tokyo, Japan), and Sanofi‐Aventis K.K. (Tokyo, Japan), and a research grant from Pfizer Research Foundation (Tokyo, Japan). Dr. Oshima has received research contracts from Pfizer Japan Inc"	
***Risk of bias***		
**Bias**	**Authors' judgement**	**Support for judgement**
Random sequence generation (selection bias)	Low risk	"computer‐generated list of random numbers"
Allocation concealment (selection bias)	Low risk	"randomized to 1 of the 4 treatment groups in a 1:1:1:1 ratio using a central procedure"
Blinding (performance bias and detection bias) All outcomes	Low risk	"double‐blinding of subjects and investigators was maintained throughout the study".
Incomplete outcome data (attrition bias) All outcomes	Unclear risk	No comment on level or handling of missing data
Selective reporting (reporting bias)	High risk	CA rates for all participants reported, but demographics, withdrawal and craving measures, and PPA for nicotine‐dependent group only

**Table:** NCT01162239

***Study characteristics***		
Methods	Country: USA Setting: outpatient medical care Aim: to assess the efficacy of the relapse prevention treatment to other extended treatments, such as health education, brief contact, and varenicline Study design: open‐label parallel 4‐arm RCT	
Participants	271 adult smokers, smoking 5+ CPD, who have all completed 12‐week course of varenicline + counselling 38.9% female, mean age 48.9.	
Interventions	Extended brief contact Extended health education Extended relapse prevention ‐ varenicline Extended relapse prevention ‐ long‐term varenicline ‐ 12 weeks of varenicline treatment at standard dosage of 2 x 0.5 mg/day, plus ongoing access to varenicline for up to 40 additional weeks All participants in all trial arms receive 12 weeks of varenicline treatment at standard dosage of 2 x 0.5 mg/day	
Outcomes	Primary: 7‐day PPA at weeks 12, 24, 52, 64, 104 Biochemically verified with exhaled CO at < 8 ppm Secondary: comparison of combined extended vs brief treatment at weeks 24 and 52	
Notes	New for 2022 update Funding by Pfizer (manufacturer‐funded study), and National Institute on Drug Abuse Author declaration of interests: not reported	
***Risk of bias***		
**Bias**	**Authors' judgement**	**Support for judgement**
Random sequence generation (selection bias)	Unclear risk	No information provided on randomisation method (trial registry only)
Allocation concealment (selection bias)	Unclear risk	No information provided (trial registry only)
Blinding (performance bias and detection bias) All outcomes	High risk	Open‐label (and no placebo used in standard varenicline duration arm)
Incomplete outcome data (attrition bias) All outcomes	Low risk	Loss to follow‐up < 50% and similar between arms
Selective reporting (reporting bias)	Low risk	No evidence of selected reporting. All abstinence outcomes reported as planned

**Table:** Niaura 2008

***Study characteristics***		
Methods	Country: USA Setting: 5 research centres Aim: to test the efficacy and safety of varenicline in smokers allowed to modify their own dosage regimen Dates conducted: December 2001‐June 2003 Study design: double‐blind placebo‐controlled RCT	
Participants	320 healthy adult volunteers, aged 18‐65, smoking ≥ 10 CPD. Allocated to varenicline (160), or placebo (160) 52% men, 91% white, mean age 42, mean CPD 22, mean FTND score 5.4 Exclusion criteria: standard pharmacotherapy trial criteria, + use of NRT within last 3 months	
Interventions	0.5 mg varenicline tablets, from 1‐4 per day, as wished Placebo tablets, from 1‐4 per day, as wished Treatment period 12 weeks, 1st week titrated dosage up to 0.5 mg x 2/day. All participants received a SC booklet *Clearing the Air* at baseline, + brief counselling (≤ 10 min) at each clinic visit. Weekly visits throughout treatment phase In follow‐up phase, clinic visits at weeks 13, 24, and 52 weeks, plus monthly phone calls between visits	
Outcomes	Primary outcome: CAR at 4‐7, 9‐12 and 9‐52 weeks Validation was by expired CO ≤ 10 ppm Secondary outcomes: CO‐confirmed CAR at 9‐24 weeks; CO‐confirmed 7‐day PPA Other outcomes: mean modal dosage; withdrawal symptoms (using MNWS, QSU‐brief and mCEQ), AEs Dropouts and losses to follow‐up were included in the analyses as continuing smokers (ITT analysis) Attrition in treatment phase was 22% in varenicline group and 29% in placebo group; losses to follow‐up by week 52 were 36% from varenicline group and 43% from placebo group	
Notes	The trial was funded by Pfizer Inc New for 2010 update Author declaration of interests: " KEW, KRR, and CBB are employees of Pfizer and have stock or stock options in Pfizer. RN has received consulting fees from Pfizer, GlaxoSmithKline, Sanofi‐Aventis, Merck, Constella, and LLC. DEJ has received consulting fees from Nabi Biopharmaceutical and receives research support from Pfizer, Nabi Biopharmaceutical, and Sanofi‐Aventis. FTL serves on speakers’ bureaus for Pfizer and Merck and is a consultant on an advisory panel with Pfizer. JTH received grant support from Pfizer. JEP received grant support from Merck, DepoMed, Pfizer, Novartis, Takeda, Sanofi‐Aventis, Symbollon, TAP, and GlaxoSmithKline. Editorial support was provided by Ray Beck, Jr, PhD of Envision Pharma and was funded by Pfizer, Inc. "	
***Risk of bias***		
**Bias**	**Authors' judgement**	**Support for judgement**
Random sequence generation (selection bias)	Low risk	"randomly permuted blocks and a pseudo‐random number generator"
Allocation concealment (selection bias)	Low risk	"participants were assigned in a 1:1 ratio to varenicline treatment or placebo in the numerical order that they were accepted to the study"
Blinding (performance bias and detection bias) All outcomes	Unclear risk	"double‐blind" but no further information
Incomplete outcome data (attrition bias) All outcomes	Low risk	Missing data imputed if prior and subsequent abstinence confirmed, otherwise assumed still smoking
Selective reporting (reporting bias)	Low risk	All expected and predicted outcomes covered

**Table:** Nides 2006

***Study characteristics***		
Methods	Country: USA Setting: 7 research centres Aim: to test efficacy, tolerability and safety of 3 doses of varenicline over 6 weeks Dates conducted: February 2000‐January 2003 Study design: phase 2 double‐blind placebo‐controlled RCT	
Participants	638 healthy volunteer smokers, aged 18‐65, smoking at least 10 CPD on average. 48% men, 87% white, average age 42, average CPD 20, mean FTND 5.5. Allocated to varenicline group 1 (128), group 2 (128), group 3 (127), bupropion (128), placebo (127) Exclusion criteria: standard pharmacotherapy trial criteria, + use of bupropion within previous 12 months, use of NRT within past 3 months	
Interventions	Varenicline tartrate 0.3 mg x 1/day for 6 weeks, + 1 week placebo Varenicline tartrate 1.0 mg x 1/day for 6 weeks, + 1 week placebo Varenicline tartrate 1.0 mg x 2/day for 6 weeks, + 1 week placebo Bupropion 150 mg x 2/day (titrated in week 1) for 7 weeks Placebo tablets x 2/day for 7 weeks All groups received self‐help booklet *Clearing the Air* at baseline, + brief (≤ 10 min) counselling at weekly clinic visits throughout treatment phase. At each visit smoking status reported and verified; lab samples taken at screening, baseline and weeks 1, 2, 4, 6 and 7 Follow‐up phase (optional): clinic visits at weeks 12, 24, 52 for brief counselling, smoking status and vital signs. Phone calls every 4 weeks from week 16	
Outcomes	Primary outcome: continuous verified 4‐week abstinence for any part of treatment period Secondary outcomes: CQR weeks 4‐7; CQR from week 4 to weeks 12, 24, and 52 Other outcomes: weight change; reduction of craving and withdrawal using MNWS, QSU‐brief and mCEQ; AEs Validation was by expired CO ≤ 10 ppm Trial report ITT analysis based on numbers treated (N = 626); for consistency our MA used numbers randomised (N = 638). Attrition was 30% during treatment period, 25% of follow‐up consenters lost during follow‐up phase	
Notes	Previous users of bupropion > 12 months before were not excluded, unlike Gonzalez and Jorenby trials; prior use ranged from 13% to 20.6% across groups Denominator in trial report is all treated; we have used all randomised in our MA The trial was funded by Pfizer Inc Author declaration of interests: "Dr Nides has received research grants, consulting fees, and honoraria from Pfizer, Sanofi‐Aventis, and GlaxoSmithKline. Dr Oncken has received research grants, consulting fees, and honoraria from Pfizer; received, at no cost, nicotine replacement and placebo products from GlaxoSmithKline for smoking cessation studies; and received honoraria from Pri‐Med. Dr Gonzales reports having received research contracts from Pfizer, Sanofi‐Aventis, GlaxoSmithKline and Nabi Biopharmaceuticals; consulting fees and honoraria from Pfizer, Sanofi‐Aventis, and GlaxoSmithKline; and owning 5 shares of Pfizer stock. Dr Rennard has had or currently has a number of relationships with companies that provide product and/or services relevant to outpatient management of chronic obstructive pulmonary disease. These relationships include serving as a consultant (Adams, Almirall, Altana, Array Biopharma, AstraZeneca, Aventis, Biolipox, Centocor, Dey, Critical Therapeutics, GlaxoSmithKline, Johnson & Johnson, Merck, Novartis, Ono Pharma, Otsuka, RJ Reynolds, Roche, Sankyo, Schering‐Plough, Scios, and Wyeth); advising regarding clinical trials (Altana, AstraZeneca, Aventis, Centocor, GlaxoSmithKline, Novartis, Pfizer, and Philip Morris); speaking at continuing medical education programs; and performing funded research at both basic and clinical levels (Altana, AstraZeneca, Boehringer Ingelheim, GlaxoSmithKline, and Novartis). He owns no stock in any pharmaceutical companies. Drs Watsky and Reeves and Mr Anziano are employees of Pfizer and own Pfizer stock or have stock options."	
***Risk of bias***		
**Bias**	**Authors' judgement**	**Support for judgement**
Random sequence generation (selection bias)	Low risk	"computer‐generated using a method of randomly permuted blocks and a pseudo‐random number generator"
Allocation concealment (selection bias)	Low risk	"assigned ... medication to subjects in numerical order of acceptance into the study"
Blinding (performance bias and detection bias) All outcomes	Unclear risk	"double‐blind", "to preserve treatment blinding"
Incomplete outcome data (attrition bias) All outcomes	Unclear risk	No information
Selective reporting (reporting bias)	Low risk	All expected and predicted outcomes covered

**Table:** O'Malley 2018

***Study characteristics***		
Methods	Country: USA Setting: home, 2 x outpatient substance abuse treatment and research facilities Aim: to test the efficacy of varenicline with medical management for patients with alcohol use disorder and comorbid smoking seeking alcohol treatment, and to evaluate the secondary effects on smoking abstinence Study design: phase 2, randomised, double‐blind, parallel‐group, placebo‐controlled trial	
Participants	131 adult smokers with alcohol use disorder. Eligible participants met alcohol‐dependence criteria and reported heavy drinking (≥ 5 drinks for men and ≥ 4 drinks for women) ≥ 2 times per week and smoking ≥ 2 times per week. 29.8% female, mean age 42.7, baseline average CPD 11.4 Exclusion criteria: current, clinically significant disease or abnormality; diagnosis of a serious psychiatric illness; current suicidal ideation or lifetime history of suicidal behavior; risk of aggression; current diagnosis of drug dependence; risk of clinically significant alcohol withdrawal; medications in the past 3 months to treat alcohol or tobacco dependence; psychotropic medications in the past month. Women of childbearing age could not be pregnant or nursing and had to be practicing effective contraception.	
Interventions	Placebo Varenicline 2 x 1 mg/day Intervention duration was 16 weeks. Participants did not receive any behavioural support for SC ‐ solely for alcohol use reduction.	
Outcomes	Percentage of heavy drinking days weeks 9‐16 No heavy drinking days weeks 9‐16 Prolonged smoking abstinence weeks 13‐16	
Notes	New for 2022 update Funding by grants from the National Institutes of Health and by the State of Connecticut Department of Mental Health and Addiction Services. Pfizer provided varenicline and placebo pills at no cost. Author declaration of interests: "Dr O’Malley reported having been a consultant or an advisory board member for Alkermes, Amygdala, Arkeo, Cerecor, Mitsubishi Tanabe, Opiant, Pfizer; a member of the American Society of Clinical Psychopharmacology Alcohol Clinical Trials Initiative supported by Abbott, Amygdala, Ethypharm, Lilly, Lundbeck, Otsuka, Pfizer, Arbor Pharmaceuticals, and Indivior; a coinvestigator on studies receiving donated medications from Astra Zeneca, Novartis; a site principal investigator for a multisite trial by Lilly; and a scientific panel member for Hazelden Foundation. Dr Petrakis reported being a consultant to Alkermes. Dr Fucito reported registering with the US Patent and Trademark Office the name and content of a web‐based program to help with sleeping and drinking (ie, Call it a Night). No other disclosures were reported"	
***Risk of bias***		
**Bias**	**Authors' judgement**	**Support for judgement**
Random sequence generation (selection bias)	Low risk	"The randomization list was generated by our study statistician (R.G.) and was implemented through a web‐based system (Endpoint Systems)."
Allocation concealment (selection bias)	Low risk	"The randomization list was generated by our study statistician (R.G.) and was implemented through a web‐based system (Endpoint Systems)."
Blinding (performance bias and detection bias) All outcomes	Low risk	"Participants, treatment providers, and research staff were blind to the assignment throughout the study" and abstinence biochemically validated
Incomplete outcome data (attrition bias) All outcomes	Low risk	"N=94 (71.8%) provided data at the 12 month follow‐up" Similar between arms
Selective reporting (reporting bias)	Low risk	Prespecified outcomes reported

**Table:** Oncken 2006

***Study characteristics***		
Methods	Country: USA Setting: 10 research centres Aim: to evaluate efficacy and safety of 4 varenicline dose regimens Dates conducted: not stated Study design: phase 2 double‐blind placebo‐controlled RCT	
Participants	647 healthy volunteer smokers, aged 18‐65, smoking at least 10 CPD. 49.5% men, 80% white, average CPD 21, mean FTND 5.5. Allocated to group 1 (129), group 2 (130), group 3 (129), group 4 (130) or placebo (129) Exclusion criteria: standard pharmacotherapy trial criteria, + use of NRT or bupropion within last 3 months; use of marijuana or tobacco other than cigarettes with last month	
Interventions	0.5 mg non‐titrated (2/day for 12 weeks) 0.5 mg titrated (week 1 1/day, weeks 2‐12 2/day) 1.0 mg non‐titrated (2/day for 12 weeks) 1.0 mg titrated (0.5 mg 1/day for 3 days, 0.5 mg 2/day for 4 days, 1.0 mg 2/day weeks 2‐12) Placebo tablets 2/d 12 weeks All groups received self‐help booklet at baseline, + brief (≤ 10 min) counselling at weekly clinic visits throughout treatment phase, and phone call 3 days post‐TQD. At each visit smoking status reported and CO verified; vital signs, weight and AEs. Urine, blood tests and ECGs at screening, baseline, weeks 1, 2, 4, 7 and 12 Follow‐up phase: smoking status + CO measured at weeks 13, 24, 52; self‐reported status by phone at weeks 16, 20, 28, 32, 36, 40, 44	
Outcomes	Primary outcome: continuous verified 4‐week abstinence at weeks 4‐7 and 9‐12 Secondary outcomes: continuous verified abstinence at weeks 2‐12 and 9‐52; 7‐day PPA throughout treatment phase and at weeks 12, 24 and 52 Other outcomes: weight change; craving and withdrawal changes using MNWS and mCEQ; AEs Validation was by expired CO ≤ 10 ppm Cessation analyses were ITT (all participants randomised), while tolerability and safety analyses were based only on those known to have used the intervention drug (N = 627). Attrition was 27% during treatment phase, and 22% of follow‐up consenters lost in follow‐up phase	
Notes	For cessation analyses, titrated and non‐titrated results were reported separately and pooled. 24‐week continuous cessation data supplied by study authors The trial was funded by Pfizer Inc Author declaration of interests: "Dr Oncken has received research grants, consulting fees, and honoraria from Pfizer; nicotine replacement and placebo products from GlaxoSmithKline at no cost for smoking cessation studies; and honoraria from Pri‐Med. Dr Gonzales has received research contracts, consulting fees, and honoraria from Pfizer, Sanofi‐Aventis, and GlaxoSmithKline and owns 5 shares of Pfizer stock that he received as a gift from his parents. Dr Rennard has had or currently has a number of relationships with companies who provide products and/or services relevant to outpatient management of chronic obstructive pulmonary disease. These relationships include serving as a consultant (for Adams, Almirall, Altana, Array Biopharma, AstraZeneca, Aventis, Biolipox, Centocor, Dey, Critical Therapeutics, GlaxoSmithKline, Johnson & Johnson, Merck, Novartis, Ono Pharma, Otsuka, RJ Reynolds, Roche, Sankyo, Schering‐Plough, Scios, and Wyeth), advising regarding clinical trials (Altana, AstraZeneca, Aventis, Centocor, GlaxoSmithKline, Novartis, Pfizer, and Philip Morris), speaking at continuing medical education programs and performing funded research at both basic and clinical levels (Altana, AstraZeneca, Boehringer Ingelheim, GlaxoSmithKline, and Novartis). He does not own any stock in any pharmaceutical companies. Dr Nides has received research grants, consulting fees, and honoraria from Pfizer, Sanofi‐Avenits, and GlaxoSmithKline. Drs Watsky and Reeves and Messrs Billing and Anziano are employees of Pfizer and own Pfizer stock or hold Pfizer stock options."	
***Risk of bias***		
**Bias**	**Authors' judgement**	**Support for judgement**
Random sequence generation (selection bias)	Unclear risk	Not stated
Allocation concealment (selection bias)	Unclear risk	"Eligible subjects were randomly assigned to 1 of 5 groups"
Blinding (performance bias and detection bias) All outcomes	Low risk	"Subjects and investigators were blinded to the study drug treatment [, and] were not encouraged to guess their treatment assignment"
Incomplete outcome data (attrition bias) All outcomes	Low risk	Missing COs or visits OK if confirmed abstinent before and after missed measure
Selective reporting (reporting bias)	Low risk	All expected and predicted outcomes covered

**Table:** Pastorino 2022

***Study characteristics***		
Methods	Study design: RCT Country: Italy Setting: community Recruitment method: volunteers were recruited through press releases, social networks, television, and other media.	
Participants	869 participants in a lung‐screening trial who smoked. 44% female. Median age 60. Heavy smokers	
Interventions	Behavioural support only Behavioural support plus 40 days of cytisine (standard dose) Behavioural support plus 84 days of cytisine (standard dose)	
Outcomes	CA at 52 weeks ‐ verified by CO ≤ 9 ppm	
Notes	Study funding: “Funded by the Scientific Directorate Fondazione IRCCS Istituto Nazionale Tumori, Italian Association for Cancer Research AIRC (AIRC 5x1000 ID 12162, extension 2017‐2020), Investigation Grant from the Foundation AIRC for the Research on Cancer (AIRC IG 2019, ID 23244), and Ricerca Corrente of the Italian Ministry of Health. The work of SG is partially supported by an Investigation Grant from the Foundation AIRC for the Research on Cancer (AIRC IG 2021, ID 25987) and by the Italian League Against Cancer (LILT, Milan). The fundings had no role in the design of the study; collection, analysis, and interpretation of data; writing of the manuscript, or the decision concerning submission.” Author declaration of interests: "We declare no competing interests." Study authors provided additional results data upon request.	
***Risk of bias***		
**Bias**	**Authors' judgement**	**Support for judgement**
Random sequence generation (selection bias)	Unclear risk	Stated randomised but method not specified
Allocation concealment (selection bias)	Unclear risk	Not reported
Blinding (performance bias and detection bias) All outcomes	High risk	A placebo was not used in the arm that did not receive cytisine.
Incomplete outcome data (attrition bias) All outcomes	Unclear risk	Attrition rates under 50% however data are not available on how many were followed up in the cytisine arms when they were split by length of treatment ‐ thus we can not see if differential attrition occurred
Selective reporting (reporting bias)	Low risk	Prespecified smoking‐related outcomes reported

**Table:** Qin 2021

***Study characteristics***		
Methods	Study design: RCT Country: China Setting: China–Japan Friendship hospital in Beijing Recruitment method: via a trial site, a hotline of SC, advertisements in the community from February 2019‐June 2020	
Participants	136 smokers diagnosed with COPD randomised; 2.9% female, average age 62; average CPD 19.21; FTND mode 0‐3	
Interventions	Varenicline, 2 mg/day for 12 weeks Bupropion, 150 mg/day for 12 weeks Common components, quote: "Participants received a counseling session for more than 60 min when they began medication at week 0, and they also received up to 10 min of counseling at weeks 1, 2, 4, 6, 9, 12, and 24."	
Outcomes	SC: abstinence at 24 weeks (no further details given). Validated by CO ≤ 10 ppm	
Notes	Study funding: "This study was supported by the Capital Health Development Research Project in China (Grant No. 2018‐2‐4066), the National Natural Science Foundation of China (Grant No. 81720108001) and the National Key R&D Program of China (Grant No. 2017YFC1309400)." Author declaration of interests: "The authors report no conflicts of interest in this work."	
***Risk of bias***		
**Bias**	**Authors' judgement**	**Support for judgement**
Random sequence generation (selection bias)	Low risk	Quote: "A biostatistician, independent of the study used Proc Plan in SAS version 9.4 (SAS Institute) to generate a table of random digit to randomly assign the numbers to the two groups. (the number of the random seed is 87,654,321)."
Allocation concealment (selection bias)	Low risk	Quote: "To ensure random concealment, the group information assigned to each participant was put in a sealed, and opaque envelope."
Blinding (performance bias and detection bias) All outcomes	Low risk	Quote: "Because of the different medication packaging, only statisticians were blinded to medication allocation however, all participants received an active evidence‐based smoking cessation pharmacotherapy."
Incomplete outcome data (attrition bias) All outcomes	Low risk	Quote: "Only 8/68 and 7/68 lost in the varenicline and bupropion arms respectively."
Selective reporting (reporting bias)	Low risk	Prespecified outcomes from trial registry reported

**Table:** Rennard 2012

***Study characteristics***		
Methods	Countries: Argentina, Brazil, Canada, China, Czech Republic, France, Germany, Hungary, Italy, Korea, Mexico, Taiwan, UK, USA Setting: 33 research centres Aim: to evaluate efficacy and safety of varenicline allowing a self‐selected quit date Dates conducted: September 2008‐December 2009 Study design: double‐blind placebo‐controlled RCT	
Participants	659 healthy volunteer smokers, aged 18‐75, motivated to quit, smoking at least 10 CPD. 60% men, mean age 43, 68% white, mean CPD 21, mean FTND 5.5, 66% had tried to quit at least once before. Allocated to varenicline (493) or placebo (166) Exclusion criteria: standard pharmacotherapy trial criteria, + use of NRT, bupropion, clonidine or nortriptyline within last 3 months, ever used varenicline; use of marijuana or tobacco other than cigarettes with last month	
Interventions	Varenicline 1 mg x 2/day, titrated in 1st week Placebo inactive tablets, same regimen Participants could choose their own quit date between days 8 and 35 Treatment period was 12 weeks. All participants received *Clearing the Air: Quit smoking today* booklet at baseline, + brief counselling (≤ 10 min) at each clinic visit. Weekly visits throughout treatment phase, and in follow‐up phase clinic visits at weeks 13, 16, 20 and 24. Phone calls at weeks 14, 18 and 22	
Outcomes	Primary outcome: CO‐validated CAR at 9‐12 weeks Secondary outcomes: CO‐validated CAR at 9‐24 weeks; 7‐day PPA at weeks 12 and 24 Other outcomes: AEs, SAEs; timing and number of quit attempts Validation was by expired CO ≤ 10 ppm Dropouts and losses to follow‐up were included in the analyses as continuing smokers (ITT analysis) Attrition to end of study (24 weeks) was 12.4% from varenicline, 20.5% from placebo	
Notes	New for 2012 update Additional information supplied by the study authors The study was funded and managed by Pfizer Inc Author declaration of interests: "SR, JH, PC, EK, and TR did not receive any financial support with respect to the writing or development of this manuscript. Funding from Pfizer Inc. has been received by the authors or their institutions for the following in the 36 months prior to submission: research grants (JH, PC, EK, and TR); advisory board membership (SR and JH); reimbursement for travel and accommodation expenses (SR, PC, EK, and TR); lectures/speaker bureau (PC); consultancy (EK); and development of educational materials (EK and TR). CA, LSA, and CR are employees of Pfizer Inc. JH has received consulting fees from several non‐profit and for‐profit entities that develop or promote smoking cessation products or that advocate for tobacco control measures."	
***Risk of bias***		
**Bias**	**Authors' judgement**	**Support for judgement**
Random sequence generation (selection bias)	Low risk	"a predefined, central, computer‐generated randomization sequence...assigned subjects in a 3:1 ratio". Block size: 4, stratified by centre
Allocation concealment (selection bias)	Low risk	See above
Blinding (performance bias and detection bias) All outcomes	Low risk	Triple‐blind (participant, care‐giver, investigator)
Incomplete outcome data (attrition bias) All outcomes	Low risk	Dropouts and attrition rates fully reported
Selective reporting (reporting bias)	Low risk	All predicted and expected outcomes reported

**Table:** Rigotti 2010

***Study characteristics***		
Methods	Country: 15 countries in Europe, Asia, Americas Setting: 39 research centres Aim: to evaluate efficacy and safety of varenicline in patients with stable CVD Dates conducted: February 2006‐August 2008 Study design: phase 3 double‐blind placebo‐controlled RCT	
Participants	714 adult smokers, aged 35‐75, smoking at least 10 CPD, with stable CVD and motivated to quit. 79% men, 80% white, mean CPD 22, mean FTND 5.6. Allocated to varenicline (355) or placebo (359), stratified by site Exclusion criteria: standard pharmacotherapy trial criteria, + use of NRT or bupropion within previous month. All had been diagnosed for at least 2 months with CVD, but not hypertension alone	
Interventions	Varenicline 1.0 mg 2/day for 12 weeks, including week 1 at titrated dose Placebo tablets as above Both groups received brief (≤ 10 min) counselling at weekly clinic visits throughout treatment phase, and phone call 3 days post‐TQD. At each visit smoking status reported and CO verified; vital signs, weight and AEs. Urine, blood tests and ECGs at screening, baseline, weeks 12 and 52 Follow‐up phase: smoking status + CO measured at weeks 13, 16, 24, 32, 40 and 52; counselling and self‐reported status by phone at weeks 14, 20, 28, 36 and 44	
Outcomes	Primary outcome: CO‐validated CAR at weeks 9‐12 Secondary outcomes: CO‐validated CAR at weeks 9‐52 and 9‐24; 7‐day PPA at weeks 12, 24 and 52 Other outcomes: AEs; SAEs; cardiovascular events; changes in blood pressure and heart rate Validation was by expired CO ≤ 10 ppm Cessation analyses were ITT (all participants randomised minus deaths), while tolerability and safety analyses were based only on those known to have used the intervention drug (N = 703). Attrition was 17.5% from the varenicline group and 20.3% from the placebo group during treatment phase, and 14.9% varenicline and 19.5% placebo who did not complete the study. This includes 2 deaths in the varenicline group and 5 in the placebo group by 52‐week follow‐up	
Notes	New for 2010 update Study funding: "This study was funded by Pfizer Inc. Editorial support for the development of this manuscript was provided by Alexandra Bruce, PhD, of UBC Scientific Solutions and was funded by Pfizer Inc." Author declaration of interests: "Drs Rigotti, Pipe, Benowitz, and Tonstad have consulted for Pfizer. Dr Rigotti has been the site principal investigator for clinical trials of smoking cessation medications funded by Pfizer, sanofi‐aventis, and Nabi Biopharmaceuticals. Dr Pipe has received educational and research support in the past from Bristol‐ Myers Squibb, Johnson & Johnson, GlaxoSmithKline, and Merrell‐Dow. Drs Benowitz and Tonstad served on the scientific planning committee for this study and have been paid consultants to Pfizer and other pharmaceutical companies that are developing and/or marketing smoking cessation medications. Dr Benowitz has been a paid expert witness in litigation against tobacco companies. At the time of the study, his family owned a small amount of Pfizer stock, but no longer does. Dr Tonstad has been the site principal investigator for clinical trials of smoking cessation medication and other medications funded by Pfizer and other pharmaceutical companies. Dr Arteaga is a statistical director at Pfizer Inc, supporting the varenicline studies. Dr Garza is a senior medical director of clinical research and development at Pfizer Inc, and the medical monitor for this study. The other authors report no conflicts."	
***Risk of bias***		
**Bias**	**Authors' judgement**	**Support for judgement**
Random sequence generation (selection bias)	Low risk	"The study sponsor conducted the randomization centrally using a computer‐generated list that prespecified the order of treatment allocation"
Allocation concealment (selection bias)	Low risk	See above
Blinding (performance bias and detection bias) All outcomes	Low risk	Described as "double‐blind" (participants and study implementation). Cardiovascular outcomes "were reviewed separately and adjudicated under blinded conditions by an independent event committee made up of 3 board‐certified cardiologists"
Incomplete outcome data (attrition bias) All outcomes	Low risk	ITT analyses conducted; participants who missed a visit but had validated abstinence at next visit were considered continuously abstinent. But 52‐week status had to be attended and confirmed
Selective reporting (reporting bias)	Low risk	All expected and predicted outcomes covered

**Table:** Rohsenow 2017

***Study characteristics***		
Methods	Country: USA Setting: university offices in Rhode Island, USA Aim: to compare varenicline with transdermal nicotine (NRT) for smokers with current substance use disorders (SUD) for effects on smoking abstinence Study design: double‐blind double‐placebo‐controlled randomised design, stratifying by MDD history	
Participants	137 adult smokers in substance‐use disorder treatment, who were substance abstinent < 12 months, and smoked 10+ CPD for the past 6 months. 47% female, mean age 39.6, baseline CPD 19.5 Participants did not need to motivated to quit Exclusion criteria: hallucinations/delusions, current SC treatment, contraindications for either medication, using medications affected by SC (antipsychotics, warfarin, theophylline and insulin), suicidal ideation, not willing to try to quit smoking and, substance use reported on the day of or before recruitment or positive breath alcohol at recruitment	
Interventions	Varenicline: 12 weeks of 2 x 1 mg/day (titrated for first week) NRT: 21 mg/day, decreasing to 7 mg/day	
Outcomes	Primary: 7‐day smoking PPA at 3 months Secondary: 7‐day PPA at 6 months; quantity and frequency of smoking and substance use at 3 and 6 months; within‐treatment abstinence; medication adherence; depressive symptoms	
Notes	New for 2022 update Funding by a personal grant 1R01DA024652 from the National Institute on Drug Abuse and Department of Veterans Affairs Author declaration of interests: "All authors report no financial interests or potential conflicts of interest except for the following: R.M.S. has received travel and honorarium from D&A Pharma and has received consultant fees from CT Laboratorie"	
***Risk of bias***		
**Bias**	**Authors' judgement**	**Support for judgement**
Random sequence generation (selection bias)	Low risk	"Urn randomization [32] to medication condition (1: 1 assignment) was stratified by gender, median score on the Fagerström Test for Nicotine Dependence [33] from a previous study [28] and history of MDD. The randomization program was run by the Project Manager, who concealed the assignment from all other staff."
Allocation concealment (selection bias)	Low risk	"Urn randomization [32] to medication condition (1: 1 assignment) was stratified by gender, median score on the Fagerström Test for Nicotine Dependence [33] from a previous study [28] and history of MDD. The randomization program was run by the Project Manager, who concealed the assignment from all other staff."
Blinding (performance bias and detection bias) All outcomes	Low risk	Double‐blind trial with placebo patch and placebo tablets used (double placebo). Abstinence biochemically validated
Incomplete outcome data (attrition bias) All outcomes	Low risk	23/60 and 25/77 did not complete follow‐ups for primary outcome at 3 months but information not stated for 6 months; however multiple imputation sensitivity analysis has been done (supplementary materials) which the study authors claim did not change the conclusion
Selective reporting (reporting bias)	Low risk	Prespecified outcomes from trial registry reported

**Table:** Rose 2013

***Study characteristics***		
Methods	Country: USA Setting: Duke University Medical Center, Durham, NC Aim: "Given the safety and tolerability profile of nicotine replacement therapy, our rationale in this study was to use nicotine replacement therapy as an initial line of treatment, and then identify early on which smokers are unlikely to benefit from nicotine alone" Study design: randomised double‐blind parallel‐arm adaptive treatment trial in 2 phases Dates conducted: not stated	
Participants	606 adult smokers, motivated to quit, aged 18‐65, mean CPD 10+ for 3 years, expired CO level 10+ ppm. 46% women, 63% white, mean CPD 21.7, mean FTND 5.8. Participants could receive up to USD 320 for study participation	
Interventions	2‐phase study All participants seen weekly for 2 weeks before TQD, and attended 4‐6 sessions after the TQD. At each session, participant received brief (< 15 min) support, + clinical trial materials. Smoking diaries, expired CO, withdrawal symptoms and reports of AEs were collected each time. Participants were recontacted at 6 months, and those reporting abstinence were invited to return to give a CO sample All participants were given open‐label active NRT patch, either 42 mg/day (baseline CO > 30 ppm) or 21 mg/day (baseline CO < 30 ppm) for 2 weeks; dose reductions allowed if side effects dictated. At 1 week, participants were classified as 'responders' (reduced ad lib smoking by > 50%, CO‐verified) or 'non‐responders' (< 50%) Phase 1 (12 weeks) Non‐responders only (N = 371‐36 who withdrew, = 335) allocated to: double‐blind varenicline, stopping NRT (N = 112) double‐blind augmentation of NRT with bupropion (N = 109) continuation on open‐label NRT alone (N = 114) All participants received dummy (placebo) versions of the other 2 treatments as well as their own active treatment. Phase 2 235 responders after week 1 assessed at 1st week after TQD (week 3). Lapsers (N = 105) were assigned a 2nd TQD 1 week later, and were allocated to the same 3 double‐blind treatment conditions as Phase 1 non‐responders. double‐blind varenicline, stopping NRT (N = 36) double‐blind augmentation of NRT with bupropion (N = 34) continuation on open‐label NRT alone (N = 35) Non‐lapsers (N = 130) remained on open‐label NRT throughout study duration All participants received dummy (placebo) versions of the other 2 treatments as well as their own active treatment. 47 participants were excluded from the analysis (27 Phase 1, 20 Phase 2) because of using contra‐indicated medications during the study or failing to meet other entry requirements. 1 individual died before EoT, and 1 was excluded for extreme CO change from the mean sample range	
Outcomes	Primary: CAR at weeks 8‐11 Secondary: CA from TQD for 11 weeks (EoT); 7‐day PPA at 6 months: CA from TQD to 6 months Validation: CO ≤ 10 ppm AEs and SAEs (reported, but not by treatment group)	
Notes	Phase 1 and Phase 2 groups combined for varenicline vs NRT analysis New for 2016 update Study funding: "Supported by a grant to Duke University from Philip Morris USA. Nicotine patches were donated by GlaxoSmithKline. The companies had no role in the planning or execution of the study, data analysis, or publication of results." Author declaration of interests: "Dr. Rose has served as a consultant for Targacept and Philip Morris USA and has a patent purchase agreement with Philip Morris International. Both authors have received research funding from Philip Morris USA."	
***Risk of bias***		
**Bias**	**Authors' judgement**	**Support for judgement**
Random sequence generation (selection bias)	Unclear risk	"randomly assigned"
Allocation concealment (selection bias)	Unclear risk	No information
Blinding (performance bias and detection bias) All outcomes	Unclear risk	No information
Incomplete outcome data (attrition bias) All outcomes	Low risk	Losses fully reported; exclusions for protocol violations or contra‐indicated medicines. 1 death and 1 'rogue' CO reading excluded
Selective reporting (reporting bias)	Low risk	Prespecified outcomes from trial registry all reported
Other bias	Unclear risk	Unexplained disparity between CONSORT (N = 103) and Results table (N = 108) denominators for rescue varenicline group

**Table:** Scharfenberg 1971

***Study characteristics***		
Methods	Country: East Germany Aim: to test the efficacy of cytisine for SC Setting: SC clinic, Magdeburg, July‐December 1967 Study design: double‐blind placebo‐controlled randomised trial	
Participants	1214 smokers recruited from 1452 applicants through smoking clinics and via initial press releases. 88.2% male. 2.5% of participants smoked < 10 CPD, 42.4% 10‐20 CPD, 48.9% 21‐30 CPD, 5.2% > 30 CPD 40.4% had smoked > 20 years. 40.6% had tried to quit at least once before Randomised to cytisine (607) or placebo (607) Exclusion criteria not stated (214 volunteers excluded at initial screening)	
Interventions	20‐day course of cytisine. 1.5 mg tablets: days 1‐3 6/day; days 4‐12 5/day; days 13‐16 4/day; days 17‐20 3/day Placebo tablets, same regimen Behavioural support: none	
Outcomes	Self‐reported abstinence at 4 weeks, 6 months and 2 years ITT analysis. Attrition rate 34% by longest follow‐up	
Notes	Study funding: not reported Author declaration of interests: not reported	
***Risk of bias***		
**Bias**	**Authors' judgement**	**Support for judgement**
Random sequence generation (selection bias)	Unclear risk	Not stated
Allocation concealment (selection bias)	Unclear risk	"a numbered pouch"
Blinding (performance bias and detection bias) All outcomes	Unclear risk	Not stated
Incomplete outcome data (attrition bias) All outcomes	Unclear risk	Not stated
Selective reporting (reporting bias)	Unclear risk	Not stated

**Table:** Schnoll 2019

***Study characteristics***		
Methods	Country: USA Setting: unclear Aim: to determine if extended‐use varenicline should be considered for treating tobacco use among cancer patients Study design: placebo‐controlled parallel RCT	
Participants	207 adult smokers with a diagnosis of cancer or a recurrence within the past 5 years, and smoking ≥ 5 cigarettes per week. Participants were motivated to quit. 50.7% female, mean age 58.5, and baseline average CPD 13.4 Exclusion criteria: daily use of nicotine products other than cigarettes, unstable substance abuse/dependence in the last year	
Interventions	Standard varenicline ‐ 12 weeks of varenicline 2 x 1 mg/day (titrated for first week) + 12 weeks of placebo Extended varenicline ‐ 24 weeks of varenicline 2 x 1 mg/day (titrated for first week) Participants were randomised 1:1. All participants had 7 x SC counselling sessions over 24 weeks (4 x in‐person, 3 x by telephone).	
Outcomes	Primary: 7‐day biochemically confirmed abstinence at weeks 24 and 52	
Notes	New for 2022 update Funding by R01 CA165001 and K24 DA045244. Pfizer provided medication and placebo free of charge. Author declaration of interests: "Drs. Schnoll and Hitsman receive medication and placebo free from, and provide consultation to, Pfizer. Drs. Schnoll and Dr. Kalhan have consulted for GlaxoSmithKline. Dr. Schnoll consults with Curaleaf."	
***Risk of bias***		
**Bias**	**Authors' judgement**	**Support for judgement**
Random sequence generation (selection bias)	Unclear risk	"The study statistician provided a randomization procedure (1:1) to the Penn Investigational Drug Service (IDS), which distributed pills."
Allocation concealment (selection bias)	Low risk	"The study statistician provided a randomization procedure (1:1) to the Penn Investigational Drug Service (IDS), which distributed pills. All personnel, except IDS, were blinded to assignment."
Blinding (performance bias and detection bias) All outcomes	Low risk	Objective verification and participants blinded during the second 12 weeks (first 12 weeks were open‐label as all participants received varenicline)
Incomplete outcome data (attrition bias) All outcomes	Low risk	77% and 75% in the 2 arms completed study according to flow‐chart
Selective reporting (reporting bias)	Low risk	Outcomes are reported as planned on clinical trials record

**Table:** Stein 2013

***Study characteristics***		
Methods	Country: USA Setting: 9 methadone‐maintained treatment centres in New England Aim: "[to] test varenicline versus placebo, and include a comparison condition of combination nicotine replacement therapy" Study design: randomised 3‐armed double‐blind controlled trial Dates conducted: December 2008‐January 2012 Analysis: sample sizes of 132 (varenicline) and 44 (placebo) estimated to give 80% power to detect quit rates of 20% and 2.5% respectively; the study was not powered to detect differences between varenicline and combination NRT	
Participants	315 adult methadone‐maintained smokers, smoking 10+ CPD, willing to set a quit date within the 1st week Allocated 3:1:3 to varenicline (137): placebo (45): combination NRT (133). Mean age 39.9, 47.6% women, 78.5% white, mean CPD 20, mean FTND 5.7	
Interventions	Varenicline: 24‐week course of varenicline tablets, 1st week titrated Placebo: 24‐week course of identical tablets and regimen Combination NRT: 24‐week course of NRT patch (42 mg for > 30 CPD, 21 mg if < 30 CPD), + ad lib nicotine gum (4 mg) as needed All participants received a standardised 15‐min session of advice to quit (5As model: Ask, Advise, Assess, Assist, and Arrange), and were asked to set a TQD for 8 days' time. All made monthly visits for support and top‐up medication. Participants were paid USD 30 for the baseline assessment and USD 40 for the 6‐month assessment	
Outcomes	Primary: 7‐day PPA at 6 months Secondary: CA from week 2 to 6 months; for non‐quitters: CPD reduction in the 28 days prior to 6‐month assessment Validation: CO < 8 ppm; urinary cotinine in varenicline and placebo participants claiming abstinence	
Notes	Funding: "Funding for this study was provided by the National Cancer Institute (RO1 CA129226). Dr. Stein is a recipient of a NIDA Mid‐Career Investigator Award (K24 DA00512). NCI and NIDA had no further role in the collection, analysis and interpretation of data; in the writing of the report; or in the decision to submit the paper for publication." New for 2016 update Author declaration of interests: "No conflict declared"	
***Risk of bias***		
**Bias**	**Authors' judgement**	**Support for judgement**
Random sequence generation (selection bias)	Unclear risk	"Participants were randomized to treatment after completing the baseline assessment". No further information
Allocation concealment (selection bias)	Unclear risk	No information
Blinding (performance bias and detection bias) All outcomes	Low risk	"double‐blind"; research assistants were "blind to participant group assignment"
Incomplete outcome data (attrition bias) All outcomes	Low risk	Losses to treatment and follow‐up reported; ITT analyses conducted
Selective reporting (reporting bias)	Low risk	Outcomes prespecified in trial registry reported

**Table:** Steinberg 2011

***Study characteristics***		
Methods	Country: New Jersey, USA Setting: Robert Wood Johnson Hospital (584‐bed, university‐based) Aim: to evaluate efficacy and safety of varenicline in hospital inpatients Dates conducted: August 2007‐March 2009 Study design: phase III triple‐blind pilot RCT	
Participants	79 adult smokers, aged 18+, smoking 10+ CPD; randomised to varenicline (40) or placebo (39) 59% men, mean age: 51, 72% white, 57% > 20 CPD, 40% FTND > 6 Admission diagnoses 57% CVD, 14% orthopaedic, 13% pulmonary, 16% other Exclusion criteria: standard pharmacotherapy criteria, + current use of any SC medications	
Interventions	Varenicline 1.0 mg x 2/day for 12 weeks, including week 1 at titrated dose Placebo tablets as above Initial visit by Clinic Co‐ordinator of local Tobacco Dependence Program for 5‐10 min counselling Subsequent sessions of 15‐min post‐discharge After discharge, data collection sessions at 4, 12 and 26 weeks, + 1 phone call at 2 weeks with research nurse USD 25 gift card for attendance at each follow‐up visit	
Outcomes	Primary outcome: 7‐day PPA at 26 weeks Secondary outcomes: 7‐day PPA at 4, 12 weeks. Repeated PPA at 4, 12 and 24 weeks. AEs and SAEs; withdrawal and craving on MNWS; motivation; CPD; utilisation of outpatient services; composite medical outcome Validation: CO validation ≤ 8 ppm. Self‐report accepted if unable to attend	
Notes	Study was funded and support by Robert Wood Johnson Foundation and Pfizer Repeated PPA at 4, 12 and 24 weeks used as strictest definition of abstinence and included in main MA New for 2012 update Author declaration of interests: "Dr. Steinberg had previously received honoraria for educational programs from Pfizer (2006–2009). The other authors declare that they have no conflicts of interest to disclose."	
***Risk of bias***		
**Bias**	**Authors' judgement**	**Support for judgement**
Random sequence generation (selection bias)	Low risk	"randomized in a 1:1 ratio through centralized telephone randomization process by the study statistician and hospital research pharmacist"
Allocation concealment (selection bias)	Low risk	See above
Blinding (performance bias and detection bias) All outcomes	Low risk	"The subject, research nurse, and treatment staff were blinded to treatment assignment"
Incomplete outcome data (attrition bias) All outcomes	Unclear risk	ITT analysis conducted; unvalidated smoking status included where ascertained for non‐attenders, but % of unvalidated status not reported
Selective reporting (reporting bias)	Low risk	All expected and predicted outcomes covered, except for detailed identification of SAEs

**Table:** Steinberg 2018

***Study characteristics***		
Methods	Country: USA Setting: community Aim: to conduct a proof‐of‐concept RCT of varenicline for smokers willing to reduce, but not quit smoking Study design: parallel, placebo‐controlled RCT	
Participants	53 current adult smokers, smoking at least 10 CPD for the past 6‐months, and interested in cutting down, but not in quitting in the next 30 days. 49.2% female, mean age 44.3, baseline average CPD 15 Exclusion criteria: any past use of varenicline, current use of bupropion/nortriptyline/nicotine preparations, use of tobacco products other than cigarettes more than once per month, currently receiving tobacco use disorder counselling, alcohol use disorder, drug abuse, psychosis, depression or suicidal ideation	
Interventions	Placebo Varenicline 2 x 1 mg/day (titrated for the first week) Participants were randomised on a 1:1 ratio. All participants received in‐person interactive behavioural support, which comprised 3 x 20‐min and 1 x 35‐min counselling visits	
Outcomes	Relevant outcomes Cigarette reduction goal (> 50% reduction) at 6 months Mean CPD over time at 1, 3 and 6 months Exhaled CO at 3 months and EoT Number of quit attempts	
Notes	New for 2022 update. Abstinence data were provided upon request by study authors Funding by Global Research Award for Nicotine Dependence (GRAND) grant #WS777117 – An independent competitive grants program supported by Pfizer. Pfizer had no role in the study design, collection, analysis or interpretation of the data, writing the manuscript, or the decision to submit the paper for publication. Author declaration of interests: "MLS and JMW have consulted for and received unrestricted educational grants and research grant support from Pfizer. SE‐L has no conflicts to declare."	
***Risk of bias***		
**Bias**	**Authors' judgement**	**Support for judgement**
Random sequence generation (selection bias)	Low risk	"Participants were randomized to active or placebo varenicline using an urn randomization procedure"
Allocation concealment (selection bias)	Unclear risk	Concealment not reported
Blinding (performance bias and detection bias) All outcomes	Low risk	Abstinence biochemically validated
Incomplete outcome data (attrition bias) All outcomes	Low risk	Attrition rates 12% in placebo arm and 29% in varenicline arm
Selective reporting (reporting bias)	Low risk	Abstience a prespecified outcome, but results not published. However, abstinence data were provided upon request by study authors

**Table:** Tashkin 2011

***Study characteristics***		
Methods	Country: USA (17 centres), Spain (3 centres), France (4 centres), Italy (3 centres) Setting: 27 research centres Aim: to test efficacy and safety of varenicline in smokers with COPD Dates conducted: May 2006‐April 2009 Study design: double‐blind placebo‐controlled RCT	
Participants	504 adult smokers with mild‐to‐moderate COPD, aged 35+, smoking 10+ CPD, motivated to quit; allocated to varenicline (250), or placebo (254). 62% men, mean age 57, CPD 24‐25, FTND score 5.9‐6.2 Treatment groups were comparable at baseline Exclusion criteria: standard pharmacotherapy trial criteria, + treatment with systemic corticosteroids or hospitalised for COPD in previous 4 weeks	
Interventions	Varenicline 1.0 mg x 2/day for 12 weeks, preceded by 1 week titrated dose Placebo tablets as above Both groups received SC educational booklet, + brief (≤ 10 min) counselling at weekly clinic visits throughout treatment phase, and phone call 3 days post‐TQD. At each visit smoking status reported and CO verified; throughout treatment and at week 52 lung function, respiratory symptoms, weight, blood pressure, pulse, temperature, ECGs, haematology and serum chemistry assessed, + AEs Follow‐up phase: smoking status + CO measured at weeks 13, 16, 24, 32, 40, 48 and 52; counselling and self‐reported status by phone at weeks 14, 20, 28, 36 and 44	
Outcomes	Primary outcome: CO‐validated CAR at weeks 9‐12 Secondary outcomes: CO‐validated CAR at weeks 9‐52 and 9‐24; 7‐day PPA at weeks 12, 24 and 52 Other outcomes: AEs; SAEs; weight change Validation was by expired CO ≤ 10 ppm Cessation analyses were ITT (all participants randomised), while tolerability and safety analyses were based only on those known to have used the intervention drug (N = 499). Attrition was 17% in the varenicline group and 24% in the placebo group during treatment phase, and 29% varenicline and 38% placebo who did not complete the study. This includes 2 deaths in the varenicline group and 1 in the placebo group	
Notes	The study was funded by Pfizer Inc New for 2010 update Author declaration of interests: "The authors have reported to the CHEST the following conflicts of interest: Dr Tashkin received grant support from Pfizer Inc and Nabi Pharmaceuticals and fees for attending advisory board meetings from Pfizer Inc. Dr Hays received a research grant from Pfizer Inc for the conduct of the clinical trial described in this manuscript. In the past 3 years, Dr Rennard has been a consultant or a member of an advisory board for Able Associates, Adelphi Research, Almirall/Prescott, APT Pharma/Britnall, Aradigm, AstraZeneca, Boehringer Ingelheim, Chiesi, CommonHealth, Consult Complete, COPDForum, Data‐Monitor, Decision Resources, Defined Health, Dey, Dunn Group, Eaton Associates, Equinox, Gerson, GlaxoSmithKline, Infomed, KOL Connection, M Pankove, MedaCorp, MDRx Financial, Mpex, Novartis, Nycomed, Oriel Therapeutics, Otsuka, Pennside Partners, Pfizer Inc (varenicline), Pharma Ventures, Pharmaxis, Price Waterhouse, Propagate, Pulmatrix, Reckner Associates, Recruiting Resources, Roche, Schlesinger Medical, SciMed, Sudler and Hennessey, TargeGen, Theravance, UBC, Uptake Medical, and VantagePoint Management. Dr Rennard has lectured for the American Thoracic Society, AstraZeneca, Boehringer Ingelheim, California Allergy Society, Creative Educational Concept, France Foundation, Information TV, Network for Continuing Ed, Novartis, Pfizer, and SOMA and has received industry‐sponsored grants from AstraZeneca, Biomarck, Centocor, Mpex, Nabi Pharmaceuticals, Novartis, and Otsuka. Ms Ma and Drs Lawrence and Lee are all employees of Pfizer Inc, own Pfizer Stock, and have Pfizer stock options."	
***Risk of bias***		
**Bias**	**Authors' judgement**	**Support for judgement**
Random sequence generation (selection bias)	Unclear risk	"participants were randomized"
Allocation concealment (selection bias)	Unclear risk	Not stated
Blinding (performance bias and detection bias) All outcomes	Unclear risk	"double blind" but details not stated
Incomplete outcome data (attrition bias) All outcomes	Unclear risk	Not stated
Selective reporting (reporting bias)	Unclear risk	All expected and predicted outcomes covered

**Table:** Tonstad 2011

***Study characteristics***		
Methods	Countries: France, Spain, Belgium, Sweden, Denmark, Norway Setting: 22 research centres Aim: to test the efficacy and safety of dianicline for SC Dates conducted: June 2006‐June 2007 Study design: double‐blind placebo‐controlled parallel group RCT Study name: EURODIAN study	
Participants	602 healthy adult volunteers, smoking 10+ CPD within previous 2 months, aged 18+; allocated to dianicline (300), or placebo (302). 42% men, mean age 45, mean CPD 21, mean previous quit attempts 3.4, mean FTND score 5.75. Treatment groups were comparable at baseline Exclusion criteria: standard pharmacotherapy trial criteria, plus any quit attempt in previous 3 months, any use of bupropion, NRT, tobacco other than cigarettes 3+ times in previous 3 months	
Interventions	Dianicline 40 mg twice/day for 7 weeks (not titrated) Placebo inactive tablets, same regimen TQD was set for days 3‐7 following baseline visit All participants received standardised brief counselling (≤ 10 min, based on *Smoke‐Free and Living It*) at each visit Weekly visits throughout weeks 1‐7, then (for treatment completers) at weeks 8, 10, 14, 18, 22 and 26 Smoking status and brief advice at each visit Participants completed smoking diaries	
Outcomes	Primary outcome: CO‐confirmed CAR for weeks 4‐7 Secondary outcomes: CO‐confirmed CAR at 26 weeks. PPA weeks 4‐7 Validation by expired CO < 10 ppm (all visits) and plasma cotinine ≤ 8 μg/L (weeks 4 and 7) Other outcomes: AEs, SAEs; craving and withdrawal symptoms Dropouts and losses to follow‐up were included in the analyses as continuing smokers (ITT analysis) 25.2% dianicline and 23% placebo participants did not complete the study. AE‐related dropouts were 4.3% dianicline and 7.6% placebo	
Notes	New for 2012 update The trial was funded by Sanofi‐Aventis. "The sponsor did not play a role in writing of the manuscript" Author declaration of interests: "Serena Tonstad and Philip Tønnesen have received honoraria for lectures and advising from sanofi‐aventis, Pfizer, Novartis, and GlaxoSmithKline, all manufacturers of drugs for smoking cessation."	
***Risk of bias***		
**Bias**	**Authors' judgement**	**Support for judgement**
Random sequence generation (selection bias)	Low risk	"a predefined, central, and computer‐generated randomization accessed through an Interactive Voice Response System assigned participants on a 1:1 ratio"
Allocation concealment (selection bias)	Low risk	See above
Blinding (performance bias and detection bias) All outcomes	Low risk	"Participants and investigators were blinded to drug treatment assignments"
Incomplete outcome data (attrition bias) All outcomes	Low risk	Dropouts fully reported
Selective reporting (reporting bias)	Low risk	All expected and predicted outcomes covered

**Table:** Tsai 2007

***Study characteristics***		
Methods	Country: Taiwan and Korea Setting: 5 sites in each country Aim: to test the efficacy and safety of varenicline for SC in Taiwanese and Korean smokers Dates conducted: February 2005‐March 2006 Study design: double‐blind placebo‐controlled RCT	
Participants	250 healthy adult volunteers, motivated to quit, aged 18‐75; allocated to varenicline (126), or placebo (124). 89% men, mean age 40.3, BMI < 15 or > 38 or weight < 45.5 kg, mean CPD 24, mean FTND score 5.1 Treatment groups were comparable at baseline Exclusion criteria: standard pharmacotherapy trial criteria	
Interventions	Varenicline 1.0 mg x 2/day Placebo tablet x 2/day Treatment period 12 weeks, 1st week titrated dosage. All participants received a SC booklet *Clearing the Air* at baseline, + brief counselling (≤ 10 min) at each clinic visit. Clinic visits at baseline and at weeks 1, 2, 3, 4, 6, 8, 10, 12, plus a 5‐min phone call at +3 days post‐TQD, and at weeks 5, 7, 9, 11 In follow‐up phase, clinic visits at weeks 13, 16, 20, 24 plus brief phone calls at weeks 14, 18, 22	
Outcomes	Primary outcome: CO‐validated CAR at 9‐12 weeks Secondary outcomes: CO‐validated CAR at 9‐24 weeks; 7‐day PPA at weeks 12 and 24 Validation was by expired CO ≤ 10 ppm Other outcomes: withdrawal symptoms (using MNWS, QSU‐brief and mCEQ), AEs Dropouts and losses to follow‐up were included in the analyses as continuing smokers (ITT analysis) Attrition in treatment phase was 2.8%, losses to follow‐up 2.5% of treatment completers	
Notes	Trial was funded by Pfizer Inc New for 2008 update Author declaration of interests: "Drs. Tsai and Cho have been members of Pfizer‐sponsored advisory panels and, together with Drs. Cheng, Kim, and Hsueh, were investigators for a Pfizer‐sponsored clinical trial."	
***Risk of bias***		
**Bias**	**Authors' judgement**	**Support for judgement**
Random sequence generation (selection bias)	Low risk	"randomly permuted blocks" (block size=4)
Allocation concealment (selection bias)	Low risk	Web‐ and telephone‐based assignment
Blinding (performance bias and detection bias) All outcomes	Low risk	Participants, investigators, study staff and sponsor personnel
Incomplete outcome data (attrition bias) All outcomes	Unclear risk	No information, but very high compliance rates
Selective reporting (reporting bias)	Unclear risk	All expected and predicted outcomes covered

**Table:** Tsukahara 2010

***Study characteristics***		
Methods	Country: Japan Setting: cessation clinic in Fukuoka University Hospital Aim: to test the efficacy and safety of varenicline for SC in Japanese smokers Dates conducted: August 2008‐November 2009 Study design: open‐label RCT Study name: the VN‐SEESAW Study	
Participants	32 adult smokers, motivated to quit, allocated to varenicline (16) or nicotine patch (16). 75% men, mean age 46, mean CPD 28 (varenicline), 25 (patch), mean Brinkman index score (CPD x years smoking) 702. 71% had tried to quit previously, and 7% had used nicotine patches before Standard pharmacotherapy trial exclusion criteria, plus attendance at any SC clinic during previous 12 months	
Interventions	Open‐label varenicline 1.0 mg x 2/day for 12 weeks, following 1 week titration Open‐label nicotine patch for 8 weeks (52.5 mg/day for 4 weeks, 35 mg/day for 2 weeks, 17.5 mg/day for 2 weeks) No non‐treatment or placebo control group Varenicline group received 8 clinic visits and nicotine group 5 visits over 12 weeks, with 5 brief counselling sessions (≤ 10 min)	
Outcomes	CO‐confirmed CAR at 9‐12 weeks, and self‐reported at 9‐24 weeks by phone interview Validation by expired CO < 8 ppm at 12 weeks, but not at 24 weeks Other outcomes: safety and tolerability by week 12, using MNWS at weeks 2, 4, 8 and 12. Also used Stress Check List and Strait‐trait Anxiety Inventory Dropouts and losses to follow‐up were included in the analyses as continuing smokers (ITT analysis) Attrition in treatment phase was 12.5% from each group	
Notes	The study was supported by the Japanese Ministry of Education, Science and Culture, Fukuoka University and FU‐Global program New for 2010 update Author declaration of interests: not reported	
***Risk of bias***		
**Bias**	**Authors' judgement**	**Support for judgement**
Random sequence generation (selection bias)	Unclear risk	"by computer" allocating men: women 3:1 to reflect Japanese smoking prevalence (M: 40%, F: 12%)
Allocation concealment (selection bias)	Unclear risk	Not stated
Blinding (performance bias and detection bias) All outcomes	Low risk	Participants and personnel were not blinded to treatment. However, both groups received an active licensed SC treatment and there is no reason to believe that participants would have favoured one over the other.
Incomplete outcome data (attrition bias) All outcomes	Unclear risk	Not stated
Selective reporting (reporting bias)	Low risk	All expected and predicted outcomes covered

**Table:** Tuisku 2016

***Study characteristics***		
Methods	Country: Finland Setting: community Aim: to investigate the efficacy of varenicline and the nicotine patch as a SC aid in volunteer daily smokers in their twenties Study design: parallel, placebo‐controlled RCT	
Participants	288 current smokers aged 18‐26 year‐old, who had smoked daily for at least the past month, and smoked ≥ 100 cigarettes in their life. Participants were motivated to quit. 50.2% of participants were female, median age was 21, and average CPD at baseline 14 Exclusion criteria: current drug or alcohol abuse, known allergy towards medications used in the study, lactation, pregnancy or intention to become pregnant during the study period	
Interventions	Light smokers were randomly assigned into 2 groups: Placebo patch for 8 weeks NRT ‐ nicotine patch 10 mg/16 h for 8 weeks Heavy smokers were randomly assigned into two groups: NRT ‐ nicotine patch 15 mg/16 h for 8 weeks Varenicline ‐ 12 weeks 2 x 1 mg/day (titrated for first week)	
Outcomes	Primary: self‐reported smoking abstinence at week 12 Secondary: self‐reported smoking abstinence at weeks 4 and 26; self‐reported abstinence verified by saliva cotinine level at week 12	
Notes	New for 2022 update Funding by Ministry of Social Affairs and Health, Finland; Finnish Research Foundation of the Pulmonary Disease; Finnish Medical Society Duodecim Author declaration of interests: "The authors declare no conflict of interest."	
***Risk of bias***		
**Bias**	**Authors' judgement**	**Support for judgement**
Random sequence generation (selection bias)	Low risk	"After assessment… at the baseline visit, simple randomisation with a computer‐generated random list… was used to allocate study subjects into the different treatment groups”
Allocation concealment (selection bias)	Unclear risk	No information reported on allocation concealment
Blinding (performance bias and detection bias) All outcomes	Low risk	Abstinence was self‐reported, however placebo or another licensed treatment were used as comparators and behavioural support was balanced between groups.
Incomplete outcome data (attrition bias) All outcomes	Low risk	Attrition rates were similar between study arms.
Selective reporting (reporting bias)	High risk	It appears 12‐month follow‐up was planned (NCT01531049) but is not reported in the published paper.

**Table:** Tulloch 2016

***Study characteristics***		
Methods	Country: Canada Setting: community Aim: to evaluate smoking abstinence with standard nicotine patch, extended use of combined formulations of NRT, or varenicline Study design: parallel RCT	
Participants	737 smokers, including those with medical and psychiatric comorbidities, motivated to quit. 46.6% female, mean age 48.61, baseline average CPD 23.2 Exclusion criteria: use of NRT or varenicline for > 72 consecutive h in the past month; serious cardiac arrhythmias or a myocardial infarction or cerebral vascular accident within the previous 10 days; severe or unstable angina pectoris; end‐stage renal disease or use of cimetidine; alcohol or substance abuse in the previous 3 months; unstable psychiatric symptoms; an inability to understand English or French. Women were excluded if pregnant, lactating, or likely to become pregnant in the next year.	
Interventions	Nicotine patch ‐ up to 10 weeks of patches (21 mg daily maximum dose) Combination NRT ‐ up to 22 weeks of nicotine patches (35 mg daily maximum dose) and gum or inhaler for up to 22 weeks Varenicline ‐ 2 x 1 mg/day for up to 24 weeks All participants received 6 x 15‐min cessation counselling interactive behavioural support from experienced nurses.	
Outcomes	Primary: CO‐confirmed CA rates from weeks 5–52. Secondary: CA rates from weeks 5–10 and 5–22; CO‐confirmed 7‐day PPA at weeks 10, 22, and 52	
Notes	New for 2022 update Funding by the Heart and Stroke Foundation of Ontario. Study authors have previously received research grants and delivered educational presentation for Pfizer. Author declaration of interests: "AP and RR have received research grants from Pfizer. AP and BR have been paid for developing and delivering educational presentations for Pfizer. AP is on the advisory board for Pfizer and Johnson & Johnson."	
***Risk of bias***		
**Bias**	**Authors' judgement**	**Support for judgement**
Random sequence generation (selection bias)	Low risk	"The randomization sequence was generated using a computer generated algorithm in Statistical Analysis Software (SAS program) by a researcher not involved in the study and blinded to the identity of participants."
Allocation concealment (selection bias)	Low risk	"Randomization numbers were placed in opaque, sealed, and consecutively numbered envelopes and opened following the completion of the baseline data collection."
Blinding (performance bias and detection bias) All outcomes	Low risk	Open‐label with higher intensity medication provided in the combination NRT arm than the nicotine patch arm.
Incomplete outcome data (attrition bias) All outcomes	Low risk	Attrition was < 50% and comparable between arms.
Selective reporting (reporting bias)	Low risk	Abstinence was reported as stated in trial register (NCT01623505)

**Table:** Vinnikov 2008

***Study characteristics***		
Methods	Country: Kyrgyzstan Setting: Mining company (Kumtor Operating Company) Aim: to test the efficacy and safety of cytisine for SC in a workplace setting Study design: double‐blind placebo‐controlled parallel‐group RCT	
Participants	197 adult smokers, aged 20+, smoking at least 15 CPD, no prior use of cytisine, and motivated to quit Randomised to cytisine (100) or placebo (97). 26 (15 cytisine, 11 placebo) who took no medication were excluded from trial report 97% men, mean age 39, mean CPD 22, mean FTND 5.3, 86% had tried to quit previously; mean previous quit attempts 3.3 Exclusion criteria: standard pharmacotherapy trial criteria	
Interventions	Tabex tablets (1.5 mg cytisine): first 3 days: 6 tablets/day; reduce smoking by half days 4‐12: 5 tablets/day; stop smoking completely days 13‐16: 4 tablets/day days 17‐20: 3 tablets/day days 21‐22: 2 tablets/day days 23‐25: 1 tablet/day Placebo tablets, same regimen Treatment period was 25 days, with TQD Day 5. All participants received "behavior counselling" (no further detail)	
Outcomes	Primary outcome: CO‐validated CAR from day 5 to week 8 Secondary outcome: CO‐validated CAR from day 5 to week 26 Validation was by expired CO ≤ 8 ppm Other outcomes: change in health‐related QoL measures, changes in body weight, AEs, SAEs Attrition to 8 weeks was 6 in cytisine group and 7 in placebo group; to 26 weeks 10 in cytisine group and 16 in placebo group	
Notes	New for 2012 update Additional information supplied by the study author Study funding: not reported Author declaration of interests: not reported	
***Risk of bias***		
**Bias**	**Authors' judgement**	**Support for judgement**
Random sequence generation (selection bias)	Low risk	"Randomization was done by independent statistician in an Excel programme and the randomization key was kept by an independent person"
Allocation concealment (selection bias)	Low risk	See above
Blinding (performance bias and detection bias) All outcomes	Low risk	"Nor patients neither investigators did not know where Tabex and where placebo were"; "follow‐up was blind"
Incomplete outcome data (attrition bias) All outcomes	Unclear risk	26 participants who did not take a single treatment dose were excluded from denominators by authors (restored to our MAs)
Selective reporting (reporting bias)	Low risk	Expected and predicted outcomes reported

**Table:** Walker 2014

***Study characteristics***		
Methods	Country: New Zealand Setting: National Quitline Aim: "a non‐inferiority trial to investigate whether cytisine was at least as effective as nicotine‐replacement‐therapy" Study design: parallel‐group non‐inferiority RCT Dates conducted: March 2011‐February 2013	
Participants	1310 daily smokers, callers to the NZ National Quitline, aged 18+, motivated to quit. Allocated to cytisine (655) or to open‐label NRT (655). Mean age 38, 57% women, 33% NZ Maori, mean CPD 19, mean FTND 5.4	
Interventions	25‐day course of cytisine (Tabex) tablets, + NRT vouchers in case they needed them after completing the cytisine course Usual care, i.e. 8‐week course of NRT (patch, gum or lozenge), tailored to dependence level, supplied by vouchers All participants received standard Quitline support, i.e. average 3 x 10‐15‐min calls over 8 weeks	
Outcomes	Self‐reported CAR (5 cigarettes or fewer) at 1 month CAR and 7‐day PPA (no smoking) at 1 week, 1 month, 2 months and 6 months. AEs Validation: none used	
Notes	Funding by Health Research Council of New Zealand; cytisine supplied at no cost by Sopharma New for 2016 update Author declaration of interests: "Dr. McRobbie reports receiving lecture fees from Johnson & Johnson and grant support from Pfizer. No other potential conflict of interest relevant to this article was reported."	
***Risk of bias***		
**Bias**	**Authors' judgement**	**Support for judgement**
Random sequence generation (selection bias)	Low risk	"randomly allocated, by computer ... in a 1:1 ratio"
Allocation concealment (selection bias)	Low risk	"Randomization was stratified with the use of minimization according to sex, ethnicity (Maori, Pacific Islander, or non‐Maori and non–Pacific Islander), and cigarette dependence, which was determined by means of the Fagerström Test of Cigarette Dependence, in which smokers were assigned to one of two groups: those with scores of 5 or lower, indicating lower dependence, and those with scores greater than 5, indicating greater dependence"
Blinding (performance bias and detection bias) All outcomes	High risk	"Participants and researchers collecting outcome data were aware of treatment allocation"
Incomplete outcome data (attrition bias) All outcomes	Low risk	Losses fully reported. By 6 months, 182 cytisine participants (28%) lost to follow‐up, and 16 withdrawals; 173 NRT participants (26%) lost to follow‐up, and 14 withdrawals. 19 cytisine users crossed over to NRT, and 1 NRT user crossed over to cytisine. ITT analyses conducted
Selective reporting (reporting bias)	Low risk	Prespecified outcomes from protocol all reported
Other bias	Unclear risk	Cytisine was supplied free, while NRT users had to pay a nominal charge (NZD 3 for an 8‐week course of each NRT item).

**Table:** Walker 2021

***Study characteristics***		
Methods	Country: New Zealand Setting: community pharmacy (for drug distribution), participants' homes Aim: to determine whether cytisine was at least as effective as varenicline in supporting smoking abstinence for ≥ 6 months in New Zealand indigenous Māori or *whānau* (extended‐family) of Māori Study design: open‐label, parallel, RCT	
Participants	679 adult daily smokers who identified as Māori or *whānau* of Māori, motivated to quit in the next 2 weeks, and were eligible for subsidised varenicline. 69.6% female, mean age 43, baseline average CPD 15.5 Exclusion criteria: pregnant/breastfeeding; currently using SC medication (including e‐cigarettes); enrolled in another cessation programme/study; used varenicline or cytisine in the previous 12 months; known hypersensitivity; self‐reported moderate/severe renal impairment; treatment for active/latent tuberculosis; heart attack, stroke or severe angina within the previous 2 weeks; uncontrolled high blood pressure; history of seizures	
Interventions	Varenicline ‐ 2 x 1.5 mg/day (titrated for first week). Cytisine ‐ days 1–3: 6 x 1.5 mg/day; days 4–12: 5 x 1.5 mg/day; days 13–16: 4 x 1.5 mg/day); days 17–20: 3 x 1.5 mg/day; and days 21–25: 2 x 1.5 mg/day. A maintenance dose of cystine 2 x 1.5 mg/day was added for day 26 to week 12 to match the treatment duration of varenicline. All participants received low‐intensity cessation behavioural support from the prescribing doctor and community stop‐smoking services or a research assistant.	
Outcomes	Primary: CA at 6 months (verified with exhaled CO < 9 ppm) Secondary outcomes measured at 1, 3, 6 and 12 months post‐quit date included: self‐reported CA, 7‐day PPA, CPD, time to (re)lapse, AEs, treatment adherence/compliance and acceptability, nicotine withdrawal/urge to smoke and healthcare utilization/health‐related QoL	
Notes	New for 2022 update Funding by the Health Research Council of NZ. Manufacturer supplied the treatment free of charge. Author declaration of interests: "All authors report grants from the Health Research Council of New Zealand and non‐financial support from Achieve Life Sciences during the conduct of the study. N. W., C.B.and M.V. V.P. report grants from Pfizer, grants from the Health Research Council of New Zealand, outside the submitted work; and has previously undertaken two trials of e‐cigarettes for smoking cessation [with e‐cigarettes purchased from a NZ e‐cigarette on‐line retailer (NZVAPOR, https://www.nzvapor.com/), e‐liquid for one trial purchased from Nicopharm, Australia (https://www. nicopharm.com.au/) and nicotine patches supplied by the NZ Government via their contract with Novartis (Sydney, Australia)]. Neither NZVAPOR nor Nicopharm have links with the tobacco industry. C.B. also reports personal fees from the Moffat Cancer Center, University of Florida, USA, and personal fees from Virginia Commonwealth University, USA outside the submitted work. J.B. also reports personal fees from New Zealand Ministry of Health Natural Health Products (NHPs) Regulations Subcommittee on the Permitted Substances List (member of subcommittee 2016–17), non‐financial support from Uppsala Monitoring Centre, Sweden (who manages the technical and scientific aspects of the WHO Programme for International Drug Monitoring); honorary consultant and herbal safety signal reviewer (2004–current), outside the submitted work. None of the above parties had any role in the design, conduct, analysis or interpretation of the trial findings or writing of the resulting publication."	
***Risk of bias***		
**Bias**	**Authors' judgement**	**Support for judgement**
Random sequence generation (selection bias)	Low risk	"Block randomization was undertaken, with varying block sizes. The randomization sequence was prepared by the trial statistician using R and loaded into the REDCap database, which was the accessed by the study doctor via a computer at the point of randomization."
Allocation concealment (selection bias)	Low risk	"Block randomization was undertaken, with varying block sizes. The randomization sequence was prepared by the trial statistician using R and loaded into the REDCap database, which was the accessed by the study doctor via a computer at the point of randomization."
Blinding (performance bias and detection bias) All outcomes	Low risk	"Abstinence was verified by a researcher or community‐based cessation provider, using standardized exhaled carbon monoxide (CO) measurement with a Bedfont Smokerlyzer".
Incomplete outcome data (attrition bias) All outcomes	High risk	Only 39% of participants in both groups were followed up at 6 months (12 months is unclear) although these numbers do not match main results table. Methods say multiple imputation used but not clear what effect this had on findings.
Selective reporting (reporting bias)	Low risk	Outcomes reported match the trial protocol paper

**Table:** Wang 2009

***Study characteristics***		
Methods	Country: China (10 sites), Singapore (3 sites), Thailand (2 sites) Aim: to test the efficacy and safety of varenicline for SC in Chinese, Singaporean and Thai smokers Dates conducted: not stated Study design: double‐blind placebo‐controlled RCT	
Participants	333 healthy adult volunteers, aged 18‐75; allocated to varenicline (165), or placebo (168). 97% men, mean age 39, BMI > 15 and < 38 or weight > 45.5 kg, mean CPD 20, mean FTND score 5.4. Treatment groups were comparable at baseline. 58% had never tried to quit before Exclusion criteria: standard pharmacotherapy trial criteria, plus any use of NRT or bupropion in previous 6 months	
Interventions	Varenicline 1.0 mg x 2/day Placebo tablet x 2/day Treatment period 12 weeks, 1st week titrated dosage. All participants received a SC booklet at baseline, + brief counselling (≤ 10 min) at each clinic visit, except for weeks 5 and 7, when counselling was conducted by phone In follow‐up phase, clinic visits at weeks 13, 16, 20, 24 plus brief phone calls at weeks 14, 18, 22. Dosing and CO checked at each visit, and lab samples taken at weeks 12 and 24	
Outcomes	Primary outcome: CO‐confirmed CAR for weeks 9‐12 Secondary outcomes: CO‐confirmed CAR for weeks 9‐24; 7‐day PPA at 24 weeks Validation by expired CO < 10 ppm Other outcomes: AEs; long‐term quit rates Dropouts and losses to follow‐up were included in the analyses as continuing smokers (ITT analysis) Attrition in treatment phase was 3.0% in varenicline group, and 3.6% in placebo group. By week 24, 4.2% of had dropped out of each group	
Notes	The trial was funded by Pfizer Inc New for 2010 update Author declaration of interests: "Pfizer Inc. funded the study and was involved with its design, analysis and writing the manuscript. All authors had complete access to all relevant data. Dahlia Garza and Simon Davies are employees of Pfizer Inc., and therefore hold shares in the company. Editorial support was provided by Aideen Young, PhD, of UBC Scientific Solutions and funded by Pfizer Inc. None of the other authors hold shares in any companies. Chen Wang and Dan Xiao are affiliated with the WHO Collaborating Centre for Tobacco or Health. WHO had no role in the study’s funding, design, analysis or write‐up."	
***Risk of bias***		
**Bias**	**Authors' judgement**	**Support for judgement**
Random sequence generation (selection bias)	Unclear risk	"eligible subjects were randomized in a 1:1 ratio"
Allocation concealment (selection bias)	Unclear risk	See above
Blinding (performance bias and detection bias) All outcomes	Unclear risk	"double‐blind", but no further information
Incomplete outcome data (attrition bias) All outcomes	Unclear risk	No information, but very high compliance rates
Selective reporting (reporting bias)	Low risk	All expected and predicted outcomes covered

**Table:** West 2011

***Study characteristics***		
Methods	Country: Poland Setting: SC clinic in Warsaw Aim: to test the efficacy and safety of cytisine for SC with minimal counselling and support Dates conducted: December 2007‐September 2010 Study design: single‐centre, double‐blind placebo‐controlled parallel‐group RCT	
Participants	740 healthy adults, smoking 10+ CPD, motivated to quit. Randomised to cytisine (370) or placebo (370) 46.5% men, mean age 48, mean CPD 23, prior quit attempts 82%, mean FTND 6.2 Exclusions were current psychiatric disorder or any medical condition contraindicated on cytisine label	
Interventions	Tabex tablets (1.5 mg cytisine): first 3 days: 6 tablets per day days 4‐12: 5 tablets per day days 13‐16: 4 tablets per day days 17‐20: 3 tablets per day days 21‐22: 2 tablets per day days 23‐25: 1 tablet per day Placebo tablets, same regimen Treatment period was 25 days. Quitting advice, randomisation and drugs dispensed at baseline visit; phone calls at TQD + 1 week later (+ optional clinic visit). Clinic visit 4 weeks post‐TQD, then phone calls at 6 months and 12 months, with visit to confirm abstinence if claimed. Behavioural support was minimal, to simulate likelihood of real‐world conditions in countries where Tabex is available	
Outcomes	Primary: CO‐validated abstinence 12 months after EoT. Abstinence defined as smoking < 5 cigarettes during preceding 6 months, and none in week before visit Secondary outcomes: sustained CO‐validated abstinence at 6‐month follow‐up; 2‐week PPA at 4 weeks; 7‐day PPA at 12 months Validation was expired CO < 10 ppm Attrition: 79 (cytisine) and 89 (placebo) participants were lost to follow‐up over 12 months. Drug discontinuation or reduction rates similar in both groups: 6.2% for cytisine and 4.6% for placebo Other outcomes: AEs, SAEs	
Notes	New for 2012 update The trial was funded by the UK National Prevention Research Initiative, Cancer Research UK, and the National Institute for Health Research Author declaration of interests: not reported	
***Risk of bias***		
**Bias**	**Authors' judgement**	**Support for judgement**
Random sequence generation (selection bias)	Low risk	"performed by a statistician at Sopharma, who generated a list of study‐group assignments for 740 participants with nQuery Advisor software. Assignments were made in variable block sizes of either 20 (10 cytisine, 10 placebo) or 10 (5 and 5)"
Allocation concealment (selection bias)	Low risk	See above
Blinding (performance bias and detection bias) All outcomes	Low risk	"Trial staff and participants were unaware of the group assignments and the randomization scheme"
Incomplete outcome data (attrition bias) All outcomes	Low risk	Dropouts and attrition fully reported
Selective reporting (reporting bias)	Low risk	All expected and predicted outcomes reported

**Table:** Westergaard 2015

***Study characteristics***		
Methods	Country: Denmark Aim: to evaluate the effect of varenicline on tobacco cessation in young smokers suffering from asthma Dates conducted: not stated Study design: double‐blind placebo‐controlled RCT	
Participants	52 young (aged 19‐40) smokers with asthma, randomised to varenicline (26) or placebo (26). CPD ≥ 10; FTND 5.6	
Interventions	Varenicline: presumed standard regimen: varenicline 1.0 mg x 2/day Placebo tablet x 2/day No further details	
Outcomes	Primary: presumed PPA at 12 weeks Secondary: presumed PPA at 0, 6, 24 weeks Validation by expired CO < 10 ppm Also assessed asthma symptom score, general health quality score (15D) and methacholine challenge	
Notes	Study funding: "The Respiratory Research Unit has received financial support from Pfizer Inc. [WS807136] in relation to the conduction of the present study. Furthermore, the Varenicline and placebo tablets were provided by Pfizer Inc. Pfizer Inc. has not been involved in study design, in the collection, analysis and interpretation of data; in the writing of the report; and in the decision to submit the article for publication." Author declaration of interests: not reported Author supplied further details	
***Risk of bias***		
**Bias**	**Authors' judgement**	**Support for judgement**
Random sequence generation (selection bias)	Unclear risk	Not stated. ""randomized, placebo‐controlled, double‐blinded trial"
Allocation concealment (selection bias)	Unclear risk	Not stated
Blinding (performance bias and detection bias) All outcomes	Unclear risk	Not stated. "double‐blinded
Incomplete outcome data (attrition bias) All outcomes	Unclear risk	Not stated; ITT analysis conducted
Selective reporting (reporting bias)	Unclear risk	Not stated

**Table:** Williams 2007

***Study characteristics***		
Methods	Country: USA and Australia Setting: 9 research centres (8 USA, 1 Australia) Aim: to test the safety of long‐term (12 months) use of varenicline in smokers trying to quit Study design: double‐blind placebo‐controlled RCT Dates conducted: October 2003‐March 2005	
Participants	377 adult smokers, aged 18‐75, smoking at least 10 CPD. 49.9% men, 88.6% white, average CPD at baseline 23, mean FTND 5.5 in treatment group, 6.05 in control group. Allocated to varenicline (251) or placebo (126) Exclusion criteria: standard pharmacotherapy trial criteria, + no use of NRT, antidepressants, antipsychotics, naltrexone during study period	
Interventions	Varenicline 1 mg x 2/day, titrated for first week Placebo inactive tablets, same regimen All participants received booklet *Clearing the Air*. Brief counselling (≤ 10 min) at each visit TQD was 1st day of week 1 visit (7‐10 days post‐randomisation) Treatment period was 52 weeks. Weekly visits throughout weeks 1‐8, then every 4 weeks to week 52, + week 53 assessment Blood and urine samples taken at screening, baseline, weeks 2, 12, 24, 36, 52 (or early termination) Complete physical exam at baseline, weeks 24 and 52; BP, pulse and weight measured at all visits, ECG at screening, baseline, weeks 2, 24 and 52 (or early termination)	
Outcomes	Primary outcome: safety of smokers treated continuously with varenicline over 52 weeks, measured at week 53 by level and tolerability of AEs and incidence of SAEs Secondary outcome: 7‐day CO‐verified PPA at all clinic visits (expired CO ≤ 10 ppm) Other outcomes: weight change; changes in vital signs Attrition was 46.2% in varenicline group, 53.2% in control group by end of study	
Notes	This was a safety study, with cessation rates collected as a secondary outcome The trial was funded and conducted by Pfizer Inc In the first version of this review, this trial appeared as Reeves 2006 (unpublished data) Author declaration of interests: "Kathryn E. Williams, Karen R. Reeves, Clare B. Billing, Jr, Ann M. Pennington, and Jason Gong are all employees of Pfizer and were involved in designing the study, data monitoring, data management, statistical analysis, and interpretation of the results, as well as the drafting, editing, and reviewing of this manuscript"	
***Risk of bias***		
**Bias**	**Authors' judgement**	**Support for judgement**
Random sequence generation (selection bias)	Unclear risk	Randomisation 2:1 varenicline to placebo. No detailed information reported
Allocation concealment (selection bias)	Unclear risk	No information reported
Blinding (performance bias and detection bias) All outcomes	Unclear risk	No information reported
Incomplete outcome data (attrition bias) All outcomes	Low risk	Missing CO and/or visit taken as smokers
Selective reporting (reporting bias)	Low risk	Primary outcome was safety, so minimal cessation data

**Table:** Williams 2012

***Study characteristics***		
Methods	Countries: Canada, USA Setting: 12 sites Aim: to evaluate primarily safety, but also efficacy of varenicline in smokers with schizophrenia or schizoaffective disorders Dates conducted: May 2008‐April 2010 Study design: double‐blind placebo‐controlled RCT. Sample size [120] was considered sufficient to detect a between‐group difference in 7‐day PPA "for a medium effect size"	
Participants	128 adults, diagnosed with stable schizophrenia or schizoaffective disorders, smoking at least 15 CPD and motivated to quit. Randomised to varenicline (85) or placebo (43). 77% men aged 18‐75	
Interventions	Varenicline 1.0 mg x 2/d for 12 weeks, including week 1 at titrated dose Placebo tablets as above Weekly clinic visits, for safety and efficacy, ≤ 30‐min counselling sessions; after treatment phase, clinic visits at weeks 13, 16, 20, 24, with brief phone calls at weeks 14, 18 and 22. Follow‐up sessions included brief (≤ 10 min) counselling. AEs collected to 30 days after treatment, and neuropsychiatric AEs to week 24	
Outcomes	Primary outcome: N of participants with AEs and SAEs from baseline to 30 days after EoT (12 weeks). N of participants with psychiatric AEs, including suicidal ideation or behaviour Secondary outcomes: CO‐confirmed PPA at weeks 12 and 24; 50%+ reduction in CPD; change in CPD from baseline. Assessments on mood and psychiatric scales Validation was by exhaled CO ≤ 10 ppm Dropouts in treatment phase: 14 (varenicline), 3 (placebo); follow‐up phase: 10 (varenicline), 3 (placebo) 1 varenicline participant died during follow‐up phase	
Notes	The study was funded by Pfizer New for 2012 update Author declaration of interests: "Dr Williams has received research support from the National Institutes of Health (NIH [National Institute of Mental Health and National Institute on Drug Abuse]) and Pfizer and has received further support from Pfizer for advisory board membership and product support. Dr Anthenelli provides consultancy and/or advisory services for Pfizer and GlaxoSmithKline, and his laboratory receives funding support from the National Institute on Alcohol Abuse and Alcoholism, the Department of Veterans Affairs, Pfizer, Nabi Biopharmaceuticals, and sanofi‐aventis. He has also received honoraria from Pfizer. Dr Morris has received research support from Pfizer. Drs Thompson and Yunis and Ms Treadow are employees of and shareholders in Pfizer. Dr George has received consulting fees from Pfizer, sanofi‐aventis, Novartis, Eli Lilly, Prepharm, AstraZeneca, and Janssen; has received research support from Pfizer and Sepracor; and has received grant support relevant to the study medication from the NIH, Canadian Institutes of Health Research, Canada Foundation for Innovation, and the Ontario Mental Health Foundation"	
***Risk of bias***		
**Bias**	**Authors' judgement**	**Support for judgement**
Random sequence generation (selection bias)	Unclear risk	"Subjects were randomized (2:1) to varenicline or placebo ... and were stratified according to antipsychotic medication type (typical vs atypical)."
Allocation concealment (selection bias)	Unclear risk	Not stated
Blinding (performance bias and detection bias) All outcomes	Low risk	Abstinence biochemically validated
Incomplete outcome data (attrition bias) All outcomes	Unclear risk	Not yet reported
Selective reporting (reporting bias)	Low risk	Outcomes prespecified in trial registry reported

**Table:** Windle 2018

***Study characteristics***		
Methods	Country: 40 centres in USA and Canada Setting: hospitals Aim: to determine the efficacy and safety of varenicline for increasing smoking abstinence rates through smoking reduction Study design: double‐blind placebo‐controlled multicentre RCT Study name: evaluation of varenicline in SC for patients post‐acute coronary syndrome (EVITA) Dates conducted: not stated	
Participants	302 adult smokers, aged 18+, smoking 10+ CPD, interested in trying to quit, hospitalised in USA or Canada for acute coronary syndrome (MI or unstable angina). Mean age 55, 25% women, mean CPD 21.5 Allocated to varenicline (151) or placebo (151) Exclusions: excessive alcohol, history of panic disorder, psychosis, bipolar disease, dementia, renal or hepatic impairment, current or recent drug use, history of suicidal ideation/attempt or family history of suicide	
Interventions	Varenicline 12 weeks, titrated 1st week Placebo 12 weeks, titrated 1st week Medication was begun in hospital. All participants received low‐intensity counselling Follow‐up at weeks 1, 2 and 8 by phone, and clinic visits at weeks 4, 12, 24 and 52	
Outcomes	Primary: 7‐day PPA at week 24 Secondary: CAR at all follow‐up visits, 7 day PPA at other follow‐up visits, ≥ 50% reduction in CPD Measures of side effects and SAEs Validation: CO ≤ 10 ppm	
Notes	New for 2016 update Study funding: "EVITA was an investigator‐initiated trial that received funding and the study drug and placebo from Pfizer Inc. Pfizer had no role in the design, conduct, analysis, interpretation of data, or reporting of the EVITA trial." Author declaration of interests: "Shamir Mehta reports funding from AstraZeneca, Boston Scientific, Bayer and Abbott. Beth Abramson has received grants or research support from AstraZeneca and Sanofi; honoraria from Amgen, AstraZeneca, Boehringer Ingelheim, Bristol–Myers Squibb, Novartis, Fournier, Merck, Pfizer, Servier and Sanofi; and consulting fees from Amgen, Bayer, Boehringer Ingelheim, Sanofi and Servier. She authored Heart Health for Canadians. Mark Eisenberg, Payam Dehghani, François Grondin and Mina Madan received honoraria from Pfizer for providing continuing medical education on SC. No other competing interests were declared"	
***Risk of bias***		
**Bias**	**Authors' judgement**	**Support for judgement**
Random sequence generation (selection bias)	Low risk	"Patients were randomized to either varenicline or matching placebo... Randomization was performed by enrolling center personnel and stratified by center using a computer‐generated list of permuted blocks of 2 and 4"
Allocation concealment (selection bias)	Unclear risk	Not stated
Blinding (performance bias and detection bias) All outcomes	Low risk	Described as "double‐blind", but no further detail. Abstinence biochemically validated
Incomplete outcome data (attrition bias) All outcomes	Low risk	Losses fully reported; ITT analyses conducted
Selective reporting (reporting bias)	Low risk	Outcomes prespecified in trial registry reported

**Table:** Wong 2012

***Study characteristics***		
Methods	Country: Canada Setting: 2 Toronto hospitals Aim: "to determine the effectiveness and safety of a perioperative SC intervention including varenicline and counseling versus placebo and counseling to increase short‐ and long‐term abstinence in surgical patients" Study design: randomised placebo‐controlled double‐blind trial Dates conducted: June 2008‐November 2010	
Participants	286 non‐cardiac elective surgery patients, smoking 10+ CPD, no abstinence > 3 months in last year, scheduled for surgery in the next 8‐30 days. Allocated to varenicline (151) or placebo (135). Mean age 52.6, 47% women, mean CPD 17.4, mean FTND 4.8	
Interventions	Varenicline: 12 weeks standard regimen, 1st week titrated Placebo: Identical‐looking tablets and regimen All participants received 2 standardised 15‐min counselling sessions by researchers, 1 in pre‐op clinic and 1 24 h after surgery, supplemented by written materials. All participants retained the same counsellor throughout the process Weekly counselling phone calls for 4 weeks, and at the end of 8 weeks. From 3‐12 months, phone calls every 4 weeks for smoking status, nicotine dependence, stage of change, CPD, brief (< 5 min) counselling. TQD was set for 24 h before surgery, and medication begun 7 days before TQD Participants were invited to visit the hospital at 3, 6, and 12 months, for assessment and testing. Participants unable to visit the hospital were sent a self‐test urinary kit	
Outcomes	7‐day PPA at 12 months; abstinence on TQD; 7‐day PPA at 3 months and 6 months. Self‐reported changes in CPD and stage of change at 3, 6 and 12 months Validation: expired CO and urinary cotinine (cut‐offs not given)	
Notes	Supported by Canadian academic institutes and Pfizer Canada New for 2016 update Author declaration of interests: "Dr. Chapman holds the GSK‐CIHR Research Chair in Respiratory Health Care Delivery. Dr. Wong is a recipient of a Merit Research Award from the Department of Anesthesia at the University of Toronto, Toronto, Ontario, Canada."	
***Risk of bias***		
**Bias**	**Authors' judgement**	**Support for judgement**
Random sequence generation (selection bias)	Low risk	"Smokers were randomly assigned to receive varenicline (Pfizer Inc., Kirkland, Quebec, Canada) or matching placebo using a computer‐generated randomization list at each center. A stratified randomization with blocks of 40, based on the smoker’s stage of change, was employed because the stage of change may predict successful abstinence from smoking."
Allocation concealment (selection bias)	Low risk	"The patient assignments were placed into sequentially numbered, opaque sealed envelopes, and were kept by an independent research pharmacist at each center who was not involved with patient care or outcome assessments. For each patient, the research pharmacist opened the envelope and provided the research coordinator with the medication or placebo (lactose, identical in appearance) according to the randomization schedule."
Blinding (performance bias and detection bias) All outcomes	Low risk	"The patients, healthcare personnel, and research staff were blinded to the randomization throughout the study period."
Incomplete outcome data (attrition bias) All outcomes	Low risk	Losses fully reported: varenicline: 6 discontinued treatment, 11 discontinued follow‐up. Placebo: 6 discontinued treatment, 10 discontinued follow‐up. ITT analyses conducted
Selective reporting (reporting bias)	Low risk	Outcomes prespecified in trial registry reported

**Table:** Yang 2016

***Study characteristics***		
Methods	Country: China Setting: hospital Study design: parallel randomised trial	
Participants	78 male smokers, motivated to quit, with a diagnosis of COPD Mean age 58, baseline average CPD 5	
Interventions	No medication Varenicline 2 x 1 mg/day	
Outcomes	PA at 24 weeks. Self‐reported	
Notes	New for 2022 update Study funding: not reported Author declaration of interests: not reported	
***Risk of bias***		
**Bias**	**Authors' judgement**	**Support for judgement**
Random sequence generation (selection bias)	Unclear risk	Stated randomised but methods not reported
Allocation concealment (selection bias)	Unclear risk	Concealment not reported
Blinding (performance bias and detection bias) All outcomes	High risk	Not placebo‐controlled
Incomplete outcome data (attrition bias) All outcomes	Unclear risk	Attrition rate unclear
Selective reporting (reporting bias)	Unclear risk	Protocol unavailable

**Table:** Zawertailo 2020

***Study characteristics***		
Methods	Country: Canada Setting: inpatient or outpatient treatment clinic Aim: to evaluate if varenicline would be a safe and efficacious treatment for tobacco dependence in people with alcohol dependence Study design: randomised, placebo‐controlled, double‐blind pilot study	
Participants	31 daily dependent smokers, aged 18‐65 years, also in treatment for alcohol dependence. Eligible participants smoked at least 10 CPD; and scored ≥ 3 on the Fagerstrom Test of Nicotine Dependence (FTND). 26.7% were female, mean age 44.6, baseline average CPD 18.4 Exclusion criteria: serious medical condition requiring immediate investigation or treatment, current pregnancy or breastfeeding, current psychiatric disorder excluding alcohol use disorder and tobacco dependence, any known contraindication to using varenicline, inability to provide informed written consent, or inability or unwillingness to attend weekly study visits for 12 consecutive weeks	
Interventions	Varenicline 2 x 1.5 mg/day (first week titrated) Placebo All participants received weekly individual in‐person SC counselling for 12 weeks	
Outcomes	Primary: abstinence from smoking (defined as no smoking during the last 4 weeks) at the EoT. Self‐reported abstinence was biochemically verified using exhaled CO of < 10 ppm Secondary: abstinence from smoking at 6 months, biochemically confirmed using exhaled CO of < 10 ppm	
Notes	New for 2022 update Funding by Pfizer and the Ministry of Health, the Canadian Institutes of Health Research Author declaration of interests: "L.Z. is currently receiving investigator‐initiated funding from Pfizer, Inc, and peer‐reviewed grant funding from the Ontario Ministry of Health and the Canadian Institutes of Health Research. B.L.F. has obtained funding from Pfizer (GRAND Awards, including salary support) for investigator‐initiated projects. P.S. has received funding from the Ontario Ministry of Health, Canadian Institutes of Health Research, Canadian Centre on Substance Abuse, Public Health Agency of Canada, Pfizer, Inc/Canada, Ontario Lung Association, and Canadian Cancer Society Research Institute. He has received honoraria for speaking engagements and consulting from Pfizer Canada, Inc, ABBVie, Bristol‐Myers Squibb, Evidera, Inc, Johnson & Johnson Group of Companies, Medcan Clinic, Inflexxion, Inc, V‐CC Systems, Inc, MedPlan Communications, Kataka Medical Communications, Miller Medical Communications, NVision Insight Group, Sun Life Financial, and Myelin & Associates For the remaining authors, no conflicts of interest are declared."	
***Risk of bias***		
**Bias**	**Authors' judgement**	**Support for judgement**
Random sequence generation (selection bias)	Low risk	"Computer generated by the CAMH Research Pharmacy",
Allocation concealment (selection bias)	Low risk	"Both participants and study staff were blinded to the randomization assignment" with allocation performed by the CAMH Research Pharmac, who were not involved in recruitment.
Blinding (performance bias and detection bias) All outcomes	Low risk	"Both participants and study staff were blinded to the randomization assignment." "Self‐reported abstinence at each weekly visit was confirmed with expired CO of less than 10 ppm using a Bedfont Smokelyzer."
Incomplete outcome data (attrition bias) All outcomes	Low risk	5/15 (placebo) and 4/16 (varenicline) dropped out. 21/31 participants attended 6 months follow‐up
Selective reporting (reporting bias)	Low risk	Pre‐specified outcomes reported

**Table:** Zhang 2022

***Study characteristics***		
Methods	Study design: RCT Country: Canada Recruitment method (quote): "The primary method of recruitment was by word of mouth and Facebook advertisement. Interested participants were directed to the study website, where they could indicate their consent for participation and complete eligibility questionnaires"	
Participants	2461 smokers; 56% female, average age 46.5, 46.9% of bupropion group and 44.9% of varenicline group smoked 11‐20 CPD	
Interventions	Bupropion 150 mg once daily for first 3 days, then twice daily for the remainder of 12 weeks. Starting 7 days prior to TQD Varenicline 0.5 mg once daily for first 3 days, then 0.5 mg twice daily for next 4 days, then 1 mg twice daily for the remainder of 12 weeks. Starting 7 days prior to TQD Common components: weekly motivational emails	
Outcomes	SC: point prevalence measured at 52 weeks. Not biochemically verified AEs: measured at 12 weeks	
Notes	Study funding: "This research was funded by Global Research Awards for Nicotine Dependence (GRAND), a peer‐reviewed research grant competition funded by Pfizer Pharmaceuticals [Zawertailo (GRAND2012) WS2391913]. The study’s funder had no role in study design, data collection, data analysis, data interpretation, or writing of the report, and had no decision to submit the article for publication. Dr. Le Foll is supported by a clinician‐scientist award from the Department of Family and Community Medicine and by the Addiction Psychiatry Chair of the Department of Psychiatry of University of Toronto. Dr. Selby is supported by a clinician‐scientist award from the Department of Family and Community Medicine and CAMH." Author declaration of interests: "All authors have completed the ICMJE uniform disclosure form at www.icmje.org/coi_disclosure.pdf and declare: no support from any organization for the submitted work; PS reports receiving funding and/ or honoraria from Pfizer Inc./Canada, Shoppers Drug Mart, Bhasin Consulting Fund Inc., Patient‐Centered Outcomes Research Institute, ABBVie, and Bristol‐Myers Squibb; BLF and LZ both receive support from Pfizer Global Research Awards in Nicotine Dependence (GRAND) Award Program; there are no other relationships or activities that could appear to have influenced the submitted work. BLF also reports grants from Brainsway, grants from Bioprojet, grants from Alkermes, grants from Canopy, grants from ACS, non‐financial support from Aurora, outside the submitted work."	
***Risk of bias***		
**Bias**	**Authors' judgement**	**Support for judgement**
Random sequence generation (selection bias)	Low risk	Study quote: "Participants were randomly assigned to one of two medication arms (varenicline or bupropion) using permuted‐block randomization in a 1:1 ratio in blocks of 100." Protocol quote: "The randomization process will be computerized."
Allocation concealment (selection bias)	Low risk	Protocol quote: "The randomization process will be computerized."
Blinding (performance bias and detection bias) All outcomes	Low risk	Study quote: "Participants were not blinded to treatment since their health care provider signing the prescription form was required to know which drug was being prescribed to their patient." However, both study groups received active smoking cessation pharmacotherapy treatment. The abstinence outcome was not biochemically verified, however, study arms received the same behavioural support.
Incomplete outcome data (attrition bias) All outcomes	Low risk	The overall number of participants lost at the 52‐week follow‐up was > 50% in each condition. However, a complete case sensitivity analysis (section 3.5.2) did not change findings at the 52‐week follow‐up (i.e. no difference between conditions)
Selective reporting (reporting bias)	Unclear risk	Not all prespecified outcomes from trial registry reported in main results paper. However, SC outcomes are reported.

**Table:** Zincir 2013

***Study characteristics***		
Methods	Study design: RCT Country: Turkey Setting: outpatient SC clinic in a hospital Recruitment method: patients who presented at the SC outpatient clinic were included in the study on a voluntary basis	
Participants	300 participants randomised; average age: 45.8 in those who stopped smoking and 40.8 in those who continued smoking; average boxes of cigarettes per year: 23.62 in those who stopped smoking and 23.26 in those who continued smoking; mean FTND: 5.9 in those who stopped smoking and 6.7 in those who continued smoking	
Interventions	Bupropion 150 mg/day, started a week before the quit day and continued from day 1‐3, raised to 300 mg daily on day 4, with this dose maintained until the end of week 12 Varenicline 0.5 mg daily, raised to 1 mg daily at day 4, then to 2 mg daily at day 8, with this dose maintained until the end of week 12 NRT: administered using either a nicotine patch or nicotine gum, or a combination of both. Nicotine patches were used in their 3 forms containing 21, 14 and 7 mg of nicotine, and in cases of excessive nicotine craving, 2 mg nicotine gum was used. For each dose of nicotine patches, 4 weeks of administration in decreasing doses was recommended. The nicotine gum was started between 12 and 24 doses (2 mg) a day and gradually decreased.	
Outcomes	SC: definition not specified; measured at 24‐28 weeks from study start AEs: measured for unspecified period	
Notes	Funding: not reported Author declaration of interests: not reported	
***Risk of bias***		
**Bias**	**Authors' judgement**	**Support for judgement**
Random sequence generation (selection bias)	Unclear risk	Quote: “…they were randomized to the pharmacological therapy groups” Comment: no further information given
Allocation concealment (selection bias)	Unclear risk	Quote: “…they were randomized to the pharmacological therapy groups" Comment: no further information given
Blinding (performance bias and detection bias) All outcomes	High risk	Quote: “This was a naturalistic clinical follow‐up study." Comment: those involved in the study were therefore unblinded
Incomplete outcome data (attrition bias) All outcomes	Unclear risk	300 participants were randomised and 251 completed the study. Therefore 49/300 (16.3%) were lost to follow‐up overall. However, it is impossible to establish the number lost to follow‐up by group.
Selective reporting (reporting bias)	Unclear risk	Protocol unavailable
Other bias	High risk	Quote: “no adverse event was reported during the study”. This is highly unlikely to be correct when considering standard definitions of AEs. There is no explanation of how AEs were assessed in this study. In addition, the wording of the paper makes the final follow‐up slightly unclear. After discussion we judged final follow‐up to be 24‐28 weeks from study start, although quit rates are higher than would be expected at this time point.

**AE:** adverse event; **BMI:** Body Mass Index (kg/m^2^); **CA:** continuous abstinence; **CAR**: Continuous Abstinence Rate; **cNRT:** combined nicotine replacement therapy; **CO:** carbon monoxide; **COPD:** chronic obstructive pulmonary disease; **CPD:** cigarettes per day; **CQR:** continuous quit rate; **CVD:** cardiovascular disease; **DSM‐IV:***Diagnostic and Statistical Manual of Mental Disorders, 4th Edition*; **ECG:** electrocardiogram; **EoT:** end of treatment; **FTND:** Fagerström Test for Nicotine Dependence; **ITT:** intention‐to‐treat; **LOCF:** last observation carried forward; **MA:** meta‐analysis; **MDD:** major depressive disorder; **MI:** motivational interviewing; **MIMS:** Monthly Index of Medical Specialities; **mCEQ:** Modified Cigarette Evaluation Questionnaire; **MNWS:** Minnesota Nicotine Withdrawal Scale; **NCT:** National Clinical Trials (ClincialTrials.gov registry); **NRT:** nicotine replacement therapy; **PA:** prolonged abstinence; **PPA:** point‐prevalence abstinence; **ppm:** parts per million; **QoL:** quality of life; **QSU‐brief:** Brief Questionnaire of Smoking Urges; **RCT:** randomised controlled trial; **SAE:** serious adverse event; **SC:** smoking cessation; **TQD:** target quit date

### Characteristics of excluded studies [ordered by study ID]

**Table:**

Study	Reason for exclusion
Brandon 2011	Short‐term (15 days) RCT, to test craving and psychological reward; cessation was not an outcome. Previously included but excluded for 2023 update
Bullen 2018	Study tested effect of e‐cigarettes as an adjunct to varenicline
Burstein 2006	RCT of tolerability and safety of varenicline in 24 elderly (≥ 65) smokers for 1 week. Not a cessation trial
Chantix 2006	Tested NRT alone or varenicline + NRT for 12 days to test safety and side effects of co‐administration. Not a cessation trial
Cui 2012	Open‐label non‐randomised pre/post study of 36 HIV+ participants; all received varenicline
Dezee 2013	RCT in which all participants were given varenicline; intervention tested was in‐person vs internet counselling
Dutra 2012	53 participants with schizophrenia given varenicline + cognitive behavioural therapy. Abstinence assessed at 12 weeks (end of treatment)
Ebbert 2009a	Open‐label, single‐arm Phase II study, for safety and efficacy of varenicline plus bupropion
Ebbert 2009b	Cohort analysis of 104 participants on varenicline + NRT and 135 participants treated prior to release of varenicline (93% used NRT)
Ebbert 2011b	Pilot study of varenicline for smokeless tobacco users. 12‐week outcome (EoT) reported, not long‐term post‐treatment. Previously included but excluded for 2023 update
Ebbert 2014	RCT in which all participants were given varenicline; the intervention being tested was bupropion vs placebo. See also Hong 2011
Evins 2014	Relapse prevention study that recruited already abstinence participants. Previously included but excluded for 2023 update
Faessel 2009	Outcomes were safety, tolerability and pharmacokinetics, not smoking cessation. Previously included but excluded for 2023 update
Fagerström 2010	431 smokeless tobacco users in Norway and Sweden, randomised to varenicline or placebo; CAR assessed at 12 and 26 weeks. Previously included but excluded for 2023 update
Falk 2014	Varenicline was used for alcohol reduction, not for smoking
Fatemi 2013	3‐arm RCT of varenicline, bupropion and placebo; only assessed to end of treatment (12 weeks).
Ferketich 2012	Pilot study of varenicline vs NRT; participants could choose their treatment; intervention being tested was the addition of a lung cancer screening programme
Ferketich 2013	Safety of varenicline among smokers enrolled in the Lung HIV study. Participants could choose varenicline or NRT, and were only followed for 3 months.
Fertig 2015	Study testing effect of varenicline on alcohol dependence
Frye 2013	Participants followed only until EoT (12 weeks)
Fucito 2011	Primary outcome was effects on drinking behaviour. Smoking status only measured at end of study (8 weeks)
Garza 2011	110 abstinent smokers treated with varenicline or placebo, to assess incidence and severity of neuropsychiatric symptoms; not a cessation trial. Previously included but excluded for 2023 update
Granatowicz 1976	No control group
Gray 2012	Pilot study of varenicline vs bupropion in older adolescents; outcome was reduction rather than cessation, and participants were only followed for 3 months.
Hajek 2011	101 smokers randomised to preloaded varenicline or placebo; abstinence not measured beyond 12 weeks
Hajek 2013	All were given varenicline, with the intervention tested being the addition of a NRT patch. Only followed to 3 months
Hajek 2015	Follow‐up under 6 months. Previously included but excluded for 2023 update
Hartwell 2014	Varenicline for drinking and smoking; smoking topography and pharmacogenetics rather than smoking cessation
Hawk 2012	RCT of extended pre‐TQD varenicline vs standard regimen; all participants got varenicline, and were followed only until end of treatment (12 weeks)
Hong 2011	Secondary analysis to Ebbert 2014, looking at depression in recipients of varenicline + bupropion vs varenicline alone
Hoogsteder 2014	All participants were given open‐label varenicline; the intervention being tested was the addition of NicVAX.
Hsueh 2014	Open‐label cohort study of smokers taking varenicline or NRT
Hsueh 2021	Study not randomized. Participants chose their own treatment.
Hughes 2011	218 smokers not ready to quit assigned to varenicline or placebo for 2‐8 weeks for cigarette reduction. Previously included but excluded for 2023 update
IRCT20100127003210N	Study not randomised
Jain 2014	RCT of smokeless tobacco users. Follow‐up of 12 weeks
Jennings 2014	Only followed to 16 weeks
Jiménez‐Ruiz 2013	Not an RCT
Kempe 1967	Observational uncontrolled study
Koegelenberg 2014	All participants took varenicline; the intervention being tested was the addition of NRT.
Maliszewski 1972	Uncontrolled study of 14 smokers on a 25‐day course of cytisine (Tabex); followed up for 2 weeks
Marakulin 1984	Follow‐up 6 weeks
McClure 2013	Laboratory study following an RCT of varenicline in a programmed lapse; abstinence only to 4 weeks. Previously included but excluded for 2023 update
McColl 2008	RCT of varenicline's potential as an abuse drug in smokers and non‐smokers; not a smoking cessation trial
McNaughton 2013	All participants received varenicline; the intervention being tested, as a relapse prevention aid, was interactive voice response phone calls
Meszaros 2013	Pilot study (10 participants, only 4 completers), only followed to 3 months; objective was reduction, not cessation. Previously included but excluded for 2023 update
Metelitsa 1987	Uncontrolled study
Mitchell 2012	Varenicline was for drinking reduction, not smoking cessation; only followed for 12 weeks. Previously included but excluded for 2023 update
Mocking 2013	7‐day administration of varenicline for emotional and cognitive processing in non‐smokers
Monova 2004	Follow‐up was 60 days
Nahvi 2014b	Follow‐up under 6 months
Nahvi 2020	Study tested an intervention for improving adherence to varenicline
NCT00387946	Follow‐up under 6 months
NCT00502216	Study of varenicline and naltrexone for tolerability and weight gain in smokers, not cessation
NCT00554840	Follow‐up under 6 months
NCT00828113	Relapse prevention study that recruited already abstinent participants. Previously included but excluded for 2023 update
NCT01093937	Follow‐up under 6 months
NCT01413516	Follow‐up under 6 months
NCT01532232	Study terminated
NCT01574703	Tobacco abstinence not measured
NCT01592695	Different levels of behavioural support between study arms
NCT01639560	Follow‐up under 6 months
NCT01771627	Follow‐up under 6 months
NCT01772641	Follow‐up under 6 months
NCT01806779	All participants got varenicline; the addition of bupropion was the intervention being tested.
NCT01892813	Different levels of behavioural support between study arms
NCT02048917	Follow‐up under 6 months
NCT02147132	Cross‐over trial
NCT02271919	Follow‐up under 6 months
NCT02501265	Study testing the addition of bupropion to a subset of participants receiving varenicline
NCT03709823	Follow‐up under 6 months
Nides 2021	Follow‐up under 6 months
Nollen 2011	All participants received varenicline, but half received extended counselling and half a single session. Cessation only measured to 3 month end point
Ostrovskaia 1994	Uncontrolled study
Park 2011	RCT of 49 smokers with lung cancer randomised to varenicline or placebo; follow‐up only for 12 weeks to EoT
Patterson 2010	Short‐term (3‐week) study of propensity to relapse with working memory deficits after 10 days of varenicline
Paun 1968	Controlled trial of cytisine (Tabex) (366 smokers) vs placebo (239 smokers) but followed only for 8 weeks. Observational study of 230 cytisine‐users followed for 26 weeks, but no comparator group
Pfeifer 2019	Did not measure smoking cessation as an outcome
Pfizer 2006	Follow‐up under 6 months
Poling 2010	RCT of varenicline in 31 methadone‐maintained smokers; trial lasted 3 months, and reduction was an outcome of interest (only 3‐month abstinence was reported)
Ramon 2014	RCT in which all participants received varenicline; intervention being tested was the addition of NRT
Rose 2014	RCT of varenicline versus varenicline + bupropion, in smokers who had failed to quit on NRT. All got varenicline
Schlienz 2014	4 weeks treatment with varenicline; outcome was impact on behavioural economic indices, not smoking cessation
Schmidt 1974	Non‐randomised trial
Schnoll 2011	RCT of open‐label varenicline + counselling; intervention being tested was recruitment strategies, not smoking cessation
Shim 2011	60 smokers with schizophrenia randomised to varenicline or placebo for 8 weeks; assessment at end of treatment, reduction but not abstinence rates reported
Sicras‐Mainar 2010	Multicentre observational non‐randomised non‐controlled study
Smith 2013	Follow‐up under 6 months
Stapleton 2008	Non‐randomised trial
Stoyanov 1972	Observational study with no comparator group and short but unstated length of follow‐up
Swan 2010	All participants were given varenicline (treated as an included study for 2012 update)
Tonstad 2006	Relapse prevention study that recruited already abstinent participants. Previously included but excluded for 2023 update
Tønnesen 2013	Relapse prevention study that recruited already abstinent participants. Previously included but excluded for 2023 update.
Weiner 2011	Follow‐up under 6 months
Zatonski 2006	Uncontrolled observational study

**CAR**: Continuous Abstinence Rate; **EoT:** end of treatment; **NRT:** nicotine replacement therapy; **RCT:** randomised controlled trial; **TQD:** target quit date

### Characteristics of studies awaiting classification [ordered by study ID]

**Table:** Wiratmoko 2013

Methods	
Participants	
Interventions	
Outcomes	
Notes	Abstract only; further details awaited

**Table:** Yujie 2014

Methods	
Participants	
Interventions	
Outcomes	
Notes	Abstract only; further details awaited

### Characteristics of ongoing studies [ordered by study ID]

**Table:** Berlin 2019

Study name	Randomised trial of electronic cigarettes with or without nicotine in smoking cessation (ECSMOKE)
Methods	Randomised, placebo‐controlled, double‐blind, double‐dummy, multicentre, parallel‐group trial
Participants	At least 650 adults smoking at least 10 CPD in the past year, motivated to quit, aged 18‐70
Interventions	e‐cigarette without nicotine plus placebo tablets of varenicline e‐cigarette with nicotine (12 mg/mL) plus placebo tablets of varenicline e‐cigarette without nicotine plus 0.5 mg varenicline tablets
Outcomes	Continuous smoking abstinence rate during the last 4 weeks of the treatment period. Validated by self‐report and expired CO < 8 ppm at 12 and 24 weeks after treatment initiation
Starting date	17 October 2018
Contact information	Ivan Berlin, +33142161678, ivan.berlin@aphp.fr
Notes	Study funding: Programme Hospitalier de Recherche Clinique (PHRC) National 2015, Ministry of Health, France

**Table:** Caponnetto 2019

Study name	Efficacy of smoking cessation with varenicline plus counselling for e‐cigarettes users (VAREVAPE)
Methods	Randomised, double‐blind, placebo‐controlled, parallel 2‐group trial
Participants	140 participants that exclusively smoke e‐cigarettes daily for > 3 months 140 'dual users' that use e‐cigarettes daily for > 3 months, who also smoke at least one combustible CPD All motivated to quit
Interventions	Counselling plus varenicline 1 mg twice a day Counselling plus matched placebo
Outcomes	Continuous smoking abstinence rate between weeks 9‐24. Validated by self‐report and expired CO < 10 ppm, and saliva cotinine levels < 7 ng/mL
Starting date	June 2019
Contact information	Pasquale Caponnetto: p.caponnetto@unict.it
Notes	Study funding: Pfizer, GRAND, Global Research Award for Nicotine Dependence

**Table:** ChiCTR1900021400

Study name	Individual tobacco cessation research based on nicotine metabolite ratio in smoking patients with chronic obstructive pulmonary disease: a randomized controlled trial
Methods	Parallel‐group RCT
Participants	224 participants aged 18‐85 years who meet the diagnostic criteria for both COPD and tobacco dependency, who have been smoking for > 5 years, with at least 10 CPD for the past 12 months. Exhaled CO > 10 ppm
Interventions	Varenicline Bupropion
Outcomes	Smoking abstinence rate. Nicotine metabolite ratio
Starting date	18 February 2019
Contact information	Xiao Dan, +86138111374263, danxiao@263.net
Notes	Study funding: The China‐Japan Friendship Hospital

**Table:** IRCT20200719048133N1

Study name	Efficacy of varenicline for smoking cessation among persistent smokers after coronary artery revascularization: a randomized placebo controlled clinical trial
Methods	Double‐blind, randomised, placebo‐controlled trial
Participants	600 participants that are male, > 18 years, and a current daily smoker of ≥ 5 CPD. Must also have a history of coronary artery revascularisation 9‐12 months prior to the study
Interventions	Varenicline 0.5 mg once a day for 3 days, 0.5 mg twice a day for 4 days, 1 mg twice a day for 12 weeks Placebo, following same regimen
Outcomes	Continuous smoking abstinence rate at the end of 26 weeks' follow‐up, by self‐report and validated by a close relative
Starting date	22 August 2020
Contact information	Masoumeh Lotfi Tokaldany, +982188029256, lotfi213366@yahoo.co.uk
Notes	Study funding: Tehran University of Medical Sciences

**Table:** Lawson 2021

Study name	Extended pre‐quit varenicline to assist in quitting smoking (EVarQuit)
Methods	Parallel group, placebo‐controlled RCT
Participants	320 participants, smoking at least 10 CPD for the past 6 months, expired CO > 7 ppm, motivated to quit
Interventions	Extended run‐in group: 4 weeks of varenicline Standard run‐in group (comparator): 3 weeks placebo, plus 1 week of varenicline Both groups will receive 11 weeks of varenicline post run‐in, after their target quit date
Outcomes	Continous smoking abstinence at 12 and 26 weeks post‐quit date, self‐report but bioverified
Starting date	01 October 2017
Contact information	Larry Hawk, lhawk@buffalo.edu
Notes	Study funding: state University of New York at Buffalo, National Cancer Institute, Pfizer

**Table:** NCT00906386

Study name	Methadone maintenance treatment and smoking cessation (MMTASC)
Methods	Randomised, double‐blind, placebo‐controlled trial
Participants	112 people who smoke and are on methadone maintenance for opioid dependence
Interventions	Varenicline 1.0 mg x 2/day vs placebo for 12 weeks
Outcomes	7‐day PPA at 26 weeks
Starting date	May 2009
Contact information	Milan Khara
Notes	Current status unknown

**Table:** NCT01243203

Study name	Smoking cessation program in the preadmission clinic: the use of a teachable moment
Methods	Double‐blind, parallel‐group RCT
Participants	300 adults scheduled for elective surgery, aged 18+, smoking 10+ CPD
Interventions	Varenicline vs placebo
Outcomes	Abstinence at 24 and 52 weeks
Starting date	November 2007
Contact information	Francis Chung
Notes	Study funding: Pfizer

**Table:** NCT01312909

Study name	Smoking cessation study in healthy adolescent smokers
Methods	Phase IV randomised, triple‐blind, placebo‐controlled trial
Participants	300 healthy adolescents (12‐19 years) smoking at least 5 CPD, with at least 1 failed quit attempt
Interventions	Varenicline 1 mg x 2/day vs varenicline 0.5 mg x 2/day vs placebo
Outcomes	CA at weeks 9‐12, 9‐24, 9‐52; 7‐day PPA at weeks 12, 24, 52; reduction in CPD
Starting date	April 2011
Contact information	Pfizer Inc
Notes	Study funding: Pfizer

**Table:** NCT01800019

Study name	The Canadian HIV Quit Smoking Trial: tackling the co‐morbidities of depression and cardiovascular disease in HIV+ smokers (CANQUIT)
Methods	Open‐label, 4‐arm, factorial RCT
Participants	256 adults who are HIV+ and smoke, 5+ CPD, willing to set a quit date
Interventions	NRT NRT + HIV‐tailored quit smoking counselling Varenicline Varenicline + HIV‐tailored quit smoking counselling
Outcomes	7‐day PPA and 4‐week CA at week 48, CO‐verified
Starting date	January 2014
Contact information	Louise Balfour, PhD, Ottawa Hospital Research Institute
Notes	Study funding: CIHR Research Operating Grant

**Table:** NCT02106637

Study name	Early in‐hospital initiation of pharmacotherapy for smoking cessation, patients after ACS
Methods	Double‐blind, parallel‐group RCT
Participants	300 adult smokers with ACS
Interventions	Varenicline vs placebo
Outcomes	CA at 1 month, 6 months, 1 year after hospitalisation; SAE rate
Starting date	June 2014
Contact information	Ilan Goldenberg, MD, Ilan.Goldenberg@sheba.health.gov.il
Notes

**Table:** NCT02162849

Study name	Reward sensitivity and pharmacotherapy for smoking cessation
Methods	Double‐blind, parallel‐group RCT
Participants	90 adults who smoke, 5+ CPD
Interventions	Varenicline + placebo patch vs nicotine patch + placebo tablet; all get behavioural counselling
Outcomes	CA at EoT, 3 months and 6 months
Starting date	April 2015
Contact information	Paul Cinciripini
Notes

**Table:** NCT02378714

Study name	Behavioral activation and varenicline for smoking cessation in depressed smokers
Methods	Parallel‐group, placebo‐controlled RCT
Participants	Adults who smoke with MDD
Interventions	Standard behavioral smoking cessation treatment plus placebo varenicline Behavioral activation for smoking cessation plus placebo varenicline Standard behavioral smoking cessation treatment plus active varenicline Behavioral activation for smoking cessation plus active varenicline
Outcomes	Bioverified PPA at 27 weeks; AE and SAE rates
Starting date	24 July 2015
Contact information	Brian Hitsman, Ph.D. Northwestern University
Notes	Study funding: 1R01CA184211‐01A1 (U.S. NIH Grant/Contract)

**Table:** NCT02460900

Study name	Optimizing smoking cessation for people with HIV/AIDS who smoke
Methods	Randomised factorial trial
Participants	Adults who smoke and have HIV/AIDS
Interventions	Varenicline and standard care Placebo and standard care Positively Smoke Free and placebo Positively Smoke Free and varenicline
Outcomes	7‐day PPA at 24 weeks
Starting date	July 2016
Contact information	Principal Investigators: Seth Himelhoch, MD, MPH and Deanna Kelly, PharmD, University of Maryland School of Medicine
Notes

**Table:** NCT02856581

Study name	Reducing surgical complications in patients with newly diagnosed lung cancer who smoke cigarettes
Methods	Parallel group, placebo‐group, phase III, RCT
Participants	23 participants, aged > 18, with a new diagnosis of lung cancer with surgery scheduled 10 days‐12 weeks after randomisation. Have smoked daily/nearly every day for previous 6 months, and at least 1 puff in the previous 7 days. Motivated to quit
Interventions	Varenicline for 12 weeks, plus behavioural intervention Placebo for 12 weeks, plus behavioural intervention
Outcomes	Primary outcomes: mortality, rehospitalisation, incidence of pulmonary complications, incidence of intensive care unit admissions, incidence of unspecific wound infection, incidence of anastomotic failure Secondary outcomes: change in QoL, length of hospital stay, smoking abstinence rates as measured by self‐report and salivary cotinine testing
Starting date	29 September 2017
Contact information	Ivana Croghan, ivana.croghan@mayo.adu
Notes	Study funding: Alliance for Clinical Trials in Oncology, National Cancer institute, Pfizer

**Table:** NCT02991781

Study name	Combined bio‐ and neuro‐ feedback vs. varenicline use for smoking cessation
Methods	RCT
Participants	Unemployed adults < 35 who smoke (subgroup of a larger trial population)
Interventions	Biofeedback and neurofeedback training Varenicline use for smoking cessation Sham neurofeedback Passive control
Outcomes	Abstinence at 2 years
Starting date	January 2017
Contact information	Principal Investigator: Panos Bamidis, Ass. Prof, Medical School, Aristotle University of Thessaloniki
Notes

**Table:** NCT03365362

Study name	Achieving smoking cessation milestones in opioid treatment patients: a randomised 2x2 factorial trial of directly observed and long‐term varenicline
Methods	2 x 2 factorial, placebo‐controlled, RCT
Participants	450 participants, aged > 18, currently smoking at least 5 CPD and > 100 in their lifetime, motivated to quit, receiving methadone or buprenorphine in an opioid treatment programme > 3 months
Interventions	Long‐term varenicline ‐ 24 weeks of standard dose Short‐term varenicline ‐ 12 weeks of standard dose, then matched placebo weeks 13‐24 Directly observed therapy ‐ varenicline from opioid treatment programme nurses at the same time as methadone Self‐administered varenicline
Outcomes	Primary outcomes: initial abstinence (period of > 24 h smoking abstinence), time to lapse, time to relapse (7 consecutive days of self‐reported smoking) Secondary outcomes: durability of tobacco abstinence, validated by exhaled CO at week 28 and week 52, cigarettes smoked/day, nicotine dependence, QoL
Starting date	25 October 2018
Contact information	Shadi Nahvi, snahvi@montefiore.org
Notes	Study funding: Albert Einstein College of Medicine, NIH, Pfizer, National Institute on Drug Abuse

**Table:** NCT03557294

Study name	Varenicline OTC trial on efficacy and safety (VOTC)
Methods	Parallel‐group, placebo‐controlled, RCT
Participants	307 participants, aged ≥ 21, daily smoking with exhaled CO > 10 ppm or positive urine cotinine, motivated to quit
Interventions	Days 1‐3, 0.5 mg varenicline once daily; days 4‐7, 0.5 mg twice daily; day 8 to end, 1 mg twice daily Days 1‐3, 0.5 mg varenicline once daily; day 4 to end: 0.5 mg twice daily 0.0 mg placebo comparator
Outcomes	Smoking abstinence, validated by self‐report and exhaled CO
Starting date	07 May 2018
Contact information	Not reported
Notes	Study funding: Arizona State University, National Institute on Drug abuse, Los Angeles Clinical Trials, University of Nevada, Reno, Pfizer

**Table:** NCT04011280

Study name	Novel pharmacotherapy approaches in smokers with serious mental illness
Methods	Phase IV, double‐blind, randomised, parallel‐group RCT
Participants	60 participants, 18‐70 years, with a diagnosis of bipolar disorder or schizophrenia spectrum disorder, smoke > 10 CPD, exhaled CO > 10 ppm, motivated to quit
Interventions	Low‐dose varenicline: 0.5 mg twice daily with 0.5 mg daily titration over 1 week Standard‐dose varenicline: 1.0 mg twice daily with standard titration
Outcomes	Primary: feasibility of combining ACT (Acceptance and Commitment Therapy) with different varenicline regimens Secondary: nicotine‐metabolite ratio
Starting date	15 July 2019
Contact information	Benjamin McKenna, bmckenna@ucsd.edu
Notes	Study funding: University of California, San Diego, Veterans Medical Research Foundation, University of California

**Table:** NCT04015414

Study name	Varenicline versus cytisine for smoking cessation in the primary care setting in Croatia and Slovenia ‐ a randomised controlled trial
Methods	Parallel‐group, RCT
Participants	380 participants, > 18 years, currently smoking, motivated to quit
Interventions	Varenicline ‐ starting 1 week prior to patient's target quit date Cytisine ‐ following standard manufacturer's dosing protocol
Outcomes	Self‐reported 7‐day abstinence from tobacco (primary outcome: 12‐weeks following TQD)
Starting date	14 July 2020
Contact information	Stjepan Oreskovin, +385912858247, sooreskov@gmail.com Jeffrey Ashburner, 617/724‐3828, jasburner@mgh.harvard.edu
Notes	Study funding: University of Zagreb, University of Ljubliana School of Medicine, University of Zagreb School of Medicine, Harvard Medical School

**Table:** NCT04188873

Study name	Optimized chronic care for smokers: developing and implementing integrated clinical systems interventions in primary care ‐ cessation trial
Methods	2x2x2x2 factorial design, RCT
Participants	608 participants, > 18 years, smoking > 4 CPD for the previous 6 months
Interventions	Factorial trial with the following arms: 12‐week varenicline with minimal counselling 12‐week varenicline with 4‐week preparation varenicline and minimal counselling 24‐week varenicline with minimal counselling 24‐week varenicline with 4‐week preparation varenicline and minimal counselling 12‐week varenicline with intensive counselling 12‐week varenicline with 4‐week preparation varenicline and intensive counselling 24 week varenicline with intensive counselling 24‐week varenicline with 4‐week preparation varenicline and intensive counselling 12‐week C‐NRT with minimal counselling 12‐week C‐NRT with 4‐week preparation C‐NRT and minimal counselling 24‐week C‐NRT with minimal counselling 24‐week C‐NRT with 4‐week preparation C‐NRT and minimal counselling 12 week C‐NRT with intensive counselling 12‐week C‐NRT with 4‐week C‐NRT and intensive counselling 24‐week C‐NRT with intensive counselling 24‐week C‐NRT with 4‐week preparation C‐NRT and intensive counselling
Outcomes	Complete 7‐day abstinence ‐ 12‐months post‐TQD, validated by exhaled CO < 5 ppm
Starting date	10 December 2020
Contact information	Megan Piper, 608‐265‐4572, mep@ctri.wisc.edu Stevens Smith, 608‐262‐7563, sss@ctri.wisc.edu
Notes	Study funding: University of Wisconsin, Madison, National Cancer Institute

**Table:** NCT04525755

Study name	A translational randomized clinical trial of varenicline sampling to promote smoking cessation and scalable treatment dissemination
Methods	Parallel‐group, open‐label RCT
Participants	648 participants, > 18 years, daily smoker (25+ days/previous month, and > 5 CPD), smoking for > 1, motivated to quit
Interventions	Varenicline 0.5 mg twice/day, for 28 days NRT, for 28 days No intervention (control)
Outcomes	Primary: 7‐day self‐reported abstinence Secondary: reduction in smoking by 50%, any quit attempts, use of smoking cessation medication
Starting date	08 February 2021
Contact information	Lisa Coles, 843‐876‐2291, colesl@musc.edu Amy Boatright, 843‐876‐2440, boatright@musc.edu
Notes	Study funding: Medical University of South Carolina

**Table:** NCT04604509

Study name	PISCES I: Precision implemented smoking cessation evaluation study
Methods	Parallel‐group, open‐label RCT
Participants	2010 participants, > 18 years, smoking > 5 CPD, motivated to quit
Interventions	Varenicline daily for 6 weeks NRT for 6 weeks Participants continue to receive varenicline as in group I or NRT as in group II for 6 additional weeks Participants switch to a different therapy and receive varenicline or NRT for 6 additional weeks (depending on which group originally assigned to) Higher‐dose varenicline or NRT Varenicline or NRT, plus bupropion Varenicline plus NRT All groups have behavioural counselling
Outcomes	Primary: EoT 7‐day PPA, expired CO, abstinence at 12 weeks
Starting date	04 August 2020
Contact information	Paul Cinciripini, 713‐745‐3822, pcinciri@mdanderson.org
Notes	Study funding: MD Anderson Cancer Center, National Cancer Institute

**Table:** NCT05102123

Study name	PREVENT: PeRiopEratiVE smokiNg cessaTion Trial
Methods	2x2 factorial, RCT
Participants	1720 participants, > 18 years, scheduled to undergo surgery within 28 days, currently smoking (> 100 cigarettes over lifetime and smoke every day in last 30 days)
Interventions	Cytisine and text messaging behavioural support Placebo and text messaging behavioural support Cytisine only Placebo only
Outcomes	Primary: complete abstinence from quit date to 6 months post‐randomisation, verified by exhaled CO < 10 ppm
Starting date	01 April 2022
Contact information	Emily Di Sante, 905‐297‐3479, emily.disante@phri.ca Jessica Vincent, 905‐297‐3479 ext 40635, jessica.vincent@phri.ca
Notes	Study funding: Population Health Research Institute

**Table:** NCT05311085

Study name	Cytisine and e‐cigarettes with supportive text‐messaging for smoking cessation (Cess@Tion)
Methods	RCT
Participants	Adults who smoke
Interventions	12 weeks of cytisine 12 weeks of a nicotine e‐cigarette 12 weeks of cytisine and 12 weeks of a nicotine e‐cigarette
Outcomes	CA at 6 months
Starting date	May 2022
Contact information	Natalie Walker, PhD, n.walker@auckland.ac.nz Chris Bullen, PhD MBChB, c.bullen@auckland.ac.nz
Notes

**Table:** Reid 2010

Study name	Varenicline versus transdermal nicotine patch for smoking cessation in patients with coronary heart disease
Methods	Randomised open‐label trial
Participants	60 adult smokers
Interventions	Varenicline or NRT patch for 12 weeks
Outcomes	CO‐confirmed CA for weeks 12‐26
Starting date	April 2009
Contact information	Robert Reid
Notes

**Table:** Russo 2021

Study name	Efficacy and safety of smoking cessation with varenicline tartrate in diabetic smokers (DIASMOKE)
Methods	Randomised, double‐blind, placebo‐controlled trial
Participants	300 adults with type 2 diabetes, who smoke
Interventions	Varenicline 1 mg x 2/day for 12 weeks vs placebo
Outcomes	CA at week 24 and week 52; AEs
Starting date	June 2011
Contact information	Riccardo Polosa
Notes	Study funding: supported by grant WS5086648 from GRAND (Global Research Award for Nicotine Dependence), an independently reviewed competitive grants programme funded by Pfizer Inc.

**Table:** TCTR20180312001

Study name	Efficacy safety and health‐related quality of life (HRQoL) of cytisine in smoking cessation
Methods	Double‐blind, RCT
Participants	130 participants, 18‐65 years, current smoker > 10 CPD, motivated to quit
Interventions	Cytisine Placebo
Outcomes	PPA rate at 4, 12, 24 and 48 weeks, verified by self‐report and exhaled CO lung function Self‐reported relapse rate QoL
Starting date	01 May 2018
Contact information	Sunee Lertsinudom, 081‐6617237, lsunee@kku.ac.th
Notes	Study funding: Government Pharmaceuticsl Organization, Thailand

**Table:** Tindle 2020

Study name	STudying Partial‐agonists for Ethanol and Tobacco Elimination in Russians with HIV (St PETER HIV)
Methods	Parallel group, placebo‐controlled, RCT
Participants	400 participants with HIV and alcohol dependence, who smoke
Interventions	Varenicline + NRT placebo Varenicline placebo + NRT Cytisine + NRT placebo Cytisine placebo + NRT
Outcomes	Smoking cessation at 12 months Percent heavy drinking days in past 30 days
Starting date	19 July 2017
Contact information	hilary.tindle@vumc.org
Notes	Study funding: "This work was supported by grants from the National Institute on Alcohol Abuse and Alcoholism (NI AAA) in support of URBAN ARCH :U01AA020780 , U24AA020779, U24AA020778 ; and by the Providence/ Boston Center for AIDS Research ( P30AI042853 ) and Tennessee Center for AIDS Research ( P30AI110527 ). The content is solely the responsibility of the authors and does not represent the official views of the National Institutes of Health."

**ACS:** acute coronary syndrome; **AE:** adverse event; **CA:** continuous abstinence; **CIHR:** Canadian Institutes of Health Research; **C‐NRT:** combined nicotine replacement therapy; **CO:** carbon monoxide; **COPD:** chronic obstructive pulmonary disease; **CPD:** cigarettes per day; **e‐cigarette:** electronic cigarette; **MDD:** major depressive disorder; **NRT:** nicotine replacement therapy; **PPA:** point‐prevalence abstinence; **ppm:** parts per million; **QoL:** quality of life; **RCT:** randomised controlled trial; **SAE:** serious adverse event; **TQD:** target quit date

## Differences between protocol and review

For this update we did some restructuring of the meta‐analyses and summary of findings tables to better present the evidence available, including adding analyses of adverse events for comparisons of varenicline with other pharmacotherapies. We excluded previously included studies as these studies recruited already‐abstinent participants (Evins 2014; Tonstad 2006; Tønnesen 2013; NCT00828113), and because follow‐up was under six months (Hajek 2015). We also excluded Brandon 2011; Ebbert 2011b; Faessel 2009; Fagerström 2010; Garza 2011; Hughes 2011; McClure 2013; Meszaros 2013; Mitchell 2012, which had previously been included for data on harms only but do not meet all inclusion criteria.

## References

[CD006103-bib-0001] Anthenelli RM, Morris C, Ramey TS, Dubrava SJ, Tsilkos K, Russ C, et al. Effects of varenicline on smoking cessation in adults with stably treated current or past major depression: a randomized trial. Annals of Internal Medicine 2013;159(6):390-400. [CENTRAL: 921009] [EMBASE: 2013581859] [PMID: 24042367] 24042367 10.7326/0003-4819-159-6-201309170-00005

[CD006103-bib-0002] Doran N, Dubrava S, Anthenelli RM. Effects of varenicline, depressive symptoms, and region of enrollment on smoking cessation in depressed smokers. Nicotine & Tobacco Research 2018;21(2):156-62. 10.1093/ntr/nty033 PMC6329403 29471329

[CD006103-bib-0003] NCT01078298. Safety and efficacy of 12 weeks of varenicline for smoking cessation in smokers with depression. clinicaltrials.gov/ct2/show/NCT01078298 (first received 2 March 2010). [ClinicalTrials.gov ID NCT01078298]

[CD006103-bib-0004] Ashare RL, Thompson M, Serrano K, Leone F, Metzger D, Frank I, et al. Placebo-controlled randomized clinical trial testing the efficacy and safety of varenicline for smokers with HIV. Drug and Alcohol Dependence 2019;200:26-33. 10.1016/j.drugalcdep.2019.03.011 PMC6588414 31082665

[CD006103-bib-0005] Ashare RL, Wileyto EP, Logue-Chamberlain E, Gross R, Tyndale RF, Lerman C, et al. Patterns of lapses and recoveries during a quit attempt using varenicline and behavioral counseling among smokers with and without HIV. Psychology of Addictive Behaviors 2021;35(7):788-96. 10.1037/adb0000619 PMC7855008 32686950

[CD006103-bib-0006] Bauer AM, Hosie Quinn M, Lubitz SF, Flitter A, Ashare RL, Leone FT, et al. Medication adherence and rate of nicotine metabolism are associated with response to treatment with varenicline among smokers with HIV. Addictive Behaviors 2021;112:106638. 10.1016/j.addbeh.2020.106638 PMC7572610 32911350

[CD006103-bib-0007] Lubitz SF, Flitter A, Ashare RL, Thompson M, Leone F, Gross R, et al. Improved clinical outcomes among persons with HIV who quit smoking. AIDS Care 2020;32(10):1217-23. 10.1080/09540121.2019.1703891 PMC7297654 31847536

[CD006103-bib-0008] NCT01710137. A placebo controlled trial of varenicline for smoking among those with HIV/AIDS. ClinicalTrials.gov/show/NCT01710137 (first received 6 October 2019).

[CD006103-bib-0009] Quinn MH, Bauer AM, Flitter A, Lubitz SF, Ashare RL, Thompson M, et al. Correlates of varenicline adherence among smokers with HIV and its association with smoking cessation. Addictive Behaviors 2020;102:106151. 10.1016/j.addbeh.2019.106151 PMC6934914 31783245

[CD006103-bib-0010] Thompson M, Schnoll R, Serrano K, Leone F, Gross R, Collman R G, et al. The effect of varenicline on mood and cognition in smokers with HIV. Psychopharmacology 2020;237(4):1223-31. 10.1007/s00213-020-05451-w PMC7125016 31938877

[CD006103-bib-0011] Aubin H-J, Bobak A, Britton JR, Oncken C, Billing CB, Gong J, et al. Authors' reply [to JE Rose]. Thorax 2008;63(8):752. 10.1136/thx.2007.090647 PMC2569194 18263663

[CD006103-bib-0012] Aubin H-J, Bobak A, Britton JR, Oncken C, Billing CB, Gong J, et al. Authors' reply [to T Hillman]. Thorax 2008;63(8):752-3. 10.1136/thx.2007.090647 PMC2569194 18263663

[CD006103-bib-0013] Aubin H-J, Bobak A, Britton JR, Oncken C, Billing CB, Gong J, et al. Varenicline versus transdermal nicotine patch for smoking cessation: results from a randomised, open-label trial. Thorax 2008;1:1-8. 10.1136/thx.2007.090647 PMC2569194 18263663

[CD006103-bib-0014] Aveyard P. The place of varenicline in smoking cessation treatment. Thorax 2008;63(8):666-8. 10.1136/thx.2008.096081 18663065

[CD006103-bib-0015] Hillman T, Rajakulasingam K, Bhowmik A. Clinically significant outcomes in smoking cessation. Thorax 2008;63(8):752. 18663078

[CD006103-bib-0016] Rose JE. Pre-cessation varenicline treatment vs post-cessation NRT: an uneven playing field. Thorax 2008;63(8):751. 18663077

[CD006103-bib-0017] Baker TB, Piper ME, Stein JH, Smith SS, Bolt DM, Fraser DL, et al. Effects of nicotine patch vs varenicline vs combination nicotine replacement therapy on smoking cessation at 26 weeks. JAMA 2016;315(4):371-9. 10.1001/jama.2015.19284 PMC4824537 26813210

[CD006103-bib-0018] Chakraborti Y, Coffman DL, Piper ME. Time-varying mediation of pharmacological smoking cessation treatments on smoking lapse via craving, cessation fatigue, and negative mood. Nicotine & Tobacco Research 2022;24(10):1548-55. 10.1093/ntr/ntac068 PMC9575970 35287166

[CD006103-bib-0019] Kaye JT, Johnson AL, Baker TB, Piper ME, Cook JW. Searching for personalized medicine for binge drinking smokers: smoking cessation using varenicline, nicotine patch, or combination nicotine replacement therapy. Journal of Studies on Alcohol and Drugs 2020;81(4):426-35. 10.15288/jsad.2020.81.426 PMC7437557 32800078

[CD006103-bib-0020] Kaye JT, Piper ME, Baker TB, Cook JW. Searching for personalized medicine for heavy drinking smokers: smoking cessation using varenicline, nicotine patch, or combination nicotine replacement therapy. Alcoholism: Clinical and Experimental Research Conference: 42nd Annual Scientific Meeting of the Research Society on Alcoholism 2019;43 Suppl 1:247A.

[CD006103-bib-0021] Kim N, McCarthy DE, Piper ME, Baker TB. Comparative effects of varenicline or combination nicotine replacement therapy versus patch monotherapy on candidate mediators of early abstinence in a smoking cessation attempt. Addiction 2020;116(4):926-35. 10.1111/add.15248 PMC7930141 32888230

[CD006103-bib-0022] McCarthy DE, Versella MV. Quitting failure and success with and without using medication: latent classes of abstinence and adherence to nicotine monotherapy, combination therapy, and varenicline. Nicotine & Tobacco Research 2019;21(11):1488-95. 10.1093/ntr/nty157 PMC6821172 30107419

[CD006103-bib-0023] Schlam TR, Baker TB, Smith SS, Cook JW, Piper ME. Anxiety sensitivity and distress tolerance in smokers: relations with tobacco dependence, withdrawal, and quitting success. Nicotine & Tobacco Research 2020;22(1):58-65. 10.1093/ntr/ntz070 PMC7297013 31056710

[CD006103-bib-0024] Baker TB, Piper ME, Smith SS, Bolt DM, Stein JH, Fiore MC. Effects of combined varenicline with nicotine patch and of extended treatment duration on smoking cessation: a randomized clinical trial. JAMA 2021;326(15):1485-93. 10.1001/jama.2021.15333 PMC8527361 34665204

[CD006103-bib-0025] NCT03176784. UW Quitting Using Intensive Treatment Study (QUITS). clinicaltrials.gov/show/NCT03176784 (first received 6 June 2017).

[CD006103-bib-0026] Benli AR, Erturhan S, Oruc MA, Kalpakci P, Sunay D, Demirel Y. A comparison of the efficacy of varenicline and bupropion and an evaluation of the effect of the medications in the context of the smoking cessation programme. Tobacco Induced Diseases 2017;15(10):1-8. [DOI: 10.1186/s12971-017-0116-0] PMC5286845 28167895

[CD006103-bib-0027] Bohadana A, Freier-Dror Y, Peles V, Babai P, Izbicki G. Extending varenicline preloading to 6 weeks facilitates smoking cessation: a single-site, randomised controlled trial: six-week varenicline preloading for smoking cessation. eClinicalMedicine 2020;19(100228):1-10. 10.1016/j.eclinm.2019.11.021 PMC7005428 32055787

[CD006103-bib-0028] Izbicki G, Freier Dror Y, Bohadana A. Extended, 6 weeks, varenicline preloading: does it facilitate smoking reduction and cessation? a randomized double-blind, placebo-controlled study. American Journal of Respiratory and Critical Care Medicine Conference: 2019 International Conference of the American Thoracic Society 2019;199(9):A2378.

[CD006103-bib-0029] NCT02634281. Extended (6-Week) varenicline preloading: does it facilitate smoking reduction and cessation? https://clinicaltrials.gov/show/NCT02634281 (first received 18 December 2015).

[CD006103-bib-0030] Bolliger CT, Issa JS, Posadas-Valay R, Safwat T, Abreu P, Correia EA, et al. Effects of varenicline in adult smokers: a multinational, 24-week, randomized, double-blind, placebo-controlled study. Clinical Therapeutics 2011;33(4):465-77. [CENTRAL: 799588] [PMID: 21635992] 21635992 10.1016/j.clinthera.2011.04.013

[CD006103-bib-0031] Brinn M, Daziel K, Carson K, Labiszewski N, Esterman A, Smith B. Cost effectiveness of an inpatient smoking cessation intervention for patients with tobacco related illnesses (Stop Trial): a multi-centre RCT [Abstract]. Respirology 2013;18 Suppl 2:16 [O027]. [CENTRAL: 849080] [EMBASE: 71010964]

[CD006103-bib-0032] Carson K, Brinn M, Peters M, Fitridge R, Koblar S, Jannes J, et al. Superiority of a course of varenicline tartrate plus counselling over counselling alone for smoking cessation: a 24-month randomised controlled trial for inpatients. Respirology 2016;21 Suppl 2:45. 10.1136/thoraxjnl-2012-202484 22993168

[CD006103-bib-0033] Carson K, Smith BJ, Peters MJ, Veale AJ, Esterman AJ. Superiority of a course of varenicline tartrate plus counselling over counselling alone for smoking cessation: A 24-month randomized controlled trial for inpatients. Respirology 2015;20 Suppl 3:115.

[CD006103-bib-0034] Carson K, Smith BJ, Peters MJ, Veale AJ, Esterman AJ. Superiority of a course of varenicline tartrate plus counselling over counselling alone for smoking cessation: a 24-month randomized controlled trial for inpatients. Respirology 2015;20:115. 10.1136/thoraxjnl-2012-202484 22993168

[CD006103-bib-0035] Carson KV, Brinn MP, Smith BJ, Garside CG, Goldsworthy SJ, Fitridge RA. Varenicline tartrate and counselling versus counselling alone in a randomised controlled trial for inpatient smoking cessation: 3 month interim results [Abstract]. Respirology 2010;15 Suppl 1:A30.

[CD006103-bib-0036] Carson KV, Smith BJ, Brinn MP, Peters MJ, Fitridge R, Koblar SA, et al. Safety of varenicline tartrate and counseling versus counseling alone for smoking cessation: a randomized controlled trial for inpatients (STOP study). Nicotine & Tobacco Research 2014;16(11):1495-502. [CENTRAL: 1036725] [EMBASE: 2014920189] [PMID: 25031315] 25031315 10.1093/ntr/ntu112

[CD006103-bib-0037] Carson-Chahhoud KV, Smith BJ, Peters MJ, Brinn MP, Ameer F, Singh K, et al. Two-year efficacy of varenicline tartrate and counselling for inpatient smoking cessation (STOP study): a randomized controlled clinical trial. PloS One 2020;15(4):e0231095. 10.1371/journal.pone.0231095 PMC7190140 32348306

[CD006103-bib-0038] Hnin K, Carson K, Brinn M, Jannes J, Ameer F, Esterman A, et al. Triggers resulting in relapse: cohort analysis from the smoking termination opportunity for inpatients (STOP) trial [Abstract]. Respirology 2014;19 Suppl 2:40 [TO 076]. [CENTRAL: 996495] [EMBASE: 71605471]

[CD006103-bib-0039] Smith B, Carson K, Dalziel K, Brinn M, Moayeri F, Clarke P, et al. Cost effectiveness of inpatient initiated varenicline tartrate (VT) plus counselling compared to counselling alone: 2 year follow-up of the Smoking Termination Opportunity for inPatients (STOP) study. European respiratory journal. Conference: European Respiratory Society Annual Congress 2016. United Kingdom 2016;48:PA4600.

[CD006103-bib-0040] Smith BJ, Carson KV, Brinn MP, Labiszewski NA, Peters MJ, Fitridge R, et al. Smoking termination opportunity for in patients (STOP): superiority of a course of varenicline tartrate plus counselling over counselling alone for smoking cessation: a 12-month randomised controlled trial for inpatients. Thorax 2013;68(5):485-6. [CENTRAL: 849081] [EMBASE: 2013242202] [NCT01141855/ClinicalTrials.gov] [PMID: 22993168] 22993168 10.1136/thoraxjnl-2012-202484

[CD006103-bib-0041] Smith BJ, Peters MJ, Fitridge RA, Esterman AJ, Litt JC, Horowitz JD, et al. Varenicline tartrate and counselling versus counselling alone in a randomised controlled trial for inpatient smoking cessation: 6 month interim results. Conference: American Thoracic Society 2011 International Conference, May 13-18 2011.

[CD006103-bib-0042] Chen LS, Baker TB, Miller JP, Bray M, Smock N, Chen J, et al. Genetic variant in CHRNA5 and response to varenicline and combination nicotine replacement in a randomized placebo-controlled trial. Clinical Pharmacology and Therapeutics 2020;108(6):1315-25. 10.1002/cpt.1971 PMC7993015 32602170

[CD006103-bib-0043] NCT02351167. Genetically informed smoking cessation trial. ClinicalTrials.gov/show/NCT02351167 (first received 30 January 2015).

[CD006103-bib-0044] Chengappa KN, Perkins KA, Brar JS, Schlicht PJ, Turkin SR, Hetrick ML, et al. Varenicline for smoking cessation in bipolar disorder: a randomized, double-blind, placebo-controlled study. Journal of Clinical Psychiatry 2014;75(7):765-72. [CENTRAL: 1014641] 10.4088/JCP.13m08756 25006684

[CD006103-bib-0045] Chengappa KN. A randomized, double-blind, placebo-controlled clinical trial of varenicline in persons with bipolar disorder motivated to quit smoking. Bipolar Disorders 2015;17:38.

[CD006103-bib-0046] Forrest PE, Brinson AJ, Gannon JM, George TP, Perkins KA, Chengappa KN. An association between the use of hypnotics and quit status in the treatment of nicotine dependence with varenicline in bipolar disorder. Journal of Clinical Psychopharmacology 2015;35(2):199-200. 10.1097/JCP.0000000000000272 PMC4344405 25627075

[CD006103-bib-0047] NCT01010204. Varenicline treatment for smoking cessation in patients with bipolar disorder (BEST). clinicaltrials.gov/ct2/show/NCT01010204 (first received 9 November 2009). [ClinicalTrials.gov ID NCT01010204]

[CD006103-bib-0048] Cinciripini PM, Green CE, Robinson JD, Karam-Hage M, Engelmann JM, Minnix JA, et al. Benefits of varenicline vs. bupropion for smoking cessation: a Bayesian analysis of the interaction of reward sensitivity and treatment. Psychopharmacology 2017;234(11):1769-79. 10.1007/s00213-017-4580-2 PMC5901731 28275830

[CD006103-bib-0049] Cinciripini PM, Karam-Hage M. Randomised controlled trial: study suggests varenicline safe and effective among adults with stable depression. Evidence-Based Medicine 2014;19(3):92. [EMBASE: 2014382469] [PMID: 24482150] 24482150 10.1136/eb-2013-101619 PMC9387515

[CD006103-bib-0050] Cinciripini PM, Robinson JD, Karam-Hage M, Minnix JA, Lam C, Versace F, et al. Effects of varenicline and bupropion sustained-release use plus intensive smoking cessation counseling on prolonged abstinence from smoking and on depression, negative affect, and other symptoms of nicotine withdrawal. JAMA Psychiatry 2013;70(5):522-33. [CENTRAL: 863881] [EMBASE: 2013278203] [PMID: 23536105] 23536105 10.1001/jamapsychiatry.2013.678 PMC4128024

[CD006103-bib-0051] Cui Y, Engelmann J M, Xian J, Minnix J A, Lam C Y, Karam-Hage M, et al. Pharmacological intervention and abstinence in smokers undergoing cessation treatment: a psychophysiological study. International Journal of Psychophysiology 2018;123:25-34. 10.1016/j.ijpsycho.2017.12.001 PMC5759327 29223599

[CD006103-bib-0052] Hays JT. Varenicline may reduce negative effect while aiding smoking cessation. Evidence-Based Medicine 2014;19(1):23. [PMID: 23990527] 23990527 10.1136/eb-2013-101435

[CD006103-bib-0053] NCT00507728. Pharmacogenetics, emotional reactivity and smoking. ClinicalTrials.gov/ct2/NCT00507728 (first received 26 July 207). [ClinicalTrials.gov ID NCT00507728]

[CD006103-bib-0054] Cinciripini PM, Minnix JA, Green CE, Robinson JD, Engelmann JM, Versace F, et al. An RCT with the combination of varenicline and bupropion for smoking cessation: clinical implications for front line use. Addiction 2018;113(9):1673-82. 10.1111/add.14250 PMC6196126 29679432

[CD006103-bib-0055] NCT00943618. Combining varenicline and bupropion for smoking cessation. ClinicalTrials.gov/ct2/NCT00943618 (first received 22 July 2009).

[CD006103-bib-0056] ACTRN12616001654448. A non-inferiority trial of cytisine versus varenicline for smoking cessation. http://www.who.int/trialsearch/Trial2.aspx?TrialID=ACTRN12616001654448 (first received 30 November 2016).

[CD006103-bib-0057] Courtney RJ, McRobbie H, Tutka P, Weaver NA, Petrie D, Mendelsohn CP, et al. Effect of cytisine vs varenicline on smoking cessation: a randomized clinical trial. JAMA 2021;326(1):56-64. 10.1001/jama.2021.7621 PMC8261608 34228066

[CD006103-bib-0058] Thomas D, Farrell M, McRobbie H, Tutka P, Petrie D, West R, et al. The effectiveness, safety and cost-effectiveness of cytisine versus varenicline for smoking cessation in an Australian population: a study protocol for a randomized controlled non-inferiority trial. Addiction 2019;114(5):923-33. 10.1111/add.14541 30589984

[CD006103-bib-0059] Cox LS, Nollen NL, Mayo MS, Faseru B, Greiner A, Ellerbeck EF, et al. Effect of varenicline added to counseling on smoking cessation among African American daily smokers: the Kick It at Swope IV randomized clinical trial. JAMA 2022;327(22):2201-9. [DOI: 10.1001/jama.2022.8274.] PMC9198729 35699705

[CD006103-bib-0060] Liebmann EP. Dissertation Abstracts International: Section B: The Sciences and Engineering. Vol. 82. 2021.

[CD006103-bib-0061] NCT02360631. Advancing tobacco use treatment for African American smokers. ClinicalTrials.gov/show/NCT02360631 (first received 10 February 2015).

[CD006103-bib-0062] De Dios MA, Anderson BJ, Stanton C, Audet DA, Stein M. Project Impact: a pharmacotherapy pilot trial investigating the abstinence and treatment adherence of Latino light smokers. Journal of Substance Abuse Treatment 2012;43(3):322-30. [CENTRAL: 836798] [EMBASE: 2012562711] [PMID: 22377389] 22377389 10.1016/j.jsat.2012.01.004 PMC3762477

[CD006103-bib-0063] Boeckmann M, Nohavova I, Dogar O, Kralikova E, Pankova A, Zvolska K, et al. Protocol for the mixed-methods process and context evaluation of the TB & Tobacco randomised controlled trial in Bangladesh and Pakistan: a hybrid effectiveness-implementation study. BMJ Open 2018;8(3):e019878. 10.1136/bmjopen-2017-019878 PMC5887198 29602847

[CD006103-bib-0064] Dogar O, Barua D, Boeckmann M, Elsey H, Fatima R, Gabe R, et al. The safety, effectiveness and cost-effectiveness of cytisine in achieving six-month continuous smoking abstinence in tuberculosis patients—protocol for a double-blind, placebo-controlled randomized trial. Addiction 2018;113(9):1716-27. 10.1111/add.14242 PMC6099220 29676824

[CD006103-bib-0065] Dogar O, Keding A, Gabe R, Marshall AM, Huque R, Barua D, et al. Cytisine for smoking cessation in patients with tuberculosis: a multicentre, randomised, double-blind, placebo-controlled phase 3 trial. Lancet Global Health 2020;8(11):e1408-17. 10.1016/S2214-109X(20)30312-0 33069301

[CD006103-bib-0066] ISRCTN43811467. Tuberculosis (TB) and tobacco. www.isrctn.com/ISRCTN43811467 (first received 23 March 2016).

[CD006103-bib-0067] Siddiqi K, Keding A, Marshall AM, Dogar O, Li J, Huque R, et al. Effect of quitting smoking on health outcomes during treatment for tuberculosis: secondary analysis of the TB & Tobacco Trial. Thorax 2021;7:74-8. 10.1136/thoraxjnl-2020-215926 34272336

[CD006103-bib-0068] Anthenelli R, Benowitz N, West R, St Aubin L, McRae T, Lawrence D, et al. Reports of suicidal ideation and behavior in the EAGLES trial. Proceedings of the Society for Research on Nicotine and Tobacco, USA 3rd - 5th March 2016;SYM5D:8.

[CD006103-bib-0069] Anthenelli RM, Benowitz NL, West R, St Aubin L, McRae T, Lawrence D, et al. Neuropsychiatric safety and efficacy of varenicline, bupropion, and nicotine patch in smokers with and without psychiatric disorders (EAGLES): a double-blind, randomised, placebo-controlled clinical trial. Lancet 2016;387(10037):2507-20. [DOI: dx.doi.org/10.1016/S0140-6736(16)30272-0] 10.1016/S0140-6736(16)30272-0 27116918

[CD006103-bib-0070] Anthenelli RM, Gaffney M, Benowitz NL, West R, McRae T, Russ C, et al. Predictors of neuropsychiatric adverse events with smoking cessation medications in the randomized controlled EAGLES trial. Journal of General Internal Medicine 2019;34(6):862-70. 10.1007/s11606-019-04858-2 PMC6544691 30847828

[CD006103-bib-0071] Ayers CR, Heffner JL, Russ C, Lawrence D, McRae T, Evins AE, et al. Efficacy and safety of pharmacotherapies for smoking cessation in anxiety disorders: subgroup analysis of the randomized, active- and placebo-controlled EAGLES trial. Depression and Anxiety 2020;37(3):247-60. 10.1002/da.22982 PMC7064930 31850603

[CD006103-bib-0072] Baker CL, Pietri G. A cost-effectiveness analysis of varenicline for smoking cessation using data from the EAGLES trial. ClinicoEconomics and outcomes research 2018;10:67-74. 10.2147/CEOR.S153897 PMC5783141 29403297

[CD006103-bib-0073] Beard E, Jackson SE, Anthenelli RM, Benowitz NL, Aubin LS, McRae T, et al. Estimation of risk of neuropsychiatric adverse events from varenicline, bupropion and nicotine patch versus placebo: secondary analysis of results from the EAGLES trial using Bayes factors. Addiction (Abingdon, England) 2021;116(10):2816-24. 10.1111/add.15440 PMC8612131 33885203

[CD006103-bib-0074] Benowitz N, Evins AE, West R, St Aubin L, McRae T, Lawrence D, et al. EAGLES trial: study design and neuropsychiatric safety results. In: Society for Research on Nicotine and Tobacco, USA 3rd - 5th March. Vol. SYM5B. 2016:7.

[CD006103-bib-0075] Benowitz NL, Pipe A, West R, Hays JT, Tonstad S, McRae T, et al. Cardiovascular safety of varenicline, bupropion, and nicotine patch in smokers: a randomized clinical trial. JAMA Internal Medicine 2018;178(5):622-31. 10.1001/jamainternmed.2018.0397 PMC6145797 29630702

[CD006103-bib-0076] Correa JB, Lawrence D, McKenna BS, Gaznick N, Saccone PA, Dubrava S, et al. Psychiatric co-morbidity and multi-morbidity in the EAGLES trial: descriptive correlates and associations with neuropsychiatric adverse events, treatment adherence, and smoking cessation. Nicotine & Tobacco Research 2021;23(10):1636-55. 10.1093/ntr/ntab056 PMC8521682 33788933

[CD006103-bib-0077] Ebbert J, Jimenez-Ruiz C, Dutro M P, Fisher M, Li J, Hays J T. Frequently reported adverse events with smoking cessation medications: post hoc analysis of a randomized trial. Mayo Clinic Proceedings 2021;96(7):1801-11. 10.1016/j.mayocp.2020.10.046 34112520

[CD006103-bib-0078] Evins AE, Benowitz NL, West R, Russ C, McRae T, Lawrence D, et al. Neuropsychiatric safety and efficacy of varenicline, bupropion, and nicotine patch in smokers with psychotic, anxiety, and mood disorders in the EAGLES trial. Journal of Clinical Psychopharmacology 2019;39(2):108-16. 10.1097/JCP.0000000000001015 PMC6488024 30811371

[CD006103-bib-0079] Evins AE, West R, Benowitz NL, Russ C, Lawrence D, McRae T, et al. Efficacy and safety of pharmacotherapeutic smoking cessation aids in schizophrenia spectrum disorders: subgroup analysis of EAGLES. Psychiatric Services (Washington, D.C.) 2021;72(1):7-15. 10.1176/appi.ps.202000032 PMC10228639 33138708

[CD006103-bib-0080] Heffner JL, Evins AE, Russ C, Lawrence D, Ayers CR, McRae T, et al. Safety and efficacy of first-line smoking cessation pharmacotherapies in bipolar disorders: subgroup analysis of a randomized clinical trial. Journal of Affective Disorders 2019;256:267-77. 10.1016/j.jad.2019.06.008 PMC8936081 31195244

[CD006103-bib-0081] NCT01456936. A phase 4, randomized, double blind, active and placebo controlled multicenter study evaluating the neuropsychiatric safety and efficacy of 12 weeks varenicline tartrate 1mg BID and bupropion hydrochloride 150 mg BID for smoking cessation in subjects with and without a history of psychiatric disorders [EAGLES]. clinicaltrials.gov/ct2/show/NCT01456936 (first received 21 October 2011).

[CD006103-bib-0082] Nollen NL, Ahluwalia JS, Sanderson Cox L, Okuyemi K, Lawrence D, Samuels L, et al. Assessment of racial differences in pharmacotherapy efficacy for smoking cessation: secondary analysis of the EAGLES randomized clinical trial. JAMA Network Open 2021;4(1):e2032053. 10.1001/jamanetworkopen.2020.32053 PMC7816102 33464316

[CD006103-bib-0083] Prochaska J, Benowitz N, West R, Anthenelli R. Evaluating adverse events in a global smoking cessation study (EAGLES): a randomized controlled trial comparing the safety and efficacy of the first-line smoking cessation aids in smokers with and without psychiatric disorders. Proceedings of the Society for Research on Nicotine and Tobacco, USA 3rd - 5th March 2016;SYM5:7.

[CD006103-bib-0084] Prochaska J. Neuropsychiatric risk concerns in the context of smoking, quitting, and cessation pharmacotherapy use. Proceedings of the Society for Research on Nicotine and Tobacco, USA 3rd - 5th March 2016;SYM5A:7.

[CD006103-bib-0085] West R, Benowitz N, Evins AE, St Aubin L, McRae T, Lawrence D, et al. Relative efficacy of varenicline, bupropion SR, and nicotine transdermal patch in aiding smoking cessation in the EAGLES trial. Proceedings of the Society for Research on Nicotine and Tobacco, USA 3rd - 5th March 2016;SYM5C:8.

[CD006103-bib-0086] West R, Evins AE, Benowitz NL, Russ C, McRae T, Lawrence D, et al. Factors associated with the efficacy of smoking cessation treatments and predictors of smoking abstinence in EAGLES. Addiction 2018;113(8):1507-16. 10.1111/add.14208 PMC6055735 29508470

[CD006103-bib-0087] Wilcox C, Oskooilar N, Guevarra K, Linh Tong M, Grosz D, Morrissey J, et al. A double-blind, active-and placebo-controlled evaluation of the neuropsychiatric safety and efficacy of varenicline and bupropion for smoking cessation in subjects with (pre-existing) psychiatric disorders: an objective blinded analysis. Neuropsychopharmacology 2015;40:S260.

[CD006103-bib-0088] Bastian H. Comment on Ebbert 2015 'Reduce to Quit' trial. www.ncbi.nlm.nih.gov/pubmed/?term=25688780 Feb 18 2015 (accessed 11 June 2015).

[CD006103-bib-0089] Ebbert JO, Hughes JR, West RJ, Rennard SI, Russ C, McRae TD, et al. Effect of varenicline on smoking cessation through smoking reduction: a randomized clinical trial. JAMA 2015;313(7):687-94. [CENTRAL: 1050651] [PMID: 25688780] 25688780 10.1001/jama.2015.280 PMC4883651

[CD006103-bib-0090] Ebbert JO, Hughes JR, West RJ. Effect of varenicline on smoking cessation through smoking reduction: a randomized clinical trial. Journal of Vascular Surgery 2015;313(7):687-94. 10.1001/jama.2015.280 PMC4883651 25688780

[CD006103-bib-0091] NCT01370356. A study to evaluate the efficacy and safety of varenicline compared to placebo for smoking cessation through reduction. clinicaltrials.gov/ct2/show/NCT01370356 (first received 9 June 2011). [ClinicalTrials.gov ID NCT01370356]

[CD006103-bib-0092] Nakamura M, Abe M, Ohkura M, Treadow J, Yu CR, Park PW. Efficacy of varenicline for cigarette reduction before quitting in Japanese smokers: a subpopulation analysis of the reduce to quit trial. Clinical Therapeutics 2017;39(4):863-72. 10.1016/j.clinthera.2017.03.007 28365035

[CD006103-bib-0093] Wilcox C, Grosz D, Tong M L, Morrissey J, De Francisco D, Guevarra K, et al. Smoking cessation through reduction: does it enhance or diminish successful quitting? Neuropsychopharmacology 2014;39:S343-4.

[CD006103-bib-0094] Ebbert JO, Croghan IT, Hurt RT, Schroeder DR, Hays JT. Varenicline for smoking cessation in light smokers. Nicotine & Tobacco Research 2016;18(10):2031-5. 10.1093/ntr/ntw123 PMC6280997 27117285

[CD006103-bib-0095] Fouz-Roson N, Montemayor-Rubio T, Almadana-Pacheco V, Montserrat-Garcia S, Gomez-Bastero AP, Romero-Munoz C, et al. Effect of 0.5 mg versus 1 mg varenicline for smoking cessation: a randomized controlled trial. Addiction 2017;112(9):1610-9. 10.1111/add.13855 28449281

[CD006103-bib-0096] Roson NF, Panadero-Paz C, Almadana-Pacheco V, Benito-Bernaldez C, Rodriguez-Martin PJ, Montemayor-Rubio T. Influence of psychiatric disorders in patients treated with varenicline. European Respiratory Journal 2017;50:PA4478.

[CD006103-bib-0097] Roson NF, Panadero-Paz C, Benito-Bernaldez C, Rodriguez-Martin PJ, Almadana-Pacheco V, Montemayor-Rubio T. Influence of respiratory and cardiovascular diseases on smoking cessation. European Respiratory Journal 2017;50 Suppl 61:PA2987.

[CD006103-bib-0098] Roson NF, Panadero-Paz C, Benito-Bernaldez C, Rodriguez-Martin PJ, Romero-Munoz C, Rubio TM. Short-term low-dose vs standard-dose varenicline therapy for smoking cessation: a randomized controlled trial. European Respiratory Journal 2017;50:PA4480.

[CD006103-bib-0099] Chen LS, Baker TB, Jorenby D, Piper M, Saccone N, Johnson E, et al. Genetic variation (CHRNA5), medication (combination nicotine replacement therapy vs. varenicline), and smoking cessation. Drug and Alcohol Dependence 2015;154:278-82. 10.1016/j.drugalcdep.2015.06.022 PMC4537319 26142345

[CD006103-bib-0100] Gonzales D, Jorenby DE, Brandon T, Arteaga C, Lee TC. Delayed quitting, lapse recovery and long-term outcomes for quitters taking varenicline, bupropion and placebo. Society for Research on Nicotine and Tobacco Annual Meeting, Portland OR, Feb 27-March 1st 2008.

[CD006103-bib-0101] Gonzales D, Jorenby DE, Brandon TH, Arteaga C, Lee TC. Emergent adverse psychiatric symptoms by therapy during 12 weeks of treatment with varenicline, bupropion SR, or placebo for smoking cessation. Society for Research on Nicotine and Tobacco Europe: Rome, September 23-26 2008.

[CD006103-bib-0102] Gonzales D, Jorenby DE, Brandon TH, Arteaga C, Lee TC. Immediate versus delayed quitting and rates of relapse among smokers treated successfully with varenicline, bupropion SR or placebo. Addiction 2010;105(11):2002-13. [DOI: 10.1111/j.1360-0443.2010.03058.x] PMC2991770 20819082

[CD006103-bib-0103] Gonzales D, Rennard SI, Nides M, Oncken C, Azoulay S, Billing CB et al. Varenicline, an α4ß2 nicotinic acetylcholine receptor partial agonist, vs sustained-release bupropion and placebo for smoking cessation. JAMA 2006;296(1):47-55. 10.1001/jama.296.1.47 16820546

[CD006103-bib-0104] Hays JT, Leischow SJ, Lawrence D, Lee TC. Adherence to treatment for tobacco dependence: association with smoking abstinence and predictors of adherence. Nicotine & Tobacco Research 2010;12(6):574-81. 10.1093/ntr/ntq047 20457644

[CD006103-bib-0105] Heffner JL, Lee TC, Arteaga C, Anthenelli RM. Predictors of post-treatment relapse to smoking in successful quitters: pooled data from two phase III varenicline trials. Drug and Alcohol Dependence 2010;109:120-5. 10.1016/j.drugalcdep.2009.12.022 PMC2875368 20071105

[CD006103-bib-0106] Jackson KC, Nahoopii R, Said Q, Dirani R, Brixner D. An employer-based cost-benefit analysis of a novel pharmacotherapy agent for smoking cessation. Journal of Occupational and Environmental Medicine 2007;49(4):453-60. 10.1097/JOM.0b013e3180459ff2 17426529

[CD006103-bib-0107] Nides M, Glover ED, Reus VI, Christen AG, Make BJ, Billing CB, et al. Varenicline versus bupropion SR or placebo for smoking cessation: a pooled analysis. American Journal of Health Behavior 2008;32(6):664-75. 10.5555/ajhb.2008.32.6.664 18442345

[CD006103-bib-0108] O'Brien CP. A new medication for the worst addiction. Current Psychiatry Reports 2007;9(5):347-8. 17915070

[CD006103-bib-0109] Ravva P, Gastonguay MR, French JL, Tensfeldt TG, Faessel HM. Quantitative assessment of exposure-response relationships for the efficacy and tolerability of varenicline for smoking cessation. Clinical Pharmacology and Therapeutics 2010;87(3):336-44. 10.1038/clpt.2009.282 20107434

[CD006103-bib-0110] Tonstad S. Practical implementation of varenicline as an aid to smoking cessation in clinical practice. Pneumologia 2009;58(3):167-74. 19817313

[CD006103-bib-0111] West R, Baker CL, Cappelleri JC, Bushmakin AG. Effect of varenicline and bupropion on craving, nicotine withdrawal symptoms, and rewarding effects of smoking during a quit attempt. UK National Smoking Cessation Conference Proceedings, London, June 2007 2007.

[CD006103-bib-0112] Xenakis JG, Kinter ET, Ishak KJ, Ward AJ, Marton JP, Willke RJ, et al. A discrete-event simulation of smoking-cessation strategies based on varenicline pivotal trial data. PharmacoEconomics 2011;29:497-510. 10.2165/11589230-000000000-00000 21452908

[CD006103-bib-0113] Gonzales D, Hajek P, Pliamm L, Nackaerts K, Tseng L-J, McRae TD, et al. Retreatment with varenicline for smoking cessation in smokers who have previously taken varenicline: a randomized, placebo-controlled trial. Clinical Pharmacology and Therapeutics 2014;96(3):390-6. [CENTRAL: 1014162] [EMBASE: 2014565570] [PMID: 24911368] 24911368 10.1038/clpt.2014.124 PMC4151018

[CD006103-bib-0114] NCT01244061. A multi-national study to assess how effective and safe the smoking cessation medicine varenicline is in smokers who have already tried varenicline in the past as a prescription medicine from their usual healthcare provider. clinicaltrials.gov/ct2/show/NCT01244061 (first received 19th November 2010). [ClinicalTrials.gov ID NCT01244061]

[CD006103-bib-0115] Gray KM, Baker NL, McClure EA, Tomko RL, Squeglia LM, Saladin ME, et al. Efficacy and safety of varenicline for adolescent smoking cessation: a randomized clinical trial. JAMA Pediatrics 2019;173(12):1146-53. 10.1001/jamapediatrics.2019.3553 PMC6802269 31609433

[CD006103-bib-0116] McClure EA, Baker NL, Hood CO, Tomko RL, Squeglia LM, Flanagan JC, et al. Cannabis and alcohol co-use in a smoking cessation pharmacotherapy trial for adolescents and emerging adults. Nicotine & Tobacco Research 2019;22(8):1374-82. 10.1093/ntr/ntz170 PMC7364825 31612956

[CD006103-bib-0117] NCT01509547. A randomized controlled trial of varenicline for adolescent smoking cessation. ClinicalTrials.gov/show/NCT01509547 (first received 13th January 2012).

[CD006103-bib-0118] Heydari G, Talischi F, Tafti SF, Masjedi MR. Quitting smoking with varenicline: parallel, randomised efficacy trial in Iran. International Journal of Tuberculosis and Lung Disease 2012;16(2):268-72. [CENTRAL: 814663] [EMBASE: 2012026893] [PMID: 22236931] 22236931 10.5588/ijtld.11.0183

[CD006103-bib-0119] IRCT138901111878N2. Evaluation of the effect of champ-ix in smoking cessation in smokers in Tehran. www.irct.ir/trial/1473 (first received 12 October 2010).

[CD006103-bib-0120] Hong H, Wang W, Chen L. Study on effect of 5A intervention method combined with varenicline for smoking cessation of COPD patients in stable stage. Chinese Nursing Research 2015;29(2C):667-70.

[CD006103-bib-0121] Hurt RT, Ebbert JO, Croghan IT, Schroeder DR, Hurt RD, Hays JT. Varenicline for tobacco-dependence treatment in alcohol-dependent smokers: a randomized controlled trial. Drug and Alcohol Dependence 2018;184:12-7. 10.1016/j.drugalcdep.2017.11.017 PMC5818285 29324248

[CD006103-bib-0122] NCT01347112. Varenicline treatment for active alcoholic smokers. clinicaltrials.gov/ct2/show/NCT01347112 (first received 4 May 2011).

[CD006103-bib-0123] Ikonomidis I, Kourea K, Vlastos D, Marinou M, Vlachos S, Varoudi M, et al. Varenicline vs. nicotine replacement therapy: a prospective study of changes in arterial stiffness, endothelial glycocalyx and oxidative stress in smokers during 1 year follow-up. European Heart Journal 2016;Suppl 1 European Society of Cardiology, ESC Congress 2016. Italy. 37:553-4.

[CD006103-bib-0124] Ikonomidis I, Marinou M, Vlastos D, Kourea K, Andreadou I, Liarakos N, et al. Effects of varenicline and nicotine replacement therapy on arterial elasticity, endothelial glycocalyx and oxidative stress during a 3-month smoking cessation program. Atherosclerosis 2017;262:123-30. 10.1016/j.atherosclerosis.2017.05.012 28549278

[CD006103-bib-0125] Ioakeimidis N, Vlachopoulos C, Georgakopoulos C, Abdelrasoul M, Skliros N, Katsi V, et al. Smoking cessation rates with varenicline and electronic cigarettes in relapsed smokers with a history of acute coronary syndrome. European Heart Journal 2018;Suppl 1: European Society of Cardiology Congress, ESC 2018. Germany. 39:242.

[CD006103-bib-0126] Johns D. Randomised controlled trial comparing varenicline plus counselling and brief counselling alone on smoking cessation in patients prone to lung cancer using carbon monoxide moniter. Supportive Care in Cancer 2017;2 Suppl 1: 2017 international MASCC/ISOO symposium: supportive care in cancer. USA:S52.

[CD006103-bib-0127] Johns DA. The efficacy of combination therapy with varenicline and bupropion for smoking cessation. Annals of Oncology 2017;Suppl 2: 7th European Lung Cancer Conference, ELCC 2017. Switzerland. 28:iii6-7.

[CD006103-bib-0128] Jorenby DE, Hays JT, Rigotti NA, Azoulay S, Watsky EJ, Williams KE, et al. Efficacy of varenicline, an α4ß2 nicotinic acetylcholine receptor partial agonist, vs placebo or sustained-release bupropion for smoking cessation. JAMA 2006;296(1):56-63. 10.1001/jama.296.1.56 16820547

[CD006103-bib-0129] O'Brien CP. A second varenicline trial. Current Psychiatry Reports 2007;9(5):348-8. 17915071

[CD006103-bib-0130] King A, Vena A, Wit H de, Grant JE, Cao D. Effect of combination treatment with varenicline and nicotine patch on smoking cessation among smokers who drink heavily: a randomized clinical trial. JAMA Network Open 2022;5(3):e220951. 10.1001/jamanetworkopen.2022.0951 PMC8897753 35244704

[CD006103-bib-0131] NCT02859142. Varenicline augmentation of patch outcomes in heavy drinkers' smoking cessation. clinicaltrials.gov/show/NCT02859142 (first received 8 August 2016).

[CD006103-bib-0132] Le Mao R, Tromeur C, Paleiron N, Sanchez O, Gagnadoux F, Jouneau S, et al. Effect of early initiation of varenicline on smoking cessation in COPD patients admitted for exacerbation: the save randomized clinical trial. COPD 2020;17(1):7-14. 10.1080/15412555.2019.1703928 31854207

[CD006103-bib-0133] NCT01694732. Efficacy of varenicline associated with intensive counselling versus placebo of varenicline associated with intensive counselling on smoking cessation at the acute phase of an exacerbation of chronic obstructive pulmonary disease (COPD). A multicenter randomized double-blind trial. ClinicalTrials.gov/show/NCT01694732 (first received 27 September 2012).

[CD006103-bib-0134] Tromeur C, Le Mao R, Couturand F. Effect of varenicline on smoking cessation in COPD patients recovering from exacerbation: a randomized trial. Fundamental & Clinical Pharmacology 2018;32 Suppl 1:32.

[CD006103-bib-0135] Ashare RL, Lerman C, Tyndale RF, Hawk LW, George TP, Cinciripini P, et al. Sleep disturbance during smoking cessation: withdrawal or side effect of treatment? Journal of Smoking Cessation 2017;12(2):63-70. 10.1017/jsc.2016.11 PMC5443250 28553407

[CD006103-bib-0136] Ashare RL, Wileyto EP, Logue-Chamberlain E, Gross R, Tyndale RF, Lerman C, et al. Patterns of lapses and recoveries during a quit attempt using varenicline and behavioral counseling among smokers with and without HIV. Psychology of Addictive Behaviors 2021;35(7):788-96. 10.1037/adb0000619 PMC7855008 32686950

[CD006103-bib-0137] Chenoweth MJ, Novalen M, Hawk LWJ, Schnoll RA, George TP, Cinciripini PM, et al. Known and novel sources of variability in the nicotine metabolite ratio in a large sample of treatment-seeking smokers. Cancer Epidemiology, Biomarkers & Prevention 2014;23(9):1773-82. 10.1158/1055-9965.EPI-14-0427 PMC4154993 25012994

[CD006103-bib-0138] Chenoweth MJ, Schnoll RA, Novalen M, Hawk LW Jr, George TP, Cinciripini PM, et al. The nicotine metabolite ratio is associated with early smoking abstinence even after controlling for factors that influence the nicotine metabolite ratio. Nicotine & Tobacco Research 2016;18(4):491-5. 10.1093/ntr/ntv125 PMC6220835 26069034

[CD006103-bib-0139] Lerman C, Schnoll RA, Hawk LW Jr, Cinciripini P, George TP, Wileyto EP, et al. Use of the nicotine metabolite ratio as a genetically informed biomarker of response to nicotine patch or varenicline for smoking cessation: a randomised, double-blind placebo-controlled trial. Lancet Respiratory Medicine 2015;3(2):131-8. 10.1016/S2213-2600(14)70294-2 PMC4480925 25588294

[CD006103-bib-0140] NCT01314001. Pharmacogenetics of nicotine addiction treatment. clinicaltrials.gov/ct2/show/NCT01314001 (first received 14 March 2011).

[CD006103-bib-0141] Peng AR, Schnoll R, Hawk LW Jr, Cinciripini P, George TP, Lerman C, et al. Predicting smoking abstinence with biological and self-report measures of adherence to varenicline: impact on pharmacogenetic trial outcomes. Drug and Alcohol Dependence 2018;190:72-81. 10.1016/j.drugalcdep.2018.04.035 PMC6085881 29986268

[CD006103-bib-0142] Peng AR, Swardfager W, Benowitz NL, Ahluwalia JS, Lerman C, Nollen NL, et al. Impact of early nausea on varenicline adherence and smoking cessation. Addiction 2020;115(1):134-44. 10.1111/add.14810 PMC6933078 31502736

[CD006103-bib-0143] Peng AR. Measures of adherence to varenicline: impact on smoking cessation outcomes. Dissertation Abstracts International 2021;82.

[CD006103-bib-0144] Robinson JD, Li L, Chen M, Lerman C, Tyndale RF, Schnoll RA, et al. Evaluating the temporal relationships between withdrawal symptoms and smoking relapse. Psychology of Addictive Behaviors 2019;33(2):105-16. 10.1037/adb0000434 PMC6405298 30614717

[CD006103-bib-0145] Tyndale RF, Zhu AZ, George TP, Paul C, Hawk L, Schnoll R, et al. Lack of associations of CHRNA5-A3-B4 genetic variants with smoking cessation treatment outcomes in Caucasian smokers despite associations with baseline smoking. Plos One 2015;10(5):e0128109. 10.1371/journal.pone.0128109 PMC4444267 26010901

[CD006103-bib-0146] Bidwell LC, Karoly HC, Hutchison KE, Bryan AD. ADHD symptoms impact smoking outcomes and withdrawal in response to Varenicline treatment for smoking cessation. Drug and Alcohol Dependence 2017;179:18-24. 10.1016/j.drugalcdep.2017.06.020 PMC5599352 28738266

[CD006103-bib-0147] Cinnamon Bidwell L, Karoly H, Bryan A, Hutchison K. ADHD symptoms moderate the efficacy of verenacline in a randomized controlled trial. Neuropsychopharmacology 2016;41:S278.

[CD006103-bib-0148] Littlewood RA, Claus ED, Wilcox CE, Mickey J, Arenella PB, Bryan AD, et al. Moderators of smoking cessation outcomes in a randomized-controlled trial of varenicline versus placebo. Psychopharmacology 2017;234(23-24):3417-29. 10.1007/s00213-017-4721-7 28889258

[CD006103-bib-0149] Mercie P, Arsandaux J, Katlama C, Ferret S, Beuscart A, Spadone C, et al. Efficacy and safety of varenicline for smoking cessation in people living with HIV in France (ANRS 144 Inter-ACTIV): a randomised controlled phase 3 clinical trial. Lancet HIV 2018;5(3):e126-35. 10.1016/S2352-3018(18)30002-X 29329763

[CD006103-bib-0150] Mercie P, Roussillon C, Katlama C, Beuscart A, Ferret S, Wirth N, et al. Varenicline vs placebo for smoking cessation: ANRS 144 Inter-ACTIV randomized trial. Topics in Antiviral Medicine 2015;23:56-7.

[CD006103-bib-0151] NCT00918307. Efficacy and safety of varenicline amongst HIV-infected patients (Inter-ACTIV). ClinicalTrials.gov/show/NCT00918307 (first received 11 June 2009).

[CD006103-bib-0152] Griffin JL, Segal KS, Nahvi S. Barriers to telephone quitline use among methadone-maintained smokers. Nicotine & Tobacco Research 2015;17(8):931-6. 10.1093/ntr/ntu267 PMC4542843 26180217

[CD006103-bib-0153] NCT01027754. Smoking cessation treatment for methadone maintenance patients. clinicaltrials.gov/ct2/show/NCT01027754 (first received 9 December 2009). [ClinicalTrials.gov ID NCT01027754]

[CD006103-bib-0154] Nahvi S, Ning Y, Segal KS, Richter KP, Arnsten JH. Varenicline efficacy and safety among methadone maintained smokers: a randomized placebo-controlled trial. Addiction 2014;109(9):1554-63. [CENTRAL: 997902] [PMID: 24862167] 24862167 10.1111/add.12631 PMC4300953

[CD006103-bib-0155] Fagerström K, Nakamura M, Cho H, Tsa S, Wang C, Davies S, et al. Varenicline treatment for smoking cessation in Asian populations: a pooled analysis of placebo-controlled trials conducted in six Asian countries. Current Medical Research & Opinion 2010;26(9):2165-73. 10.1185/03007995.2010.505130 20666691

[CD006103-bib-0156] Igarashi A, Takuma H, Fukada T, Tsutani K. Cost-utility analysis of varenicline, an oral smoking-cessation drug, in Japan. Pharmacoeconomics 2009;27(3):247-61. 10.2165/00019053-200927030-00007 19354344

[CD006103-bib-0157] Nakamura M, Oshima A, Fujimoto Y, Maruyama N, Ishibashi T, Reeves KR. Efficacy and tolerability of nicotinic varenicline, an alpha4beta2 acetylcholine receptor partial agonist, in a 12-week, randomized, placebo-controlled, dose-response study with 40-week follow-up for smoking cessation in Japanese smokers. Clinical Therapeutics 2007;29(6):1040-56. [CENTRAL: 611262] [EMBASE: 2007380498] [PMID: 17692720] 17692720 10.1016/j.clinthera.2007.06.012

[CD006103-bib-0158] Nakamura M, Oshima A, Ohkura M, Arteaga C, Suwa K. Predictors of lapse and relapse to smoking in successful quitters in a varenicline post hoc analysis in Japanese smokers. Clinical Therapeutics 2014;36(6):918-27. [CENTRAL: 995377] [EMBASE: 2014542011] 10.1016/j.clinthera.2014.03.013 24811751

[CD006103-bib-0159] NCT01162239. Maintaining nonsmoking. ClinicalTrials.gov/show/NCT01162239 (first received 14 July 2010).

[CD006103-bib-0160] Niaura R, Taylor Hays J, Jorenby DE, Leone FT, Pappas JE, Reeves KR, et al. The efficacy and safety of varenicline for smoking cessation using a flexible dosing strategy in adult smokers: a randomized controlled trial. Current Medical Research and Opinion 2008;24(7):1931-41. [clinicaltrials.gov ID: NCT00150228] 10.1185/03007990802177523 18513462

[CD006103-bib-0161] Nides M, Glover ED, Reus VI, Christen AG, Make BJ, Billing CB, et al. Varenicline versus bupropion SR or placebo for smoking cessation: a pooled analysis. American Journal of Health Behavior 2008;32(6):664-75. 10.5555/ajhb.2008.32.6.664 18442345

[CD006103-bib-0162] Nides M, Oncken C, Gonzalez D, Rennard S, Watsky EJ, Anziano R, et al. Smoking cessation with varenicline, a selective α4β2 nicotinic receptor partial agonist. Archives of Internal Medicine 2006;166:1561-8. 10.1001/archinte.166.15.1561 16908788

[CD006103-bib-0163] Oncken C, Watsky E, Reeves K, Anziano R. Varenicline is efficacious and well tolerated in promoting smoking cessation: results from a 7-week, randomized, placebo- and bupropion-controlled trial [POS1-047]. Society for Research on Nicotine and Tobacco 11th Annual Meeting, 20-23 March 2005; Prague, Czech Republic 2005.

[CD006103-bib-0164] Bold KW, Zweben A, Fucito LM, Piepmeier ME, Muvvala S, Wu R, et al. Longitudinal findings from a randomized clinical trial of varenicline for alcohol use disorder with comorbid cigarette smoking. Alcoholism, Clinical and Experimental Research 2019;43(5):937-44. 10.1111/acer.13994 PMC7039307 30817018

[CD006103-bib-0165] NCT01553136. 1/2-multi-site study: varenicline treatment of alcohol dependent smokers. ClinicalTrials.gov/show/NCT01553136 (first received 13 March 2012).

[CD006103-bib-0166] O'Malley SS, Zweben A, Fucito LM, Wu R, Piepmeier ME, Ockert DM, et al. Effect of varenicline combined with medical management on alcohol use disorder with comorbid cigarette smoking: a randomized clinical trial. JAMA Psychiatry 2018;75(2):129-38. 10.1001/jamapsychiatry.2017.3544 PMC5838706 29261824

[CD006103-bib-0167] Oncken C, Gonzales D, Nides M, Rennard S, Watsky E, Billing CB, et al. Efficacy and safety of the novel selective nicotinic acetylcholine receptor partial agonist, varenicline, for smoking cessation. Archives of Internal Medicine 2006;166(15):1571-7. [CENTRAL: 567305] [EMBASE: 2006391945] [PMID: 16908789] 16908789 10.1001/archinte.166.15.1571

[CD006103-bib-0168] Oncken C, Watsky E, Reeves K, Anziano R. Smoking cessation with varenicline, a selective nicotinic receptor partial agonist: results from a phase 2 study [POS1-046]. Society for Research on Nicotine and Tobacco 11th Annual Meeting, 20-23 March 2005; Prague, Czech Republic 2005.

[CD006103-bib-0169] Pastorino U, Ladisa V, Trussardo S, Sabia F, Rolli L, Valsecchi C, et al. Cytisine therapy improved smoking cessation in the randomized SMILE lung cancer screening trial. Journal of Thoracic Oncology 2022;17(11):1276-86. [DOI: 10.1016/j.jtho.2022.07.007.] 35908731

[CD006103-bib-0170] ChiCTR1900021400. Individual tobacco cessation research based on nicotine metabolite ratio in smoking patients with chronic obstructive pulmonary disease: a randomized controlled trial. https://trialsearch.who.int/Trial2.aspx? TrialID=ChiCTR1900021400 (first received 19 February 2019).

[CD006103-bib-0171] Qin R, Liu Z, Zhou X, Cheng A, Cui Z, Li J, et al. Adherence and efficacy of smoking cessation treatment among patients with COPD in China. International Journal of Chronic Obstructive Pulmonary Disease 2021;16:1203-14. 10.2147/COPD.S301579 PMC8096422 33958864

[CD006103-bib-0172] Hughes JR, Russ C, Messig MA. Association of deferring a quit attempt with smoking cessation success: a secondary analysis. Journal of Substance Abuse Treatment 2014;46(2):264-7. [CENTRAL: 911546] [EMBASE: 2013742404] [PMID: 24074849] 24074849 10.1016/j.jsat.2013.08.015

[CD006103-bib-0173] Hughes JR, Russ CI, Arteaga CE, Rennard SI. Efficacy of a flexible quit date versus an a priori quit date approach to smoking cessation: a cross-study analysis. Addictive Behaviors 2011;36(12):1288-91. [CENTRAL: 830553] [PMID: 21872998] 21872998 10.1016/j.addbeh.2011.08.001

[CD006103-bib-0174] Rennard S, Hughes J, Cinciripini PM, Kralikova E, Raupach T, Arteaga C, et al. A randomized placebo-controlled trial of varenicline for smoking cessation allowing flexible quit dates. Nicotine & Tobacco Research 2012;14(3):343-50. [CENTRAL: 831332] [PMID: 22080588] 22080588 10.1093/ntr/ntr220 PMC3281242

[CD006103-bib-0175] Ockene I, Salmoirago-Blotcher E. Varenicline for smoking cessation in patients with coronary heart disease [editorial]. Circulation 2010;121(2):188-90. 10.1161/CIRCULATIONAHA.109.915546 20048200

[CD006103-bib-0176] Rigotti N, Pipe A, Benowitz N, Arteaga C, Garza D, Tonstad S, et al. A randomized trial of varenicline for smoking cessation in patients with cardiovascular disease: analysis of efficacy by baseline characteristics. Circulation 2010;Conference:2. 10.1161/CIRCULATIONAHA.109.869008 PMC4096941 20048210

[CD006103-bib-0177] Rigotti NA, Pipe AL, Benowitz NL, Arteaga C, Garza D, Tonstad S. Efficacy and safety of varenicline for smoking cessation in patients with cardiovascular disease. Circulation 2010;121(2):221-9. 10.1161/CIRCULATIONAHA.109.869008 PMC4096941 20048210

[CD006103-bib-0178] Rigotti NA, Pipe AL, Benowitz NL, Arteaga C, Garza D, Tonstad S. Response to letter regarding article, efficacy and safety of varenicline for smoking cessation in patients with cardiovascular disease: a randomized trial. Circulation 2010;122(9):e446. 10.1161/CIRCULATIONAHA.109.869008 PMC4096941 20048210

[CD006103-bib-0179] Martin RA, Rohsenow DJ, Tidey JW. Smokers with opioid use disorder may have worse drug use outcomes after varenicline than nicotine replacement. Journal of Substance Abuse Treatment 2019;104:22-7. 10.1016/j.jsat.2019.06.005 PMC6684327 31370981

[CD006103-bib-0180] NCT00756275. Varenicline and motivational advice for smokers with substance use disorders. ClinicalTrials.gov/ct2/show/NCT00756275 (first received 22 September 2008). [ClinicalTrials.gov ID NCT00756275]

[CD006103-bib-0181] Rohsenow D, Tidey JW, Martin RA, Colby S, Monti PM. Varenicline versus nicotine patch plus brief advice for sober smokers in substance treatment. Drug and Alcohol Dependence 2015;156:e191-2.

[CD006103-bib-0182] Rohsenow DJ, Tidey JW, Martin RA, Colby SM, Monti PM. Varenicline helps smokers with SUD stop smoking without harming recovery (POS5-63). Society for Research on Nicotine and Tobacco 21st Annual Meeting February 25-28 Philadelphia 2015.

[CD006103-bib-0183] Rohsenow DJ, Tidey JW, Martin RA, Colby SM, Swift RM, Leggio L, et al. Varenicline versus nicotine patch with brief advice for smokers with substance use disorders with or without depression: effects on smoking, substance use and depressive symptoms. Addiction 2017;112(10):1808-20. 10.1111/add.13861 PMC12435192 28498504

[CD006103-bib-0184] NCT00894166. Evaluation of a tailored smoking cessation treatment algorithm based on initial treatment response and genotype (ConNIC3). clinicaltrials.gov/ct2/show/NCT00894166 (first received 6 May 2009).

[CD006103-bib-0185] Rose JE, Behm FM. Adapting smoking cessation treatment according to initial response to precessation nicotine patch. American Journal of Psychiatry 2013;170(8):860-7. [CENTRAL: 873084] [EMBASE: 2013550171] [PMID: 23640009] 23640009 10.1176/appi.ajp.2013.12070919 PMC4562286

[CD006103-bib-0186] Uhl GR, Walther D, Musci R, Fisher C, Anthony JC, Storr CL, et al. Smoking quit success genotype score predicts quit success and distinct patterns of developmental involvement with common addictive substances. Molecular Psychiatry 2014;19(1):50-4. [PMID: 23128154] 23128154 10.1038/mp.2012.155 PMC3922203

[CD006103-bib-0187] Benndorf S, Kempe G, Scharfenberg G, Wendekamm R, Winkelvoss E. Results of smoking cessation treatment with cytisin (Tabex®) [Ergebnisse der medikamentösen raucherentwöhnung mit cytisin (Tabex®)]. Das Deutsche Gesundheitwesen 1968;23(44):2092-6. 5730918

[CD006103-bib-0188] Benndorf S, Scharfenberg G, Kempe G, Wendekamm R, Winkelvoss E. Smoking cessation treatment with cytisin (Tabex®): half-yearly outcomes for former smokers abstinent at four weeks from beginning of treatment [Medikamentöse raucherentwöhnung mit cytisin (Tabex®): ergebnisse der halbjahresbefragung bei den vier wochen nach kurbeginn abstinenten ehemaligen rauchern]. Das Deutsche Gesundheitwesen 1970;24:774-6. 5524300

[CD006103-bib-0189] Benndorf S, Scharfenberg G, Kempe G, Winkelvoss E, Wendekamm R. Further reports on a double blind trial of the Bulgarian cytisine compound Tabex® on 1214 smokers wishing to quit and practical experience in conducting clinics for such smokers [Weitere mitteilungen über einen doppelten blindversuch mit dem cytisinhaltigen bulgarischen präparat Tabex an 1214 entwöhnungswilligen rauchern und praktische erfahrungen bei der durchfürung einer sprechstunde für entwöhnungswillige raucher]. Das Deutsche Gesundheitswesen 1969;24:1135-40.

[CD006103-bib-0190] Scharfenberg G, Benndorf S, Kempe G. Cytisine (Tabex®) as a treatment for smoking cessation [Cytisin (Tabex®) als medikamentöse raucherentwöhnungshilfe]. Das Deutsche Gesundheitwesen 1971;26(10):463-5. 4930772

[CD006103-bib-0191] Carroll AJ, Veluz-Wilkins AK, Blazekovic S, Kalhan R, Leone FT, Wileyto El, et al. Cancer-related disease factors and smoking cessation treatment: analysis of an ongoing clinical trial. Psycho-oncology 2018;27(2):471-6. 10.1002/pon.4483 PMC5958302 28636795

[CD006103-bib-0192] Crawford G, Weisbrot J, Bastian J, Flitter A, Jao NC, Carroll A, et al. Predictors of varenicline adherence among cancer patients treated for tobacco dependence and its association with smoking cessation. Nicotine & Tobacco Research 2019;21(8):1135-9. 10.1093/ntr/nty133 PMC7182717 29955828

[CD006103-bib-0193] May JR, Jao NC, McCarter K, Klass E, Pearman T, Leone F, et al. Change in health-related quality of life among individuals with cancer undergoing smoking cessation treatment involving varenicline. Oncology Nursing Forum 2021;48(1):112-20. 10.1188/21.ONF.112-120 PMC7983103 33337436

[CD006103-bib-0194] NCT01756885. Extended duration varenicline for smoking among cancer patients: a clinical trial. ClinicalTrials.gov/show/NCT01756885 (first received 28 December 2012).

[CD006103-bib-0195] Schnoll R, Leone F, Veluz-Wilkins A, Miele A, Hole A, Jao N C, et al. A randomized controlled trial of 24 weeks of varenicline for tobacco use among cancer patients: efficacy, safety, and adherence. Psycho-oncology 2019;28(3):561-9. 10.1002/pon.4978 PMC6936106 30680852

[CD006103-bib-0196] De Dios MA, Anderson BJ, Caviness CM, Stein MD. Early quit days among methadone-maintained smokers in a smoking cessation trial. Nicotine & Tobacco Research 2014;16(11):1463-9. [CENTRAL: 1042497] 10.1093/ntr/ntu099 PMC4271088 24951495

[CD006103-bib-0197] NCT00790569. Varenicline versus nicotine replacement for methadone-maintained smokers. https://clinicaltrials.gov/ct2/show/NCT00790569 (first received 13 November 2008).

[CD006103-bib-0198] Stein MD, Caviness CM, Kurth ME, Audet D, Olson J, Anderson BJ. Varenicline for smoking cessation among methadone-maintained smokers: a randomized clinical trial. Drug and Alcohol Dependence 2013;133(2):486-93. [CENTRAL: 870955] [EMBASE: 2013694276] [PMID: 23953658] 23953658 10.1016/j.drugalcdep.2013.07.005 PMC3818400

[CD006103-bib-0199] Steinberg MB, Randall J, Greenhaus S, Schmelzer AC, Richardson DL, Carson JL. Tobacco dependence treatment for hospitalized smokers: a randomized, controlled, pilot trial using varenicline. Addictive Behaviors 2011;36(12):1127-32. 10.1016/j.addbeh.2011.07.002 21835552

[CD006103-bib-0200] NCT01308736. Varenicline-aided cigarette reduction in smokers not ready to quit. ClinicalTrials.gov/show/NCT01308736 (first received 4 March 2011).

[CD006103-bib-0201] Steinberg ML, Lu SE, Williams JM. Varenicline for smoking reduction in smokers not yet ready to quit: a double-blind, proof-of-concept randomized clinical trial. Addictive Behaviors 2018;84:20-6. 10.1016/j.addbeh.2018.03.026 29604474

[CD006103-bib-0202] Antoniu SA, Trofor AC. Varenicline for smoking cessation intervention in chronic obstructive pulmonary disease. Expert Opinion on Pharmacotherapy 2011;12(16):2595-7. 10.1517/14656566.2011.605786 21819275

[CD006103-bib-0203] Kotz D, Van Schayck OC. What justifies a placebo-controlled trial of varenicline for smoking cessation in patients with COPD? [comment]. Chest 2011;139:968-9. 10.1378/chest.10-2919 21467069

[CD006103-bib-0204] Tashkin DP, Rennard S, Hays JT, Ma W, Lawrence D, Lee TC. Effects of varenicline on smoking cessation in mild-to-moderate COPD: a randomized controlled trial. Chest 2011;139:591-9. 10.1378/chest.10-0865 20864613

[CD006103-bib-0205] Tashkin DP, Rennard S, Taylor Hays J, Lawrence D, Marton JP, Lee TC. Lung function and respiratory symptoms in a 1-year randomized smoking cessation trial of varenicline in COPD patients. Respiratory Medicine 2011;105(11):1682-90. [CENTRAL: 811557] [6378] [PMID: 21621992] 21621992 10.1016/j.rmed.2011.04.016

[CD006103-bib-0206] Tonstad S, Holme I, Tønnesen P. Dianicline, a novel α4β2 nicotinic acetylcholine receptor partial agonist, for smoking cessation: a randomized placebo-controlled clinical trial. Nicotine & Tobacco Research 2011;13(1):1-6. 10.1093/ntr/ntq191 21041839

[CD006103-bib-0207] Fagerström K, Nakamura M, Cho H, Tsa S, Wang C, Davies S, et al. Varenicline treatment for smoking cessation in Asian populations: a pooled analysis of placebo-controlled trials conducted in six Asian countries. Current Medical Research & Opinion 2010;26(9):2165-73. 10.1185/03007995.2010.505130 20666691

[CD006103-bib-0208] Tsai S-T, Cho H-J, Cheng H-S, Kim C-H, Hsueh K-C, Billing CB, et al. A randomized, placebo-controlled trial of varenicline, a selective α4β2 nicotinic acetylcholine receptor partial agonist, as a new therapy for smoking cessation in Asian smokers. Clinical Therapeutics 2007;29(6):1027-39. 10.1016/j.clinthera.2007.06.011 17692719

[CD006103-bib-0209] Fujiwara H. Smoking is a disease and smokers are patients. Circulation Journal 2010;74(4):628-9. 10.1253/circj.cj-10-0165 20234103

[CD006103-bib-0210] Tsukahara H, Noda K, Keijiro S. A randomized controlled open comparative trial of varenicline vs nicotine patch in adult smokers. Circulation Journal 2010;74(4):771-8. 10.1253/circj.cj-09-0803 20154405

[CD006103-bib-0211] NCT01531049. Smoking cessation in young adults in Northern Finland. ClinicalTrials.gov/show/NCT01531049 (first received 10 February 2012).

[CD006103-bib-0212] Tuisku A, Salmela M, Nieminen P, Toljamo T. Varenicline and nicotine patch therapies in young adults motivated to quit smoking: a randomized, placebo-controlled, prospective study. Basic & Clinical Pharmacology & Toxicology 2016;119(1):78-84. 10.1111/bcpt.12548 26709238

[CD006103-bib-0213] Clyde M, Pipe A, Els C, Reid R, Fu A, Clark A, et al. Nicotine metabolite ratio and smoking outcomes using nicotine replacement therapy and varenicline among smokers with and without psychiatric illness. Journal of Psychopharmacology 2018;32(9):979-85. 10.1177/0269881118773532 29788791

[CD006103-bib-0214] Clyde M, Pipe A, Els C, Reid R, Tulloch H. Factor structure of the Smoking Cessation Self-Efficacy Questionnaire among smokers with and without a psychiatric diagnosis. Psychology of Addictive Behaviors 2017;31(2):162-70. 10.1037/adb0000250 28182446

[CD006103-bib-0215] NCT01623505. Reducing cardiovascular disease by combining smoking cessation pharmacotherapy and behavioural counselling. clinicaltrials.gov/ct2/show/NCT01623505 (first received 20 June 2012).

[CD006103-bib-0216] Tulloch H, Pipe A, Els C, Aitken D, Clyde M, Corran B, et al. Flexible and extended dosing of nicotine replacement therapy or varenicline in comparison to fixed dose nicotine replacement therapy for smoking cessation: rationale, methods and participant characteristics of the FLEX trial. Contemporary Clinical Trials 2014;38(2):304-13. [CENTRAL: 999078] [EMBASE: 2014493992] 10.1016/j.cct.2014.05.011 24861558

[CD006103-bib-0217] Tulloch HE, Pipe AL, Clyde MJ, Reid RD, Els C. The quit experience and concerns of smokers with psychiatric illness. American Journal of Preventive Medicine 2016;50(6):709-18. 10.1016/j.amepre.2015.11.006 26711162

[CD006103-bib-0218] Tulloch HE, Pipe AL, Els C, Clyde MJ, Reid RD. Flexible, dual-form nicotine replacement therapy or varenicline in comparison with nicotine patch for smoking cessation: a randomized controlled trial. BMC Medicine 2016;14(1):80. 10.1186/s12916-016-0626-2 PMC4884360 27233840

[CD006103-bib-0219] Zhang KM, Clyde M, Pipe A, Reid R, Els C, Tulloch HE. Do women and men differ in baseline smoking characteristics and quit rates following treatment with smoking cessation medications? A secondary analysis of the flex study. Journal of Cardiopulmonary Rehabilitation and Prevention. Conference: 2018 Fall Conference of the Canadian Association of Cardiovascular Prevention and Rehabilitation, CACPR 2018. Canada 2018;38(6):E21.

[CD006103-bib-0220] Vinnikov D, Brimkulov N, Burjubaeva A. A double-blind, randomised, placebo-controlled trial of cytisine for smoking cessation in medium-dependent workers. Journal of Smoking Cessation 2008;3(1):57-62.

[CD006103-bib-0221] Walker N, Howe C, Bullen C, McRobbie H, Glover M, Parag V, et al. Study protocol for a non-inferiority trial of cytisine versus nicotine replacement therapy in people motivated to stop smoking. BMC Public Health 2011;11(1):880. [CENTRAL: 814611] [EMBASE: 22104038] [6430] [PMID: 22104038] 22104038 10.1186/1471-2458-11-880 PMC3234196

[CD006103-bib-0222] Walker N, Howe C, Glover M, McRobbie H, Barnes J, Nosa V, et al. Cytisine versus nicotine for smoking cessation. New England Journal of Medicine 2014;371(25):2353-62. [CENTRAL: 1037839] [EMBASE: 2014610901] 10.1056/NEJMoa1407764 25517706

[CD006103-bib-0223] NCT02957786. Cytisine versus varenicline for smoking cessation. clinicaltrials.gov/show/NCT02957786 (first received 8 November 2016).

[CD006103-bib-0224] Walker N, Smith B, Barnes J, Verbiest M, Kurdziel T, Parag V, et al. Cytisine versus varenicline for smoking cessation for Maori (the indigenous people of New Zealand) and their extended family: protocol for a randomized non-inferiority trial. Addiction 2019;114(2):344-52. 10.1111/add.14449 PMC6587772 30276931

[CD006103-bib-0225] Walker N, Smith B, Barnes J, Verbiest M, Parag V, Pokhrel S, et al. Cytisine versus varenicline for smoking cessation in New Zealand indigenous Maori: a randomized controlled trial. Addiction 2021;116(10):2847-58. 10.1111/add.15489 PMC8519028 33761149

[CD006103-bib-0226] Fagerström K, Nakamura M, Cho H, Tsa S, Wang C, Davies S, et al. Varenicline treatment for smoking cessation in Asian populations: a pooled analysis of placebo-controlled trials conducted in six Asian countries. Current Medical Research & Opinion 2010;26(9):2165-73. 10.1185/03007995.2010.505130 20666691

[CD006103-bib-0227] Wang C, Xiao D, Chan KP, Pothirat C, Garza D, Davies S. Varenicline for smoking cessation: a placebo-controlled, randomized study. Respirology 2009;14(3):384-92. [clinicaltrials.gov ID: NCT00371813] 10.1111/j.1440-1843.2008.01476.x 19192221

[CD006103-bib-0228] West R, Zatonski W, Cedzynska M, Lewandowska D, Pazik J, Aveyard PA, et al. Placebo-controlled trial of cytisine for smoking cessation. New England Journal of Medicine 2011;365:1193-200. [Current Controlled Trials ID: ISRCTN37568749] 10.1056/NEJMoa1102035 21991893

[CD006103-bib-0229] West R, Zatonski W. Cytisine increased smoking cessation in adults. Annals of Internal Medicine 2012;156(2):JC1-6. [CENTRAL: 897954] [EMBASE: 2012033460] 10.7326/0003-4819-156-2-201201170-02006 22250168

[CD006103-bib-0230] Westergaard CG, Porsbjerg C, Backer V. The effect of smoking cessation on airway inflammation in young asthma patients. Clinical & Experimental Allergy 2013;44:353-61. 10.1111/cea.12243 24286379

[CD006103-bib-0231] Westergaard CG, Porsbjerg C, Backer V. The effect of varenicline on smoking cessation in a group of young asthma patients. American Journal of Respiratory and Critical Care Medicine 2014;189:A1091. 10.1016/j.rmed.2015.07.017 26427627

[CD006103-bib-0232] Westergaard CG, Porsbjerg C, Backer V. The effect of varenicline on smoking cessation in a group of young asthma patients. Respiratory Medicine 2015;109(11):1416-22. [DOI: 10.1016/j.rmed.2015.07.017] 26427627

[CD006103-bib-0233] Reeves K, Watsky E, Williams K, Azoulay S, Billing B, Gong J. The safety of varenicline taken for 52 weeks for smoking cessation [RPOS3-54]. Society for Research on Nicotine and Tobacco 12th Annual Conference Orlando Fla, USA 2006.

[CD006103-bib-0234] Spangler JG, Williams KE, Reeves KR, Billing CB, Pennington AM, Gong J. Comment and reply: a double-blind study evaluating the long-term safety of varenicline for smoking cessation. Current Medical Research and Opinion 2008;24(2):577-9. 10.1185/030079908x261140 18198011

[CD006103-bib-0235] Williams KE, Reeves KR, Billing CB, Pennington AM, Gong J. A double-blind study evaluating the long-term safety of varenicline for smoking cessation. Current Medical Research and Opinion 2007;23(4):793-801. 10.1185/030079907x182185 17407636

[CD006103-bib-0236] NCT00644969. Smoking cessation study for patients with schizophrenia or schizoaffective disorder. clinicaltrials.gov/ct2/show/NCT00644969 (first received 27 March 2008).

[CD006103-bib-0237] Williams JM, Anthenelli RM, Morris CD, Treadow J, Thompson JR, Yunis C, et al. A randomized, double-blind, placebo-controlled study evaluating the safety and efficacy of varenicline for smoking cessation in patients with schizophrenia or schizoaffective disorder. Journal of Clinical Psychiatry 2012;73(5):654-60. [CENTRAL: 836870] [EMBASE: 2012321245] [PMID: 22697191] 22697191 10.4088/JCP.11m07522

[CD006103-bib-0238] Dehghani P, Habib B, Windle SB, Roy N, Old W, Grondin FR, et al. Smokers and postcessation weight gain after acute coronary syndrome. Journal of the American Heart Association 2017;6(4):e004785. 10.1161/JAHA.116.004785 PMC5532997 28420644

[CD006103-bib-0239] Eisenberg MJ, Windle SB, Roy N, Old W, Grondin F, Bata I, et al. Varenicline for smoking cessation in hospitalized patients with acute coronary syndrome. Circulation 2016;133(1):21-30. 10.1161/CIRCULATIONAHA.115.019634 26553744

[CD006103-bib-0240] NCT00794573. Evaluation of varenicline (Champix) in smoking cessation for patients post-acute coronary syndrome (EVITA) trial (EVITA). clinicaltrials.gov/ct2/show/NCT00794573 (first received 20 November 2008).

[CD006103-bib-0241] Windle SB, Bata I, Madan M, Abramson BL, Eisenberg MJ. A randomized controlled trial of the efficacy and safety of varenicline for smoking cessation after acute coronary syndrome: design and methods of the Evaluation of Varenicline in Smoking Cessation for Patients Post-Acute Coronary Syndrome trial. American Heart Journal 2015;170(4):635-40. 10.1016/j.ahj.2015.07.010 26386786

[CD006103-bib-0242] Windle SB, Dehghani P, Roy N, Old W, Grondin FR, Bata I, et al. Smoking abstinence 1 year after acute coronary syndrome: follow-up from a randomized controlled trial of varenicline in patients admitted to hospital. CMAJ : Canadian Medical Association Journal 2018;190(12):E347-54. 10.1503/cmaj.170377 PMC5871438 29581161

[CD006103-bib-0243] Windle SB, Dehghani P, Roy N, Old WD, Grondin FR, Bata I, et al. Sustained smoking abstinence 12 months after acute coronary syndrome: follow-up from a randomized controlled trial of varenicline in hospitalized patients. Canadian Journal of Cardiology. Conference: 69th Annual Meeting of the Canadian Cardiovascular Society. Canada. 2016;10 Suppl 1:S109-10.

[CD006103-bib-0244] NCT00937508. Smoking cessation program in the pre-admission clinic. clinicaltrials.gov/ct2/show/NCT00937508 (first received 13 July 2009).

[CD006103-bib-0245] Wong J, Abrishami A, Yang Y, Zaki A, Friedman Z, Selby P, et al. A perioperative smoking cessation intervention with varenicline: a double-blind, randomized, placebo-controlled trial. Anesthesiology 2012;117(4):755-64. [CENTRAL: 835869] [EMBASE: 2012567287] [PMID: 22890119] 22890119 10.1097/ALN.0b013e3182698b42

[CD006103-bib-0246] Yang DX, Gu CJ, Ni L, Li N, Li QY, Zhou JP. Assessment of efficacy of medication combined with WeChat platform for quitting smoking in patients with chronic obstructive pulmonary disease. Journal of Shanghai Jiaotong University (Medical Science) 2016;36(3):385-9.

[CD006103-bib-0247] NCT01286584. Varenicline in residential treatment (ViRT). clinicaltrials.gov/ct2/show/NCT01286584 (first received 31 January 2011).

[CD006103-bib-0248] Zawertailo L, Ivanova A, Ng G, Le Foll B, Selby P. Safety and efficacy of varenicline for smoking cessation in alcohol-dependent smokers in concurrent treatment for alcohol use disorder: a pilot, randomized placebo-controlled trial. Journal of Clinical Psychopharmacology 2020;40(2):130-6. 10.1097/JCP.0000000000001172 32068562

[CD006103-bib-0249] NCT02146911. The MATCH (Medication Aids for Tobacco Cessation and Health) study. clinicaltrials.gov/show/NCT02146911 (first received 26 May 2014).

[CD006103-bib-0250] Zawertailo L, Mansoursadeghi-Gilan T, Zhang H, Hussain S, Le Foll B, Selby P. Varenicline and bupropion for long-term smoking cessation (the MATCH Study): protocol for a real-world, pragmatic, randomized controlled trial. JMIR Research Protocols 2018;7(10):e10826. 10.2196/10826 PMC6231835 30341043

[CD006103-bib-0251] Zhang H, Mansoursadeghi-Gilan T, Hussain S, Veldhuizen S, Le Foll B, Selby P, et al. Evaluating the effectiveness of bupropion and varenicline for smoking cessation using an internet-based delivery system: a pragmatic randomized controlled trial (MATCH study). Drug and Alcohol Dependence 2022;232:109312. 10.1016/j.drugalcdep.2022.109312 35151504

[CD006103-bib-0252] Zincir SB, Zincir S, Kaymak E, Sunbul EA. Comparison of the effectiveness of varenicline, extended-release bupropion and nicotine replacement therapy on the success and the maintenance of a smoking cessation program. Bulletin of Clinical Psychopharmacology 2013;23(3):224-30. [CENTRAL: 914104] [EMBASE: 2013628205]

[CD006103-bib-0253] Brandon TH, Drobes DJ, Unrod M, Heckman BW, Oliver JA, Roetzheim RC, et al. Varenicline effects on craving, cue reactivity, and smoking reward. Psychopharmacology 2011;218(2):391-403. 10.1007/s00213-011-2327-z PMC4667942 21559801

[CD006103-bib-0254] Bullen C, Verbiest M, Galea-Singer S, Kurdziel T, Laking G, Newcombe D, et al. The effectiveness and safety of combining varenicline with nicotine e-cigarettes for smoking cessation in people with mental illnesses and addictions: study protocol for a randomised-controlled trial. BMC Public Health 2018;18(1):596. 10.1186/s12889-018-5351-7 PMC5935940 29728074

[CD006103-bib-0255] Burstein AH, Fullerton T, Clark DJ, Faessel HM. Pharmacokinetics, safety, and tolerability after single and multiple oral doses of varenicline in elderly smokers. Journal of Clinical Pharmacology 2006;46:1234-40. 10.1177/0091270006291837 17050788

[CD006103-bib-0256] Pfizer Inc. Chantix prescribing information. www.chantix.com/content/Prescribing_Information.jsp (accessed 10 February 2007).

[CD006103-bib-0257] Cui Q, Robinson L, Elston D, Smaill F, Cohen J, Quan C, et al. Safety and tolerability of varenicline tartrate (Champix^®^/Chantix^®^) for smoking cessation in HIV-infected subjects: a pilot open-label study. AIDS Patient Care and STDs 2012;26(1):12-19. [DOI: 10.1089/apc.2011.0199] PMC3242617 22007690

[CD006103-bib-0258] Cowan CM, Wink JS, DeZee KJ. Use of the patient health questionnaire-2 to predict suicidal ideations in patients taking varenicline. American Journal on Addictions / American Academy of Psychiatrists in Alcoholism and Addictions 2012;21(4):356-62. [PMID: 22691015] 22691015 10.1111/j.1521-0391.2012.00243.x

[CD006103-bib-0259] Dezee KJ, Wink JS, Cowan CM. Internet versus in-person counseling for patients taking varenicline for smoking cessation. Military Medicine 2013;178(4):401-5. [CENTRAL: 959429] [PMID: 23707824] 23707824 10.7205/MILMED-D-12-00272

[CD006103-bib-0260] Dutra SJ, Stoeckel LE, Carlini SV, Pizzagalli DA, Evins AE. Varenicline as a smoking cessation aid in schizophrenia: effects on smoking behavior and reward sensitivity. Psychopharmacology 2012;219(1):25-34. [DOI: 10.1007/s00213-011-2373-6] PMC3267781 21695488

[CD006103-bib-0261] Ebbert JO, Croghan IT, Sood A, Schroeder DR, Hays JT, Hurt RD. Varenicline and bupropion sustained-release combination therapy for smoking cessation. Nicotine & Tobacco Research 2009;11(3):234-9. 10.1093/ntr/ntn031 19246427

[CD006103-bib-0262] Ebbert JO, Burke MV, Hays JT, Hurt RD. Combination treatment with varenicline and nicotine replacement therapy. Nicotine & Tobacco Research 2009;11(5):572-6. 10.1093/ntr/ntp042 19351781

[CD006103-bib-0263] Ebbert JO, Croghan IT, North F, Schroeder DR. A pilot study to assess smokeless tobacco use reduction with varenicline. Nicotine & Tobacco Research 2011;13(9):820-6. 10.1093/ntr/ntq134 PMC2948050 20724382

[CD006103-bib-0264] Ebbert JO, Hatsukami DK, Croghan IT, Schroeder DR, Allen SS, Hays JT, et al. Combination varenicline and bupropion SR for tobacco-dependence treatment in cigarette smokers: a randomized trial. JAMA 2014;311(2):155-63. [CENTRAL: 921055] [EMBASE: 2014041620] [PMID: 24399554] 24399554 10.1001/jama.2013.283185 PMC3959999

[CD006103-bib-0265] NCT00935818. Varenicline and bupropion for smoking cessation (CHANBAN). clinicaltrials.gov/ct2/show/NCT00935818 (first received 9 July 2009).

[CD006103-bib-0266] Cather C, Hoeppner S, Pachas G, Pratt S, Achtyes E, Cieslak KM, et al. Improved depressive symptoms in adults with schizophrenia during a smoking cessation attempt with varenicline and behavioral therapy. Journal of Dual Diagnosis 2017;13(3):168-78. 10.1080/15504263.2017.1319585 PMC12817128 28414583

[CD006103-bib-0267] Evins AE, Cather C, Pratt SA, Pachas GN, Hoeppner SS, Goff DC, et al. Maintenance treatment with varenicline for smoking cessation in patients with schizophrenia and bipolar disorder: a randomized clinical trial. JAMA 2014;311(2):145-54. [CENTRAL: 921056] [EMBASE: 2014041619] [PMID: 24399553] 24399553 10.1001/jama.2013.285113 PMC4124884

[CD006103-bib-0268] Evins AE. Extended duration pharmacotherapy with varenicline prevents relapse to smoking in adult smokers with schizophrenia. Neuropsychopharmacology 2013;38:S63-4. [CENTRAL: 993941] [EMBASE: 71278012]

[CD006103-bib-0269] NCT00621777. A study of varenicline for prevention of relapse to smoking in patients with schizophrenia. clinicaltrials.gov/ct2/show/NCT00621777 (first received 22 February 2008).

[CD006103-bib-0270] Pachas GN, Cather C, Pratt SI, Hoeppner B, Nino J, Carlini SV, et al. Varenicline for smoking cessation in schizophrenia: safety and effectiveness in a 12-week open-label trial. Journal of Dual Diagnosis 2012;8(2):117-25. 10.1080/15504263.2012.663675 PMC3414422 22888309

[CD006103-bib-0271] Thorndike AN, Achtyes ED, Cather C, Pratt S, Pachas GN, Hoeppner SS, et al. Weight gain and 10-year cardiovascular risk with sustained tobacco abstinence in smokers with serious mental illness: a subgroup analysis of a randomized trial. Journal of Clinical Psychiatry 2016;77(3):e320-6. 10.4088/JCP.15m10074 PMC12810507 27046320

[CD006103-bib-0272] Thorndike AN, Hoeppner SS, Cather C, Pachas GN, Achtyes ED, Evins AE. Weight gain and cardiovascular risk reduction associated with tobacco abstinence in smokers with serious mental illness. Circulation 2015;131:AP008. 10.4088/JCP.15m10074 PMC12810507 27046320

[CD006103-bib-0273] Faessel H, Ravva P, Williams K. Pharmacokinetics, safety, and tolerability of varenicline in healthy adolescent smokers: a multicenter, randomized, double-blind, placebo-controlled, parallel-group study. Clinical Therapeutics 2009;31:177-89. [clinicaltrials.gov ID: NCT00463918] 10.1016/j.clinthera.2009.01.003 19243716

[CD006103-bib-0274] Fagerström K, Gilljam H, Metcalfe M, Tonstad S, Messig M. Stopping smokeless tobacco with varenicline: randomised double blind placebo controlled trial. BMJ 2010;341:c6549. [DOI: 10.1136/bmjc6549] PMC2997603 21134997

[CD006103-bib-0275] Falk DE, Litten RZ, Ryan ML, Fertig JB. A double-blind, placebo-controlled trial assessing the efficacy of varenicline tartrate for alcohol dependence. Alcoholism, Clinical and Experimental Research 2014;38 Suppl s1:139A. [CENTRAL: 993967] [EMBASE: 71503638]

[CD006103-bib-0276] Litten RZ, Ryan ML, Fertig JB, Falk DE, Johnson B, Dunn KE, et al. A double-blind, placebo-controlled trial assessing the efficacy of varenicline tartrate for alcohol dependence. Journal of Addiction Medicine 2013;7(4):277-86. 10.1097/ADM.0b013e31829623f4 PMC3914416 23728065

[CD006103-bib-0277] Fatemi SH, Yousefi MK, Kneeland RE, Liesch SB, Folsom TD, Thuras PD. Antismoking and potential antipsychotic effects of varenicline in subjects with schizophrenia or schizoaffective disorder: a double-blind placebo and bupropion-controlled study. Schizophrenia Research 2013;146(1-3):376-8. [CENTRAL: 877011] [PMID: 23507358] 23507358 10.1016/j.schres.2013.02.015

[CD006103-bib-0278] NCT01111149. Varenicline and smoking cessation in schizophrenia (VSCS). clinicaltrials.gov/ct2/show/NCT01111149 (first received 27 April 2010).

[CD006103-bib-0279] Ferketich AK, Otterson GA, King M, Hall N, Browning KK, Wewers ME. A pilot test of a combined tobacco dependence treatment and lung cancer screening program. Lung Cancer 2012;76(2):211-5. [CENTRAL: 814368] [EMBASE: 2012198269] [PMID: 22088938] 22088938 10.1016/j.lungcan.2011.10.011 PMC4272196

[CD006103-bib-0280] Ferketich AK, Diaz P, Browning KK, Lu B, Koletar SL, Reynolds NR, et al. Safety of varenicline among smokers enrolled in the lung HIV study. Nicotine & Tobacco Research 2013;15(1):247-54. [EMBASE: 2012756151] [PMID: 22589421] 22589421 10.1093/ntr/nts121 PMC3524069

[CD006103-bib-0281] Fertig JB, Ryan ML, Falk DE, Litten RZ. Moderators of the varenicline treatment effect in a double-blind, placebo controlled trial for alcohol dependence. Alcoholism: Clinical and Experimental Research 2015;39:80A. 10.1097/ADM.0000000000000133 PMC4523402 26083958

[CD006103-bib-0282] Frye MA, Ebbert JO, Prince CA, Lineberry TW, Geske JR, Patten CA. A feasibility study of varenicline for smoking cessation in bipolar patients with subsyndromal depression. Journal of Clinical Psychopharmacology 2013;33(6):821-3. [PMID: 23963060] 23963060 10.1097/JCP.0b013e31829cc3cd

[CD006103-bib-0283] Fucito LM, Toll BA, Wu R, Romano DM, Tek C, O'Malley SS. A preliminary investigation of varenicline for heavy drinking smokers. Psychopharmacology 2011;215(4):655-63. 10.1007/s00213-010-2160-9 PMC3645986 21221531

[CD006103-bib-0284] Garza D, Murphy M, Tseng L-J, Riordan HJ, Chatterjee A. A double-blind randomized placebo-controlled pilot study of neuropsychiatric adverse events in abstinent smokers treated with varenicline or placebo. Biological Psychiatry 2011;69:1075-82. 10.1016/j.biopsych.2010.12.005 21295286

[CD006103-bib-0285] Granatowicz J. Smoking cessation through the use of cytisine and other therapy. World Smoking Health 1976;1:8-11.

[CD006103-bib-0286] Gray KM, Carpenter MJ, Lewis AL, Klintworth EM, Upadhyaya HP. Varenicline versus bupropion XL for smoking cessation in older adolescents: a randomized, double-blind pilot trial. Nicotine & Tobacco Research 2012;14(2):235-9. [CENTRAL: 830824] [EMBASE: 2012065214] [PMID: 21778151] 21778151 10.1093/ntr/ntr130 PMC3265741

[CD006103-bib-0287] Dhelaria RK, Rothberg M. Is varenicline effectiveness declining in randomized trials? Archives of Internal Medicine 2011;171(19):1770-1. 10.1001/archinternmed.2011.493 22025439

[CD006103-bib-0288] Hajek P, McRobbie H, Myers K, Stapleton J, Dhanji AR. Is varenicline effectiveness declining in randomized trials? - Reply. Archives of Internal Medicine 2011;171(19):1771-2. 10.1001/archinternmed.2011.493 22025439

[CD006103-bib-0289] Hajek P, McRobbie HJ, Myers KE, Stapleton J, Dhanji A-R. Use of varenicline for 4 weeks before quitting smoking. Archives of Internal Medicine 2011;171(8):770-7. 10.1001/archinternmed.2011.138 21518946

[CD006103-bib-0290] Simon JA. Smoking cessation interventions: a primer for physicians. Archives of Internal Medicine 2011;171(8):777-8. 10.1001/archinternmed.2011.137 21518947

[CD006103-bib-0291] Hajek P, Smith KM, Dhanji A-R, McRobbie H. Is a combination of varenicline and nicotine patch more effective in helping smokers quit than varenicline alone? A randomised controlled trial. BMC Medicine 2013;11(1):140. [CENTRAL: 963647] [EMBASE: 2013375051] [PMID: 23718718] 23718718 10.1186/1741-7015-11-140 PMC4231450

[CD006103-bib-0292] Hajek P, McRobbie H, Myers Smith K, Phillips A, Cornwall D, Dhanji AR. Erratum: increasing varenicline dose in smokers WHO do not respond to the standard dosage: a randomized clinical trial (JAMA internal medicine (2015) 175: 2 (266-271)). JAMA Internal Medicine 2016;176(1):143. 10.1001/jamainternmed.2014.6916 25545858

[CD006103-bib-0293] Hajek P, McRobbie H, Myers Smith K, Phillips A, Cornwall D, Dhanji AR. Increasing varenicline dose in smokers who do not respond to the standard dosage: a randomized clinical trial. JAMA Internal Medicine 2015;175(2):266-71. [CENTRAL: 1047635] [EMBASE: 2015718793] [PMID: 25545858] 25545858 10.1001/jamainternmed.2014.6916

[CD006103-bib-0294] Hartwell EE, Roche DJ, Ray LA. Pharmacogenetics of naltrexone and varenicline in heavy drinking smokers. Alcoholism, Clinical and Experimental Research 2014;38 Suppl s1:223A. [CENTRAL: 993964] [EMBASE: 71503974]

[CD006103-bib-0295] Roche DJ, Bujarski S, Hartwell E, Green R, Ray LA. Combined varenicline and naltrexone treatment reduces smoking topography intensity in heavy-drinking smokers. Pharmacology, Biochemistry, and Behavior 2015;134:92-8. 10.1016/j.pbb.2015.04.013 PMC4457679 25933795

[CD006103-bib-0296] Hawk LW, Ashare RL, Lohnes SF, Schlienz NJ, Rhodes JD, Tiffany ST, et al. The effects of extended pre-quit varenicline treatment on smoking behavior and short-term abstinence: a randomized clinical trial. Clinical Pharmacology and Therapeutics 2012;91(2):172-80. [CENTRAL: 814229] [PMID: 22130118] 22130118 10.1038/clpt.2011.317 PMC3325094

[CD006103-bib-0297] NCT00835900. An alternative dosing schedule for varenicline for smoking cessation. clinicaltrials.gov/ct2/show/NCT00835900 (first received 4 February 2009).

[CD006103-bib-0298] Hong LE, Thaker GK, McMahon RP, Summerfelt A, Rachbeisel J, Fuller RL, et al. Effects of moderate-dose treatment with varenicline on neurobiological and cognitive biomarkers in smokers and nonsmokers with schizophrenia or schizoaffective disorder. Archives of General Psychiatry 2011;68(12):1195-206. [CENTRAL: 810278] [6348] [PMID: 21810630] 21810630 10.1001/archgenpsychiatry.2011.83 PMC3618988

[CD006103-bib-0299] NCT00492349. Varenicline adjunctive treatment in schizophrenia. clinicaltrials.gov/ct2/show/NCT00492349 (first received 27 June 2007).

[CD006103-bib-0300] Hoogsteder PH, Kotz D, Van Spiegel PI, Viechtbauer W, Van Schayck OC. Efficacy of the nicotine vaccine 3'-AmNic-rEPA (NicVAX) co-administered with varenicline and counselling for smoking cessation: a randomized placebo-controlled trial. Addiction 2014;109(8):1252-9. [CENTRAL: 1000793] [PMID: 24894625] 24894625 10.1111/add.12573

[CD006103-bib-0301] Hsueh K-C, Hsueh S-C, Chou M-Y, Pan L-F, Tu M-S, McEwen A, et al. Varenicline versus transdermal nicotine patch: a 3-year follow-up in a smoking cessation clinic in Taiwan. Psychopharmacology 2014;231(14):2819-23. [CENTRAL: 1050988] [EMBASE: 2014439532] [PMID: 24522334] 24522334 10.1007/s00213-014-3482-9

[CD006103-bib-0302] Hsueh KC, Tang PL, McRobbie H. Effectiveness of varenicline versus combination nicotine replacement therapy for smoking cessation: one-year outcomes in a smoking cessation clinic in Taiwan. Nicotine & Tobacco Research 2021;23(7):1094-102. 10.1093/ntr/ntab018 33538831

[CD006103-bib-0303] Hughes JR, Rennard SI, Fingar JR, Talbot SK, Callas PW, Fagerström KO. Efficacy of varenicline to prompt quit attempts in smokers not currently trying to quit: a randomized placebo-controlled trial. Nicotine & Tobacco Research 2011;13(10):955-64. 10.1093/ntr/ntr103 PMC3218639 21652735

[CD006103-bib-0304] NCT00595868. Efficacy of varenicline in ambivalent smokers. ClinicalTrials.gov/ct2/show/NCT00595868 2007 (accessed 2 January 2016).

[CD006103-bib-0305] IRCT20100127003210N. Cytisine versus nicotine for smoking cessation in hospitalized psychiatric patients. http://www.who.int/trialsearch/Trial2.aspx?TrialID=IRCT20100127003210N16 (first received 2019).

[CD006103-bib-0306] Jain R, Jhanjee S, Jain V, Gupta T, Mittal S, Goelz P, et al. A double-blind placebo-controlled randomized trial of varenicline for smokeless tobacco dependence in India. Nicotine & Tobacco Research 2014;16(1):50-7. [CENTRAL: 1000326] [PMID: 23946326] 23946326 10.1093/ntr/ntt115 PMC3864491

[CD006103-bib-0307] Jennings C, Kotseva K, De Bacquer D, Hoes A, Velasco J de, Brusaferro S, et al. Effectiveness of a preventive cardiology programme for high CVD risk persistent smokers: the EUROACTION PLUS varenicline trial. European Heart Journal 2014;35(21):1411-20. [CENTRAL: 996426] [EMBASE: 2014386940] [PMID: 24616337] 24616337 10.1093/eurheartj/ehu051

[CD006103-bib-0308] Jiménez-Ruiz CA, Barrios M, Peña S, Cicero A, Mayayo M, Cristóbal M, et al. Increasing the dose of varenicline in patients who do not respond to the standard dose. Mayo Clinic Proceedings 2013;88(12):1443-5. 10.1016/j.mayocp.2013.08.015 24290118

[CD006103-bib-0309] Bacvarov VI. Smoking cessation through medication: remarks on G. Scharfenberg, E Winkelvoss and S. Benndorf, Munch Med Wschr. 109 (1967) 1687-1689. Munchener Medizinische Wochenschrift 1967;109(50):2663-5. 5632012

[CD006103-bib-0310] Kempe G. Observation about the Bulgarian medicine for smoking withdrawal Tabex, produced by Pharmachim-Sofia. Savr Med 1967;18(4):355-6.

[CD006103-bib-0311] Koegelenberg CF. Efficacy of varenicline combined with nicotine replacement therapy vs varenicline alone for smoking cessation: A randomized clinical trial. [German]. Zeitschrift fur Gefassmedizin 2014;11(3):26. [CENTRAL: 994156] [EMBASE: 2014813108] [PMID: 25005652] 25005652 10.1001/jama.2014.7195

[CD006103-bib-0312] Maliszewski L, Straczynski A. Therapeutic use of Tabex. Wiadomoscie Lekarskie 1972;25(24):2207-10. 4645090

[CD006103-bib-0313] Marakulin VS, Komarov VM, Chuprin VV. Treatment of nicotinism [Ob opyte lecheniia nikotinizma]. Voenno-Meditsinskii Zhurnal 1984;1:55-8. 6702122

[CD006103-bib-0314] McClure EA, Vandrey RG, Johnson MW, Stitzer ML. Effects of varenicline on abstinence and smoking reward following a programmed lapse. Nicotine & Tobacco Research 2013;15(1):139-48. [CENTRAL: 873078] [EMBASE: 2012756138] [PMID: 22573730] 22573730 10.1093/ntr/nts101 PMC3524062

[CD006103-bib-0315] NCT00944554. Varenicline for relapse prevention. ClinicalTrials.gov/ct2/ 2009 (accessed 2 November 2010).

[CD006103-bib-0316] McColl SL, Burstein AH, Reeves KR, Billing CB, Stolar M, Sellers EM. Human abuse liability of the smoking cessation drug varenicline in smokers and nonsmokers. Clinical Therapeutics and Pharmacology 2008;83(4):607-14. 10.1038/sj.clpt.6100510 18288085

[CD006103-bib-0317] McNaughton B, Frohlich J, Graham A, Young QR. Extended interactive voice response telephony (IVR) for relapse prevention after smoking cessation using varenicline and IVR: a pilot study. BMC Public Health 2013;13:824. [CENTRAL: 1015643] 10.1186/1471-2458-13-824 PMC3848019 24020450

[CD006103-bib-0318] Meszaros ZS, Abdul-Malak Y, Dimmock JA, Wang D, Ajagbe TO, Batki SL. Varenicline treatment of concurrent alcohol and nicotine dependence in schizophrenia: a randomized, placebo-controlled pilot trial. Journal of Clinical Psychopharmacology 2013;33(2):243-7. [CENTRAL: 908808] [EMBASE: 2013155799] [PMID: 23422399] 23422399 10.1097/JCP.0b013e3182870551

[CD006103-bib-0319] Meszaros ZS, Abdul-Malak Y, Dimmock JA, Wang D, Batki SL. Varenicline treatment of alcohol and nicotine dependence in schizophrenia: problems encountered in a pilot trial. American Journal on Addictions / American Academy of Psychiatrists in Alcoholism and Addictions 2012;21(4):393-4. [CENTRAL: 834600] [EMBASE: 70815316]

[CD006103-bib-0320] NCT00727103. Varenicline treatment in alcohol and nicotine dependent patients with schizophrenia. clinicaltrials.gov/ct2/show/NCT00727103 (first received 1 August 2008).

[CD006103-bib-0321] Metelitsa VI. Pharmacological agents in controlling smoking. Biulleten Vsesoiuznogo Kardiologicheskogo Nuachnogo Tsentra AMN SSSR 1987;10(1):109-12. 3606829

[CD006103-bib-0322] Mitchell JM, Teague CH, Kayser AS, Bartlett SE, Fields HL. Varenicline decreases alcohol consumption in heavy-drinking smokers. Psychopharmacology 2012;223(3):299-306. [CENTRAL: 845347] [PMID: 22547331] 22547331 10.1007/s00213-012-2717-x PMC3438402

[CD006103-bib-0323] Mocking RJ, Patrick Pflanz C, Pringle A, Parsons E, McTavish SF, Cowen PJ, et al. Effects of short-term varenicline administration on emotional and cognitive processing in healthy, non-smoking adults: a randomized, double-blind, study. Neuropsychopharmacology 2013;38(3):476-84. 10.1038/npp.2012.205 PMC3547198 23072834

[CD006103-bib-0324] Monova A, Monova D, Petrov V, Peneva E, Todorova M, Petrova M. Final report on double blind, placebo controlled, randomized clinical study for evaluation of efficacy, safety and tolerability of Tabex in patients with chronic nicotinism (tabacism): TAB - SPH - 04014. Sopharma plc unpublished report 2004.

[CD006103-bib-0325] Nahvi S, Segal KS, Litwin AH, Arnsten JH. Rationale and design of a randomized controlled trial of varenicline directly observed therapy delivered in methadone clinics. Addiction Science & Clinical Practice 2014;9:9. [CENTRAL: 1037892] 10.1186/1940-0640-9-9 PMC4084498 24928218

[CD006103-bib-0326] Nahvi S, Adams TR, Ning Y, Zhang C, Arnsten JH. Effect of varenicline directly observed therapy versus varenicline self-administered therapy on varenicline adherence and smoking cessation in methadone-maintained smokers: a randomized controlled trial. Addiction 2020;116(4):902-13. 10.1111/add.15240 PMC7983847 32857445

[CD006103-bib-0327] NCT00387946. Efficacy and safety of dianicline treatment as an aid to smoking cessation in cigarette smokers (AMERIDIAN). clinicaltrials.gov/ct2/show/NCT00387946 (first received 13 October 2006).

[CD006103-bib-0328] NCT00502216. Naltrexone and varenicline: weight gain and tolerability in smokers. ClinicalTrials.gov/show/NCT00502216 (first received 17 July 2007).

[CD006103-bib-0329] NCT00554840. Comparison of varenicline and placebo for smoking cessation in schizophrenia. ClinicalTrials.gov/ct2/show/NCT00554840 (first received 7 November 2007).

[CD006103-bib-0330] NCT00828113. Long-term varenicline treatment for smoking cessation. clinicaltrials.gov/ct2/show/NCT00828113 (first received 23 January 2009).

[CD006103-bib-0331] NCT01093937. Study of varenicline for smoking cessation/reduction in patients with bipolar disorder. clinicaltrials.gov/ct2/show/NCT01093937 (first received 26 March 2010).

[CD006103-bib-0332] NCT01413516. A two-part pilot study of dosing, safety and efficacy of varenicline initiated during an acute smoke-free hospitalization and continued post-hospitalization. clinicaltrials.gov/show/NCT01413516 (first received 10 August 2011).

[CD006103-bib-0333] NCT01532232. Treatment of tobacco dependence in breast cancer patients: a randomized trial of varenicline (Chantix). ClinicalTrials.gov/show/NCT01532232 (first received 14 February 2012).

[CD006103-bib-0334] NCT01574703. Study to evaluate cardiac assessments following different treatments of smoking cessation medications in subjects with and without psychiatric disorders [CATS]. ClinicalTrials.gov/show/NCT01574703 (first received 10 April 2012).

[CD006103-bib-0335] NCT01592695. Tailored tobacco cessation program for rural veterans with comorbid depression, alcoholism or obesity. ClinicalTrials.gov/show/NCT01592695 (first received 7 May 2012).

[CD006103-bib-0336] NCT01639560. Varenicline for light smokers (ChanLight). clinicaltrials.gov/ct2/show/NCT01639560 (first received 12 July 2012).

[CD006103-bib-0337] NCT01771627. Pilot study of varenicline vs. nicotine patch delivered by a telephone quitline to promote smoking cessation. ClinicalTrials.gov/show/NCT01771627 (first received 18 January 2013).

[CD006103-bib-0338] NCT01772641. A combination of scheduled reduced smoking with varenicline to enhance cessation. ClinicalTrials.gov/show/NCT01772641 (first received 21 January 2013).

[CD006103-bib-0339] NCT01806779. Combination bupropion/varenicline for smoking cessation in male smokers. ClinicalTrials.gov/show/NCT01806779 (first received 7 March 2013).

[CD006103-bib-0340] NCT01892813. Tailored tobacco intervention. ClinicalTrials.gov/show/NCT01892813 (first received 4 July 2013).

[CD006103-bib-0341] NCT02048917. Optimization of smoking cessation strategies in community cancer programs for newly diagnosed lung and head and neck cancer patients. Clinicaltrials.gov/show/NCT02048917 (first received 29 January 2014). [CENTRAL: 1013511]

[CD006103-bib-0342] NCT02147132. A pilot randomized, placebo-controlled, crossover study of the effect of the nicotine nasal spray and varenicline on cigarette smoking following methadone dosing in methadone-maintained patients. ClinicalTrials.gov/show/NCT02147132 (first received 26 May 2014).

[CD006103-bib-0343] NCT02271919. Varenicline and combined nicotine replacement therapy (NRT) for initial smoking cessation and rescue Treatment in smokers: a randomized pilot trial. ClinicalTrials.gov/show/NCT02271919 (first received 22 October 2014).

[CD006103-bib-0344] NCT02501265. Adaptive pharmacotherapy for smoking cessation. https://clinicaltrials.gov/show/NCT02501265 (first received 17 July 2015).

[CD006103-bib-0345] NCT03709823. Trial of cytisine in adult smokers. clinicaltrials.gov/ct2/show/NCT03709823 (first received 17 October 2018).

[CD006103-bib-0346] Nides M, Rigotti NA, Benowitz N, Clarke A, Jacobs C. A multicenter, double-blind, randomized, placebo-controlled phase 2b trial of cytisinicline in adult smokers (the ORCA-1 trial). Nicotine & Tobacco Research 2021;23(10):1656-63. 10.1093/ntr/ntab073 PMC8403245 33847362

[CD006103-bib-0347] Buchanan TS, Berg CJ, Cox LS, Nazir N, Benowitz NL, Yu L, et al. Adherence to varenicline among African American smokers: an exploratory analysis comparing plasma concentration, pill count, and self-report. Nicotine & Tobacco Research 2012;14(9):1083-91. [CENTRAL: 834603] [EMBASE: 2012554324] [PMID: 22367976] 22367976 10.1093/ntr/ntr333 PMC3432278

[CD006103-bib-0348] Nollen NL, Cox LS, Nazir N, Ellerbeck EF, Owen A, Pankey S, et al. A pilot clinical trial of varenicline for smoking cessation in Black smokers. Nicotine & Tobacco Research 2011;13(9):868-73. 10.1093/ntr/ntr063 PMC3203399 21498427

[CD006103-bib-0349] Ostrovskaia TP. Results of clinical investigation of anti-nicotine drug patches. Meditsinskaia Tekhnika 1994;3:42-3. 7934734

[CD006103-bib-0350] Park ER, Japuntich S, Temel J, Lanuti M, Pandiscio J, Hilgenberg J, et al. A smoking cessation intervention for thoracic surgery and oncology clinics. Journal of Thoracic Oncology 2011;6(6):1059-65. 10.1097/JTO.0b013e318215a4dc PMC3104083 21512406

[CD006103-bib-0351] NCT00948649. Effects of Chantix on relapse prevention for smoking cessation. clinicaltrials.gov/ct2/show/NCT00948649 (first received 29 July 2009).

[CD006103-bib-0352] Patterson F, Jepson C, Loughead J, Perkins K, Strasser AA, Siegel S, et al. Working memory deficits predict short-term smoking resumption following brief abstinence. Drug and Alcohol Dependence 2010;106(1):61-4. [CENTRAL: 731184] [PMID: 19733449] 19733449 10.1016/j.drugalcdep.2009.07.020 PMC2815089

[CD006103-bib-0353] Patterson F, Jepson C, Strasser AA, Loughead J, Perkins KA, Gur RC, et al. Varenicline improves mood and cognition during smoking abstinence. Biological Psychiatry 2009;65(2):144-9. [CENTRAL: 687104] [PMID: 18842256] 18842256 10.1016/j.biopsych.2008.08.028 PMC2615779

[CD006103-bib-0354] Rudnick ND, Strasser AA, Phillips JM, Jepson C, Patterson F, Frey JM, et al. Mouse model predicts effects of smoking and varenicline on event-related potentials in humans. Nicotine & Tobacco Research 2010;12(6):589-97. [CENTRAL: 751949] [PMID: 20395358] 20395358 10.1093/ntr/ntq049 PMC2878727

[CD006103-bib-0355] Paun D, Franze J. Smoking cessation with cytisine 'Tabex' tablets [Raucherentwöhnung mit cytisinhaltigen "Tabex" tabletten]. Sonderduck Aus Das Deutsche Gesundheitwesen 1968;23(44):2088-91. 5730917

[CD006103-bib-0356] Paun D, Franze Y. Tabex: registering and treatment of smokers with chronic bronchitis in the consultation against tobacco smoking - Berlin. Medico-Biologic Information 1970;3:15-9.

[CD006103-bib-0357] Pfeifer P, Fehr C. Efficacy of varenicline in patients with severe alcohol dependence: a pilot double-blind randomized and controlled study. Journal of Clinical Psychopharmacology 2019;39(4):398-402. 10.1097/JCP.0000000000001056 31188242

[CD006103-bib-0358] Pfizer Inc. Flexible dosing trial. NDA 21-928 [reported in CDER 2006] 2006.

[CD006103-bib-0359] Poling J, Rounsaville B, Gonsai K, Severino K, Sofuoglu M. The safety and efficacy of varenicline in cocaine using smokers maintained on methadone: a pilot study. American Journal on Addictions 2010;19:401-8. 10.1111/j.1521-0391.2010.00066.x PMC2966972 20716302

[CD006103-bib-0360] Ramon JM, Morchon S, Baena A, Masuet-Aumatell C. Combining varenicline and nicotine patches: a randomized controlled trial study in smoking cessation. BMC Medicine 2014;12(1):172. [CENTRAL: 1066789] 10.1186/s12916-014-0172-8 PMC4198796 25296623

[CD006103-bib-0361] NCT01303861. Concurrent bupropion/varenicline for smoking cessation (ConNic4). clinicaltrials.gov/ct2/show/NCT01303861 (first received 25 February 2011).

[CD006103-bib-0362] Rose JE, Behm FM. Combination treatment with varenicline and bupropion in an adaptive smoking cessation paradigm. American Journal of Psychiatry 2014;171(11):1199-205. [CENTRAL: 1021652] [EMBASE: 2014905509] [PMID: 24934962] 24934962 10.1176/appi.ajp.2014.13050595 PMC4557205

[CD006103-bib-0363] Rose JE, Behm FM. Combination varenicline/bupropion treatment benefits male NRT-nonresponders. Society for Research on Nicotine and Tobacco 19th Annual Meeting March 13-16 Boston MA 2013:261.

[CD006103-bib-0364] Schlienz NJ, Hawk LWJ, Tiffany ST, O'Connor RJ, Mahoney MC. The impact of pre-cessation varenicline on behavioral economic indices of smoking reinforcement. Addictive Behaviors 2014;39(10):1484-90. [CENTRAL: 993961] [EMBASE: 2014413435] [PMID: 24949949] 24949949 10.1016/j.addbeh.2014.05.008 PMC4109268

[CD006103-bib-0365] Schmidt F. Medical support of nicotine withdrawal. Report on a double blind trial in over 5000 smokers (author's transl) [Medikamentose unterstutzung der raucherentwohnung: bericht uber versuche an uber 5000 rauchern im doppelblindversuch]. Munch Med Wachr 1974;116(11):557-64. 4208732

[CD006103-bib-0366] Schnoll RA, Cappella J, Lerman C, Pinto A, Patterson F, Wileyto EP, et al. A novel recruitment message to increase enrollment into a smoking cessation treatment program: preliminary results from a randomized trial. Health Communication 2011;26(8):735-42. 10.1080/10410236.2011.566829 PMC4243839 21667366

[CD006103-bib-0367] Shim JC, Jung D, Oh M, Kong B, Ha T, Cho D, et al. Varenicline treatment for smoking cessation in people with schizophrenia: a randomized double-blind placebo-controlled trial. Schizophrenia Bulletin 2011;37:320-1.

[CD006103-bib-0368] Shim JC, Jung DU, Jung SS, Seo YS, Cho DM, Lee JH, et al. Adjunctive varenicline treatment with antipsychotic medications for cognitive impairments in people with schizophrenia: a randomized double-blind placebo-controlled trial. Neuropsychopharmacology 2012;37(2):660-8. 10.1038/npp.2011.238 PMC3260973 22048460

[CD006103-bib-0369] Sicras-Mainar A, Navarro-Artieda R, Diaz-Cerezo S, Sanz de Burgoa V. Effectiveness of varenicline compared with bupropion and nicotine replacement therapy (NRT) for smoking cessation in two smoking specialized units in the primary care setting. Society for Research on Nicotine and Tobacco Europe Conference: Bath, UK, 6-9 October 2010.

[CD006103-bib-0370] NCT00802919. Varenicline for cognitive deficits and cigarette smoking in schizophrenia. ClinicalTrials.gov/ct2/show/NCT00802919 (first received 5 December 2008).

[CD006103-bib-0371] Smith RC, Amiaz R, Mei S, Maayan L, Jin H, Boules S, et al. Varenicline effects on smoking, cognition, and psychiatric symptoms in schizophrenia. Neuropsychopharmacology 2013;38:S364-5. [CENTRAL: 990503] [EMBASE: 71278474]

[CD006103-bib-0372] Stapleton JA, Watson L, Spirling LI, Smith R, Milbrandt A, Ratcliffe M, et al. Varenicline in the routine treatment of tobacco dependence: a pre-post comparison with nicotine replacement therapy and an evaluation in those with mental illness. Addiction 2008;103(1):146-54. 10.1111/j.1360-0443.2007.02083.x 18028247

[CD006103-bib-0373] Stoyanov S, Yanachkova M. On the therapeutic effectiveness and tolerability of Tabex [Bulgarian]. Savremenna Medicina 1972;23(6):30-3.

[CD006103-bib-0374] Catz SL, Jack LM, McClure JB, Javitz HS, Deprey M, Zbikowski SM, et al. Adherence to varenicline in the COMPASS smoking cessation intervention trial. Nicotine & Tobacco Research 2011;13(5):361-8. 10.1093/ntr/ntr003 PMC3082504 21350041

[CD006103-bib-0375] Halperin AC, McAfee TA, Jack LM, Catz SL, McClure JB, Deprey TM, et al. Impact of symptoms experienced by varenicline users on tobacco treatment in a real world setting. Journal of Substance Abuse Treatment 2009;36:428-34. 10.1016/j.jsat.2008.09.001 PMC2776715 19004600

[CD006103-bib-0376] McClure JB, Swan GE, Catz SL, Jack L, Javitz H, McAfee T, et al. Smoking outcome by psychiatric history after behavioral and varenicline treatment. Journal of Substance Abuse and Treatment 2010;38:394-402. 10.1016/j.jsat.2010.03.007 PMC2860053 20363092

[CD006103-bib-0377] McClure JB, Swan GE, Jack L, Catz SL, Zbikowski SM, McAfee TA, et al. Mood, side-effects and smoking outcomes among persons with and without probably lifetime depression taking varenicline. Journal of General Internal Medicine 2009;24:563-9. 10.1007/s11606-009-0926-8 PMC2669860 19238488

[CD006103-bib-0378] Swan GE, McClure JB, Jack LM, Zbikowski SM, Javitz HS, Catz SL, et al. Behavioral counseling and varenicline treatment for smoking cessation. American Journal of Preventive Medicine 2010;38(5):482-90. 10.1016/j.amepre.2010.01.024 PMC2879135 20409497

[CD006103-bib-0379] Zbikowski SM, Jack LM, McClure JB, Deprey M, Javitz HS, McAfee TA, et al. Utilization of services in a randomized trial testing phone- and web-based interventions for smoking cessation. Nicotine & Tobacco Research 2011;13(5):319-27. 10.1093/ntr/ntq257 PMC3082503 21330267

[CD006103-bib-0380] Bolin K, Mörk A-C, Wilson K. Smoking-cessation therapy using varenicline: the cost-utility of an additional 12-week course of varenicline for the maintenance of smoking abstinence. Journal of Evaluation in Clinical Practice 2009;15:478-85. 10.1111/j.1365-2753.2008.01045.x 19536915

[CD006103-bib-0381] Hajek P, Tønneson P, Arteaga C, Russ C, Tonstad S. Varenicline in prevention of relapse to smoking: effect of quit pattern on response to extended treatment. Addiction 2009;104:1597-602. 10.1111/j.1360-0443.2009.02646.x 19686530

[CD006103-bib-0382] Knight C, Howard P, Baker CL, Marton JP. The cost-effectiveness of an extended course (12 + 12 weeks) of varenicline compared with other available smoking cessation strategies in the United States: an extension and update to the BENESCO model. Value in Health 2010;13(2):209-14. 10.1111/j.1524-4733.2009.00672.x 19912599

[CD006103-bib-0383] Knight CJ, Howard PA, Baker CL. An evaluation of the cost-effectiveness of an extended course of varenicline in preventing smokers who have quit from relapsing [PSM3]. Value in Health 2007;10(6):A472.

[CD006103-bib-0384] Lee JH, Jones PG, Bybee K, O'Keefe JH. A longer course of varenicline therapy improves smoking cessation rates. Preventive Cardiology 2008;11(4):210-4. 10.1111/j.1751-7141.2008.00003.x 19476573

[CD006103-bib-0385] O'Brien CP. Varenicline as maintenance therapy. Current Psychiatry Reports 2007;9(5):348-8. 10.1007/s11920-007-0044-4 17915072

[CD006103-bib-0386] Tonstad S, Tønnesen P, Hajek P, Williams KE, Billing CB, Reeves KR. Effect of maintenance therapy with varenicline on smoking cessation: a randomized controlled trial. JAMA 2006;296(1):64-71. [clinicaltrials.gov ID: NCT00143286] 10.1001/jama.296.1.64 16820548

[CD006103-bib-0387] NCT00977249. Varenicline for long-term NRT users. clinicaltrials.gov/ct2/show/NCT00977249 (first received 15 September 2009).

[CD006103-bib-0388] Tønnesen P, Mikkelsen K. Varenicline to stop long-term nicotine replacement use: a double-blind, randomized, placebo-controlled trial. Nicotine & Tobacco Research 2013;15(2):419-27. [CENTRAL: 861602] [EMBASE: 2013064611] [PMID: 23024246] 23024246 10.1093/ntr/nts146

[CD006103-bib-0389] Weiner E, Buchholz A, Coffay A, Liu F, McMahon RP, Buchanan RW, et al. Varenicline for smoking cessation in people with schizophrenia: a double blind randomized pilot study. Schizophrenia Research 2011;129:94-5. 10.1016/j.schres.2011.02.003 PMC3691956 21376537

[CD006103-bib-0390] Zatonski W, Cedzynska M, Przewozniak K, Karpinska E, Lewandowska D, Pstrucha E, et al. An open label observational study of herbal cytisine (Tabex) as an aid to smoking cessation [POS1-058]. Society for Research on Nicotine and Tobacco 11th Annual Meeting, 20-23 March 2005; Prague, Czech Republic 2005.

[CD006103-bib-0391] Zatonski W, Cedzynska M, Tutka P, West R. An uncontrolled trial of cytisine (Tabex) for smoking cessation. Tobacco Control 2006;15(6):481-4. 10.1136/tc.2006.016097 PMC2563682 17130378

[CD006103-bib-0392] Wiratmoko MR, Yunus F, DSusanto A, Ginting TT, Kekalih A. Efficacy of varenicline, an nicotinic acetylcholine receptor partial agonist, vs placebo for smoking cessation. A randomized controlled trial. Respirology 2013;18 Suppl 4:66 [OS205]. [CENTRAL: 989836] [EMBASE: 71371580]

[CD006103-bib-0393] Yujie W, Huliang L. Efficacy and safety of varenicline for smoking cessation in patients with CAD undergoing PCI. Journal of the American College of Cardiology 2014;64(16 Suppl 1):C207. [CENTRAL: 1054389] [EMBASE: 71665049]

[CD006103-bib-0394] Berlin I, Dautzenberg B, Lehmann B, Palmyre J, Liegey E, De Rycke Y, et al. Randomised, placebo-controlled, double-blind, double-dummy, multicentre trial comparing electronic cigarettes with nicotine to varenicline and to electronic cigarettes without nicotine: the ECSMOKE trial protocol. BMJ Open 2019;9(5):e028832. 10.1136/bmjopen-2018-028832 PMC6538086 31129603

[CD006103-bib-0395] NCT03630614. Randomized trial of electronic cigarettes with or without nicotine in smoking cessation. clinicaltrials.gov/ct2/show/NCT03630614 (first received 15 August 2018).

[CD006103-bib-0396] Caponnetto P, Maglia M, Polosa R. Efficacy of smoking cessation with varenicline plus counselling for e-cigarettes users (VAREVAPE): a protocol for a randomized controlled trial. Contemporary Clinical Trials Communications 2019;15:100412. 10.1016/j.conctc.2019.100412 PMC6667779 31388599

[CD006103-bib-0397] ChiCTR1900021400. Individual tobacco cessation research based on nicotine metabolite ratio in smoking patients with chronic obstructive pulmonary disease: a randomized controlled trial. www.who.int/trialsearch/Trial2.aspx?TrialID=ChiCTR1900021400 2019.

[CD006103-bib-0398] IRCT20200719048133N1. Effect of varenicline in smoking cessation after invasive treatment of coronary artery disease. en.irct.ir/trial/49723 (first received 23 July 2020).

[CD006103-bib-0399] Lawson SC, Gass JC, Cooper RK Jr, Tonkin SS, Colder CR, Mahoney MC, et al. The impact of three weeks of pre-quit varenicline on reinforcing value and craving for cigarettes in a laboratory choice procedure. Psychopharmacology 2021;238(2):599-609. 10.1007/s00213-020-05713-7 PMC10031567 33219852

[CD006103-bib-0400] NCT03262662. EVarQuit: extended pre-quit varenicline to assist in quitting smoking. clinicaltrials.gov/show/NCT03262662 (first received 25 August 2017).

[CD006103-bib-0401] NCT00906386. Methadone maintenance treatment and smoking cessation (MMTASC). clinicaltrials.gov/ct2/show/NCT00906386 (first received 21 May 2009).

[CD006103-bib-0402] NCT01243203. Smoking cessation program in the preadmission clinic: the use of a teachable moment. ClinicalTrials.gov/show/NCT01243203 (first received 18 November 2010).

[CD006103-bib-0403] NCT01312909. Smoking cessation study in healthy adolescent smokers. clinicaltrials.gov/ct2/show/NCT01312909 (first received 11 March 2011).

[CD006103-bib-0404] NCT01800019. The Canadian HIV quit smoking trial: tackling the co-morbidities of depression and cardiovascular disease in HIV+ smokers. ClinicalTrials.gov/show/NCT01800019 (first received 27 February 2013).

[CD006103-bib-0405] NCT02106637. Early in-hospital initiation of pharmacotherapy for smoking cessation, concomitant with nurse-led support, in patients after an acute coronary syndrome (ACS). ClinicalTrials.gov/show/NCT02106637 (first received 8 April 2014).

[CD006103-bib-0406] NCT02162849. The effects of behavioral counseling plus nicotine replacement therapy (NRT) or varenicline on smoking cessation among smokers high and low in intrinsic reward sensitivity. ClinicalTrials.gov/show/NCT02162849 (first received 13 June 2014).

[CD006103-bib-0407] NCT02378714. Behavioral activation and varenicline for smoking cessation in depressed smokers. clinicaltrials.gov/show/NCT02378714 (first received 4 March 2015).

[CD006103-bib-0408] NCT02460900. Optimizing smoking cessation for people with HIV/AIDS who smoke. clinicaltrials.gov/show/NCT02460900 (first received 3 June 2015).

[CD006103-bib-0409] NCT02856581. Management of tobacco treatment intervention in reducing surgical complications in patients with newly diagnosed lung cancer who smoke cigarettes. clinicaltrials.gov/show/NCT02856581 (first received 5 August 2016).

[CD006103-bib-0410] NCT02991781. Combined bio- and neuro- feedback vs. varenicline use for smoking cessation. clinicaltrials.gov/show/NCT02991781 (first received 14 December 2016).

[CD006103-bib-0411] NCT03365362. A trial of directly observed and long-term varenicline. clinicaltrials.gov/show/NCT03365362 (first received 7 December 2017).

[CD006103-bib-0412] NCT03557294. Varenicline OTC trial on efficacy and safety. clinicaltrials.gov/show/NCT03557294 (first received 15 June 2018).

[CD006103-bib-0413] NCT04011280. Novel pharmacotherapy approaches in smokers with serious mental illness. clinicaltrials.gov/show/NCT04011280 first received 8 July 2019).

[CD006103-bib-0414] NCT04015414. Varenicline versus cytisine for smoking cessation in primary care setting. clinicaltrials.gov/show/NCT04015414 (first received 11 July 2019).

[CD006103-bib-0415] NCT04188873. Cessation screening project. clinicaltrials.gov/show/NCT04188873 (first received 6 December 2019).

[CD006103-bib-0416] NCT04525755. STARS (Smoking Treatment And Remote Sampling) study. clinicaltrials.gov/show/NCT04525755 (first received 25 August 2020).

[CD006103-bib-0417] NCT04604509. Nicotine replacement therapy, counseling, varenicline, and bupropion for smoking cessation, the PISCES I trial. clinicaltrials.gov/show/NCT04604509 (first received 27 October 2020).

[CD006103-bib-0418] NCT05102123. PeRiopEratiVE smokiNg cessaTion Trial (PREVENT). clinicaltrials.gov/show/NCT05102123 (first recieved 1 November 2021).

[CD006103-bib-0419] NCT05311085. Cytisine and e-cigarettes with supportive text-messaging for smoking cessation (Cess@Tion). clinicaltrials.gov/show/NCT05311085 (first received 5 April 2022).

[CD006103-bib-0420] NCT00959972. Varenicline versus transdermal nicotine patch for smoking cessation in patients with coronary heart disease. clinicaltrials.gov/ct2/show/NCT00959972 (first received 17 August 2009).

[CD006103-bib-0421] Reid RD, Armstrong A, McDonnell L, Aitken DA, Robert L, LaRue A, et al. Varenicline versus transdermal nicotine patch for smoking cessation in patients with coronary heart disease: a pilot randomized trial. Canadian Journal of Cardiology 2010;26 Suppl D:53D. [EMBASE: 70674067]

[CD006103-bib-0422] EUCTR2009-017599-26-IT. Efficacy and safety of smoking cessation with varenicline tartrate in diabetic smokers: a double-blind, placebo-controlled, randomized trial. www.clinicaltrialsregister.eu/ctr-search/search?query=2009-017599-26 (first received 22 January 2010).

[CD006103-bib-0423] NCT01387425. Efficacy and safety of smoking cessation with varenicline tartrate in diabetic smokers (DIASMOKE). clinicaltrials.gov/ct2/show/NCT01387425 (first received 4 July 2011).

[CD006103-bib-0424] Russo C, Caponnetto P, Cibella F, Maglia M, Alamo A, Campagna D, et al. A double blind randomized controlled trial investigating efficacy and safety of varenicline for smoking cessation in patients with type 2 diabetes: study protocol. Internal & Emergency Medicine 2021;16(7):1823-39. 10.1007/s11739-021-02684-1 PMC8502165 33735416

[CD006103-bib-0425] TCTR20180312001. Efficacy safety and health-related quality of life (HRQoL) of cytisine in smoking cessation. www.who.int/trialsearch/Trial2.aspx?TrialID=TCTR20180312001 (first received 2017).

[CD006103-bib-0426] NCT02797587. Studying partial-agonists for ethanol and tobacco elimination in Russians with HIV (St PETER HIV). clinicaltrials.gov/show/NCT02797587 (first received 13 June 2016).

[CD006103-bib-0427] Tindle HA, Freiberg MS, Gnatienko N, Blokhina E, Cheng DM, Yaroslavtseva T, et al. Design of a randomized controlled trial of smoking cessation medications for alcohol reduction among HIV-positive heavy drinkers and daily smokers in St. Petersburg, Russia. Contemporary Clinical Trials Communications 2020;19:100625. 10.1016/j.conctc.2020.100625 PMC7889999 33659761

[CD006103-bib-0428] Raw M, McNeill A, Arnott D. Varenicline: guidance for health professionals on a new prescription-only stop smoking medication. www.ash.org.uk/html/cessation/ASHVareniclineguidance.pdf (accessed November 2006).

[CD006103-bib-0429] Actions on Smoking and Health (ASH). Briefing: Health inequalities and smoking. ash.org.uk/uploads/ASH-Briefing_Health-Inequalities.pdf 2019.

[CD006103-bib-0430] Cahill K, Stevens S, Perera R, Lancaster T. Pharmacological interventions for smoking cessation: an overview and network meta-analysis. Cochrane Database of Systematic Reviews 2013, Issue 5. Art. No: CD009329. [DOI: 10.1002/14651858.CD009329.pub2] PMC8406789 23728690

[CD006103-bib-0431] Coe JW, Brooks PR, Vetelino MG, Wirtz MC, Arnold EP, Huang J, et al. Varenicline: an alpha4beta2 nAChR nicotinic receptor partial agonist for smoking cessation. Journal of Medicinal Chemistry 2005;48:3474-7. 10.1021/jm050069n 15887955

[CD006103-bib-0432] Davies NM, Thomas KH. The Food and Drug Administration and varenicline: should risk communication be improved? Addiction 2017;112(4):555-8. [DOI: 10.1111/add.13592.] PMC5347897 27558015

[CD006103-bib-0433] Deeks JJ, Higgins JP, Altman DG, editor(s). Chapter 10: Analysing data and undertaking meta-analyses. In: Higgins JP, Thomas J, Chandler J, Cumpston M, Li T, Page MJ, Welch VA, editor(s). Cochrane Handbook for Systematic Reviews of Interventions Version 6.3 (updated February 2022). Cochrane, 2022. Available from training.cochrane.org/handbook.

[CD006103-bib-0434] Ebbert JO, Montori VM, Erwin PJ, Stead LF. Interventions for smokeless tobacco use cessation. Cochrane Database of Systematic Reviews 2011, Issue 2. Art. No: CD004306. [DOI: 10.1002/14651858.CD004306.pub4] 21328266

[CD006103-bib-0435] Etter J-F. Cytisine for smoking cessation: a literature review and a meta-analysis. Archives of Internal Medicine 2006;166:1-7. 10.1001/archinte.166.15.1553 16908787

[CD006103-bib-0436] Etter JF, Burri M, Stapleton J. The impact of pharmaceutical company funding on results of randomized trials of nicotine replacement therapy for smoking cessation: a meta-analysis. Addiction 2007;102(5):815-22. 10.1111/j.1360-0443.2007.01822.x 17493109

[CD006103-bib-0437] Etter JF, Lukas RJ, Benowitz NL, West R, Dresler CM. Cytisine for smoking cessation: a research agenda. Drug and Alcohol Dependence 2008;92(1-3):3-8. 10.1016/j.drugalcdep.2007.06.017 17825502

[CD006103-bib-0438] Fabbri A, Nejstgaard CH, Grundy Q, Bero L, Dunn AG, Mohammad A, et al. Association between conflicts of interest and authors’ positions on harms of varenicline: a cross-sectional analysis. Journal of General Internal Medicine 2022;37(2):290-7. 10.1007/s11606-021-06915-1 PMC8811060 34037923

[CD006103-bib-0439] US Food and Drug Administration. FDA issues Public Health Advisory on Chantix. www.fda.gov/NewsEvents/Newsroom/PressAnnouncements/2008/ucm116849.htm (accessed 3 September 2015).

[CD006103-bib-0440] Foulds J, Burke M, Steinberg M, Williams JM, Ziedonis DM. Advances in pharmacotherapy for tobacco dependence. Expert Opinion on Emerging Drugs 2004;9(1):39-53. 10.1517/eoed.9.1.39.32951 15155135

[CD006103-bib-0441] Gotti C, Clementi F. Cytisine and cytisine derivatives. More than smoking cessation aids. Pharmacological Research 2021;170:105700. [DOI: 10.1016/j.phrs.2021.105700] 34087351

[CD006103-bib-0442] Higgins JP, Thompson SG, Deeks JJ, Altman DG. Measuring inconsistency in meta-analyses. BMJ 2003;7414:557-60. 10.1136/bmj.327.7414.557 PMC192859 12958120

[CD006103-bib-0443] Higgins JP, Altman DG, Sterne JA, editor(s). Chapter 8: Assessing risk of bias in included studies. In: Higgins JP, Green S, editor(s). Cochrane Handbook for Systematic Reviews of Interventions Version 5.1.0 (updated March 2011). The Cochrane Collaboration, 2011. Available from training.cochrane.org/handbook.

[CD006103-bib-0444] Hughes JR, Keely JP, Niaura RS, Ossip-Klein DJ, Richmond RL, Swan GE. Measures of abstinence in clinical trials: issues and recommendations. Nicotine & Tobacco Research 2003;5(1):13-25. 12745503

[CD006103-bib-0445] Kirchhoff VD, Nguyen HT, Soczynska JK, Woldeyohannes H, McIntyre RS. Discontinued psychiatric drugs in 2008. Expert Opinion on Investigational Drugs 2009;18(10):1431-43. 10.1517/13543780903184591 19715445

[CD006103-bib-0446] Leaviss J, Sullivan W, Ren S, Everson-Hock E, Stevenson M, Stevens JW, et al. What is the clinical effectiveness and cost-effectiveness of cytisine compared with varenicline for smoking cessation? a systematic review and economic evaluation. Health Technology Assessment 2014;18(33):1-120. 10.3310/hta18330 PMC4780997 24831822

[CD006103-bib-0447] Sterling LH, Windle SB, Filion KB, Touma L, Eisenberg MJ. Varenicline and adverse cardiovascular events: a systematic review and meta-analysis of randomized controlled trials. Journal of the American Heart Association 2016;5:e002849. [DOI: 10.1161/JAHA.115.002849] PMC4802486 26903004

[CD006103-bib-0448] Liberati A, Altman DG, Tetzlaff J, Mulrow C, Gotzsche PC, Ioannidis JP, et al. The PRISMA statement for reporting systematic reviews and meta-analyses of studies that evaluate health care interventions: explanation and elaboration. PLOS One 2009;6:e1000100. 10.1371/journal.pmed.1000100 PMC2707010 19621070

[CD006103-bib-0449] Lindson N, Theodoulou A, Livingstone-Banks J, Aveyard P, Fanshawe TR, Ordóñez-Mena JM, et al. Pharmacological and electronic cigarette interventions for smoking cessation in adults: component network meta‐analyses. Cochrane Database of Systematic Reviews 2022, Issue 3. Art. No: CD015226. [DOI: 10.1002/14651858.CD015226] PMC10495240 37696529

[CD006103-bib-0450] Lindson-Hawley N, Hartmann-Boyce J, Fanshawe TR, Begh R, Farley A, Lancaster T. Interventions to reduce harm from continued tobacco use. Cochrane Database of Systematic Reviews 2016, Issue 10. Art. No: CD005231. [DOI: 10.1002/14651858.CD005231.pub3] PMC6463938 27734465

[CD006103-bib-0451] Livingstone-Banks J, Norris E, Hartmann-Boyce J, West R, Jarvis M, Chubb E, et al. Relapse prevention interventions for smoking cessation. Cochrane Database of Systematic Reviews 2019, Issue 10. Art. No: CD003999. [DOI: 10.1002/14651858.CD003999.pub5] PMC6372978 30758045

[CD006103-bib-0452] Livingstone-Banks J, Lindson N, Hartmann-Boyce J, Aveyard P. Effects of interventions to combat tobacco addiction: Cochrane update of 2019 and 2020 reviews. Addiction 2022;117(6):1573-88. [DOI: 10.1111/add.15769] 34859525

[CD006103-bib-0453] Livingstone-Banks J, Siddiqui F, Croucher R, Mehrotra R, Vidyasagaran A, Siddiqi K. Interventions for smokeless tobacco use cessation. Cochrane Database of Systematic Reviews 2022, Issue 1. Art. No: CD015314. [DOI: 10.1002/14651858.CD015314] PMC11998898 40232040

[CD006103-bib-0454] Mills EJ, Wu P, Lockhart I, Thorlund K, Puhan M, Ebbert JO. Comparisons of high-dose and combination nicotine replacement therapy, varenicline and bupropion for smoking cessation: a systematic review and multiple-treatment meta-analysis. Annals of Medicine 2012;44(6):588-97. 10.3109/07853890.2012.705016 22860882

[CD006103-bib-0455] National Institute for Health and Clinical Excellence. Varenicline for smoking cessation. www.nice.org.uk/nicemedia/pdf/TA123Guidance.pdf (accessed 14th April 2008) 2007.

[CD006103-bib-0456] Rigotti NA, Kruse GR, Livingstone-Banks J, Hartmann-Boyce J. Treatment of tobacco smoking: a review. JAMA 2022;327(6):566-77. [10.1001/jama.2022.0395] 10.1001/jama.2022.0395 35133411

[CD006103-bib-0457] Schünemann H, Brożek J, Guyatt G, Oxman A, editor(s). Handbook for grading the quality of evidence and the strength of recommendations using the GRADE approach (updated October 2013). GRADE Working Group, 2013. Available from gdt.guidelinedevelopment.org/app/handbook/handbook.html.

[CD006103-bib-0458] Schünemann HJ, Higgins JP, Vist GE, Glasziou P, Akl EA, Skoetz N, et al. Chapter 14: Completing ‘Summary of findings’ tables and grading the certainty of the evidence. In: Higgins JP, Thomas J, Chandler J, Cumpston M, Li T, Page MJ, Welch VA editor(s). Cochrane Handbook for Systematic Reviews of Interventions Version 6.3 (updated February 2022). Cochrane, 2022. Available from www.training.cochrane.org/handbook.

[CD006103-bib-0459] Shiffman S, West R, Gilbert D. Recommendations for the assessment of tobacco craving and withdrawal in smoking cessation trials. Nicotine & Tobacco Research 2004;6(4):599-614. 10.1080/14622200410001734067 15370156

[CD006103-bib-0460] Stead LF, Hughes JR. Lobeline for smoking cessation. Cochrane Database of Systematic Reviews 2003, Issue 3. Art. No: CD000124. [DOI: 10.1002/14651858.CD000124] 10796490

[CD006103-bib-0461] Thomas KH, Martin RM, Knipe DW, Higgins JP, Gunnell D. Risk of neuropsychiatric adverse events associated with varenicline: systematic review and meta-analysis. BMJ 2015;350:h1109. [PROSPERO 2014:CRD42014009224] 10.1136/bmj.h1109 PMC4357491 25767129

[CD006103-bib-0462] Thomas, KH, Dalili, MN, López-López, JA, Keeney, E, Phillippo, DM, Munafò, MR, et al. Comparative clinical effectiveness and safety of tobacco cessation pharmacotherapies and electronic cigarettes: a systematic review and network meta-analysis of randomized controlled trials. Addiction 2020;117:861-76. [DOI: 10.1111/add.15675] PMC9293179 34636108

[CD006103-bib-0463] Tutka P, Zatoński W. Cytisine for the treatment of nicotine addiction: from a molecule to therapeutic efficacy. Pharmacological Reports 2005;58:777-98. 17220536

[CD006103-bib-0464] Tutka P, Mróz T, Zatoński W. Cytisine - renaissance of well known alkaloid. Pharmacological aspects of efficacy in the treatment of tobacco dependence [Cytyzyna – renesans znanego alkaloidu. Aspekty farmakologiczne zastosowania w leczeniu uzależnienia od nikotyny]. Farmakoterapia w Psychiatrii i Neurologii 2006;1:33-9.

[CD006103-bib-0465] Tutka P. Nicotinic receptor partial agonists as novel compounds for the treatment of smoking cessation. Expert Opinion on Investigational Drugs 2008;17(10):1473-85. 10.1517/13543784.17.10.1473 18808308

[CD006103-bib-0466] Tutka P, Vinnikov D, Courtney RJ, Benowitz NL. Cytisine for nicotine addiction treatment: a review of pharmacology, therapeutics and an update of clinical trial evidence for smoking cessation. Addiction 2019;114(11):1951-69. [DOI: 10.1111/add.14721] 31240783

[CD006103-bib-0467] US Department of Health and Human Services. Smoking cessation: a report of the Surgeon General. Atlanta, GA: U.S. Department of Health and Human Services, Centers for Disease Control and Prevention, National Center for Chronic Disease Prevention and Health Promotion, Office on Smoking and Health, 2020.

[CD006103-bib-0468] Walsh RA. Australia's experience with varenicline: usage, costs and adverse reactions [letter]. Addiction 2011;106:449-52. 10.1111/j.1360-0443.2010.03282.x 21208325

[CD006103-bib-0469] West R, Hajek P, Stead L, Stapleton J. Outcome criteria in smoking cessation trials: proposal for a common standard. Addiction 2005;100:299-303. 10.1111/j.1360-0443.2004.00995.x 15733243

[CD006103-bib-0470] World Health Organization. Two new tobacco cessation medicines added to the WHO essential medicines list. www.who.int/news/item/05-11-2021-two-new-tobacco-cessation-medicines-added-to-the-who-essential-medicines-list (accessed 16 November 2022).

[CD006103-bib-0471] World Health Organization. World Health Organization fact sheets: Tobacco. www.who.int/en/news-room/fact-sheets/detail/tobacco (accessed 16 November 2022).

[CD006103-bib-0472] Cahill K, Stead LF, Lancaster T. Nicotine receptor partial agonists for smoking cessation. Cochrane Database of Systematic Reviews 2007, Issue 1. Art. No: CD006103. [DOI: 10.1002/14651858.CD006103.pub2] 17253581

[CD006103-bib-0473] Cahill K, Stead LF, Lancaster T. Nicotine receptor partial agonists for smoking cessation. Cochrane Database of Systematic Reviews 2008, Issue 3. Art. No: CD006103. [DOI: 10.1002/14651858.CD006103.pub3] 17253581

[CD006103-bib-0474] Cahill K, Stead LF, Lancaster T. Nicotine receptor partial agonists for smoking cessation. Cochrane Database of Systematic Reviews 2010, Issue 2. Art. No: CD006103. [DOI: 10.1002/14651858.CD006103.pub5] 17253581

[CD006103-bib-0475] Cahill K, Stead LF, Lancaster T. Nicotine receptor partial agonists for smoking cessation. Cochrane Database of Systematic Reviews 2012, Issue 4. Art. No: CD006103. [DOI: 10.1002/14651858.CD006103.pub6] 17253581

[CD006103-bib-0476] Cahill K, Lindson-Hawley N, Thomas KH, Fanshawe TR, Lancaster T. Nicotine receptor partial agonists for smoking cessation. Cochrane Database of Systematic Reviews 2016, Issue 5. Art. No: CD006103. [DOI: 10.1002/14651858.CD006103.pub7] PMC6464943 27158893

[CD006103-bib-0477] Hey K, Lancaster T, Bala M. Nicotine receptor partial agonists for smoking cessation. Cochrane Database of Systematic Reviews 2006, Issue 3. Art. No: CD006103. [DOI: 10.1002/14651858.CD006103] 17253581

